# Alkanes in Minisci-Type Reaction under Photocatalytic Conditions with Hydrogen Evolution

**DOI:** 10.1021/acs.orglett.3c02619

**Published:** 2023-10-11

**Authors:** Loris Laze, Beatriz Quevedo-Flores, Irene Bosque, Jose C. Gonzalez-Gomez

**Affiliations:** Instituto de Síntesis Orgánica (ISO) and Departamento de Química Orgánica, Universidad de Alicante, 03080 Alacant, Spain

## Abstract

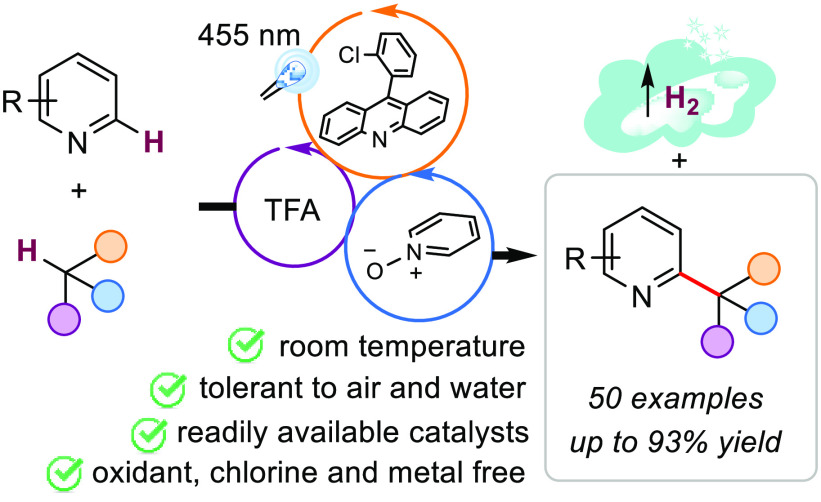

We report herein a protocol for the selective activation of C(*sp*^3^)–H bonds based on the interplay of two readily available organic catalysts and their successful implementation in cross-coupling azaarenes with alkanes. This Minisci-like reaction is promoted by visible light at room temperature and is free from chemical oxidants, metals, and chlorinated solvents. A wide range of substrates are compatible, including some bioactive molecules. Mechanistic studies support a dual catalytic cycle with H_2_ evolution.

Nitrogen heterocycles are abundant in natural products, agrochemicals, and pharmaceuticals.^1^ Most unique small-molecule drugs approved by FDA contain a nitrogen heterocycle.^2^ The straightforward alkylation of azaarenes is of pivotal importance in drug discovery,^3^ especially if late-stage functionalization of bioactive compounds is possible.^4^

The cross-dehydrogenative coupling (CDC) of azaarenes with alkanes has become one of the most appealing approaches to the Minisci reaction. This convergent strategy uses abundant feedstocks, avoiding prefunctionalized substrates.^5^ However, sacrificial oxidants are commonly required for this net oxidative process.^6^ In recent years, the use of photocatalysis,^7^ electrocatalysis,^8^ and electrophotocatalysis,^9^ has opened other access for radical generation from C(*sp*^3^)–H bonds.^10^ Very recently, the CDC of heteroarenes with alkanes has been accomplished without external chemical oxidants by the *in situ* generation of chlorine atoms (Cl^**•**^), either using photoelectrochemical^11^ or dual photocobalt-catalysis^12^ for the hydrogen evolution. In addition, photoinduced ligand-to-metal charge transfer has also been used to generate Cl^**•**^ and promote this transformation.^13^ These approaches exploit the high bond dissociation energy of HCl (BDE = 102 kcal/mol) to activate C(*sp*^3^)-H bonds by hydrogen atom transfer (HAT).^14^ In addition, diphenyl phosphate has also been successfully used in stoichiometric amounts as a HAT reagent to promote this transformation.^15^ Notably, this latter photochemical reaction was accomplished in 1,2-dichloroethane using a stop-flow microtubing reactor. Furthermore, it has been recently demonstrated that 1,2-dichloroethane can produce Cl^**•**^ under aerobic photocatalytic conditions, promoting the desired transformation.^16^ In fact, chlorinated solvents are prominent as reaction media for many organic transformations, including C–H activations, despite their serious health effects and environmental concerns.^17^ Therefore, *we proposed herein a user-friendly protocol for the CDC of azaarenes with alkanes where most of the previously commented issues are addressed* (Figure 1a).^18^

**Figure 1:**
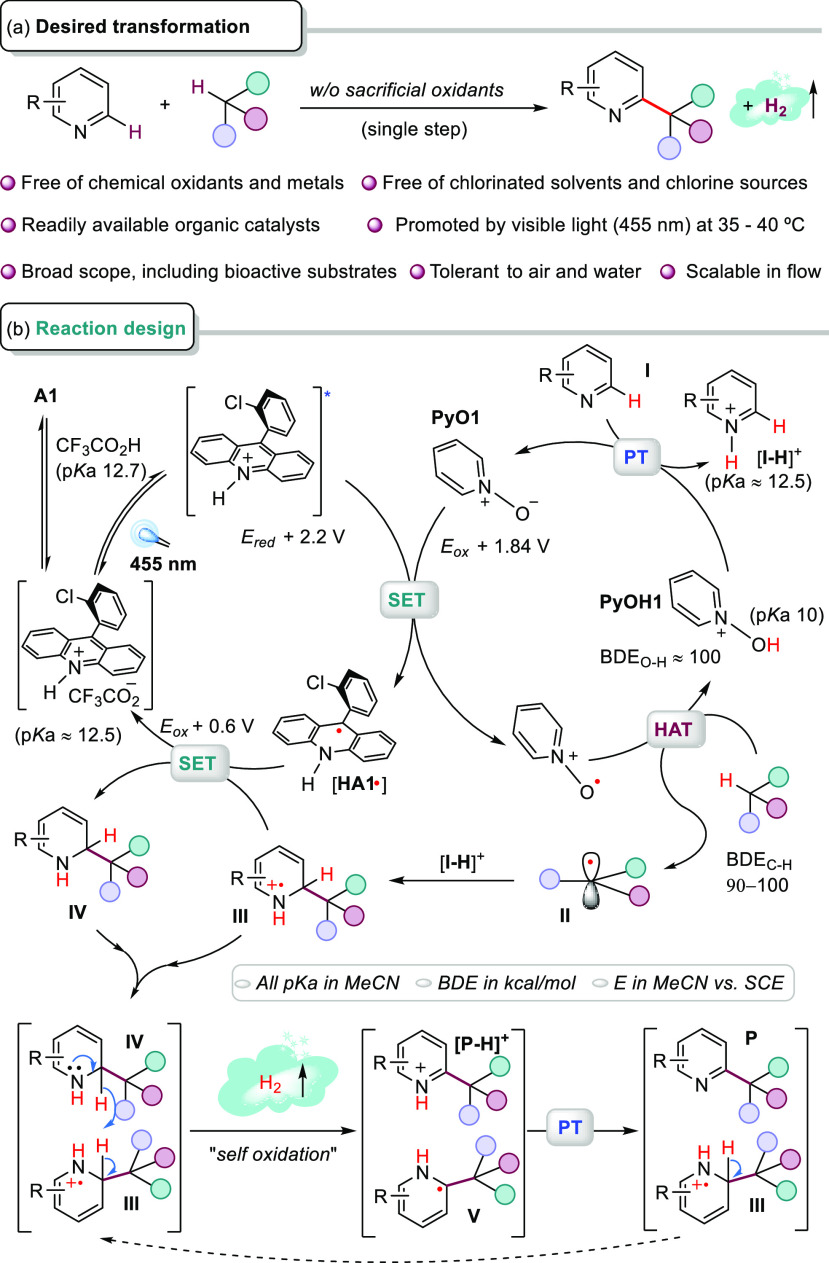
Desired transformation and reaction design.

Neutral 9-arylacridines were extensively used to promote photocatalytic decarboxylation of carboxylic acids through proton-coupled-electron transfer, and it is known that acridinium’s formed with trifluoroacetic acid (TFA) become photoactive with visible light.^19^ We thus hypothesized (Figure 1b) that, in the presence of TFA and blue light (455 nm), the excited state of the resulting acridinium (*E*_red_ 2.2 V vs. SCE)^20^ is oxidant enough to remove an electron from pyridine *N*-oxide (**PyO1**), without redox interference of the trifluoroacetate anion (*E*_ox_ > +2.25 V vs. SCE).^21^ The resulting *N*-oxyl radical could abstract hydrogen atoms from C(*sp*^3^)–H bonds^22^ forming **PyOH1**(23) that is significantly more acidic than TFA in MeCN^24^ and can be deprotonated by azaarenes to reset the **PyO1**. The resulting protonated azaarene **[I–H]**^**+**^ might add a transient nucleophilic radical (**II**) to obtain radical cation **III**, which after a single-electron-transfer (SET) with **HA1**^•^, would enable the turnover of **HA1**^**+**^ and the dihydroazaarene **IV**’s formation. As proposed by Kano and co-workers,^25^ intermediate **IV** could transfer a hydride to **III**, producing H_2_, the protonated product and radical **V**. A final proton transfer should deliver the product and intermediate **III**, which **HA1**^**•**^ or **IV** might quench. Our approach differs conceptually from Gryko’s protocol, where PyOs are used in stoichiometric amounts to form an EDA complex with the azaarene and more energetic photons (405 nm).^26^

To test our hypothesis, lepidine and cyclohexane were chosen as substrates, using commercial **PyO1** and acridine **A1** (prepared in one step, see SI) as organic catalysts. To our delight, the reaction was promoted by blue light irradiation (455 nm) under an argon atmosphere at room temperature (Table S1, entry 1). Following this encouraging result, we demonstrated that the reaction requires irradiation to proceed, that photocatalyst **A1** significantly improves the reaction yield, and that without deoxygenation, the reaction outcome was improved (entry 2). Given that ^3^O_2_ is a very efficient triplet quencher for acridinium salts,^27^ the performance of the reaction in the presence of air suggests the participation of the singlet-excited state of the photocatalyst.^19a^ Most importantly, this protocol is more user-friendly than others because inert gases or special equipment (glovebox, stop-flow microtubing reactor, etc.) are not required and the reaction is promoted at room temperature (30–35 °C). We thus found that 200 mol % of TFA, 25 mol % of **PyO1**, and 5 mol % of **A1** in 7:3 MeCN/HFIP with cyclohexane (5 equiv) and [lepidine] = 0.10 M were optimal reaction conditions. Although the reaction works in MeCN, using HFIP as a cosolvent might help solubilize **PyO1** and other polar substrates without redox interference.^28^ Increasing the load of **PyO1** slightly (30 mol %) allows the reaction to complete (entry 8). We also examined other PyOs and diphenyl phosphate as HAT catalysts and other 9-arylacridines in our model reaction (Table S2). However, poorer or similar results were obtained compared to those shown in entry 8 of Table S1.

Having found the optimal reaction conditions, we examined the substrate scope (Figure 2). Substituted quinolines reacted smoothly at C4 or C2 to obtain the cyclohexyl derivatives in moderate-to-good yields (**1**-**10**, 41%–93%), showing good functional group tolerance. Pyridines were also suitable substrates, mainly obtaining the monoalkylated products for *p*-Ph and *p*-CO_2_Et substrates (**11**, **12**) and the dialkylated product **13** with the more reactive *p*-CN pyridine. We also explored quinoxalin-2(1*H*)-ones,^29^ obtaining the desired products (**14**-**16**), showing good tolerance to nitro groups, albeit with larger excess of cyclohexane. Notably, 1,4-diazines reacted selectively to afford monoalkylated products (**17**, **18**) in good yields. We were pleased to observe that phenanthridine gave product **19** in an excellent yield. Benzothiazole and benzimidazole substrates gave products **20** and **21** in moderate yields. Some other azaarenes were recalcitrant substrates under our conditions (listed in Figure S19). Other cycloalkanes reacted with lepidine to give the desired products in good to excellent yields (**22**, **23**), even using lower excess of the alkane (3 equiv for **23**). Methylcyclopentane reacted mainly at the tertiary C–H bond and secondary bonds, with a normalized selectivity tertiary vs. secondary of 89% for isomers of **24**. Bridged alkanes also reacted smoothly, providing exclusively *exo*-norbornane derivative **25** and a 91:9 mixture of C1:C2-**26** from adamantane (97.6% normalized selectivity). The challenging acyclic alkanes exhibited excellent site-selectivity for tertiary C–H bonds (products **27**–**29**). The functionalization of benzylic C–H bonds was less efficient, furnishing products **30** and **31** in lower yields. Notably, *p*-cymene reacted with the least hindered benzylic C–H bond. Substrates with a short alkyl chain and electron-withdrawing groups have shown poor reactivity but excellent selectivity at the γ-CH position (valeronitrile → **32**; isoamyl acetate → **33**). When different amides were examined, only methylacetamide and pyrrolidinone gave the products **34** and **35** in low to moderate yields (failed substrates are shown in Figure S19). Cyclic ethers and acyclic methyl *tert*-butyl ether reacted selectively to give the corresponding α-heteroatom CDC products **36**–**38**. Notably, methanol reacts smoothly, providing the hydroxymethyl derivative **39** or its deuterated analog **40** in good yields, which complements the methylation observed under other photochemical conditions via the Spin-Center Shift (SCS) pathway.^30^ Increasing the steric demand at α-positions of alcohols decreased their reactivity, with ethanol providing **41** in 36% yield, while other branched alcohols failed, and isoamyl alcohol reacted mainly at the γ-position (**42**). The weakness of a C–H bond in a formyl group (BDE 88 kcal/mol) was used to direct the HAT event.^31^ However, the product depended on the substitution at the adjacent position, in contrast to primary 3-methylbutanal, which afforded product **43** via the SCS pathway, secondary/tertiary aldehydes provided alkylated heteroarenes **44** and **45** in good yields after decarbonylation. Our methodology was successfully applied to functionalizing natural nicotine, cinchonine, and quinine with cyclohexane, obtaining products **46**–**48** in reasonably good yields. These results illustrate the compatibility of *m*-substituted pyridines and the tolerance to diverse functional groups, such as tertiary amines, free hydroxyl groups, and terminal double bonds. Examining the functionalization of *O*-methylmenthol with lepidine, we found that only three equivalents were needed to obtain product **49** in good yield and excellent site-selectivity. Finally, when ambroxide was examined, selective H-abstraction next to the O atom was followed by radical addition to protonated lepidine and SCS with C–O bond cleavage, giving product **50** in good yield.

**Figure 2:**
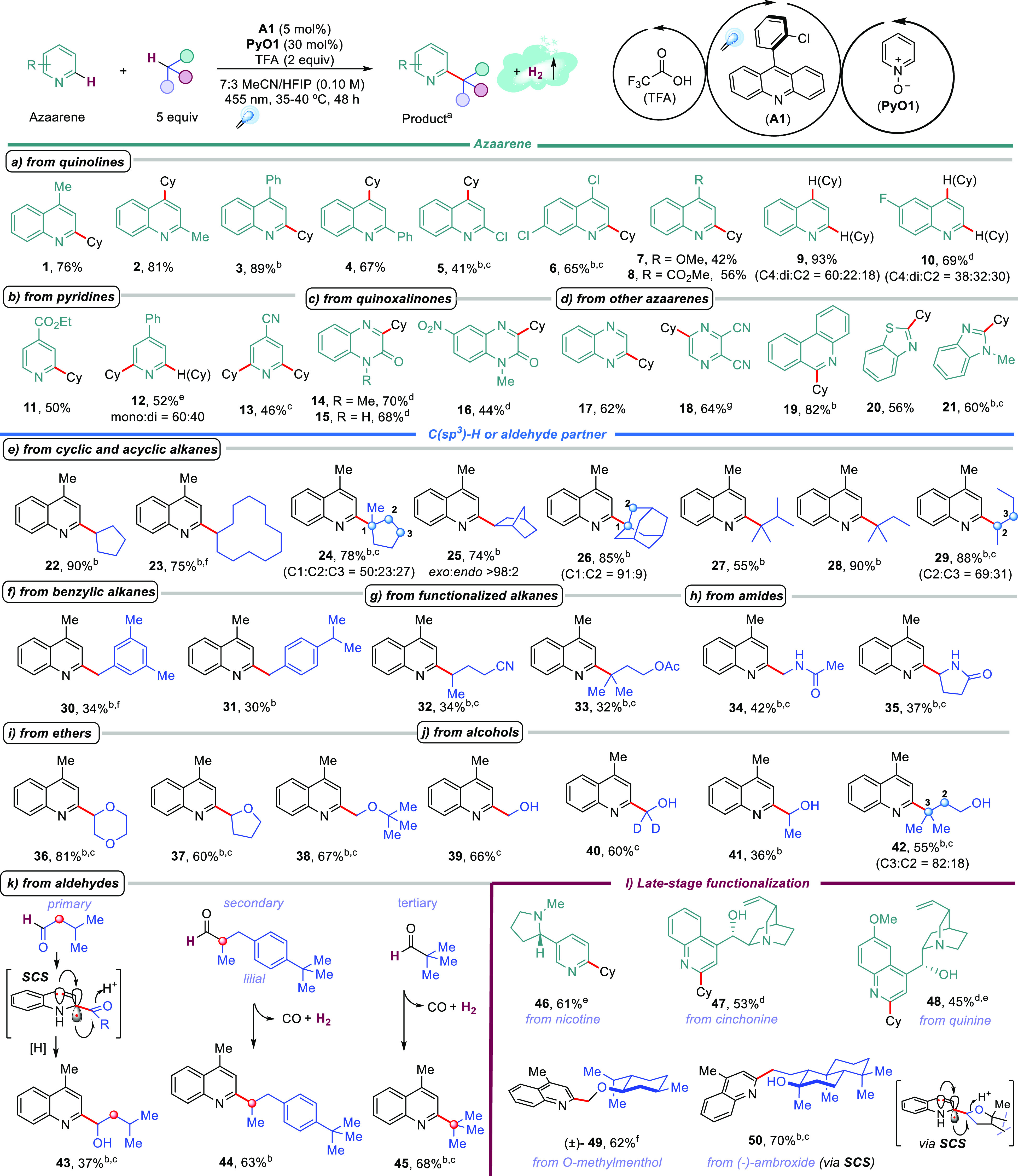
Substrate scope. ^a^Yields for isolated pure products are given. ^b^**PyO1** was added in two portions, 20 mol % at the beginning and 10 mol % after 24 h. ^c^10 equiv of R-H. ^d^23 equiv of R-H. ^e^3 equiv of TFA. ^f^3 equiv of R-H. ^g^4 equiv of TFA.

Preliminary mechanistic studies (Figure 3) support the proposed catalytic cycles shown in Figure 1b. Radical trapping experiments with TEMPO or 1,1-diphenylethene demonstrated the formation of cyclohexyl radical, likely through HAT from cyclohexane to the pyridine-*N*-oxyl radical (Figure 3a). The quantum yield of the reaction during the first 30 min is significantly below 1 (Figure 3b) to ensure that either the radical chain propagation is inefficient or the reaction takes place through a closed photoredox cycle. The reaction profile (Figure S1) shows that it is much faster in the initial stages. Therefore, the quantum yield should be lower after the first hours, and a closed photoredox cycle is more plausible. We prepared 2-cyclohexylbenzothiazole (**20**) from the corresponding hydrogenated **20-H**_**2**_ under the standard reaction conditions (Figure 3c), which supports the intermediacy of these compounds and their dehydrogenation. Remarkably, gas evolution was observed during the experiments in flow (Figure S14), and the formation of H_2_ was further confirmed by GC-TCD analysis (Figure 3d and Figure S13).

**Figure 3:**
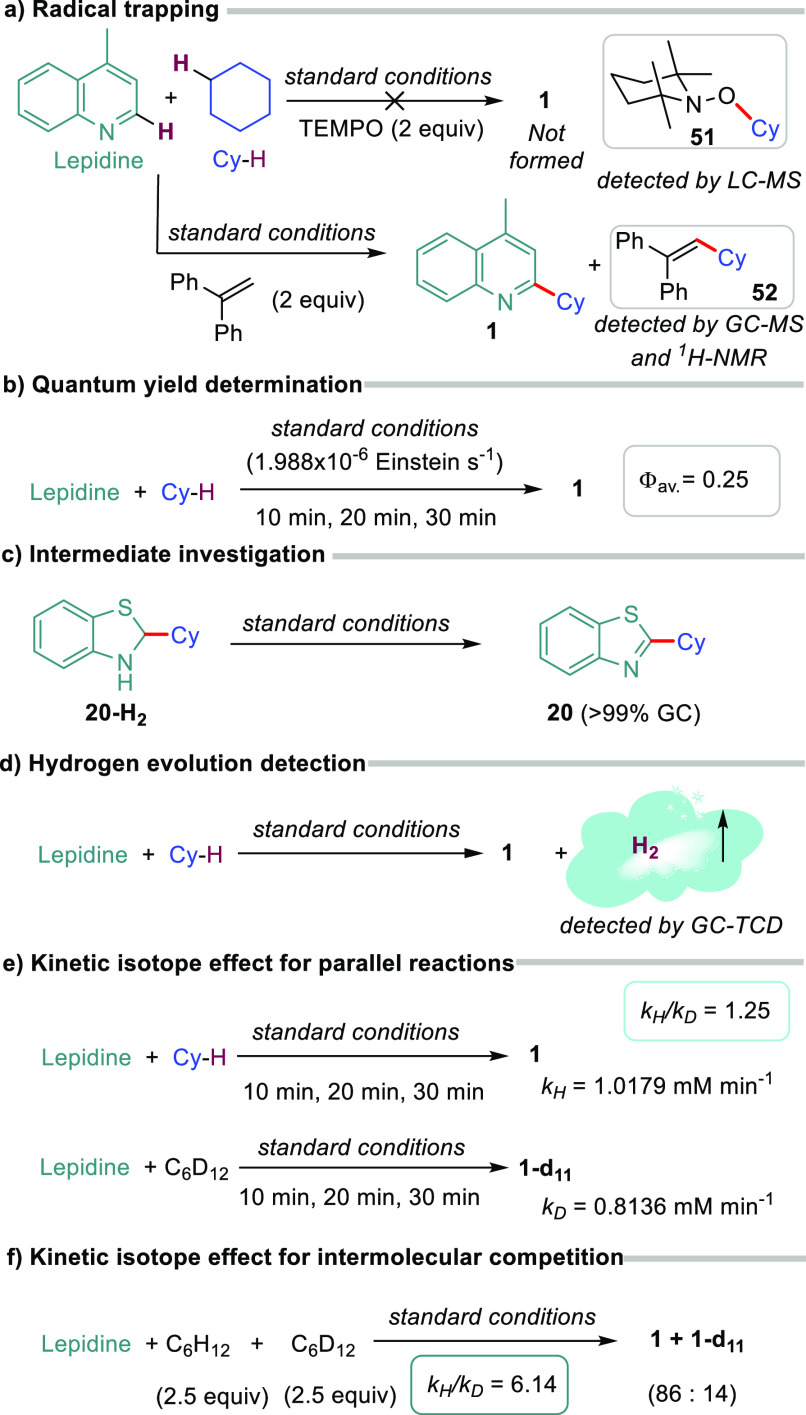
Mechanistic studies.

The UV–vis spectra of all of the reaction components confirmed that none of them absorbed light at 455 nm. Still, adding TFA to a solution of **A1** caused a significantly increased absorption between 390 and 460 nm (Figures S7–S8). Additionally, Stern–Volmer quenching experiments of a solution of **A1** and an excess of TFA (Figure S6) show that **PyO1** is the best single quencher from all reaction components, which is consistent with a SET from **PyO1** to [**HA1**]^+*^. The deuterium kinetic isotope effects (KIEs) were determined from two parallel reactions to obtain **1**/**1-d**_**11**_ (Figure 3e) and a competition experiment (Figure 3f), giving *k*_H_/*k*_D_ = 1.25 and 6.14, respectively. The same study for forming product **20** (Figures S17 and S18) afforded *k*_H_/*k*_D_ = 1.64 and 3, respectively. The difference obtained for the KIEs suggests that the alkyl radical formation via HAT is product-determining but not the turnover-determining step.^32^

To showcase the synthetic utility of our protocol, we took advantage of the homogeneous reaction mixture to scale up the process using continuous flow for better light harvesting.^33^ Our target was 4,7-dichloro-2-cyclohexylquinoline (**6**) because it can be readily transformed into different 4-aminoquinolines (Figure 4), which are analogs of active pharmaceutical ingredients (APIs).^34^ After carefully optimizing the residence time (Table S6), under otherwise identical conditions to the batch protocol, except that *the acridine load was decreased to 2.5 mol %*, we prepared compound **6** in the gram scale (see details in SI). Most importantly, the productivity was significantly improved in flow.

**Figure 4:**
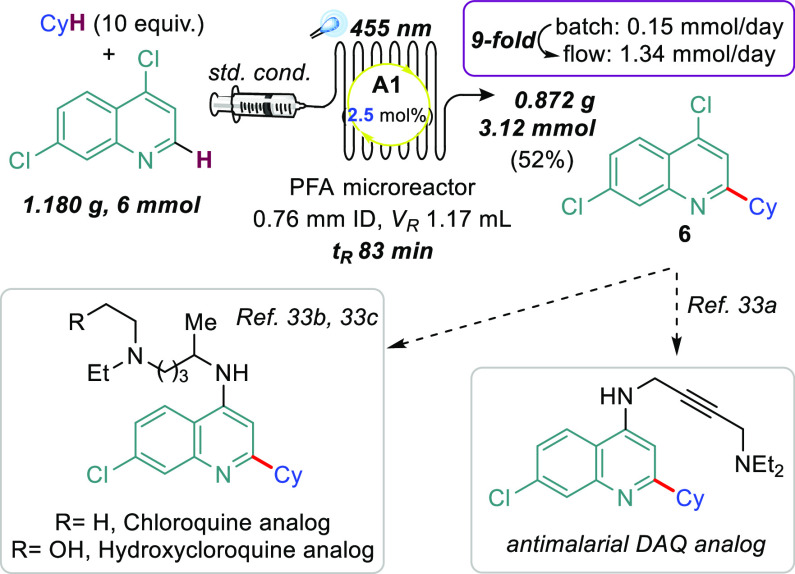
Scale up in flow and formal syntheses of APIs.

In conclusion, we have demonstrated that the CDC of azaarenes with unactivated alkanes can be promoted by visible light without sacrificial oxidants, metals, halide sources, or chlorinated solvents. A catalytic system based on a readily available 9-arylacridine photocatalyst and pyridine *N*-oxide was used for the first time in this transformation. Mechanistic studies support a dual photoredox/HAT catalytic cycle with a H_2_ evolution. The developed catalytic system may help in approaching future C–H functionalizations.

## Data Availability

The data underlying this study are available in the published article and its Supporting Information.

## Supporting Information Available

Experimental procedures and optimization, mechanistic studies, and full characterization (including NMR spectra) of all products (PDF)
