# Supplementary material for: Alkanes in Minisci-Type Reaction under Photocatalytic Conditions with Hydrogen Evolution
Source: Org Lett. 2023 Oct 11;25(48):8541–6. doi: 10.1021/acs.orglett.3c02619 (PMC10714401; doi:10.1021/acs.orglett.3c02619)
Supplement: Supplementary file 1 — ol3c02619_si_001.pdf [file ol3c02619_si_001.pdf]

Supporting information for

Alkanes in Minisci-Type Reaction under Photocatalytic Conditions with Hydrogen Evolution.

Loris Laze, Beatriz Quevedo-Flores, Irene Bosque,* Jose C. Gonzalez-Gomez*

Instituto de Síntesis Orgánica (ISO) and Departamento de Química Orgánica

Universidad de Alicante, Apdo. 99, 03080 Alicante, Spain

irene.bosque@ua.es; josecarlos.gonzalez@ua.es

Table of contents

MATERIALS AND METHODS 5

General methods 6

Preparation of HAT Catalysts 6

General Procedure for the Preparation of Pyridine *N*-oxides (**PyO1** and **PyO2**): 6

Preparation of **PyO3**: 6

Preparation of acridine catalysts 7

9-(2-Chlorophenyl)-2,7-dimethylacridine (A1): 7

9-(2-Methylphenyl)-2,7-dimethylacridine (A2): 7

9-(2-Bromophenyl)-2,7-dimethylacridine (A3): 8

9-([1,1'-Biphenyl]-2-yl)acridine (A4) 8

N-(2-Methoxy-ethoxymethyl)-9-acridone (S1) 9

9-Mesitylacridine (A5) 9

Preparation of CDC starting materials 10

Synthesis of methyl quinoline-4-carboxylate 10

Synthesis of *O*-Methylmenthol 10

Synthesis of isoamyl acetate 11

Synthesis of 2-cyclohexyl-2,3-dihydrobenzo[d]thiazole (**20-H2**) 11

Optimization of the reaction conditions in batch. 12

C(sp^2)-H alkylation of azaarenes with C(sp^3)-H substrates: General procedure (GP) for the CDC reaction.. 13

Reaction progress for the synthesis of **1**: 14

Flow experiments: 15

Flow reactor set-up: 15

Optimization of conditions for the synthesis of product **1**: 16

Optimization of conditions for the synthesis of product **6.** 17

Calibration curves: 17

Optimization results: 18

Further optimization of the reaction in flow for the synthesis of product **6**: 19

Gram-scale synthesis of **6** using flow chemistry 20

Quenching experiments 21

UV-Vis absorption spectra 23

Quantum Yield measurement 25

Determination of the photon flux: 25

Determination of the quantum yield of the reaction: 26

Other mechanistic studies 27
TEMPO addition 27
1,1-Diphenylethylene addition 27
Dehydrogenation of intermediate **20-H_2** under the reaction conditions 29
Reaction in the absence of O_2. 29
H_2 detection experiments 29
GC detection of H_2 gas: 29
Visual observation: 30
Kinetic Isotopic Experiments (KIE) 31
Individual/parallel runs for products **1** and **1-D_{11}**: 31
Competitive runs for products **1** and **1-D_{11}**: 32
Individual/parallel runs for products **20** and **20-D_{11}**: 33
Competitive runs for products **20** and **20-D_{11}**: 34
Characterization of Products 35
Alkylation of Lepidine with cyclohexane 35
Alkylation of Quinaldine with Cyclohexane 35
Alkylation of 4-Phenylquinoline with Cyclohexane 35
Alkylation of 2-Phenylquinoline with Cyclohexane 36
Alkylation of 2-Chloroquinoline with Cyclohexane 36
Alkylation of 4,7-Dichloroquinoline with Cyclohexane 37
Alkylation of 4-Methoxyquinoline with Cyclohexane 37
Alkylation of Quinoline with Cyclohexane 38
Alkylation of Quinoline with Cyclohexane 39
Alkylation of 6-Fluoroquinoline with Cyclohexane 40
Alkylation of ethyl nicotinate with Cyclohexane 41
Alkylation of 4-Phenylpyridine with Cyclohexane 41
Alkylation of Isonicotinonitrile with Cyclohexane 42
Alkylation of 1-methylquinoxalin-2(1H)-one with Cyclohexane 43
Alkylation of Quinoxalin-2(1H)-one with Cyclohexane 43
Alkylation of 1-Methyl-6-nitroquinoxalin-2(1H)-one with Cyclohexane 43
Alkylation of Quinoxaline with Cyclohexane 44
Alkylation of pyrazine-2,3-dicarbonitrile with Cyclohexane 44
Alkylation of Phenanthridine with Cyclohexane 45
Alkylation of Benzo[*d*]thiazole with Cyclohexane 45
Alkylation of 1-methyl-1H-benzo[*d*]imidazole with Cyclohexane 45
Alkylation of Lepidine with Cyclopentane 46

Alkylation of Lepidine with Cyclododecane 46
Alkylation of Lepidine with Methylcyclopentane 47
Alkylation of Lepidine with Bicyclo[2.2.1]heptane 48
Alkylation of Lepidine with Adamantane 49
Alkylation of Phenanthridine with 2,3-Dimethylbutane 49
Alkylation of Lepidine with 2-Methylbutane 50
Alkylation of Lepidine with Pentane 50
Alkylation of Lepidine with Mesitylene 51
Alkylation of Lepidine with *p*-cymene 51
Alkylation of Lepidine with Pentanenitrile 52
Alkylation of Lepidine with isoamyl acetate 52
Alkylation of Lepidine with *N*-methylacetamide 52
Alkylation of Lepidine with pyrrolidone 53
Alkylation of Lepidine with 1,4-Dioxane 53
Alkylation of Lepidine with Tetrahydrofuran 54
Alkylation of Lepidine with *tert*-butyl methyl ether 54
Alkylation of Lepidine with Methanol 55
Alkylation of Lepidine with Methanol-d4 55
Alkylation of Lepidine with Ethanol 56
Alkylation of Lepidine with Isoamyl Alcohol 56
Alkylation of Lepidine with 3-methylbutanal 57
Alkylation of Lepidine with lilial 57
Alkylation of Lepidine with Pivalaldehyde 58
Alkylation of (*S*)-Nicotine with Cyclohexane 58
Alkylation of Cinchonine with Cyclohexane 59
Alkylation of Quinine with Cyclohexane 59
Alkylation of Lepidine with *O*-Methylmenthol 60
Alkylation of Lepidine with (-)-ambroxide 60
Unsuccessful substrates: 61
NMR spectra of synthesized compounds 62
References 121

MATERIALS AND METHODS

Solvents and reagents: All solvents and commercially available reagents were purchased as reagent grades and were used without further purification unless otherwise stated.

General methods: All air- and moisture-insensitive reactions were carried out under an ambient atmosphere and monitored by thin-layer chromatography (TLC) and Gas Chromatography-mass spectroscopy (GC-MS). TLCs were performed on silica gel 60 F_{254}, using aluminum plates and visualized by exposure to ultraviolet light. Flash column chromatography (FC) was performed using Merck silica gel 60 (230–400 mesh). Yields refer to purified compounds unless otherwise stated.

Photochemistry: The photochemical reactions were carried out in an Evoluchem PhotoRedOx Box Duo™ reactor (for more details, see: https://www.hepatochem.com/photoreactors-leds-accessories/photoredox-duo/) equipped with two 18 W EvoluChem LEDs 450PF radiating at 450 nm with total irradiance of 34 mW × cm^{-2} (for light spectrum and other details, see: https://www.hepatochem.com/product/hck1012-xx-002/). Vials of borosilicate glass were placed at fixed positions of the vial holder designed for the Evoluchem photoreactor.

Analytical Information:

Melting points (MP) of the products obtained were measured using capillaries and were not corrected.

NMR spectra were recorded at 300 or 400 MHz for ^1H and 75 or 101 MHz for ^{13}C, using $CDCl_3$ or DMSO-d_6 as solvent. For ^1H-NMR in $CDCl_3$, TMS was used as an internal standard (0.00 ppm). For ^1H-NMR in DMSO-d_6, the residual signal was used as the internal standard (2.50 ppm). Data are reported as (s = singlet, d = doublet, t = triplet, q = quartet, m = multiplet or unresolved, brs = broad signal, coupling constant(s) in Hz, integration). ^{13}C-NMR spectra were recorded with ^1H-decoupling at 101 MHz and referenced to $CDCl_3$ at 77.16 ppm or DMSO-d_6 at 39.52 ppm.

LRMS were obtained using an Agilent 5977B mass spectrometer with a quadrupole analyzer coupled with a gas chromatographer Agilent 8890. The oven temperature was: 3 min at 80 ºC, then 20 ºC/min ramp until 300 ºC, then 3 min at 300 ºC.

HRMS analyses were carried out in the electron impact mode (EI) at 70 eV using a quadrupole mass analyzer or by Q-TOF using electrospray ionization (ESI) mode.

UV-Vis experiments were performed on a VWR UV-3100PC spectrophotometer using a quartz cuvette of 10 mm path length and 3 mL of volume.

Quenching experiments were performed in a Horiba spectrofluorometer using a quartz cuvette of 3 mL. Monochromators were set with 1.5 nm of entrance and exit slit for excitation and emission monochromators, and an integration time of 0.1 s was selected.

The H_2 detection was performed in a gas chromatographer Agilent 8890GC at 75 ºC using a molecular sieve column of 5 A, a 20 mL/min flow with He, and a TCD detector set at 200 ºC.

General methods

Preparation of HAT Catalysts

General Procedure for the Preparation of Pyridine *N*-oxides (**PyO1** and **PyO2**):

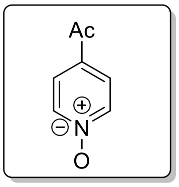

PyO1: 0.406 g (98%)

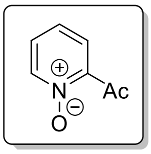

PyO2: 0.140 g (34%)

Following a procedure previously reported, pyridine *N*-oxides were prepared.[1] A solution of the pyridine (1.0 equiv) in dry CH_2Cl_2 (0.17 M) was prepared, and *m*-CPBA (1.2 equiv, 77% w/w) was added portion-wise to the cooled solution at 0 °C. The mixture was stirred at room temperature overnight, concentrated to ¼ of its volume and purified by flash-column chromatography (silica, EtOAc to 10% MeOH/EtOAc).

Preparation of **PyO3**:

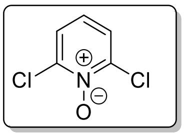

PyO3: 0.567 g (70%)

2,6-Dichloropyridine *N*-oxide was prepared following a modified procedure from the literature.[2] The corresponding pyridine (1.0 equiv) and UHP (2.0 equiv) were dissolved in anhydrous dichloromethane (0.3 M). TFAA (2.0 equiv) was added dropwise to the cooled reaction mixture at 0 °C and left stirring at the same temperature for 30 min and then 3 h at room temperature. After this, the reaction mixture was quenched with a statured sodium persulfate solution and extracted three times with dichloromethane. The combined organic layers were dried with $MgSO_4$. After filtration, the organic solvent was concentrated to ¼ of its volume and purified by flash-column chromatography (silica, Hexane to 30% EtOAc).

Preparation of acridine catalysts

9-(2-Chlorophenyl)-2,7-dimethylacridine (A1):

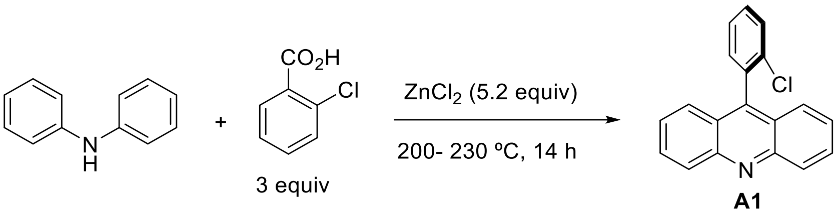

Following a reported protocol,[3] to a 20 mL pressure tube, diphenylamine (1 g, 5 mmol), 2-chlorobenzoic acid (2.40 g, 15 mmol, 3 equiv.), and zinc chloride (3.6 g, 26 mmol, 5.2 equiv.) were added. The reaction was allowed to stir at 220-230 °C in a sand bath for 14 h. After completion, the reaction was quenched with a saturated ammonium hydroxide solution (50 mL, 30% w/w) and extracted with EtOAc (3 x 60 mL). The collected organic layers were washed with brine and dried over $MgSO_4$. Removal of the solvent and purification by FC eluting with a gradient from 10% to 20% EtOAc in *n*-hexane afforded acridine **A1** as a yellow solid (1.020 g¸70%).

TLC: Rf = 0.29 (9:1 hexane/EtOAc, UV)

GC (T_i= 80 °C): R_t 10.111 min.

MS: *m/z* (%) 291 (M^+, ^{37}Cl, 26), 289 (M^+, ^{35}Cl, 77) 254 (100), 126 (32), 291 (31), 253 (29).

NMR data matched that reported in the literature.[4]

9-(2-Methylphenyl)-2,7-dimethylacridine (A2):

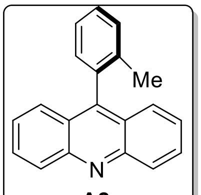

Following a reported protocol,[4] to a 20 mL pressure tube, diphenylamine (1 g, 5 mmol), 2-methylbenzoic acid (1.30 mL, 10 mmol, 2 equiv.), and zinc chloride (1.38 g, 10 mmol, 2 equiv.) were added. The reaction was allowed to stir at 220 °C in a sand bath for 6 h. After this, the reaction was quenched with saturated ammonium hydroxide (15 mL, 30% w/w) and left stirring overnight at room temperature. At this point, the reaction was extracted with EtOAc (3 x 20 mL). The collected organic layers were washed with brine and dried over $MgSO_4$. Removal of the solvent and purification by FC eluting with a gradient from 10% to 30% EtOAc in *n*-hexane afforded acridine **A2** as a yellow solid (1.21 g¸90%).

TLC: Rf = 0.36 (9:1 hexane/EtOAc, UV)

GC (Ti= 80 °C): Rt 9.625 min.

MS: *m/z* (%) 269 (M^+, 100), 268 (73), 267 (33), 270 (20), 254 (19).

NMR data matched that reported in the literature. [4]

9-(2-Bromophenyl)-2,7-dimethylacridine (A3):

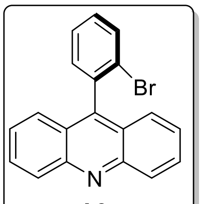
A3

It was prepared from diphenylamine (5 mmol), 2-bromobenzoic acid (10 mmol), and zinc chloride (10 mmol), using the same procedure used for **A2**. The purification by FC eluting with a gradient from 5% to 10% EtOAc in *n*-hexane afforded **A3** as a yellow solid (414 mg¸ 25%).

TLC: Rf = 0.28 (9:1 hexane/EtOAc, UV)

GC (Ti= 80 ºC): Rt 10.490 min.

MS: *m/z* (%) 333 (M^+, 37), 335 (M^+, 37), 254 (100), 335 (43), 127 (24), 253 (23), 255 (22).

NMR data matched that reported in the literature. [4]

9-([1,1'-Biphenyl]-2-yl)acridine (A4)

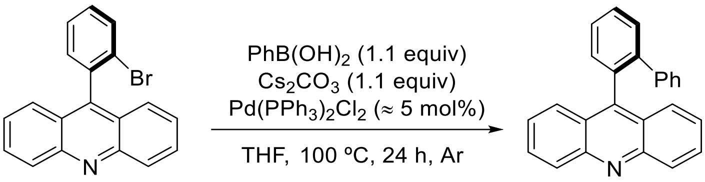

A3 A4

A solution of acridine **A3** (0.30 mmol, 100 mg), phenylboronic acid (0.33 mmol, 41 mg, 1.1 equiv.), $Pd(PPh_3)_2Cl_2$ (0.015 mmol, 11 mg, 0.05 equiv.) and Cs_2CO_3 (0.33 mmol, 108 mg, 1.1 equiv.) in THF (4 mL) were stirred in a sealed tube at 100 °C (sand bath) for 24 h under an argon atmosphere. After cooling at room temperature, water was added (5 mL), and the reaction mixture was extracted with EtOAc (3x10 mL). The collected organic layers were washed with saturated NH_4Cl aqueous solution, followed by brine, and dried over $MgSO_4$. The solvent was evaporated under reduced pressure, and the residue was purified by FC eluting with 10% of EtOAc in *n*-hexane to furnish acridine **A4** as a yellow solid (60 mg¸60%).

TLC: Rf = 0.33 (9:1 hexane/EtOAc, UV)

GC (Ti= 80 ºC): Rt 11.475 min.

MS: *m/z* (%) 331 (M^+, 100), 330 (77), 332 (25), 207 (24), 328 (21).

NMR data matched that reported in the literature. [4]

Preparation of $Pd(PPh_3)_2Cl_2$ catalyst

This catalyst was prepared according to the following procedure:[5] $PdCl_2$ (53 mg, 0.3 mmol) was added to an oven-dried *Schlenck* flask, followed by dry THF (3 mL) and LiCl (26 mg, 0.6 mmol) under Ar atmosphere. The reaction mixture was stirred under an Ar atmosphere for 5 min at 25 ºC. After this time, PPh_3 (157 mg, 0.60 mmol) was added to the resulting grey suspension and the rxm was stirred for at least 2 h at 25 ºC. The formed pale-yellow suspension remained under the Ar atmosphere and was directly used for the Suzuki cross-coupling reaction.

N-(2-Methoxy-ethoxymethyl)-9-acridone (S1)

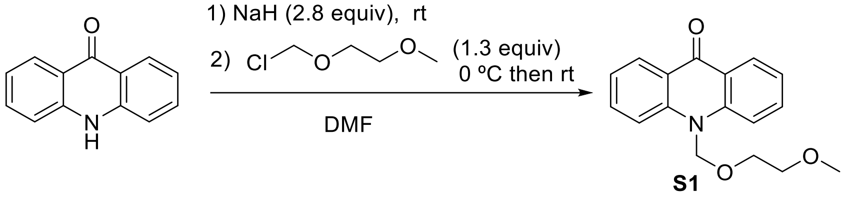

Following a reported protocol,[6] sodium hydride (550 mg of a 60% suspension in oil; 14 mmol) was added under an argon atmosphere to a solution of 9(10H)-acridone (1 g; 5 mmol) in DMF (100 ml). The reaction mixture (rxm) was stirred for 30 min at room temperature (RT) and then was cooled to 0 °C. Next, 2-Methoxyethoxymethyl chloride (0.75 ml; 6.5 mmol) was added carefully, and the rxm was stirred for 30 min at 0 °C and 2 h at RT. The reaction mixture was distributed between EtOAc (100 mL) and H_2O (400 mL). The phases were separated, and the aqueous phase was further extracted with EtOAc (3x50 mL). The collected organic phases were washed with a saturated NaCl solution and dried over $MgSO_4$. Subsequently, the solvent was removed under reduced pressure, and the residue was submitted to FC eluting with 30% EtOAc in *n*-hexane to obtain pure **S1** as a light-yellow solid (1.15 g¸80%).

TLC: Rf = 0.41 (9:1 hexane/EtOAc, UV)

GC (T_i= 80 °C): Rt 10.700 min.

MS: *m/z* (%) 283 (M^+, 80), 209 (26), 207 (22), 195 (30), 180 (26), 152 (30).

9-Mesitylacridine (A5)

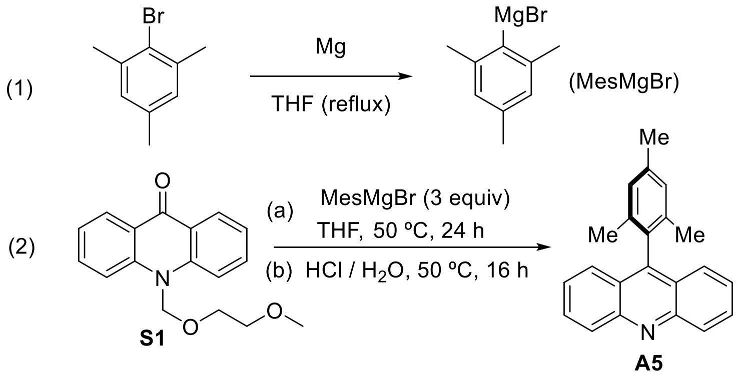

Step 1: Magnesium turnings (0.365 g, 1.5 equiv.) were triturated and added to a two-necked round-bottomed flask (50 mL) connected to a reflux condenser and an addition funnel under an Ar atmosphere. A solution of 2-Bromomesitylene (1.5 mL, 10 mmol, 1 equiv.) in dry THF (10 mL) was charged in the funnel, and a portion (2-3 mL) was added to the Mg. The suspension was sonicated over 5 min. The rest of the solution was added dropwise with stirring at RT and then refluxed for 3 h (abundant bubbling was observed after some minutes at 60 °C).

Step 2: To a solution of *N*-(2-methoxy-ethoxymethyl)-9-acridone (**S1**) (226 mg, 0.80 mmol) in dry THF (25 mL) at 0 °C under argon was added a freshly prepared solution of mesityl magnesium bromide in THF (5 mL, above described) over 15 min *via* syringe. Then the rxm was stirred at 50 °C for 24 h, observing a color change

from yellow to brown. After cooling to RT, the rxm was transferred to a 250 mL flask, and a 12 M aqueous HCl solution (30 mL) and water (25 mL) were added. The resultant mixture was stirred for 16 h at 50 °C. The mixture was treated with a saturated aqueous solution of $NaHCO_3$ and extracted with EtOAc. The collected organic layers were washed with brine and dried over $MgSO_4$. The crude product was purified by FC eluting with a gradient from 0% to 5% EtOAc in *n*-hexane to afford acridine **A5** as a yellow solid (175 mg, 74%).

TLC: Rf = 0.38 (9:1 hexane/EtOAc, UV)

GC (Ti= 80 ºC): Rt 9.839 min.

MS: *m/z* (%) 297 (M^+, 100), 296 (44), 207 (28), 281 (28), 282 (25), 298 (24).

NMR data matched that reported in the literature.[7]

Preparation of CDC starting materials

Substrates used in the CDC reaction were commercially available except for the following ones:

Synthesis of methyl quinoline-4-carboxylate

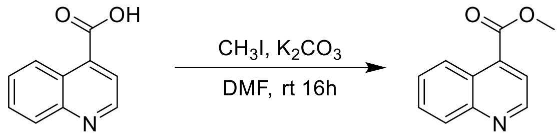

Following a reported protocol,[8] a solution of quinoline-4-carboxylic acid (104 mg, 0.6 mmol), methyl iodide (112 µL, 1.8 mmol) and potassium carbonate (300 mg, 2.4 mmol) in DMF (4 mL) was stirred at room temperature for 16h. The reaction mixture was diluted with brine and extracted with EtOAc. The combined organic layers were dried, filtered and concentrated in vacuo. After purification by FC, the product was obtained as a white oil (87 mg, 0.46 mmol, 77%) using from 5% to 10% EtOAc in *n*-hexane as the eluent.

Synthesis of *O*-Methylmenthol

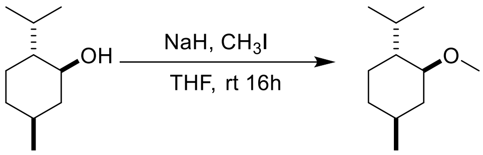

To a suspension of NaH (60% in mineral oil, 132 mg, 3.3 mmol, 1.1 equiv) in anhydrous THF (12 mL), (±)-menthol (469 mg, 3 mmol, 1 equiv) was added at 0 °C under an argon atmosphere and the reaction mixture was stirred for 30 min. After adding methyl iodide (595 mg, 4.2 mmol, 1.4 equiv), the reaction mixture was warmed to room temperature and stirred for 12 h. The reaction was quenched with water (5 mL). THF was removed under vacuum, and the residue was extracted with ethyl acetate (5 mL × 2). The organic phase was combined, washed with brine (10 mL) and dried with anhydrous Na_2SO_4. The solvent was removed by evaporation under reduced pressure to give the pure product as a colorless liquid (80 % yield, 408 mg, 2.4 mmol).[9]

Synthesis of isoamyl acetate

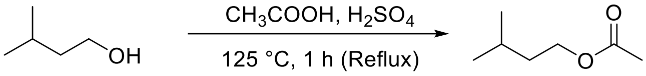

To a solution of acetic acid glacial (4 mL, 70 mmol) and isoamyl alcohol (3 mL, 27 mmol) were added concentrated H_2SO_4 (0.8 mL), and the solution mixture was stirred at 125°C for 1 h. After cooling to room temperature, the solution mixture was washed with a saturated solution of $NaHCO_3$ (3 x 15 mL), then dried over $MgSO_4$ and filtrated. The filtered solution mixture was distilled, collecting the fraction from 132-134 °C. After distillation, the product was obtained as a colorless liquid (3.16 g, 90 % yield, 24 mmol).

Synthesis of 2-cyclohexyl-2,3-dihydrobenzo[d]thiazole (**20-H2**)

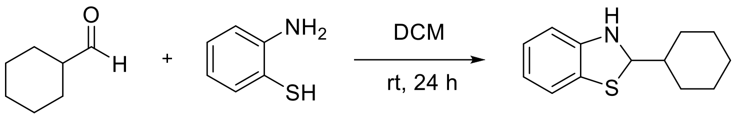

20-H2

To a stirred solution of cyclohexanecarbaldehyde (1.1 mL, 8.9 mmol) in dichloromethane (10 ml) was added 4Å molecular sieves (7.0 g). 2-Aminothiophenol (855 mg, 8.0 mmol) was added dropwise to the mixture and stirred at room temperature for 24 h.[10] After completion of the reaction, the reaction mixture was filtered and washed with EtOAc to remove molecular sieves and residue. The solvent filtrated was concentrated in vacuo. After purification by FC, the product was obtained as a green solid (1 g, 4.5 mmol, 50%) using from 1% to 5% EtOAc in *n*-hexane as the eluent.

Optimization of the reaction conditions in batch.

Table S1: Screening different conditions with A1 and PyO1.

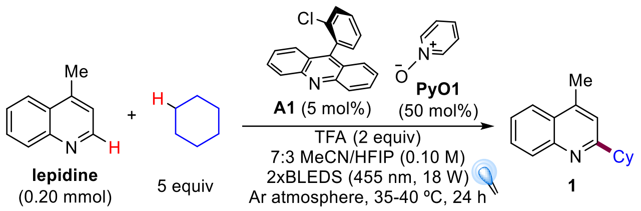

Entry	Deviation from above	Yield of **1** (%)[a]
1[b]	5 min Ar bubbling/Ar atmosphere[c]	81/65
2[b]	no Ar/no **A1**/no light	**91**/25/0
3[d,b]	TFA (mol%): 0/20/50/100/300	0/2/20/44/65
4[d]	**PyO1** (25 mol%), 22 h *vs.* 32 h	81 *vs.* **94**
5[d]	**PyO1** (25 mol%), MeCN *vs.* HFIP	69 *vs.* 0
6[d]	**PyO1** (25 mol%), 10 equiv CyH	82
7[d]	**PyO1** (25 mol%), [lepidine]= 0.20 M	76
8[d]	**PyO1** (30 mol%), 32 h	**100 (76)**[e]

[a]GC yield based on remaining SM without calibration. [b]**A1** (10 mol%). [c]Three cycles of freeze-pump-thaw-Ar backfilled.[d]Closed under air. [e]Isolated pure product.

Table S2: Screening different HAT catalysts and acridine photocatalysts.

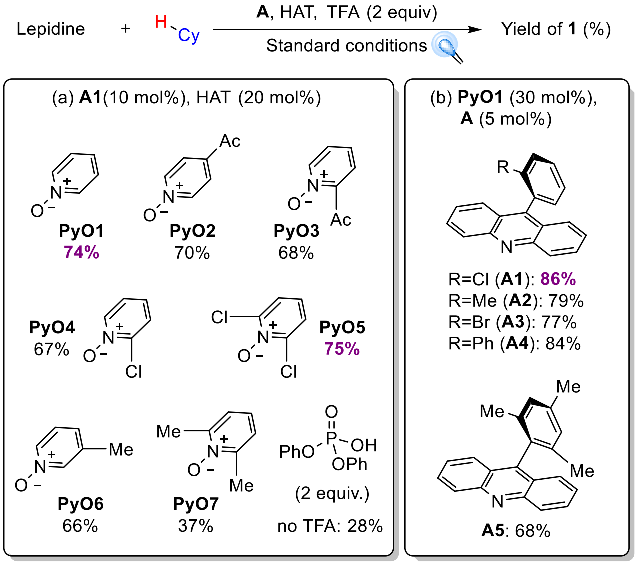

C(sp2)-H alkylation of azaarenes with C(sp3)-H substrates: General procedure (GP) for the CDC reaction

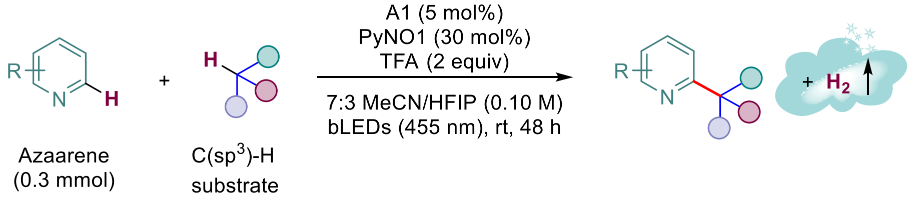

In a two-dram vial, equipped with a stirring bar, was added the azaarene (0.30 mmol), pyridine *N*-oxide (8.4 mg, 0.09 mmol, 30 mol%), and 9-(2-Chlorophenyl)-2,7-dimethylacridine (**A1**, 4.4 mg, 0.015 mmol, 5 mol%), followed by a mixture of acetonitrile/1,1,1,3,3,3-Hexafluoro-2-propanol (7:3, 3 mL). Then TFA (45 µL, 0.60 mmol, 2 equiv) and the C(sp^3)-H substrate (3, 5, 10, 23 equiv, amount specified in the individual experiment) were added to the reaction mixture. All reagents were added in open-air conditions. The vial was closed, inserted into the PhotoRedOx Box Duo reactor, and irradiated with blue LEDs (450 nm, two LEDs were used for four reaction vials) for about 48 h, maintaining the stirring at room temperature (35-40 ºC controlled by a fan in the photoreactor). Once this time elapsed, K_2CO_3 was added (207 mg,1.5 mmol, 5 equiv), and the reaction mixture was stirred for 30 min. After this time, the suspension was filtered through Celite, eluting with EtOAc (4 x 4 mL). After evaporating the solvent under vacuum, the residue was purified by FC.

Reaction progress for the synthesis of **1**:

In a two-dram vial was added the lepidine (43 µL, 0.30 mmol), pyridine *N*-oxide (8 mg, 30 mol %), and 9-(2-chlorophenyl)-2,7-dimethylacridine (**A1**, 4.4 mg, 0.015 mmol, 5 mol%), followed by a mixture of acetonitrile/1,1,1,3,3,3-Hexafluoro-2-propanol (7:3, 3 mL). Then TFA (45 µL, 0.60 mmol, 2 equiv) and cyclohexane (162 µL, 1.50 mmol, 5 equiv) were added to the reaction mixture. The vial was closed, inserted into the PhotoRedOx Box Duo reactor, and irradiated with blue LEDs (450 nm, two LEDs were used for four reaction vials). At the desired times (T1 to T9), an aliquot of 170 µL was taken from the reaction vial and diluted up to 1.5 mL with MeCN (Figure S1). These samples were injected in the GC, obtaining the corresponding GC yields (Table S1).

Table S3: GC yields over time of a reaction towards the synthesis of **1**.

Exp.	Time	Time elapsed (h)	GC yield (%)[a]
T1	9:00 a.m.	0	0
T2	10:00 a.m.	1	35
T3	11:00 a.m.	2	48
T4	1:00 p.m.	4	61
T5	3:00 p.m.	6	65

Exp.	Time	Time elapsed (h)	GC yield (%)[a]
T6	7:00 p.m.	10	68
T7	1:00 p.m. (+1)	28	79
T8	5:00 p.m. (+1)	32	84
T9	9:00 a.m. (+2)	48	88

[a] Uncorrected GC yields.

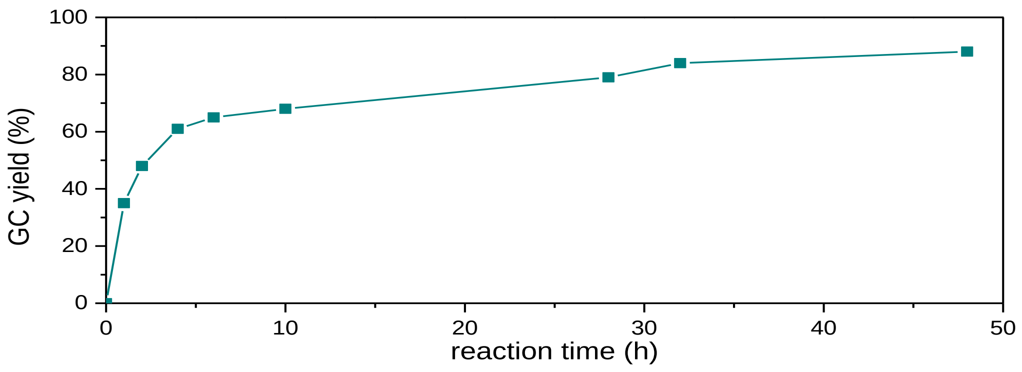

Figure S1: Reaction progress in the synthesis of **1.**

Flow experiments:

Flow reactor set-up:

Flow reactor characteristics: Residence volume: 1. 17 mL; Length: 2.70 m; Internal diameter: 0.7 mm)

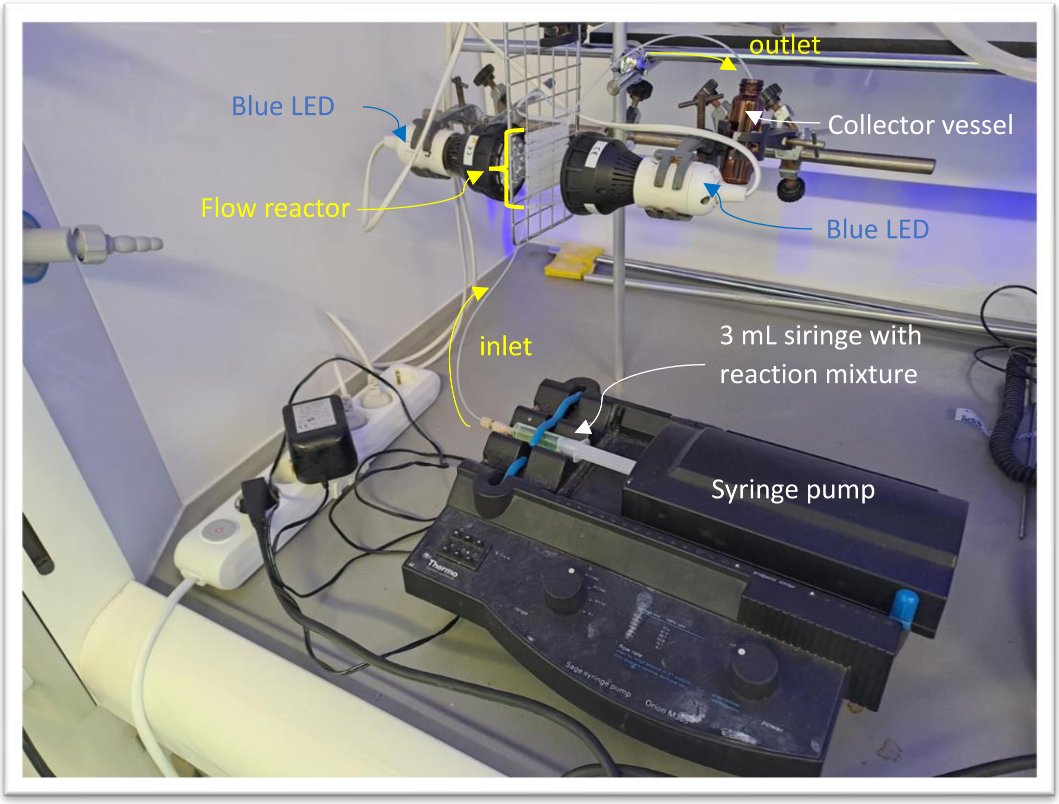

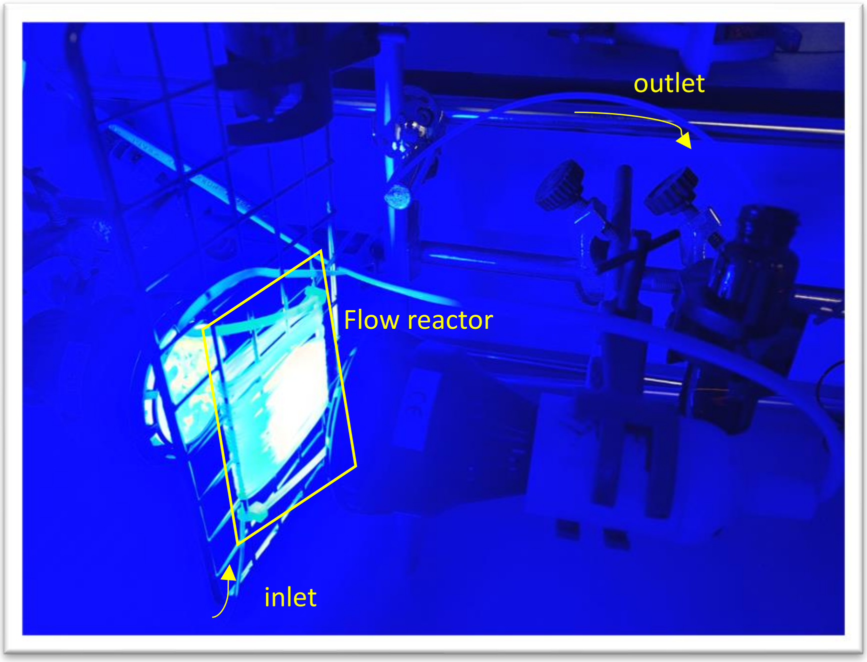

Figure S2: Flow reactor pictures

Optimization of conditions for the synthesis of product 1:

In a two-dram vial was added the lepidine (14 µL, 0.10 mmol), pyridine *N*-oxide (20 mol%, 30 mol% or 20+10 mol%), and **A1** (1.5 mg, 0.005 mmol, 5 mol%), followed by a mixture of acetonitrile/1,1,1,3,3,3-hexafluoro-2-propanol (7:3, 1 mL). Then TFA (15 µL, 0.20 mmol, 2 equiv) and cyclohexane (108 µL, 1 mmol, 10 equiv) were added to the reaction mixture. Once all reactants were mixed, a 3 mL syringe was charged with the reaction mixture, connected to the flow reactor, and irradiated with two blue LEDs (455 nm). The reaction mixture was pumped using a syringe pump to furnish the desired residence time (t_R). The crude reaction mixture was analyzed by GC, and the product was isolated when noted.

Table S4: Flow experiments results to furnish product **1**.

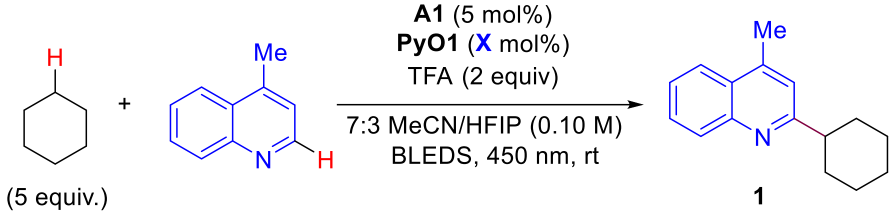

**PyO1** (**X** mol%)	t_R (min)	GC yield of **1** (%)[a]
30 mol%	7.5	30%
30 mol%	96	78%
30 mol%	213	82%
**30 mol%**	**438**	**96%/95% (82%)**
20 mol%	106	65%
20 mol%	212	73%
20 + 10 mol% [b]	212	76% (72%)

[a] Uncorrected GC yields. Isolated yields in parenthesis. [b] 20 mol% of **PyO1** was added at the beginning of the reaction, flowing at 106 min of residence time, and then, 10 mol% more of **PyO1** was added and flowed an extra 106 min through the flow reactor, allowing a total of 212 min of residence time.

Optimization of conditions for the synthesis of product **6.**

Calibration curves:

Calibration curves for 4,7-dichloroquinoline (SM) and 2-cyclohexyl-4,7-dichloroquinoline (**6**) were performed in a GC using decane as internal standard (IS). Three stock solutions of 0.1 M in MeCN were prepared for 4,7-dichloroquinoline, **6** and IS. GC samples were prepared by taking 100, 200, 300 or 400 µL of the IS stock solution and mixing them with 100 µL of the 4,7-dichloroquinoline stock solution (for the starting material curve) or with 100 µL of **6** stock solution (for the product curve). A total volume of 1 mL was achieved using MeCN. These prepared samples were directly injected into the GC (in the method used, retention times are: IS (5 min); SM (12 min); **6** (17 min)).

Table S5: Calibration data obtained from direct integration of the GC peaks.

Area IS	Area 6	Area 6 /Area IS	Yield of 6 (%)
0	0	0	0%
2721	451	0.16574	25%
3237	792	0.24467	33%
2580	1079	0.41821	50%
938	721	0.76865	100%
**Area IS**	**Area SM**	**Area SM/Area IS**	**Yield of SM (%)**
0	0	0	0%
827	148	0.17896	25%
579	152	0.26252	33%
1486	646	0.43472	50%
748	664	0.88770	100%

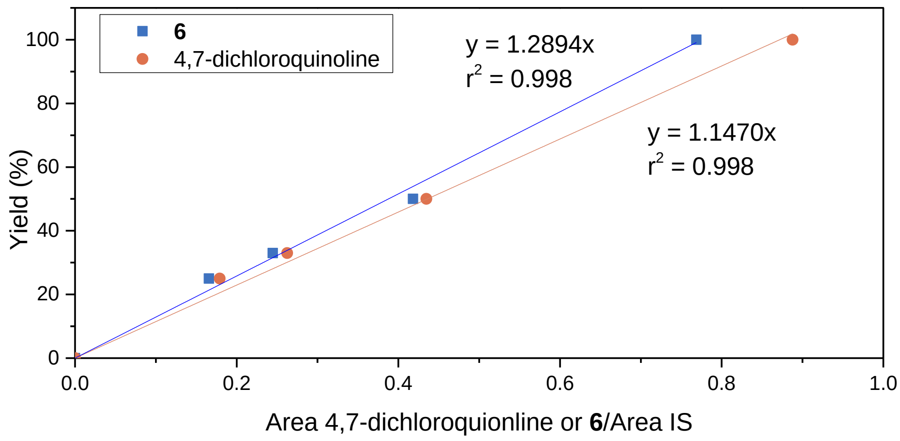

Figure S3: Calibration curves for starting material 4,7-dichloroquinoline and product **6**.

Optimization results:

In a two-dram vial was added the 4,7dichloroquinoline (20 mg, 0.10 mmol), pyridine *N*-oxide (2.7 mg, 0.03 mmol, 30 mol%), and **A1** (1.5 mg, 0.005 mmol, 5 mol%), followed by a mixture of acetonitrile/1,1,1,3,3,3-hexafluoro-2-propanol (7:3, 1 mL). Then TFA (15 µL, 0.20 mmol, 2 equiv) and cyclohexane (108 µL, 1 mmol, 10 equiv) were added to the reaction mixture. Then, the reaction mixture was transferred into a 3 mL syringe, connected to the flow reactor, and irradiated with two blue LEDs. The reaction mixture was pumped using a syringe pump to furnish the desired residence time (t_R). To the crude reaction mixture, 1 equivalent of decane (IS) was added, and the result was analyzed by GC. The product was isolated when noted.

Table S6: Optimization of the residence time in the flow experiments in the synthesis of **6**.

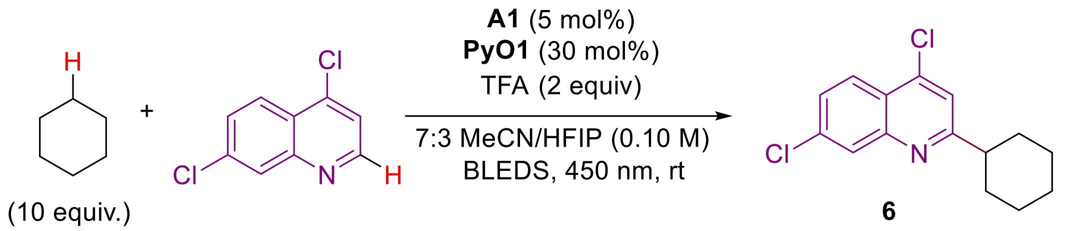

t_R (min)	GC yield of 4,7-dichloroquinoline (%)[a]	GC yield of **6** (%)[a]
3	85%	36%
8	76%	44%
24	35%	59%
**83**	**19%**	**67% (58%)**
124	12%	67%
248	4%	68%
286	0%	42%
149	0%	52%
466	0%	10%

[a] Yields calculated from the GC using decane as internal standard according to the calibration curves. Isolated yields in parenthesis.

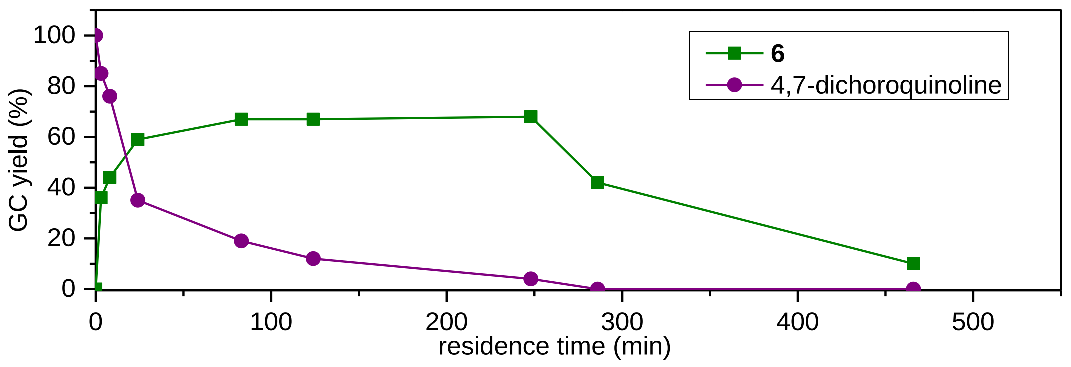

Figure S4: GC yield vs. residence time of product **6** and starting material 4,7-dichloroquinoline using decane as internal standard.

Further optimization of the reaction in flow for the synthesis of product 6:

In a two-dram vial was added the 4,7dichloroquinoline (20 mg, 0.10 mmol), pyridine *N*-oxide (as specified in the table), and **A1** (2.5 or 5 mol%), followed by a mixture of acetonitrile/1,1,1,3,3,3-hexafluoro-2-propanol (7:3, 1 mL). Then TFA (15 µL, 0.20 mmol, 2 equiv) and cyclohexane (0.5 or 1 mmol) were added to the reaction mixture. Then, the reaction mixture was transferred into a 3 mL syringe, connected to the flow reactor, and irradiated with two blue LEDs. The reaction mixture was pumped using a syringe pump to furnish the desired residence time (t_R). To the crude reaction mixture, 1 equivalent of decane (IS) was added, and the result was analyzed by GC. The product was isolated when noted.

Table S7: Further optimization of the amounts of cyclohexane, **A1** and concentration in the flow experiments in the synthesis of **6**.

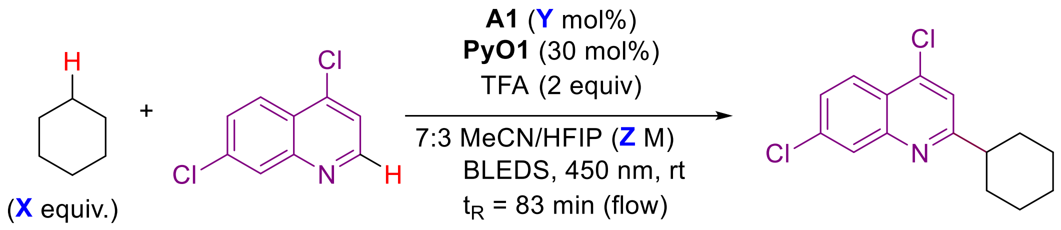

**X** (equiv)	**Y** (mol%)	**Z** (M)	GC yield of SM[a]	GC yield of **6**[a]
10	5	0.10	19%	67% (58%)
5	5	0.10	2%	56%
10	2.5	0.10	7%	66%
**10**	**2.5**	**0.12**	**9%**	**66% (58%)**
5	2.5	0.12	13%	54%

[a] Yields calculated from the GC using decane as internal standard according to the calibration curves. Isolated yields in parenthesis.

Gram-scale synthesis of **6** using flow chemistry

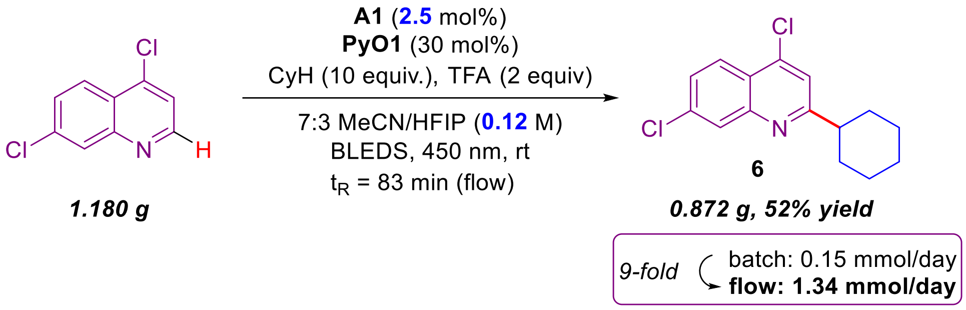

Using the optimized conditions of Table S5 (*vide supra*), the synthesis of **6** was achieved in a gram scale using the following procedure: In a 100 mL flask was added the 4,7-dichloroquinoline (1.18 g, 6 mmol), pyridine *N*-oxide (171 mg, 1.8 mmol, 30 mol%), and **A1** (43 mg, 0.15 mmol, 2.5 mol%) followed by a mixture of acetonitrile/1,1,1,3,3,3-Hexafluoro-2-propanol (7:3, 50 mL). Then TFA (918 µL, 12 mmol, 2 equiv) and cyclohexane (6.47 mL, 60 mmol, 10 equiv) were added to the reaction mixture. Once all reactants were mixed, the reaction mixture was charged into a 60 mL syringe, connected to the flow reactor, and irradiated with two blue LEDs (455 nm). The reaction mixture was pumped using a syringe pump at 0.014 mL/min (residence time of 83 min). A total time of 59 h (2.5 days) was needed. Once this time elapsed, K_2CO_3 was added (4.14 g, 30 mmol, 5 equiv), and the reaction mixture was stirred for 30 min. After this time, the suspension was filtered through Celite, eluting with EtOAc (4 x 10 mL). After evaporating the solvent under vacuum, the residue was purified by FC (0.5% EtOAc in Hexane), obtaining 0.872 g of product **6** (52% yield).

Quenching experiments

Stock solutions of a mixture of 9-(2-chlorophenyl)-2,7-dimethylacridine (**A1**) with TFA and quenchers (lepidine, **1**, and cyclohexane) were prepared. The stock solution of **A1** was prepared by mixing **A1** (10 µmol, 2.87 mg) and TFA (2.6 mmol, 200 µL) in 50 mL of MeCN to obtain a final stock solution with concentration [**A1+TFA**] = 0.2 mM. The stock solutions of each quencher were prepared by diluting **PyO1** (38 mg, 0.4 mmol) or lepidine (53 µL, 0.4 mmol) or cyclohexane (43 µL, 0.4 mmol) in MeCN (2 mL), obtaining stock solutions with concentrations [**PyO1**] = 0.2 M, [**Lep**] = 0.2 M or [**Cy-H**] = 0.2 M respectively.

For the emission spectra, 1 mL of the stock solution of **A1+TFA** was mixed with the corresponding amount of stock solution of quencher (15 µL, 30 µL and 45 µL) and diluted to a final volume of 3 mL with MeCN ($[Q]_{final}$ = 0, 1, 2, 3 mM). Samples were irradiated at 400 nm, and the emission spectra were recorded from 420 nm to 750 nm, observing a maximum emission at 490 nm (Figure S5). When using the mixtures as quenchers, the final concentration of each component remained the same ($[Q]_{final}$ = 0, 1, 2, 3 mM).

The same procedure was repeated, sparging the MeCN used with N_2, obtaining similar results.

A solution of 4 mM lepidine in MeCN in the absence or presence of 3 µL of TFA was also excited at 400 nm to rule out any interference of a possible emission at 490 nm, which was not observed.

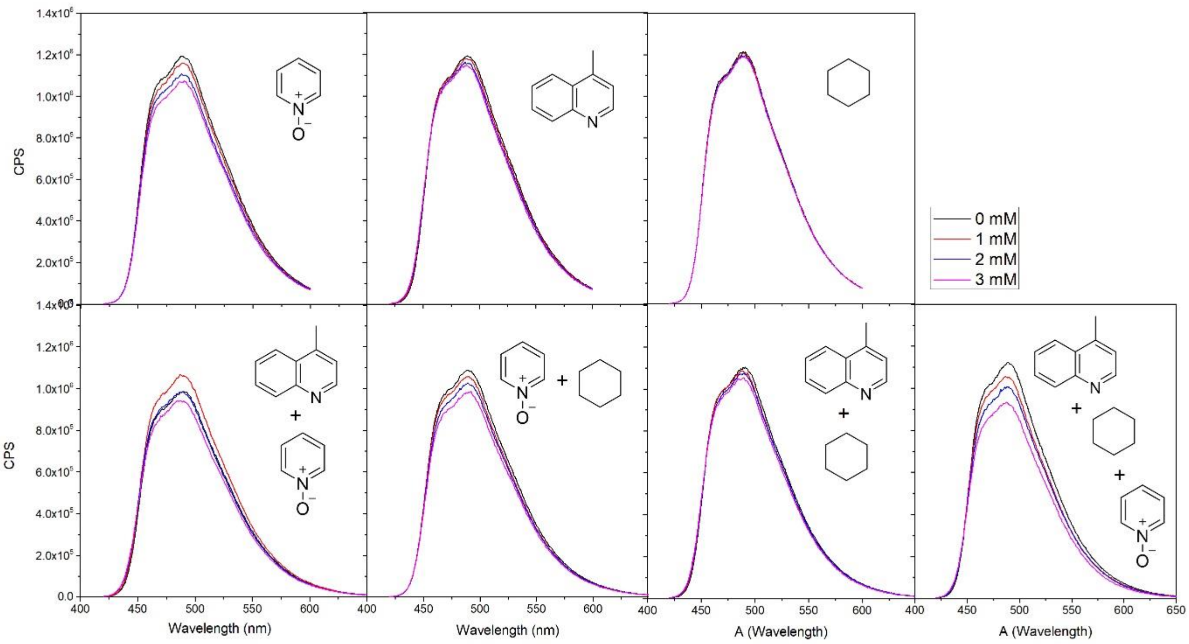

Figure S5: Emission spectra for **A1**+TFA upon adding **PyO1**, lepidine, cyclohexene or the different combinations at concentrations ranging from 0 to 3 mM.

The Stern-Volmer representation at an emission wavelength of 490 nm was performed (Figure S6). As observed, **PyO1** was found to be the best single quencher. The mixture of Lep+CyH+PyO1 was found to have a more prominent quenching response due to the fast recovery of the actual quencher (**PyO1**) through HAT from CyH and deprotonation by Lep (lepidine), which better supports the proposed mechanism.

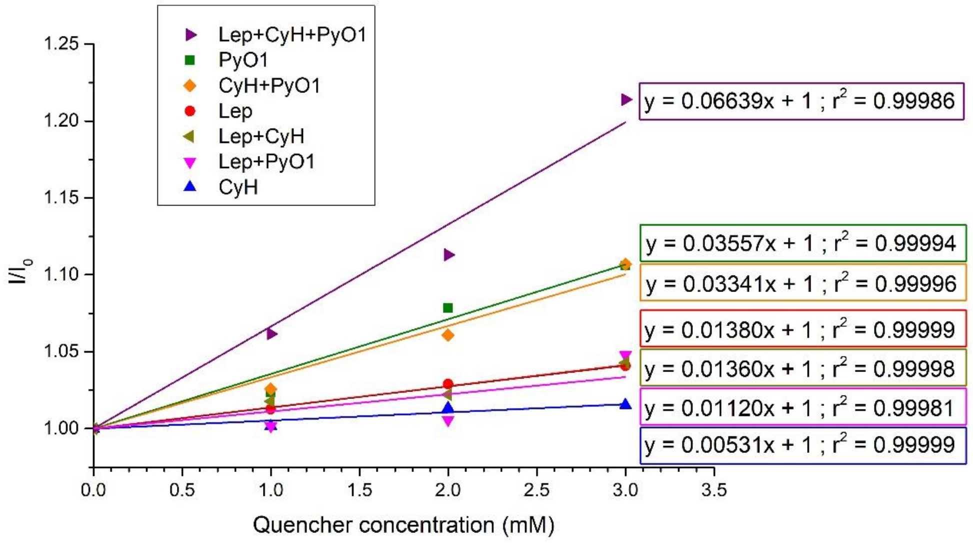

Figure S6: Stern-Volmer plot for photocatalyst **A1**+TFA with potential quenchers **PyO1**, lepidine, cyclohexane and all possible combinations.

UV-Vis absorption spectra

UV-visible absorption spectra were recorded from 200 to 700 nm for **A1** ([**A1**] = 0.2 mM), cyclohexane ([CyH] = 66 mM), lepidine ([Lep] = 66 mM) and **PyO1** ([**PyO1**] = 66 mM) (Figures S7, S8 and S9). To 3 mL of the previous solutions, 40 µL (0.520 mmol) of TFA was added, and the UV-vis spectra were recorded.

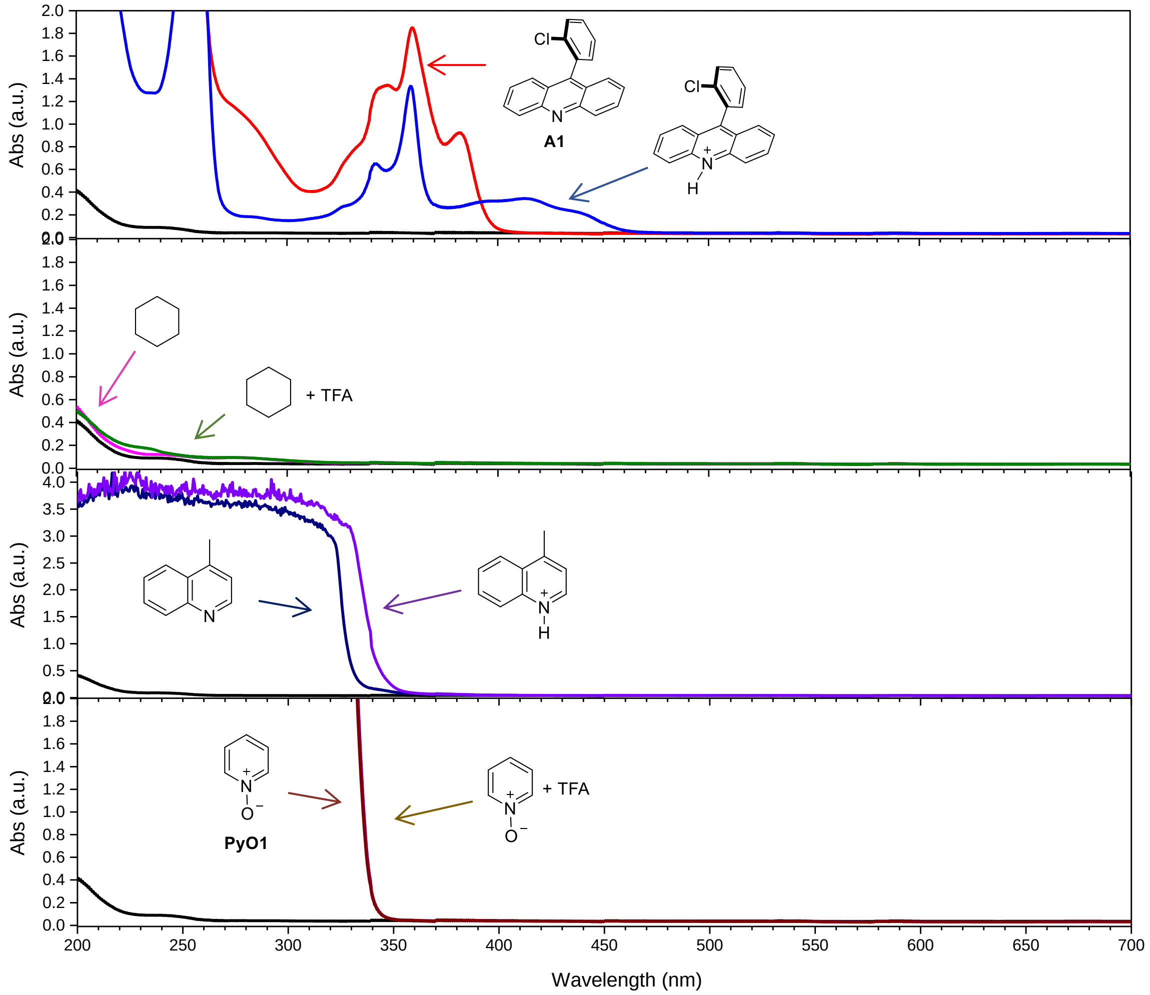

Figure S7: Individual absorption UV-vis spectra from 200 to 700 nm for **A1**, cyclohexane, lepidine and **PyO1**, and for the previous upon addition of 4 µL of TFA.

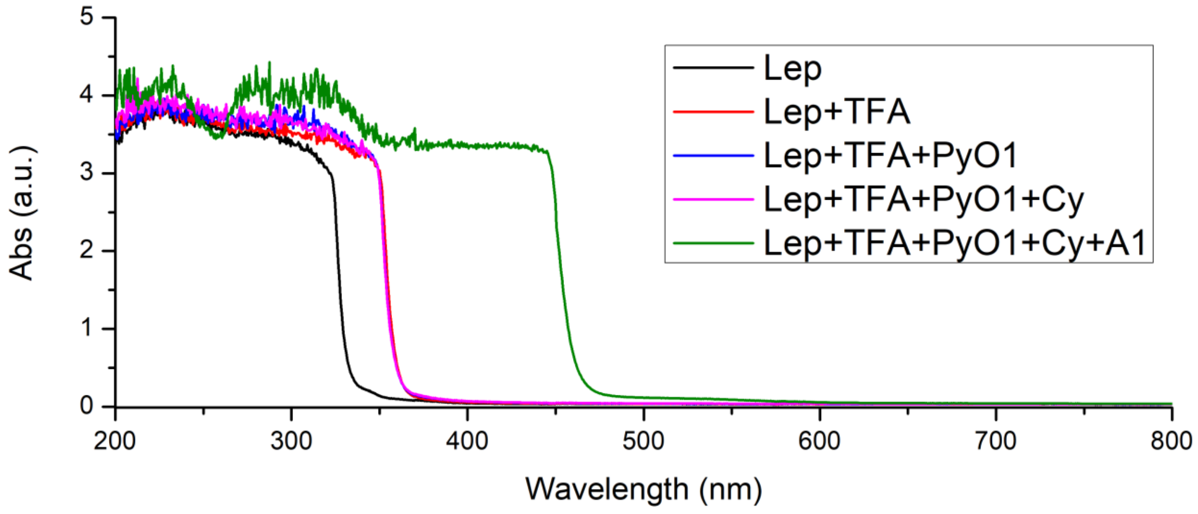

Figure S8: Absorption UV-vis spectra from 200 to 800 nm for Lepidine (0.3 mmol) in MeCN (3 mL), and sequential additions of TFA (2 equiv), **PyO1** (30 mol%), cyclohexane (5 equiv) and **A1** (5 mol%).

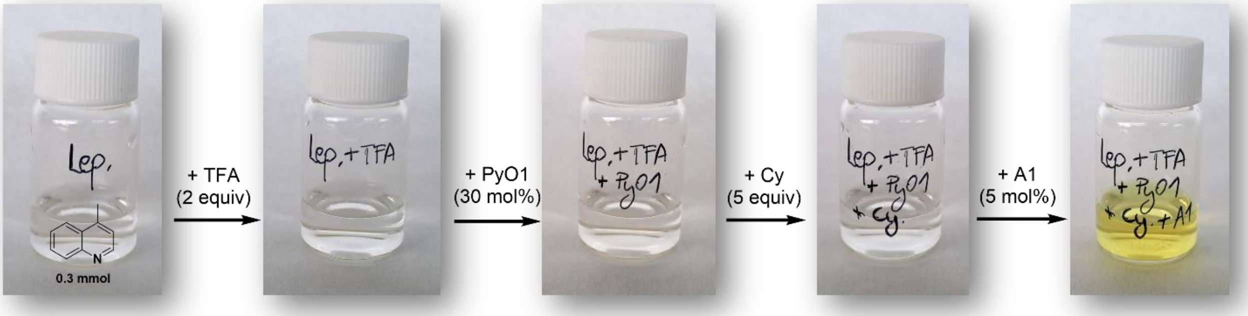

Figure S9: Visual comparison of a solution containing 0.3 mmol of lepidine in MeCN (3 mL), and after sequential additions of TFA (2 equiv), **PyO1** (30 mol%), cyclohexane (5 equiv) and **A1** (5 mol%).

Quantum Yield measurement

A modification of a reported method was used:[11]

Determination of the photon flux:

The photon flux was measured for the conditions used in our photochemical setup using a two-dram vial in a PhotoRedOX Box Duo reactor (Evoluchem™) equipped with two 18 W EvoluChem lamps radiating at 455 nm. The photoreduction of Fe(III) to Fe(II) was monitored by determining the absorbance at 510 nm of the complex with 1,10-phenanthroline:

Actinometer solution (0.10 M): A 10 mL volumetric flask was charged with potassium ferrioxalate trihydrate (491,2 g, 1 mmol), distilled H_2O, and H_2SO_4 96% (0.2 mL, 3.7 mmol).

A buffered solution of phenanthroline (0.0055 M) was prepared in a 100 mL volumetric flask by dissolving phenanthroline (100 mg, 0.554 mmol), sodium acetate (11.25 g, 137 mmol), **H_2SO_4** (96%, 1.3 mL, 25 mmol), and distilled water (pH 4).

A sample of the Actinometer solution (2 mL) is illuminated for a specific time (5 s, + 5 s, + 5 s). After each irradiation, an aliquot (50 μL) is transferred to a 10 mL volumetric flask, where 4 mL of phenanthroline solution is added and raised to 10 mL. A blank sample was prepared following the same procedure but keeping the sample in the dark.

To determine the fraction of light absorbed at 455 nm by the actinometer, a sample of this solution is measured at 455 nm (without dilution).

The absorbance of the solution was measured at 510 nm. A non-irradiated sample was also prepared, and the absorbance was measured at 510 nm. The formation of Fe(II) was calculated using eq 1.1.

- Quantum yield for the photoreduction of ferrioxalate at 455 nm is 1.1 mol× $einstein^{-1}$
- F is the fraction of light absorbed by the actinometer solution at 455 nm:

$$F = 1 - 10^{-A(455\,nm)}$$

- The reaction rate can be calculated with the data obtained at different irradiation times, after sequential transfer of samples (supposing that in the dark no reaction)

$$mol(Fe(II)) = \frac{\Delta A\ (510)}{\epsilon\ d}\ 2\ mL\ \frac{10\ mL}{0.05\ mL} 10^{\ -3} \qquad \boldsymbol{Eq\ 1.1}$$

$$mol(Fe(II)) = \frac{\Delta A\ (510)}{1.11x10^4\ M^{-1}cm^{-1}\ x\ 1cm}\ 2\ mL\ \frac{10\ mL}{0.05\ mL}\ 10^{\ -3} = 3.6036 \cdot 10^{-5} \cdot \Delta A\ (510) \quad \boldsymbol{Eq\ 1.2}$$

$$Photon\ flux\ \left(\frac{einstein}{s}\right) = \frac{Reaction\ rate\ (\frac{mol}{s})}{Quantum\ yield\ (\frac{mol}{einstein}) \times F} \qquad \boldsymbol{Eq. 2.1}$$

$$Photon\ flux\ \left(\frac{einstein}{s}\right) = \frac{2.1364 \cdot 10^{-6}\ (\frac{mol}{s})}{1.1\ (\frac{mol}{einstein}) \times 0.9769} = 1.98804 \cdot 10^{-6}\ Einstein \cdot s^{-1} \qquad \boldsymbol{Eq. 2.2}$$

Determination of the quantum yield of the reaction:

The quantum yield was calculated as follows:

$$\emptyset_{455\ nm}(\%) = \frac{mol\ of\ \mathbf{1}}{t\ (s) \times photon\ flux\ (einstein \bullet s^{-1}) \times F} \times 100 \qquad \boldsymbol{Eq. 3.1}$$

Where mol of product **1** represents the amount of product generated after the irradiation time (t in seconds). The fraction of light absorbed (F) by the protonated photocatalyst at 455 nm was considered 1. Thus, after three measurements, the data obtained was:

Run	Irradiation time (s)	Mol of product	Quantum yield
1	600	0.0003	25.15%
2	1200	0.0006	25.15%
3	1800	0.0009	25.15%

$$\boldsymbol{\emptyset_{455\ nm}(\%) = 25\%} \qquad \boldsymbol{Eq. 3.2}$$

Other mechanistic studies

TEMPO addition

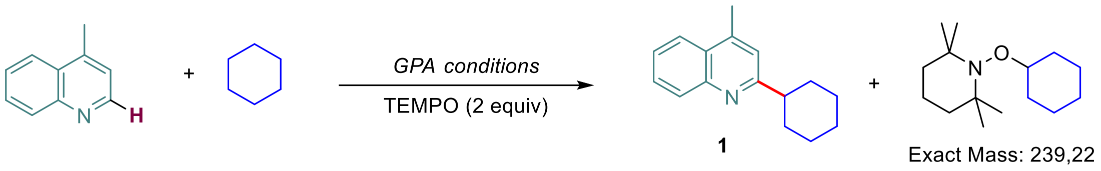

In a two-dram vial was added the lepidine (43 µL, 0.30 mmol), pyridine *N*-oxide (9 mg, 0.09 mmol, 30 mol%), and **A1** (4.5 mg, 0.005 mmol, 5 mol%), followed by a mixture of acetonitrile/1,1,1,3,3,3-hexafluoro-2-propanol (7:3, 3 mL). Then TFA (46 µL, 0.60 mmol, 2 equiv), cyclohexane (161 µL, 3 mmol, 5 equiv) and TEMPO (94 mg, 0.6 mmol, 2 equiv) were added to the reaction mixture. Once the time elapsed, the reaction mixture was analyzed by GC-MS (ESI), observing the formation of the TEMPO-cyclohexyl adduct (Figure S10).

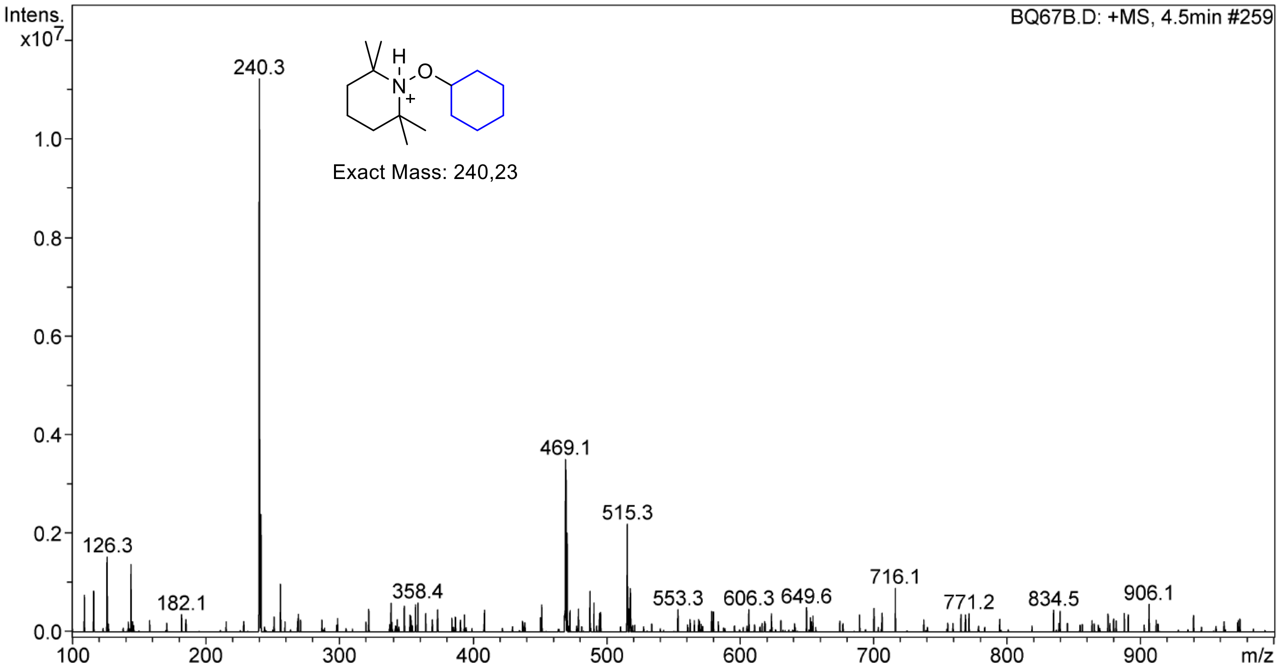

Figure S10: MS for the TEMPO-Cy adduct.

1,1-Diphenylethylene addition

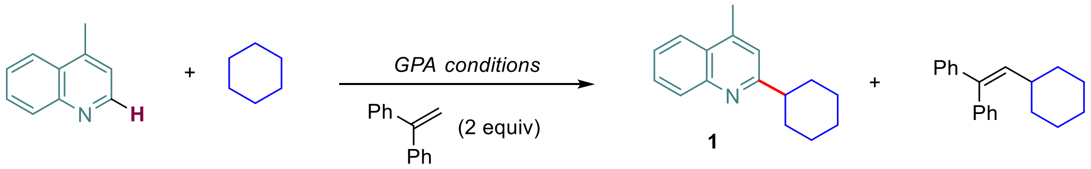

In a two-dram vial was added the lepidine (43 µL, 0.30 mmol), pyridine *N*-oxide (9 mg, 0.09 mmol, 30 mol%), and **A1** (4.5 mg, 0.005 mmol, 5 mol%), followed by a mixture of acetonitrile/1,1,1,3,3,3-hexafluoro-2-propanol

(7:3, 3 mL). Then TFA (46 µL, 0.60 mmol, 2 equiv), cyclohexane (161 µL, 3 mmol, 5 equiv) and 1,1-diphenylethylene (105 µL, 0.6 mmol, 2 equiv) were added to the reaction mixture. Once the time elapsed, the reaction mixture was analyzed by GC-MS (ESI) (Figure S12), observing the formation of the 1-cyclohexyl-2,2-diphenylethylene adduct. The adduct was isolated (together with 1,1-diphenylethylene) and analyzed by ^{1}H-NMR (Figure S11).

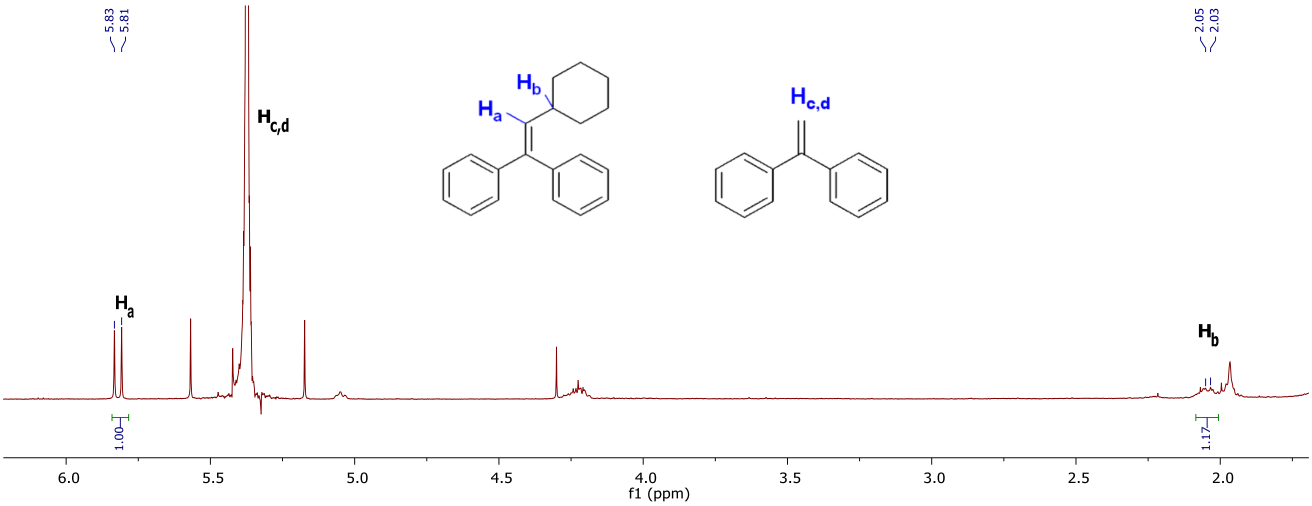

Figure S11: ^{1}H-NMR of the 1-cyclohexyl-2,2-diphenylethylene adduct isolated together with 1,1-diphenylethylene.

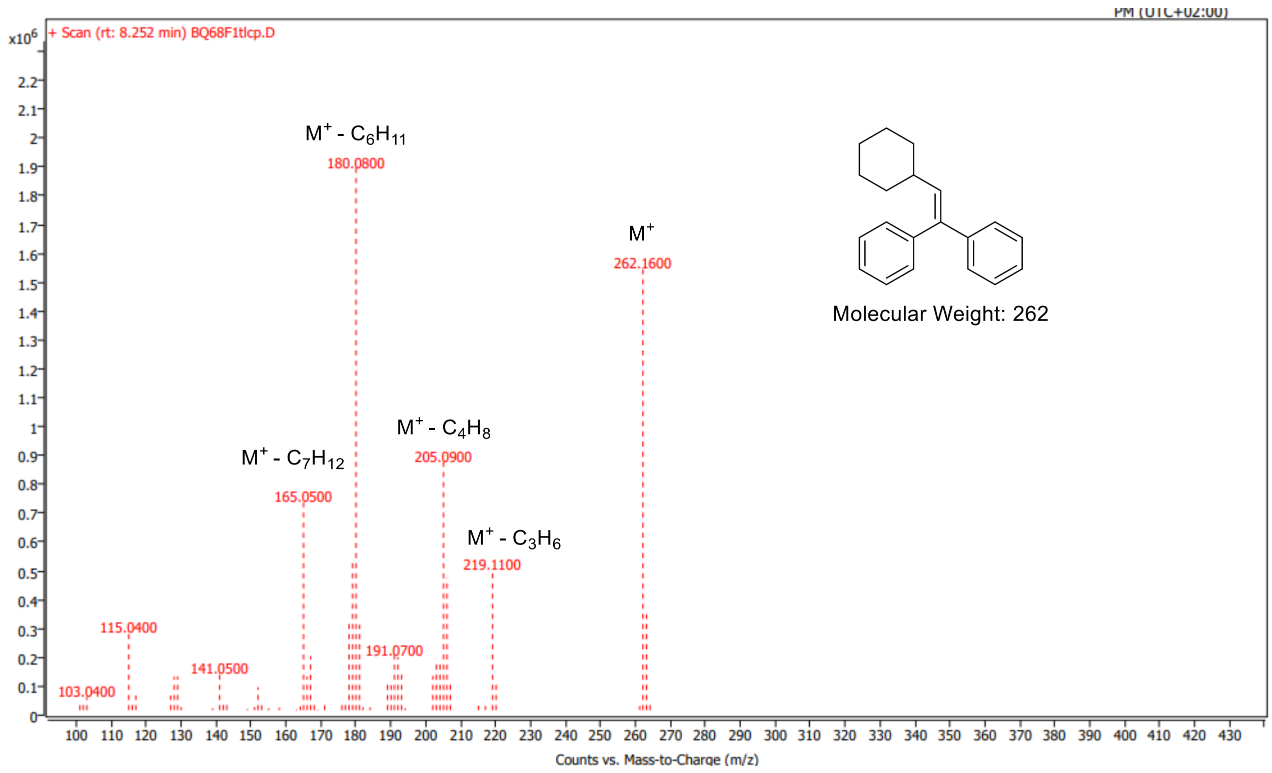

Figure S12: Mass-spectroscopy of the 1-cyclohexyl-2,2-diphenylethylene adduct.

Dehydrogenation of intermediate **20-H$_2$** under the reaction conditions

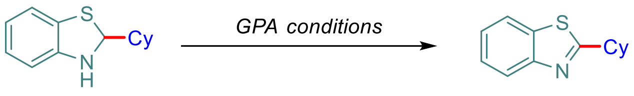

In a two-dram vial was added the 2-cyclohexyl-2,3-dihydrobenzo[d]thiazole (50 mg, 0.23 mmol), pyridine *N*-oxide (7 mg, 0.07 mmol, 30 mol%), and **A1** (4 mg, 0.004 mmol, 5 mol%), followed by a mixture of acetonitrile/1,1,1,3,3,3-hexafluoro-2-propanol (7:3, 2.5 mL). Then TFA (36 µL, 0.46 mmol, 2 equiv) was added to the reaction mixture. After 24 h, an aliquot of 170 µL was taken, dissolved in ethyl acetate up to 1.5 mL and injected into the GC. Complete conversion into the corresponding product was observed.

Reaction in the absence of O_2.

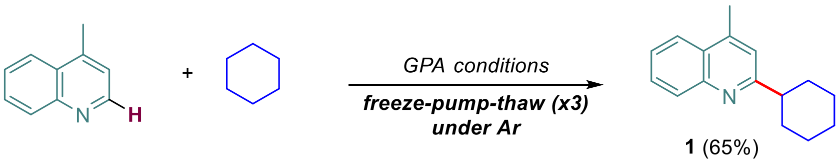

In a two-dram vial was added the lepidine (43 µL, 0.30 mmol), pyridine *N*-oxide (9 mg, 0.09 mmol, 30 mol%), and **A1** (4.5 mg, 0.005 mmol, 5 mol%), followed by a mixture of acetonitrile/1,1,1,3,3,3-hexafluoro-2-propanol (7:3, 3 mL). Then TFA (46 µL, 0.60 mmol, 2 equiv) and cyclohexane (161 µL, 1.5 mmol, 5 equiv) were added to the reaction mixture. The vial was capped with a septum. The reaction was then set under Argon using the freeze-pump-thaw method (three times) to remove the O_2 from the reaction vessel. After 48 h, the reaction mixture was analyzed by GC-MS (ESI), observing the formation of the corresponding product in a 65% yield.

H_2 detection experiments

GC detection of H_2 gas:

The reaction run under Ar atmosphere (see above) was used to detect H_2 gas. Once the reaction elapsed and before analyzing the yield of product **1**, 250 µL of the headspace were injected into a gas chromatograph having a TCD. Test injections in the absence and in the presence of 1% of H_2 were performed to confirm the presence of H_2 gas in the headspace of the reaction mixture. The carrying gas used in the GC was He, giving a negative signal on detecting H_2 gas (Figure S13).

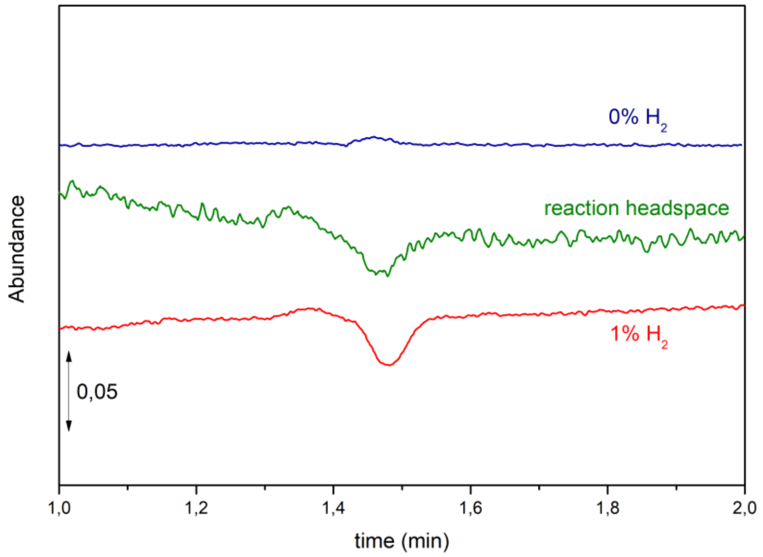

Figure S13: GC with a TCD in a sample containing no H_2 (blue, top), 1% of H_2 (red, bottom) and the headspace of a reaction between lepidine and cyclohexane following GPA (middle, green).

Visual observation:

This phenomenon was observed in every successful reaction that were set up in the flow system:

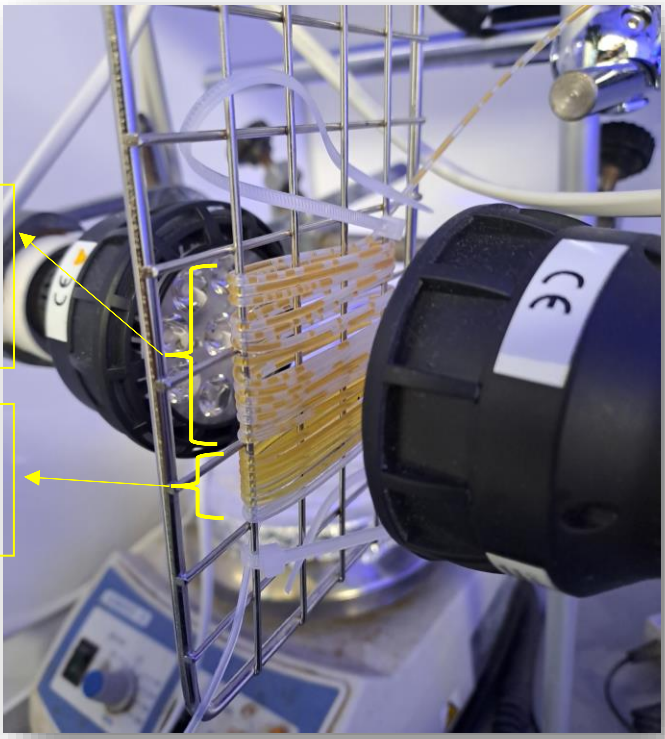

Figure S14: Visual observation of H_2 gas evolution

Kinetic Isotopic Experiments (KIE)

Individual/parallel runs for products **1** and **1-D11**:

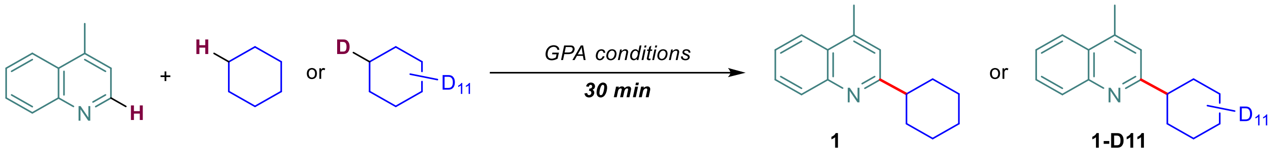

In a two-dram vial was added the lepidine (43 µL, 0.30 mmol), pyridine *N*-oxide (9 mg, 0.09 mmol, 30 mol%), and **A1** (4.5 mg, 0.005 mmol, 5 mol%), followed by a mixture of acetonitrile/1,1,1,3,3,3-hexafluoro-2-propanol (7:3, 3 mL). Then TFA (46 µL, 0.60 mmol, 2 equiv) and cyclohexane (161 µL, 1.5 mmol, 5 equiv) were added to the reaction mixture. The same reaction was set up in a separate two-dram vial using cyclohexane-D_{12} instead. From both experiments, aliquots of 170 µL were taken after 10, 20 and 30 min. These aliquots were diluted up to 1.5 mL with ethyl acetate and injected into the GC-MS.

Table S8: Yield and concentration of **1** and **1-D$_{11}$** (measured by GC) at the initial stages of the reactions.

time (min)	yield of **1** (%)	[**1**] /mM	yield of **1-D$_{11}$**(%)	[**1-D$_{11}$**] /mM
10	9,8	9,8	8,1	8,1
20	20,9	20,9	16,3	16,3
30	30,3	30,3	24,4	24,4

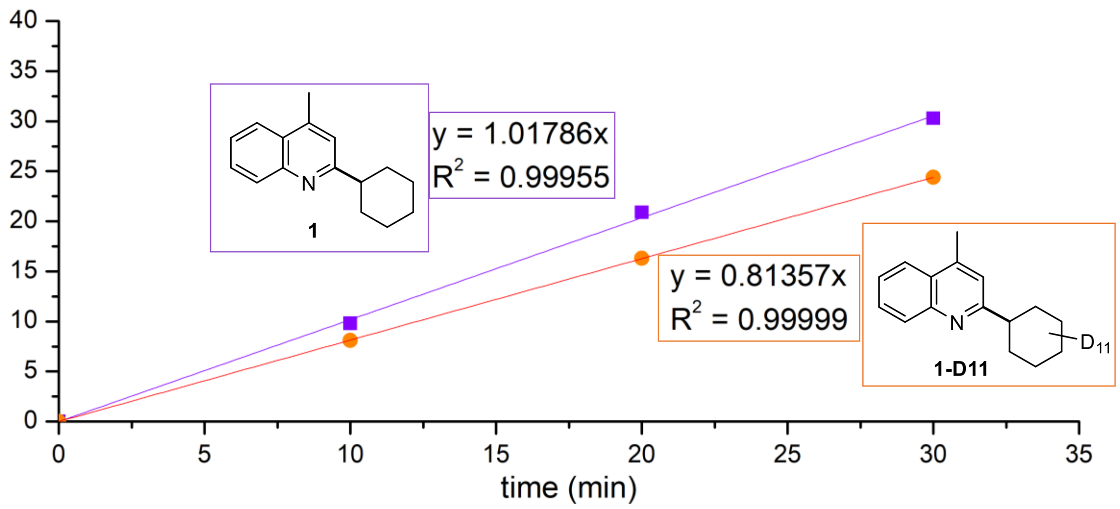

Figure S15. Initial rate of formation of **1** or **1-D$_{11}$**

For product **1**: k_H = 1.01786 mM·min^{-1}
For product **1-D$_{11}$**: k_D = 0.81357 mM·min^{-1}

$$\boldsymbol{KIE_{ind-Lep} = \frac{k_H}{k_D} = 1.3} \qquad \boldsymbol{Eq. 4}$$

Competitive runs for products **1** and **1-D$_{11}$**:

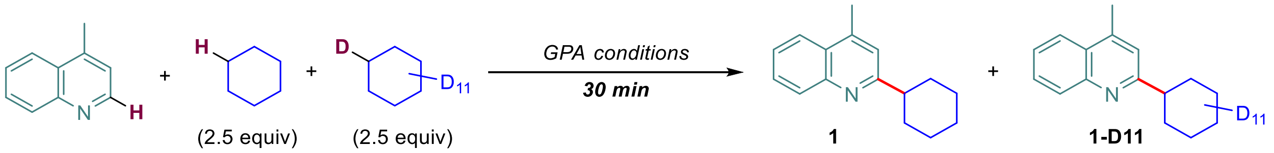

In a two-dram vial was added the lepidine (43 µL, 0.30 mmol), pyridine *N*-oxide (9 mg, 0.09 mmol, 30 mol%), and **A1** (4.5 mg, 0.005 mmol, 5 mol%), followed by a mixture of acetonitrile/1,1,1,3,3,3-hexafluoro-2-propanol (7:3, 3 mL). Then TFA (46 µL, 0.60 mmol, 2 equiv), cyclohexane (80 µL, 0.75 mmol, 2.5 equiv) and cyclohexane-D$_{12}$ (80 µL, 0.75 mmol, 2.5 equiv) were added. Once this time elapsed, K_2CO_3 was added (200 g, 1.5 mmol, 5 equiv), and the reaction mixture was stirred for 30 min. After this time, the suspension was filtered through Celite, eluting with EtOAc (4 x 5 mL). After evaporating the solvent under vacuum, the residue was purified by FC (1-5% EtOAc in Hexane).

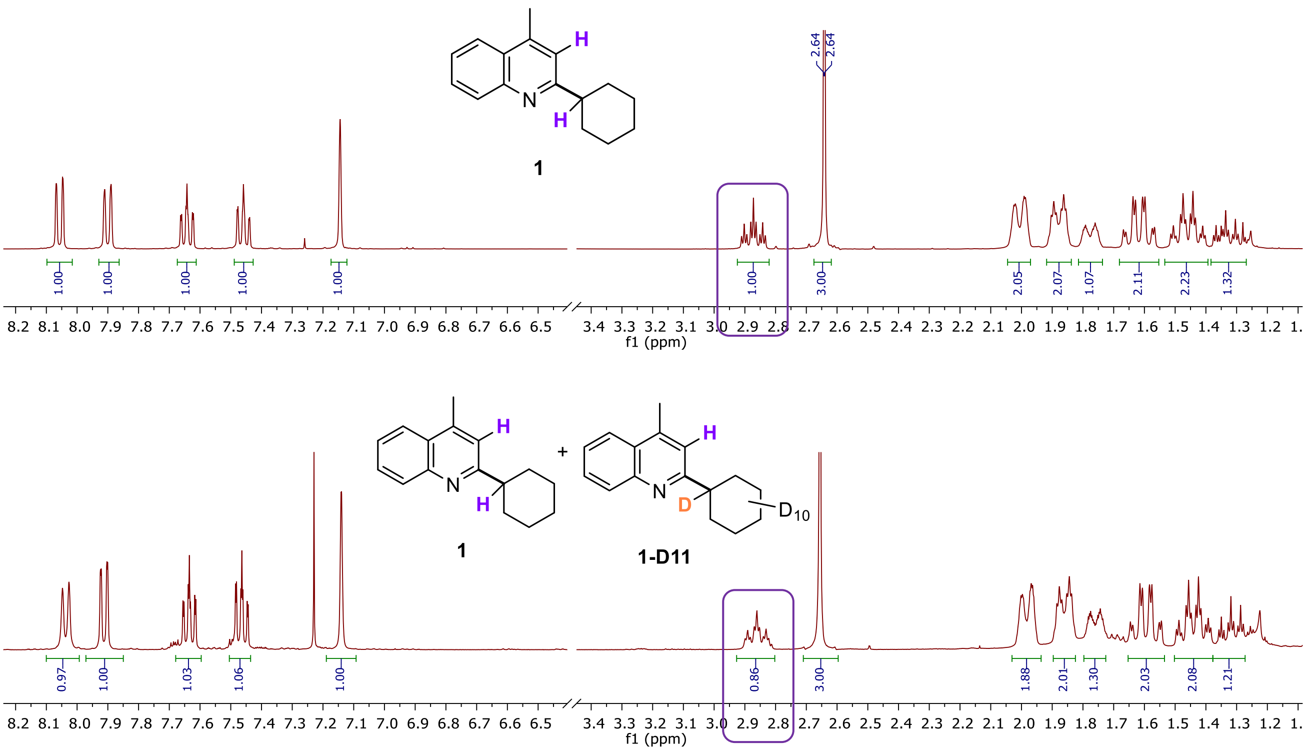

Figure S16: ^{1}H-NMR of pure product **1** (top) and mixture of **1** and **1-D$_{11}$** from the competitive KIE experiment (bottom).

Integral for product **1**: 0.86

Integral missing for product **1-D$_{11}$**: 1-0.86 = 0.14

$$\boldsymbol{KIE_{comp-Lep}} = \frac{product\ with\ H}{product\ with\ D} = \mathbf{6.1} \qquad \boldsymbol{Eq. 5}$$

Individual/parallel runs for products 20 and 20-D11:

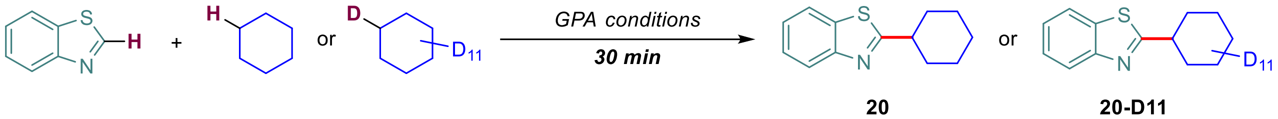

In a two-dram vial was added benzothiazole (33 µL, 0.30 mmol), pyridine *N*-oxide (9 mg, 0.09 mmol, 30 mol%), and **A1** (4.5 mg, 0.005 mmol, 5 mol%), followed by a mixture of acetonitrile/1,1,1,3,3,3-hexafluoro-2-propanol (7:3, 3 mL). Then TFA (46 µL, 0.60 mmol, 2 equiv) and cyclohexane (161 µL, 1.5 mmol, 5 equiv) were added to the reaction mixture. The same reaction was set up in a separate two-dram vial using cyclohexane-D12 instead. From both experiments, aliquots of 170 µL were taken after 10, 20 and 30 min. These aliquots were diluted up to 1.5 mL with ethyl acetate and injected into the GC-MS.

Table S9: Yield and concentration of **20** and **20-D$_{11}$** (measured by GC) at the initial stages of the reactions.

time (min)	yield of **20** (%)	[**20**] /mM	yield of **20-D11**(%)	[**20-D11**] /mM
10	3,5	3,5	2,0	2,0
20	7,4	7,4	4,5	4,5
30	11,2	11,2	6,9	6,9

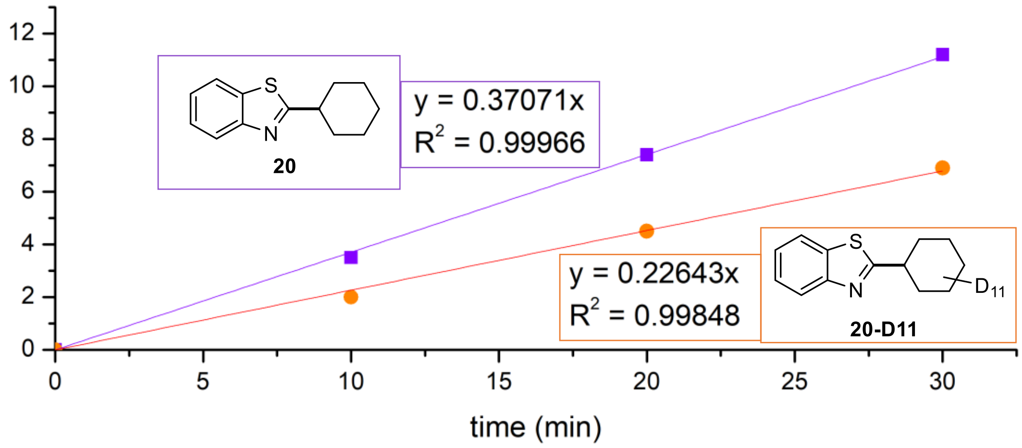

Figure S17. Initial rate of formation of **20** or **20-D$_{11}$**

For product **20**: k_H = 0.37071 mM·min^{-1}

For product **20-D$_{11}$**: k_D = 0.22643 mM·min^{-1}

$$\boldsymbol{KIE_{ind-Bzthia} = \frac{k_H}{k_D} = 1.6} \qquad \textbf{\textit{Eq. 6}}$$

Competitive runs for products **20** and **20-D11**:

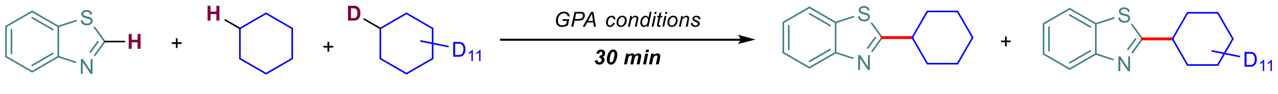

In a two-dram vial was added the benzothiazole (33 µL, 0.30 mmol), pyridine *N*-oxide (9 mg, 0.09 mmol, 30 mol%), and **A1** (4.5 mg, 0.005 mmol, 5 mol%), followed by a mixture of acetonitrile/1,1,1,3,3,3-hexafluoro-2-propanol (7:3, 3 mL). Then TFA (46 µL, 0.60 mmol, 2 equiv), cyclohexane (80 µL, 0.75 mmol, 2.5 equiv) and cyclohexane-D12 (75 µL, 0.75 mmol, 2.5 equiv) were added. Once this time elapsed, K_2CO_3 was added (200 g, 1.5 mmol, 5 equiv), and the reaction mixture was stirred for 30 min. After this time, the suspension was filtered through Celite, eluting with EtOAc (4 x 5 mL). After evaporating the solvent under vacuum, the residue was purified by FC 5-10% EtOAc in Hexane.

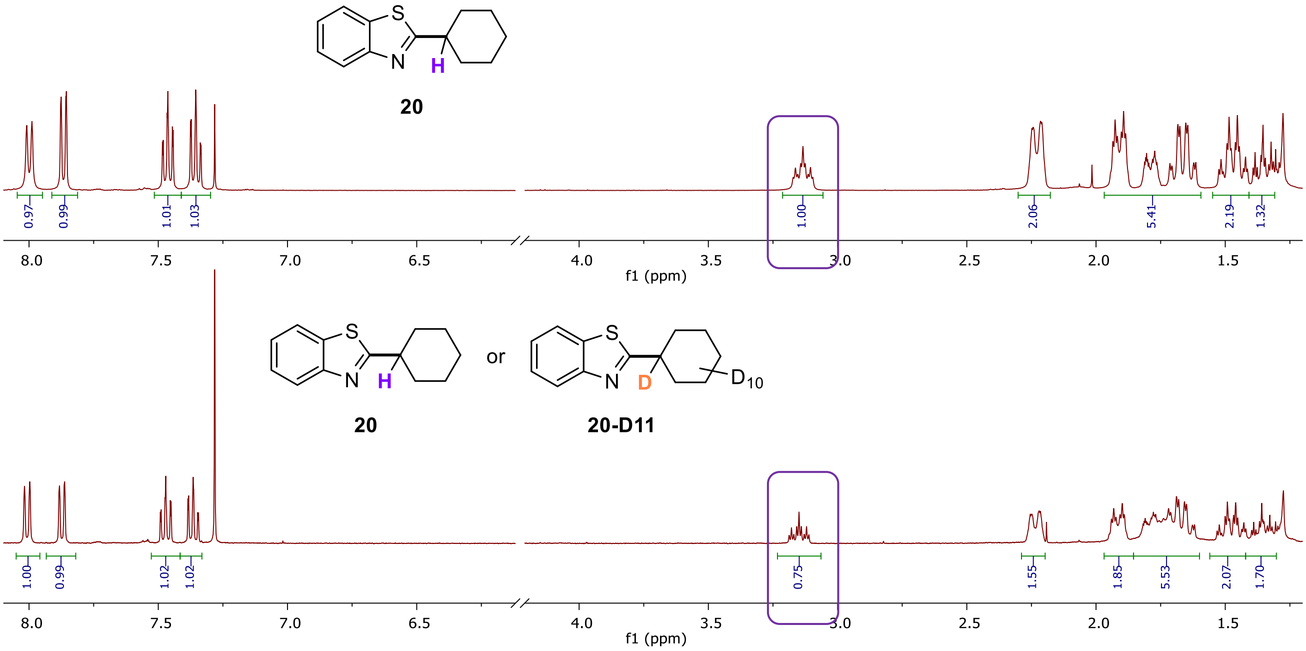

Figure S18: ^{1}H-NMR of pure product **20** (top) and mixture of **20** and **20-D₁₁** from the competitive KIE experiment (bottom).

Integral for product **1**: 0.75

Integral missing for product **1-D**$_{11}$: 1-0.75 = 0.25

$$KIE_{comp-Bzthia} = \frac{product\ H}{product\ D} = 3.0 \qquad Eq. 7$$

Characterization of Products

Alkylation of Lepidine with cyclohexane

Following the GP with lepidine (43 µL, 0.30 mmol) and cyclohexane (162 µL, 1.50 mmol, 5 equiv) in 32 h. The product was obtained as a light-yellow oil (51.5 mg, 0.23 mmol, 76%) after FC using a gradient from 0% to 5% of EtOAc in n-hexane as the eluent. The spectroscopy data matched with previously reported in the literature.[12]

2-Cyclohexyl-4-methylquinoline (**1**):

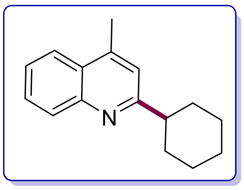

TLC: Rf = 0.4 (95:5 hexane/EtOAc, UV).

GC (Ti= 80 ºC): Rt 8.07 min.

MS: *m/z* (%) 225 (M^+, 23), 224 (17), 210 (12), 196 (26), 184 (14), 171 (27), 157 (48).

^{1}H NMR (400 MHz, $CDCl_3$): *δ* 8.06 (dd, *J* = 8.6, 1.3 Hz, 1H), 7.90 (dd, *J* = 8.3, 1.4 Hz, 1H), 7.64 (ddd, *J* = 8.4, 6.8, 1.4 Hz, 1H), 7.46 (ddd, *J* = 8.2, 6.8, 1.3 Hz, 1H), 7.14 (d, *J* = 1.0 Hz, 1H), 2.87 (tt, *J* = 12.0, 3.4 Hz, 1H), 2.64 (d, *J* = 1.0 Hz, 3H), 2.04 – 1.97 (m, 2H), 1.88 (dt, *J* = 12.7, 3.3 Hz, 2H), 1.82 – 1.73 (m, 1H), 1.62 (qd, *J* = 12.4, 3.2 Hz, 2H), 1.46 (qt, *J* = 12.5, 3.2 Hz, 2H),1.39 – 1.23 (m, 1H).

^{13}C NMR (101 MHz, $CDCl_3$): *δ* 166.5, 147.6, 144.3, 129.5, 128.9, 127.0, 125.4, 123.6, 120.2, 47.6, 32.8, 26.6, 26.1, 18.9.

Alkylation of Quinaldine with Cyclohexane

Following the GP with 2-methylquinoline (43 µL, 0.30 mmol) and cyclohexane (162 µL, 1.5 mmol, 5 equiv) in 60 h. After purification by FC, the product was obtained as a colorless oil (54.6 mg, 0.24 mmol, 81%) using 0% to 5% EtOAc in *n*-hexane as the eluent. The spectroscopy data matched with previously reported in the literature.[12]

4-Cyclohexyl-2-methylquinoline (2):

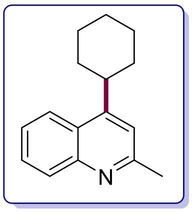

TLC: Rf = 0.27 (9:1 hexane/EtOAc, UV).

GC (Ti= 80 ºC): Rt 8.079 min.

MS: *m/z* (%) 225 (M^+, 100), 224 (23), 210 (31), 182 (35), 170 (42), 169 (24), 157 (60).

^{1}H NMR (300 MHz, $CDCl_3$): δ 8.09 – 8.01 (m, 2H), 7.65 (ddd, *J* = 8.4, 6.9, 1.4 Hz, 1H), 7.49 (ddd, *J* = 8.3, 6.8, 1.3 Hz, 1H), 7.17 (s, 1H), 3.39 – 3.22 (m, 1H), 2.73 (s, 3H), 2.04 – 1.81 (m, 5H), 1.67 – 1.37 (m, 5H).

^{13}C NMR (101 MHz, $CDCl_3$): *δ* 158.8, 153.8, 147.9, 129.4, 129.1, 125.5, 125.3, 122.9, 118.5, 38.9, 33.7, 27.1, 26.4, 25.5.

Alkylation of 4-Phenylquinoline with Cyclohexane

Following the GP (but adding 20 mol% of pyridine *N*-oxide at the beginning of the reaction and an extra 10 mol% after 24 h) with 4-phenyloquinoline (62 µL, 0.30 mmol) and cyclohexane (162 µL, 1.5 mmol, 5 equiv)

in 46 h. After purification by FC, the product was obtained as a colorless oil (76.6 mg, 0.27 mmol, 89%) using from 5% to 10% EtOAc in *n*-hexane as the eluent. The spectroscopy data matched with previously reported in the literature.[13]

2-Cyclohexyl-4-phenylquinoline (3):

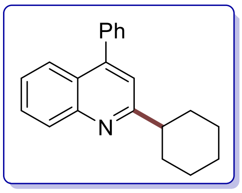

TLC: Rf = 0.46 (9:1 hexane/EtOAc, UV).

GC (Ti= 80 ºC): Rt 10.029 min.

MS: *m/z* (%) 287 (M^+, 32), 232 (100), 219 (66), 232 (39), 287 (28), 258 (26), 286 (19).

^{1}H NMR (400 MHz, $CDCl_3$): δ 8.14 (ddd, *J* = 8.5, 1.2, 0.6 Hz, 1H), 7.87 (ddd, *J* = 8.4, 1.5, 0.6 Hz, 1H), 7.68 (ddd, *J* = 8.4, 6.8, 1.4 Hz, 1H), 7.54 – 7.47 (m, 5H), 7.42 (ddd, *J* = 8.2, 6.8, 1.3 Hz, 1H), 7.28 (s, 1H), 2.98 (tt, *J* = 12.1, 3.4 Hz, 1H), 2.08 (dtt, *J* = 13.7, 3.8, 1.9 Hz, 2H), 1.91 (dt, *J* = 12.9, 3.3 Hz, 2H), 1.80 (dtt, *J* = 12.8, 3.2, 1.5 Hz, 1H), 1.67 (qd, *J* = 12.5, 3.2 Hz, 2H), 1.49 (qt, *J* = 12.7, 3.3 Hz, 2H), 1.35 (tt, *J* = 12.7, 3.4 Hz, 1H).

^{13}C NMR (101 MHz, $CDCl_3$): δ 166.4, 148.8, 148.3, 138.6, 129.6, 129.4, 129.3, 128.6, 128.4, 125.8, 125.7, 125.6, 119.9, 47.7, 33.0, 26.7, 26.2.

Alkylation of 2-Phenylquinoline with Cyclohexane

Following the GP with 2-phenylquinoline (62 mg, 0.30 mmol) and cyclohexane (162 µL, 1.5 mmol, 5 equiv) in 46 h. After purification by FC, the product was obtained as a colorless oil (57.7 mg, 0.20 mmol, 67%) using 0% to 5% EtOAc in *n*-hexane as the eluent. The spectroscopy data matched with previously reported in the literature.[13]

4-Cyclohexyl-2-phenylquinoline (4):

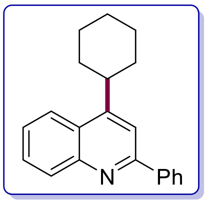

TLC: Rf = 0.51 (9:1 hexane/EtOAc, UV).

GC (Ti= 80 ºC): Rt 10.779 min.

MS: *m/z* (%) 287 (M^+,100), 232 (75), 286 (68), 219 (37), 288 (29), 230 (26), 244 (219.

^{1}H NMR (400 MHz, $CDCl_3$): δ 8.28 – 8.03 (m, 4H), 7.76 (s, 1H), 7.71 (ddd, *J* = 8.4, 6.8, 1.4 Hz, 1H), 7.54 (dddd, *J* = 7.7, 6.1, 2.8, 1.5 Hz, 3H), 7.50 – 7.41 (m, 1H), 3.48 – 3.23 (m, 1H), 2.09 (d, *J* = 9.9 Hz, 2H), 2.01 – 1.83 (m, 3H), 1.70 – 1.50 (m, 4H), 1.45 – 1.31 (m, 1H).

^{13}C NMR (101 MHz, $CDCl_3$): δ 157.3, 154.2, 148.4, 140.1, 130.6, 129.2, 129.1, 128.8, 127.7, 126.0, 125.9, 122.8, 115.5, 39.2, 33.7, 26.9, 26.3.

Alkylation of 2-Chloroquinoline with Cyclohexane

Following the GP (but adding 20 mol% of pyridine *N*-oxide at the beginning of the reaction and an extra 10 mol% after 24 h) with 2-chloroquinoline (49 mg, 0.30 mmol) and cyclohexane (324 µL, 3 mmol, 10 equiv) in 46 h. After purification by FC, the product was obtained as a colorless oil (30.1 mg, 0.12 mmol, 41%) using 0% to 1% EtOAc in *n*-hexane as the eluent. The spectroscopy data matched with previously reported in the literature.[14]

2-Chloro-4-cyclohexylquinoline (5):

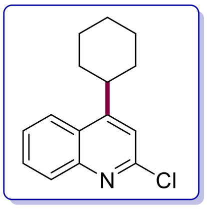

TLC: Rf = 0.62 (95:5 hexane/EtOAc, UV).

GC (Ti= 80 ºC): Rt 8.664 min.

MS: *m/z* (%) 247 (M^+,^{37}Cl, 32), 245 (100), 210 (38), 207 (19), 202 (19), 191 (20), 190 (47), 189 (39), 177 (60), 154 (30), 1228 (22).

^{1}H NMR (400 MHz, $CDCl_3$): δ 8.03 (ddd, *J* = 8.5, 7.1, 1.3 Hz, 2H), 7.70 (ddd, *J* = 8.3, 6.9, 1.4 Hz, 1H), 7.56 (ddd, *J* = 8.4, 6.9, 1.4 Hz, 1H), 7.26 (s, 1H), 3.29 (tt, *J* = 8.4, 3.3 Hz, 1H), 2.04 – 1.90 (m, 4H), 1.89 – 1.80 (m, 1H), 1.60 – 1.49 (m, 4H), 1.38 – 1.29 (m, 1H).

^{13}C NMR (101 MHz, $CDCl_3$): δ 157.1, 151.2, 148.3, 130.1, 129.6, 126.7, 125.4, 123.3, 118.8, 39.2, 33.5, 26.9, 26.3.

Alkylation of 4,7-Dichloroquinoline with Cyclohexane

Following the GP (but adding 20 mol% of pyridine *N*-oxide at the beginning of the reaction and an extra 10 mol% after 24 h) with 4,7-dichloroquinoline (59 mg, 0.30 mmol) and cyclohexane (324 µL, 3 mmol, 10 equiv) in 46 h. After purification by FC, the product was obtained as a colorless oil (54.4 mg, 0.19 mmol, 65%) using 0% to 5% EtOAc in *n*-hexane as the eluent. The spectroscopy data matched with previously reported in the literature.[15]

4,7-Dichloro-2-cyclohexylquinoline (6):

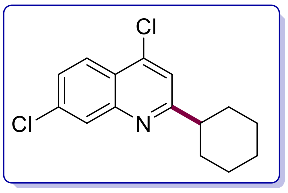

TLC: Rf = 0.47 (9:1 hexane/EtOAc, UV).

GC (Ti= 80 ºC): Rt 8.899 min.

MS: *m/z* (%) 283 (M^+, ^{37}Cl/^{37}Cl, 3), 281 (M^+, ^{35}Cl/^{37}Cl, 15) 279 (M^+, ^{35}Cl/^{35}Cl, 22), 278 (17), 252 (19), 250 (29), 244 (29), 226 (70), 225 (26).

^{1}H NMR (300 MHz, $CDCl_3$): δ 8.09 (d, *J* = 8.7 Hz, 1H), 8.05 (dd, *J* = 2.1, 0.5 Hz, 1H), 7.51 (dd, *J* = 8.9, 2.1 Hz, 1H), 7.40 (s, 1H), 2.85 (tt, *J* = 11.9, 3.4 Hz, 1H), 2.01 – 1.95 (m, 2H), 1.93 – 1.85 (m, 2H), 1.82 – 1.75 (m, 1H), 1.67 – 1.53 (m, 2H), 1.50 – 1.41 (m, 2H), 1.35 – 1.24 (m, 1H).

^{13}C NMR (101 MHz, $CDCl_3$): δ 168.3, 149.2, 142.7, 136.3, 128.5, 127.7, 125.4, 123.7, 120.8, 47.4, 32.7, 26.5, 26.1.

Alkylation of 4-Methoxyquinoline with Cyclohexane

Following the GP with 4-methoxyquinoline (43 µL, 0.30 mmol) and cyclohexane (162 µL, 1.5 mmol, 5 equiv) in 46 h. After purification by FC, the product was obtained as a colorless oil (30.3 mg, 0.13 mmol, 42%) using 0% to 10% EtOAc in *n*-hexane as the eluent. The spectroscopy data matched with previously reported in the literature.[13]

2-Cyclohexyl-4-methoxyquinoline (7):

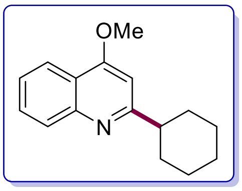

TLC: Rf = 0.34 (9:1 hexane/EtOAc, UV).

GC (Ti= 80 ºC): Rt: 8.545 min.

MS: *m/z* (%) 241 (M^+, 21), 186 (100), 173 (30), 187 (25), 241 (21), 212 (19).

^{1}H NMR (400 MHz, $CDCl_3$): *δ* 8.13 (ddd, *J* = 8.3, 1.5, 0.6 Hz, 1H), 7.99 (dt, *J* = 8.5, 0.9 Hz, 1H), 7.64 (ddd, *J* = 8.5, 6.9, 1.5 Hz, 1H), 7.42 (ddd, *J* = 8.1, 6.8, 1.2 Hz, 1H), 6.64 (s, 1H), 4.03 (s, 3H), 2.88 (tt, *J* = 12.0, 3.4 Hz, 1H), 2.03 (dtd, *J* = 14.3, 3.5, 1.7 Hz, 2H), 1.89 (dq, *J* = 12.9, 3.3 Hz, 2H), 1.79 (dtt, *J* = 12.6, 3.2, 1.5 Hz, 1H), 1.62 (qd, *J* = 12.4, 3.2 Hz, 2H), 1.47 (qt, *J* = 12.6, 3.2 Hz, 2H), 1.33 (qt, *J* = 12.5, 3.2 Hz, 1H).

^{13}C NMR (101 MHz, $CDCl_3$): *δ* 168.2, 162.7, 148.6, 129.7, 128.4, 124.9, 121.6, 120.4, 98.0, 55.6, 48.2, 33.0, 26.6, 26.2.

Alkylation of Quinoline with Cyclohexane

Following the GP (but adding 20 mol% of pyridine *N*-oxide at the beginning of the reaction and an extra 10 mol% after 24 h) with methyl quinoline-4-carboxylate (56 mg, 0.30 mmol) and cyclohexane (162 µL, 1.5 mmol, 5 equiv) in 46 h. After purification by FC, the product was obtained as a yellow oil (45 mg, 0.17 mmol, 56 %) using 1% to 5% EtOAc in *n*-hexane as the eluent. The spectroscopy data matched with previously reported in the literature.[16]

Methyl 2-cyclohexylquinoline-4-carboxylate (8):

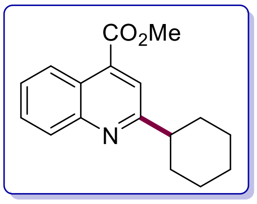

TLC: Rf = 0.56 (95:5 Hexane/EtOAc), UV).

GC (Ti= 80 ºC): 8.9 min.

MS: m/z (%) 214 (M^+, 100) 202 (61), 210 (37), 215 (27), 254 (21), 240 (20).

^{1}H NMR (400 MHz, $CDCl_3$): δ 8.67 (ddd, *J* = 8.6, 1.5, 0.6 Hz, 1H), 8.10 (ddd, *J* = 8.5, 1.3, 0.6 Hz, 1H), 7.83 (s, 1H), 7.71 (ddd, *J* = 8.4, 6.9, 1.4 Hz, 1H), 7.57 (ddd, *J* = 8.3, 6.9, 1.3 Hz, 1H), 4.03 (s, 3H), 2.96 (tt, *J* = 12.1, 3.4 Hz, 1H), 2.04 (dtd, *J* = 13.0, 3.7, 2.1 Hz, 2H), 1.90 (dt, *J* = 12.7, 3.3 Hz, 2H), 1.79 (dddt, *J* = 12.9, 5.0, 3.4, 1.7 Hz, 1H), 1.66 (qd, *J* = 12.4, 3.3 Hz, 2H), 1.47 (qt, *J* = 12.6, 3.2 Hz, 2H), 1.41 – 1.29 (m, 1H).

^{13}C NMR (101 MHz, $CDCl_3$): δ 167.0, 166.2, 148.7, 135.2, 129.55, 129.53, 127.1, 125.3, 123.7, 121.1, 52.6, 47.3, 32.6, 26.4, 26.0.

Alkylation of Quinoline with Cyclohexane

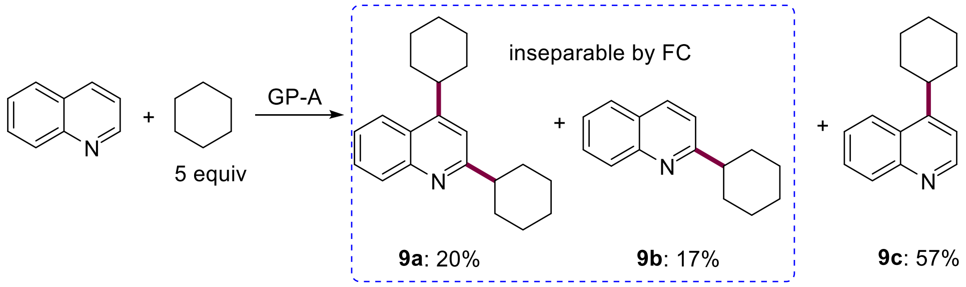

(di:C2:C4 = 22:18:60)

Following the GP with quinoline (36 µL, 0.30 mmol) and cyclohexane (162 µL, 1.5 mmol, 5 equiv) in 46 h. After purification by FC, products **9a** and **9b** were obtained as an inseparable mixture (colorless oil, 28 mg, 0.06 mmol (20%) and 0.05 mmol (17%), respectively) using from 0% to 2% EtOAc in *n*-hexane as the eluent. Elution from 2% to 20% EtOAc in *n*-hexane afforded **9c** as a colorless oil (36.0 mg, 0.17 mmol, 57%).

2,4-Dicyclohexylquinoline (9a):

The spectroscopy data matched with previously reported in the literature.[17]

TLC: Rf = 0.53 (9:1 hexane/EtOAc, UV).

GC (Ti= 80 ºC): Rt 10.057 min.

MS: *m/z* (%) 293 (M^+, 21) 238 (100), 207 (71), 225 (56), 281 (46), 239 (29), 253 (28).

^{1}H NMR (400 MHz, $CDCl_3$, for the mixture of **9a** and **9b**): δ 8.17 – 8.02 (m, 4H), 7.78 (dd, *J* = 8.1, 1.5 Hz, 1H), 7.68 (dddd, *J* = 9.7, 8.3, 6.8, 1.5 Hz, 2H), 7.50 (dddd, *J* = 8.1, 6.8, 5.0, 1.3 Hz, 2H), 7.35 (d, *J* = 8.5 Hz, 1H), 7.23 (s, 1H), 3.32 (tdt, *J* = 11.6, 8.3, 4.2 Hz, 1H), 2.95 (tt, *J* = 12.0, 3.4 Hz, 2H), 2.10 – 1.88 (m, 11H), 1.88 – 1.77 (m, 3H), 1.73 – 1.60 (m, 5H), 1.60 – 1.43 (m, 7H), 1.42 – 1.30 (m, 4H).

^{13}C NMR (101 MHz, $CDCl_3$, for the mixture of **9a** and **9b**): δ 166.9, 166.6, 147.9, 136.4, 131.0, 129.8, 129.3, 129.0, 128.9, 128.8, 127.5, 127.1, 125.8, 125.7, 125.4, 122.9, 119.7, 115.8, 47.7, 33.7, 33.0, 32.9, 27.0, 26.7, 26.6, 26.2.

2-Cyclohexylquinoline (9b):

TLC: Rf = 0.53 (9:1 hexane/EtOAc, UV).

GC (Ti= 80 ºC): Rt 7.530 min.

MS: *m/z* (%) 211 (M+, 28) 156 (100), 143 (57), 182 (32), 157 (23), 210 (19).

4-Cyclohexylquinoline (9c):

The spectroscopy data matched with previously reported in the literature.[18]

TLC: Rf = 0.15 (9:1 hexane/EtOAc, UV).

GC (Ti= 80 ºC): Rt 7.918.

MS: *m/z* (%) 211 (M^+,100), 143 (47), 168 (33), 154 (32), 156 (31), 210 (29), 155 (24), 167 (22).

^{1}H NMR (400 MHz, $CDCl_3$): δ 8.84 (d, *J* = 4.6 Hz, 1H), 8.11 (ddd, *J* = 12.6, 8.5, 1.4 Hz, 2H), 7.69 (ddd, *J* = 8.4, 6.8, 1.4 Hz, 1H), 7.55 (ddd, *J* = 8.3, 6.8, 1.3 Hz, 1H), 7.28 (d, *J* = 4.6 Hz, 1H), 3.33 (tt, *J* = 8.4, 3.2 Hz, 1H), 2.01 (dq, *J* = 10.8, 2.4, 2.0 Hz, 2H), 1.98 – 1.90 (m, 2H), 1.85 (dqd, *J* = 10.8, 3.0, 1.4 Hz, 1H), 1.55 (tdd, *J* = 9.9, 4.9, 2.5 Hz, 4H), 1.36 (ddt, *J* = 12.7, 9.0, 3.6 Hz, 1H).

^{13}C NMR (101 MHz, $CDCl_3$): δ 153.6, 150.2, 148.2, 130.3, 128.9, 126.9, 126.2, 123.0, 117.5, 38.9, 33.5, 26.9, 26.3.

Alkylation of 6-Fluoroquinoline with Cyclohexane

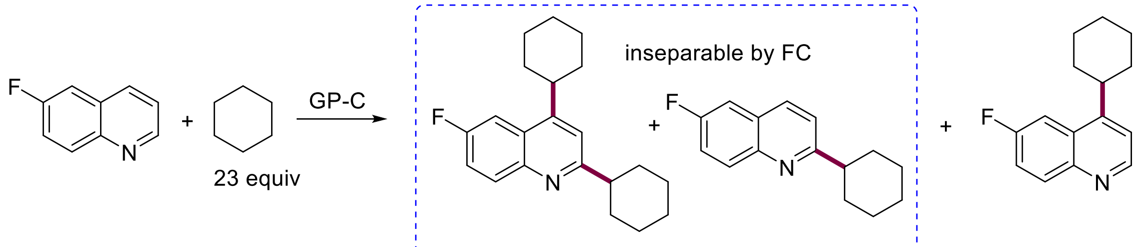

10a: 21% **10b**: 22% **10c**: 26%

(di:C2:C4 = 32:30:38)

Synthesis of 2,4-dicyclohexyl-6-fluoroquinoline, 2-cyclohexyl-6-fluoroquinoline (10a+10b) and 4-Cyclohexyl-6-fluoroquinoline (10c):

Following the GP with 6-fluoroquinoline (37 µL, 0.30 mmol) and cyclohexane (0.7 mL, 6.9 mmol, 23 equiv) in 46 h. After purification by FC, products **10a** and **10b** were obtained as an inseparable mixture (colorless oil, 35 mg, 0.063 mmol (21%) and 0.067 mmol (22%), respectively) using from 0% to 2% EtOAc in *n*-hexane as the eluent. Further, elution with increased polarity (up to 10% EtOAc) furnished **10c** as a colorless oil (18.1 mg, 0.08 mmol, 26%). The products were unreported in the literature.

2,4-Dicyclohexyl-6-fluoroquinoline (10a) and 2-Cyclohexyl-6-fluoroquinoline (10b):

TLC (10a): Rf = 0.52 (9:1 hexane/EtOAc, UV).

GC (10a) (Ti= 80 °C): Rt 9.860 min.

MS (10a): *m/z* (%) 311 (M^+, 17), 256 (100), 243 (61), 257 (28), 282 (15).

HRMS (10a) (EI-TOF): *m/z* calcd for $C_{21}H_{25}N$(-HF) 291.1987, found 291.1983.

TLC (10b): Rf = 0.52 (9:1 hexane/EtOAc, UV).

GC (10b) (Ti= 80 °C): Rt 7.419 min.

MS (10b): *m/z* (%) 229 (M^+, 18), 174 (100), 161 (65), 200 (25), 175 (21).

HRMS (10b) (EI-TOF): *m/z* calcd for $C_{15}H_{15}FN$ (-H) 228,1189, found 228.1202.

^{1}H NMR (400 MHz, $CDCl_3$, for the mixture of **10a** and **10b**): δ 8.11 – 7.98 (m, 3H), 7.61 (dd, J = 10.6, 2.8 Hz, 1H), 7.46 – 7.31 (m, 4H), 7.21 (s, 1H), 3.13 (qt, J = 6.1, 3.0 Hz, 1H), 2.89 (dddd, J = 12.0, 8.6, 6.9, 3.4 Hz, 2H), 2.00 (dqd, J = 8.9, 3.7, 1.7 Hz, 6H), 1.90 (ddd, J = 15.9, 8.2, 4.6 Hz, 6H), 1.78 (dtd, J = 12.7, 3.2, 1.5 Hz, 2H), 1.64 (tt, J = 12.4, 3.6 Hz, 4H), 1.57 – 1.51 (m, 4H), 1.45 (tt, J = 12.6, 3.2 Hz, 4H), 1.34 (tq, J = 12.6, 3.5 Hz, 4H).

^{13}C NMR (101 MHz, $CDCl_3$, for the mixture of **10a** and **10b**): δ 166.0, 144.9, 135.8, 135.7, 132.1, 132.0, 131.5, 131.4, 131.0, 128.9, 120.5, 119.5, 119.2, 118.8, 118.5, 116.6, 110.6, 110.4, 106.7, 106.5, 47.6, 47.5, 39.3, 33.6, 33.0, 32.9, 27.0, 26.7, 26.6, 26.3, 26.2.

^{19}F NMR (400 MHz, $CDCl_3$, for the mixture of **10a** and **10b**): δ -114.9, -115.1 ppm

4-Cyclohexyl-6-fluoroquinoline (10c):

TLC: Rf = 0.26 (9:1 hexane/EtOAc, UV).

GC (Ti= 80 ºC): Rt 7.704 min.

MS: *m/z* (%) 229 (M^+,100), 161 (55), 172 (37), 173 (32), 174 (30), 186 (28), 228 (20), 185 (20).

^{1}H NMR (400 MHz, $CDCl_3$): δ 8.79 (d, J = 4.6 Hz, 1H), 8.11 (dd, J = 9.2, 5.7 Hz, 1H), 7.67 (dd, J = 10.6, 2.8 Hz, 1H), 7.46 (ddd, J = 9.3, 8.0, 2.8 Hz, 1H), 7.29 (d, J = 4.6 Hz, 1H), 3.16 (tt, J = 11.2, 3.0 Hz, 1H), 1.99 (dtd, J = 6.5, 3.7, 1.6 Hz, 2H), 1.96 – 1.90 (m, 2H), 1.86 (dtt, J = 12.3, 3.1, 1.6 Hz, 1H), 1.58 – 1.46 (m, 4H), 1.39 – 1.30 (m, 1H).

HRMS (EI-TOF): *m/z* calcd for $C_{15}H_{16}FN$ 229,1267, found 229.1260.

^{13}C NMR (101 MHz, $CDCl_3$): δ 161.8, 153.2, 153.1, 149.74, 149.72, 145.5, 132.8, 132.7, 119.2, 119.0, 118.1, 106.9, 106.7, 39.3, 33.5, 26.9, 26.3.

^{19}F NMR (400 MHz, $CDCl_3$): δ -128.9 ppm.

Alkylation of ethyl nicotinate with Cyclohexane

Following the GP with ethyl isonicotinate (45 µL, 0.30 mmol) and cyclohexane (162 µL, 1.5 mmol, 5 equiv) in 46 h. After purification by FC, the product was obtained as a green oil (35 mg, 0.15 mmol, 50 %) using 1% to 5% EtOAc in *n*-hexane as the eluent. The spectroscopy data matched with previously reported in the literature.[14]

Synthesis of ethyl 2-cyclohexylisonicotinate (11):

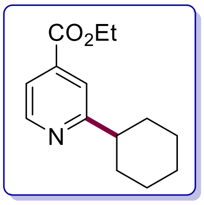

TLC: Rf = 0.30 (99:1 Hexane/EtOAc), UV).

GC (Ti= 80 ºC): 9.2 min

MS: *m/z* (%) 260 (M^+, 100), 247 (91), 315 (81), 286 (54), 232 (39), 261 (33), 314 (29), 316 (21).

^{1}H NMR (400 MHz, $CDCl_3$): δ 8.66 (dd, J = 5.1, 0.9 Hz, 1H), 7.71 (t, J = 1.3 Hz, 1H), 7.64 (dd, J = 5.1, 1.6 Hz, 1H), 4.40 (q, J = 7.1 Hz, 2H), 2.84 – 2.69 (m, 1H), 2.05 – 1.92 (m, 2H), 1.87 (dt, J = 13.0, 3.3 Hz, 2H), 1.81 – 1.69 (m, 1H), 1.64 – 1.50 (m, 2H), 1.48 – 1.42 (m, 2H), 1.39 (q, J = 7.1 Hz, 3H), 1.35 – 1.20 (m, 1H).

^{13}C NMR (101 MHz, $CDCl_3$): δ 167.6, 165.5, 149.7, 138.1, 120.3, 120.2, 61.6, 46.5, 32.8, 26.4, 25.9, 14.2.

Alkylation of 4-Phenylpyridine with Cyclohexane

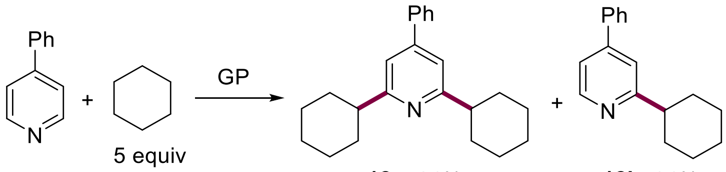

Following the GP with 4-phenylpyridine (47 mg, 0.30 mmol) and cyclohexane (162 µL, 1.5 mmol, 5 equiv) in 46 h, but using 3 equivalents of TFA (60 µL, 0.90 mmol, 3 equiv). After purification by FC, **12a** (20.1 mg, 0.06

mmol, 21%) and **LO401b** (22.1 mg, 0.09 mmol, 31%) were isolated as colorless oils using from 2% to 4% EtOAc in *n*-hexane as the eluent.

2,6-Dicyclohexyl-4-phenylpyridine (12a):

The spectroscopy data matched with previously reported in the literature.[11]

TLC: Rf = 0.67 (95:5 hexane/EtOAc, UV).

GC (Ti= 80 ºC): Rt 10.392 min.

MS: *m/z* (%) 319 (M^+, 60), 264 (100), 251 (51), 290 (35), 265 (31), 318 (24), 278 (17).

^{1}H NMR (400 MHz, $CDCl_3$): δ 7.66 – 7.59 (m, 2H), 7.52 – 7.36 (m, 3H), 7.17 (s, 2H), 2.76 (td, J = 11.5, 3.4 Hz, 2H), 2.00 (s, 4H), 1.86 (d, J = 11.9 Hz, 4H), 1.80 – 1.73 (m, 2H), 1.62 – 1.45 (m, 7H), 1.44 – 1.32 (m, 3H).

^{13}C NMR (101 MHz, $CDCl_3$): δ 167.1, 149.5, 148.9, 138.9, 129.1, 128.9, 127.2, 119.3, 119.2, 46.8, 33.1, 26.7, 26.2.

2-Cyclohexyl-4-phenylpyridine (12b):

The spectroscopy data matched with previously reported in the literature.[11]

TLC: Rf = 0.27 (95:5 hexane/EtOAc, UV).

GC (Ti= 80 ºC): Rt 8.409 min.

MS: *m/z* (%) 237 (M^+, 65), 182 (100), 208 (50), 169 (35), 183 (27), 236 (24), 193 (18).

^{1}H NMR (400 MHz, $CDCl_3$): δ 8.57 (dd, J = 5.2, 0.8 Hz, 1H), 7.66 – 7.59 (m, 2H), 7.51 – 7.40 (m, 3H), 7.38 – 7.34 (m, 1H), 7.34 – 7.28 (m, 1H), 2.77 (tt, J = 11.8, 3.4 Hz, 1H), 2.05 – 1.96 (m, 2H), 1.88 (dd, J = 12.7, 3.5 Hz, 2H), 1.79 – 1.73 (m, 1H), 1.67 – 1.54 (m, 2H), 1.52 – 1.37 (m, 3H).

^{13}C NMR (101 MHz, $CDCl_3$): δ 166.9, 149.2, 149.0, 138.7, 129.0, 128.8, 127.0, 119.2, 119.1, 46.6, 33.0, 26.6, 26.0.

Alkylation of Isonicotinonitrile with Cyclohexane

Following the GP with 4-cyanopyridine (31 mg, 0.30 mmol) and cyclohexane (324 µL, 3 mmol, 10 equiv) in 42 h. After purification by FC, the product was obtained as a colorless oil (36.9 mg, 0.14 mmol, 46 %) using 0% to 3% EtOAc in *n*-hexane as the eluent. The spectroscopy data matched with previously reported in the literature.[17]

2**,6-Dicyclohexylisonicotinonitrile (13)**:

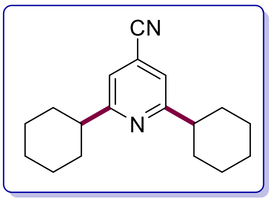

TLC: Rf = 0.62 (95:5 hexane/EtOAc, UV).

GC (Ti= 80 ºC): Rt 8.760 min.

MS: *m/z* (%) 268 (M^+, 96), 267 (51), 240 (17), 239 (63), 214 (27), 213 (100), 200 (75).

^{1}H NMR (300 MHz, $CDCl_3$): δ 7.16 (s, 2H), 2.71 (tt, J = 11.6, 3.4 Hz, 2H), 1.97 – 1.90 (m, 3H), 1.90 – 1.80 (m, 5H), 1.79 –1.72 (m, 2H), 1.56 – 1.47 (m, 2H), 1.46 – 1.40 (m, 5H), 1.39 – 1.30 (m, 3H).

^{13}C NMR (101 MHz, $CDCl_3$): δ 167.3, 120.7, 119.7, 117.7, 46.5, 32.8, 26.5, 26.1.

Alkylation of 1-methylquinoxalin-2(1H)-one with Cyclohexane

Following the GP with 1-methylquinoxalin-2(1H)-one (48 mg, 0.30 mmol) and cyclohexane (0.7 mL, 6.9 mmol, 23 equiv) in 46 h. After purification by FC, the product was obtained as a white solid (50.8 mg, 0.21 mmol, 70%) using from 5% to 10% EtOAc in *n*-hexane as the eluent. The spectroscopy data matched with previously reported in the literature.[19]

3-Cyclohexyl-1-methylquinoxalin-2(1H)-one (14):

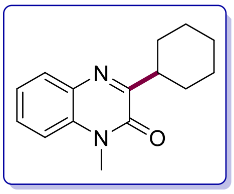

TLC: Rf = 0.24 (9:1 hexane/EtOAc, UV).

GC (Ti= 80 ºC): Rt 8.838 min.

MS: *m/z* (%) 242 (M^+, 46), 174 (100), 187 (41), 227 (24), 175 (15).

^{1}H NMR (400 MHz, $CDCl_3$): δ 7.82 (ddd, *J* = 8.0, 1.6, 0.5 Hz, 1H), 7.49 (ddd, *J* = 8.3, 7.3, 1.6 Hz, 1H), 7.34 – 7.24 (m, 2H), 3.68 (s, 3H), 3.33 (tt, *J* = 11.4, 3.2 Hz, 1H), 1.95 (d, *J* = 12.2 Hz, 2H), 1.85 (dd, *J* = 12.1, 3.0 Hz, 2H), 1.80 – 1.70 (m, 1H), 1.64 – 1.46 (m, 3H), 1.42 (dt, *J* = 12.0, 2.9 Hz, 1H), 1.34 – 1.22 (m, 1H).

^{13}C NMR (101 MHz, $CDCl_3$): δ 164.4, 154.6, 133.0, 132.9, 129.8, 129.4, 123.4, 113.5, 40.9, 30.6, 29.1, 26.4, 26.2.

Alkylation of Quinoxalin-2(1H)-one with Cyclohexane

Following the GP, quinoxalin-2(1H)-one (44 mg, 0.30 mmol) and cyclohexane (0.7 mL, 6.9 mmol, 23 equiv) in 46 h. After purification by FC, the product was obtained as a white solid (46.5 mg, 0.20 mmol, 68%) using from 0% to 5% EtOAc in *n*-hexane as the eluent. The spectroscopy data matched with previously reported in the literature.[18]

3-Cyclohexylquinoxalin-2(1H)-one (15):

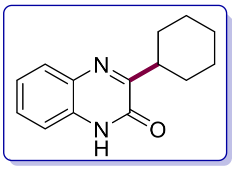

TLC: Rf = 0.24 (9:1 hexane/EtOAc, UV).

^{1}H NMR (500 MHz, DMSO-d_6): δ 12.30 (s, 1H), 7.69 (dd, *J* = 8.0, 1.4 Hz, 1H), 7.45 (ddd, *J* = 8.4, 7.1, 1.5 Hz, 1H), 7.25 (td, *J* = 7.9, 7.2, 1.3 Hz, 2H), 3.16 (tt, *J* = 11.5, 3.2 Hz, 1H), 1.89 – 1.82 (m, 2H), 1.79 (dt, *J* = 12.9, 3.3 Hz, 2H), 1.70 (dtd, *J* = 14.4, 3.4, 1.7 Hz, 1H), 1.44 (qd, *J* = 12.7, 2.9 Hz, 2H), 1.34 (tt, *J* = 12.8, 3.2 Hz, 2H), 1.23 (tq, *J* = 12.7, 5.2, 4.4 Hz, 1H).

^{13}C NMR (126 MHz, DMSO-d_6): δ 164.8, 154.2, 131.7, 131.5, 129.4, 128.2, 123.0, 115.1, 39.4, 30.1, 25.9, 25.8.

Alkylation of 1-Methyl-6-nitroquinoxalin-2(1H)-one with Cyclohexane

Following the GP with 1-methyl-6-nitroquinoxalin-2(1H)-one (62 mg, 0.30 mmol) and cyclohexane (0.7 mL, 6.9 mmol, 23 equiv) in 46 h. After purification by FC, the product was obtained as a white solid (37.8 mg, 0.13 mmol, 44%) using from 10% to 30% EtOAc in *n*-hexane as the eluent. The spectroscopy data matched with previously reported in the literature.[20]

3-Cyclohexyl-1-methyl-6-nitroquinoxalin-2(1H)-one (16):

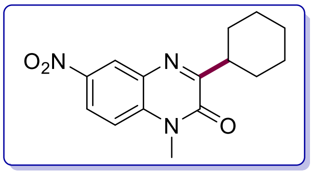

TLC: Rf = 0.15 (9:1 hexane/EtOAc, UV).

^{1}H NMR (400 MHz, $CDCl_3$): *δ* 8.70 (d, *J* = 2.6 Hz, 1H), 8.35 (dd, *J* = 9.2, 2.6 Hz, 1H), 7.37 (d, *J* = 9.2 Hz, 1H), 3.73 (d, *J* = 1.2 Hz, 3H), 3.32 (tt, *J* = 11.4, 3.3 Hz, 1H), 1.98 – 1.91 (m, 2H), 1.87 (dt, *J* = 12.7, 3.1 Hz, 2H), 1.77 (dtt, *J* = 12.7, 3.3, 1.5 Hz, 1H), 1.60 – 1.50 (m, 2H), 1.44 (tt, *J* = 12.6, 3.0 Hz, 2H), 1.32 (tt, *J* = 12.4, 3.5 Hz, 1H).

^{13}C NMR (101 MHz, $CDCl_3$): *δ* 166.9, 154.1, 143.2, 137.6, 132.0, 125.5, 123.9, 114.0, 40.9, 30.4, 29.6, 26.1, 26.0.

Alkylation of Quinoxaline with Cyclohexane

Following the GP with quinoxaline (39 mg, 0.30 mmol) and cyclohexane (162 µL, 1.50 mmol, 5 equiv) in 42 h. After purification by FC, the product was obtained as a colorless oil (39.5 mg, 0.19 mmol, 62%) using 0% to 5% EtOAc in *n*-hexane as the eluent. The spectroscopy data matched with previously reported in the literature.[19]

2-Cyclohexylquinoxaline (17):

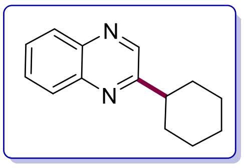

TLC: Rf = 0.38 (95:5 hexane/EtOAc, UV).

GC (Ti= 80 ºC): Rt 7.601 min.

MS: *m/z* (%) 212 (M^+, 75), 183 (19), 158 (21), 145 (15).

^{1}H NMR (300 MHz, $CDCl_3$): *δ* 8.78 (s, 1H), 8.07 (dt, *J* = 7.6, 2.8 Hz, 2H), 7.78 – 7.65 (m, 2H), 2.98 (tt, *J* = 11.9, 3.4 Hz, 1H), 2.09 – 1.88 (m, 4H), 1.75 – 1.63 (m, 2H), 1.56 – 1.31 (m, 4H).

^{13}C NMR (101 MHz, $CDCl_3$): *δ* 161.2, 145.0, 142.0, 141.4, 130.1, 129.2 (2C), 128.9, 45.1, 32.5, 26.5, 26.0.

Alkylation of pyrazine-2,3-dicarbonitrile with Cyclohexane

Following the GP with pyrazine-2,3-dicarbonitrile (40 mg, 0.30 mmol) and cyclohexane (162 µL, 1.5 mmol, 5 equiv) in 46 h. After purification by FC, the product was obtained as a green oil (41 mg, 0.19 mmol, 64%) using 1% to 10% EtOAc in *n*-hexane as the eluent. The spectroscopy data matched with previously reported in the literature.[14]

5-Cyclohexylpyrazine-2,3-dicarbonitrile (18):

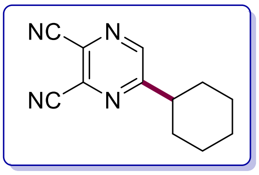

TLC: Rf = 0.18 (95:5 Hexane/EtOAc), UV).

GC (Ti= 80 ºC): 7.5 min

MS: *m/z* (%) 144 (M^+, 100), 157 (56), 212 (54), 145 (25).

^{1}H NMR (400 MHz, $CDCl_3$): δ 8.74 (s, 1H), 2.92 (tt, *J* = 11.9, 3.3 Hz, 1H), 2.02 – 1.85 (m, 4H), 1.79 (s, 1H), 1.61 (d, *J* = 3.5 Hz, 2H), 1.51 – 1.40 (m, 2H), 1.38 – 1.22 (m, 1H).

^{13}C NMR (101 MHz, $CDCl_3$): δ 165.7, 146.3, 133.1, 130.9, 126.2, 113.0, 44.3, 31.8, 25.8, 25.3.

Alkylation of Phenanthridine with Cyclohexane

Following the GP (but adding 20 mol% of pyridine *N*-oxide at the beginning of the reaction and an extra 10 mol% after 24 h) with phenanthridine (54 mg, 0.30 mmol) and cyclohexane (162 µL, 1.5 mmol, 5 equiv) in 46 h. After purification by FC, the product was obtained as a colorless oil (64.2 mg, 0.25 mmol, 82%) using 0% to 5% EtOAc in *n*-hexane as the eluent. The spectroscopy data matched with previously reported in the literature.[13]

6-Cyclohexylphenanthridine (19):

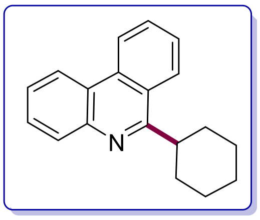

TLC: Rf = 0.72 (97:3 hexane/EtOAc, UV).

GC (Ti= 80 ºC): Rt 9.768 min.

MS: *m/z* (%) 261 (M^+,44), 206 (100), 260 (68), 193 (44), 207 (33), 217 (17), 104 (16).

^{1}H NMR (400 MHz, $CDCl_3$) δ 8.65 (dd, *J* = 8.4, 1.2 Hz, 1H), 8.53 (dd, *J* = 8.2, 1.4 Hz, 1H), 8.32 (dd, *J* = 8.3, 1.3 Hz, 1H), 8.17 (d, *J* = 8.1 Hz, 1H), 7.81 (ddd, *J* = 8.3, 7.0, 1.3 Hz, 1H), 7.70 (dddd, *J* = 10.4, 8.3, 7.0, 1.3 Hz, 2H), 7.61 (ddd, *J* = 8.3, 7.0, 1.4 Hz, 1H), 3.63 (tt, *J* = 11.2, 3.4 Hz, 1H), 2.17 – 2.06 (m, 2H), 2.03 – 1.94 (m, 4H), 1.86 (ddtd, *J* = 12.8, 4.6, 3.1, 1.5 Hz, 1H), 1.66 – 1.52 (m, 2H), 1.46 (qt, *J* = 12.9, 3.3 Hz, 1H).

^{13}C NMR (101 MHz, $CDCl_3$) δ 165.4, 143.9, 133.1, 130.0, 128.5, 127.1, 126.2, 125.7, 124.8, 123.4, 122.6, 121.9, 42.1, 32.4, 27.0, 26.4.

Alkylation of Benzo[*d*]thiazole with Cyclohexane

Following the GP with benzo[d]thiazole (33 µL, 0.30 mmol) and cyclohexane (162 µL, 1.50 mmol, 5 equiv) in 42 h. After purification by FC, the product was obtained as a colorless oil (36.5 mg, 0.17 mmol, 56 %) using 0% to 5% EtOAc in *n*-hexane as the eluent. The spectroscopy data matched with previously reported in the literature.[21]

2-Cyclohexylbenzo[*d*]thiazole (20):

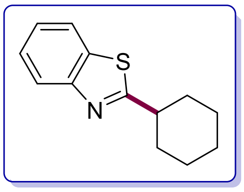

TLC: Rf = 0.42 (95:5 hexane/EtOAc, UV).

GC (Ti= 80 ºC): Rt 7.605 min.

MS: *m/z* (%) 217 (M^+, 15), 188 (17), 163 (19) 149 (90).

^{1}H NMR (300 MHz, $CDCl_3$): *δ* 7.98 (dt, *J* = 8.2, 0.9 Hz, 1H),7.85 (ddd, *J* = 7.9, 1.3, 0.6 Hz, 1H), 7.44 (ddd, *J* = 8.3, 7.2, 1.3 Hz, 1H), 7.33 (ddd, *J* = 8.3, 7.2, 1.2 Hz, 1H), 3.11 (tt, *J* = 11.6, 3.6 Hz, 1H), 2.26 – 2.16 (m, 2H), 1.93 – 1.83 (m, 2H), 1.82 – 1.73 (m, 1H), 1.71 – 1.56 (m, 2H), 1.53 – 1.36 (m, 2H), 1.35 – 1.27 (m, 1H).

^{13}C NMR (101 MHz, $CDCl_3$): *δ* 177.8, 153.2, 134.6, 125.9, 124.6, 122.7, 121.7, 43.6, 33.6, 26.2, 25.9.

Alkylation of 1-methyl-1H-benzo[*d*]imidazole with Cyclohexane

Following the GP (but adding 20 mol% of pyridine *N*-oxide at the beginning of the reaction and an extra 10 mol% after 24 h) with 1-methyl-1H-benzo[*d*]imidazole (40 mg, 0.30 mmol) and cyclohexane (324 µL, 3.0 mmol, 10 equiv) in 46 h. After purification by FC, the product was obtained as a yellow solid (38.5 mg, 0.18

mmol, 60%) using from 10% to 15% EtOAc in *n*-hexane as the eluent. The spectroscopy data matched with previously reported in the literature.[13]

2-Cyclohexyl-1-methyl-1H-benzo[d]imidazole (21):

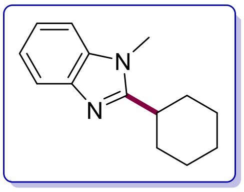

TLC: Rf = 0.20 (9:1 hexane/EtOAc, UV).

GC (Ti= 80 ºC): Rt 8.182 min.

MS: *m/z* (%) 214 (M^+, 27), 159 (100), 146 (32), 160 (23), 185 (16).

^{1}H NMR (400 MHz, $CDCl_3$): δ 7.81 – 7.74 (m, 1H), 7.34 – 7.23 (m, 3H), 3.77 (s, 3H), 2.88 (tt, J = 11.7, 3.5 Hz, 1H), 2.08 – 1.79 (m, 7H), 1.53 – 1.36 (m, 3H).

^{13}C NMR (101 MHz, $CDCl_3$): δ 159.1, 142.6, 135.7, 122.0, 121.8, 119.4, 108.9, 36.4, 31.6, 29.6, 26.4, 25.9.

Alkylation of Lepidine with Cyclopentane

Following the GP (but adding 20 mol% of pyridine *N*-oxide at the beginning of the reaction and an extra 10 mol% after 24 h) with lepidine (43 µL, 0.30 mmol) and cyclopentane (141 µL, 1.5 mmol, 5 equiv) in 46 h. After purification by FC, the product was obtained as a colorless oil (56.9 mg, 0.27 mmol, 90%) using 0% to 5% EtOAc in *n*-hexane as the eluent. The spectroscopy data matched with previously reported in the literature.[21]

2-Cyclopentyl-4-methylquinoline (22):

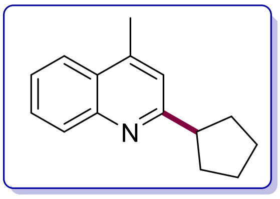

TLC: Rf = 0.54 (95:5 hexane/EtOAc, UV).

GC (Ti= 80 ºC): Rt 7.551 min.

MS: *m/z* (%) 211 (M^+,46), 170 (100), 182 (51), 183 (51), 171 (32), 168 (32), 210 (28), 167 (24).

^{1}H NMR (400 MHz, $CDCl_3$): δ 8.09 (dt, J = 8.4, 0.9 Hz, 1H), 7.93 (ddd, J = 8.3, 1.6, 0.6 Hz, 1H), 7.66 (ddd, J = 8.4, 6.9, 1.4 Hz, 1H), 7.49 (ddd, J = 8.2, 6.9, 1.3 Hz, 1H), 7.18 (d, J = 1.0 Hz, 1H), 3.45 – 3.33 (m, 1H), 2.68 (d, J = 1.0 Hz, 3H), 2.23 – 2.11 (m, 2H), 1.94 – 1.82 (m, 4H), 1.80 – 1.70 (m, 2H).

^{13}C NMR (101 MHz, $CDCl_3$) δ 165.9, 147.2, 144.7, 129.3, 129.2, 127.1, 125.6, 123.6, 120.7, 48.6, 33.7, 26.2, 18.9.

Alkylation of Lepidine with Cyclododecane

Following the GP (but adding 20 mol% of pyridine *N*-oxide at the beginning of the reaction and an extra 10 mol% after 24 h) with lepidine (43 µL, 0.30 mmol) and cyclododecane (151.2 mg, 0.9 mmol, 3 equiv) in 46 h. After purification by FC, the product was obtained as a white solid (69.6 mg, 0.22 mmol, 75%) using from 0% to 5% EtOAc in *n*-hexane as the eluent. The spectroscopy data matched with previously reported in the literature.[13]

2-Cyclododecyl-4-methylquinoline (23):

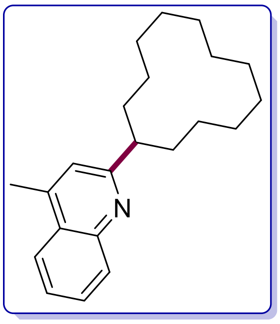

TLC: Rf = 0.68 (95:5 hexane/EtOAc, UV).

GC (Ti= 80 ºC): Rt 10.490 min.

MS: *m/z* (%) 309 (M^+,30), 170 (100), 157 (30), 171 (20), 196 (16), 226 (12).

^{1}H NMR (400 MHz, $CDCl_3$): δ 8.16 – 8.08 (m, 1H), 7.94 (dd, *J* = 8.3, 1.4 Hz, 1H), 7.67 (ddd, *J* = 8.4, 6.8, 1.5 Hz, 1H), 7.49 (ddd, *J* = 8.2, 6.8, 1.3 Hz, 1H), 7.14 (d, *J* = 1.1 Hz, 1H), 3.14 (p, *J* = 6.6 Hz, 1H), 2.69 (d, *J* = 0.9 Hz, 3H), 1.92 (dtd, *J* = 14.2, 7.2, 5.1 Hz, 2H), 1.73 (ddt, *J* = 10.6, 7.7, 5.4 Hz, 2H), 1.64 – 1.33 (m, 18H).

^{13}C NMR (101 MHz, $CDCl_3$): δ 166.6, 147.3, 144.2, 129.4, 128.9, 126.9, 125.4, 123.5, 121.4, 42.9, 30.2, 23.96, 23.90, 23.7, 23.4, 22.9, 18.8.

Alkylation of Lepidine with Methylcyclopentane

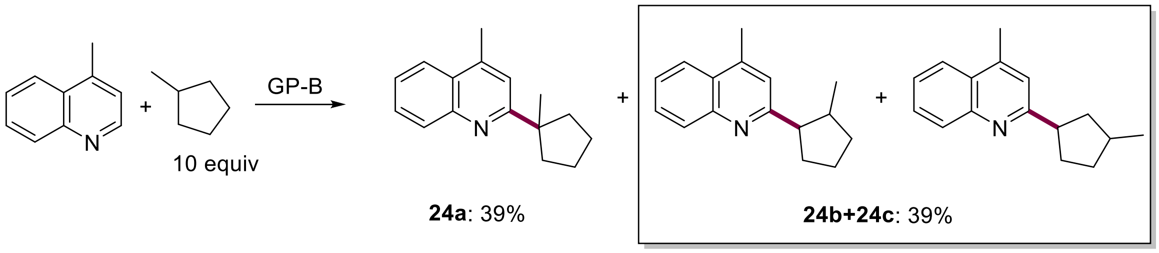

Following the GP (but adding 20 mol% of pyridine *N*-oxide at the beginning of the reaction and an extra 10 mol% after 24 h) with lepidine (43 µL, 0.30 mmol) and methylcyclopentane (340 µL, 3 mmol, 10 equiv) in 46 h. After purification by FC, **24a** (26.3 mg, 0.11 mmol, 39%) and **24b**+**24c** (26.3 mg, 0.11 mmol, 39%) were isolated as colorless oils using from 0% to 5% EtOAc in *n*-hexane as the eluent. The spectroscopy data matched with previously reported in the literature.[13]

4-Methyl-2-(1-methylcyclopentyl)quinoline (24a):

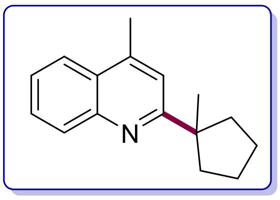

TLC Rf = 0.76 (97:3 hexane/EtOAc, UV).

GC (Ti= 80 ºC): Rt 7.568 min.

MS: *m/z* (%) 225 (M^+,30), 184 (100), 210 (64), 207 (27), 196 (27), 182 (21), 185 (18).

^{1}H NMR (400 MHz, $CDCl_3$): δ 8.13 (d, *J* = 20.6 Hz, 1H), 7.94 (dd, *J* = 8.3, 1.5 Hz, 1H), 7.67 (ddd, *J* = 8.4, 6.8, 1.4 Hz, 1H), 7.50 (ddd, *J* = 8.2, 6.8, 1.3 Hz, 1H), 7.31 (d, *J* = 1.2 Hz, 1H), 2.70 (d, *J* = 1.0 Hz, 3H), 2.37-2.29 (m, 2H), 1.90 – 1.71 (m, 6H), 1.44 (s, 3H).

^{13}C NMR (101 MHz, $CDCl_3$): δ 168.9, 129.6, 129.1, 126.7, 125.6, 123.5, 120.2, 50.1, 39.2, 28.4, 24.6, 19.1.

4-Methyl-2-(2-methylcyclopentyl)quinoline + 4-Methyl-2-(3-methylcyclopentyl)quinoline (24b+24c):

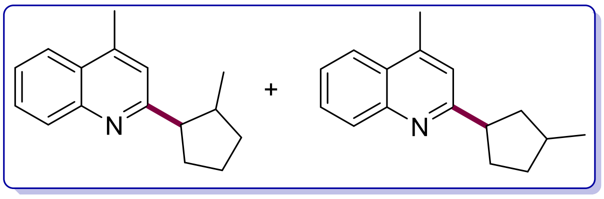

TLC Rf = 0.70 (97:3 hexane/EtOAc, UV).

GC 24b (Ti= 80 ºC): Rt 7.584 min.

MS 24b: *m/z* (%) 225 (M^+,30), 184 (100), 210 (64), 207 (27), 196 (27), 182 (21), 185 (19).

GC 24c (Ti= 80 ºC): Rt 7.568 min.

MS 24c: *m/z* (%) 225 (M^+, 30), 184 (100), 210 (64), 207 (27), 196 (27), 182 (21), 185 (19).

^{1}H NMR (400 MHz, $CDCl_3$, for the mixture of **24b** and **24c**): δ 8.19 – 8.09 (m, 3H), 7.96 (ddt, J = 8.2, 3.5, 1.2 Hz, 3H), 7.69 (ddt, J = 8.5, 6.9, 1.5 Hz, 3H), 7.57 – 7.48 (m, 3H), 7.22 (dd, J = 5.9, 1.0 Hz, 1.75H), 7.19 (d, J = 1.0 Hz, 1.25H), 3.58 (p, J = 8.2 Hz, 0.92H), 3.50 (ddd, J = 9.1, 7.4, 2.3 Hz, 0.75H), 2.91 (q, J = 9.7 Hz, 1.33H), 2.71 (d, J = 1.0 Hz, 6.34H), 2.71 (d, J = 1.0 Hz, 2.66H), 2.37 – 1.75 (m, 18H), 1.55 – 1.34 (m, 3H), 1.14 (d, J = 6.5 Hz, 2.76H), 1.11 (d, J = 6.8 Hz, 2.25H), 1.03 (d, J = 6.6 Hz, 3.99H).

^{13}C NMR (101 MHz, $CDCl_3$, for the mixture of **24b** and **24c**): δ 166.3, 165.8, 165.1, 147.1, 146.9, 145.1, 145.0, 129.4, 129.4, 129.3, 129.2, 129.2, 129.1, 127.2, 127.1, 127.0, 125.8, 125.7, 123.71, 123.69, 120.9, 120.7, 120.6, 56.7, 48.8, 47.2, 43.2, 42.5, 41.4, 35.6, 35.4, 35.2, 34.45, 34.43, 34.3, 34.2, 32.6, 29.8, 24.5, 21.3, 20.7, 19.0, 18.96.

Alkylation of Lepidine with Bicyclo[2.2.1]heptane

Following the GP (but adding 20 mol% of pyridine *N*-oxide at the beginning of the reaction and an extra 10 mol% after 24 h) with lepidine (43 µL, 0.30 mmol) and bicyclo[2.2.1]heptane (145 mg, 1.5 mmol, 5 equiv) in 46 h. After purification by FC, the product was obtained as a white solid (53.1 mg, 0.22 mmol, 74%) using from 0% to 2% EtOAc in *n*-hexane as the eluent. The spectroscopy data matched with previously reported in the literature.[13]

2-(Bicyclo[2.2.1]heptan-2-yl)-4-methylquinoline (25):

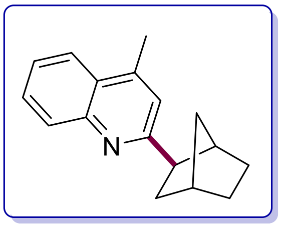

TLC: Rf = 0.81 (95:5 hexane/EtOAc, UV).

GC (Ti= 80 ºC): Rt 8.384 min.

MS: *m/z* (%) 237 (M^+,12), 170 (100), 208 (53), 171 (19), 209 (13).

^{1}H NMR (400 MHz, $CDCl_3$): δ 8.10 – 8.03 (m, 1H), 7.96 – 7.90 (m, 1H), 7.66 (ddd, J = 8.4, 6.8, 1.5 Hz, 1H), 7.48 (ddd, J = 8.2, 6.8, 1.3 Hz, 1H), 7.18 (d, J = 1.1 Hz, 1H), 3.03 (dd, J = 8.9, 5.6 Hz, 1H), 2.67 (d, J = 1.0 Hz, 3H), 2.57 (t, J = 1.8 Hz, 1H), 2.42 (s, 1H), 2.24 (s, 1H), 1.79 – 1.60 (m, 4H), 1.53 – 1.44 (m, 1H), 1.39 – 1.31 (m, 1H), 1.19 (ddd, J = 9.7, 2.5, 1.4 Hz, 1H).

^{13}C NMR (101 MHz, $CDCl_3$): δ 165.4, 147.2, 144.0, 129.5, 128.8, 126.7, 125.3, 123.4, 121.5, 49.8, 43.0, 36.7, 36.3, 36.0, 30.5, 29.1, 18.7.

Alkylation of Lepidine with Adamantane

Following the GP (but adding 20 mol% of pyridine *N*-oxide at the beginning of the reaction and an extra 10 mol% after 24 h) with lepidine (43 µL, 0.30 mmol) and pentane (198 mg, 1.5 mmol, 5 equiv) in 46 h. After purification by FC, the product was obtained as a colorless oil (70.6 mg, 0.25 mmol, 85% (C1:C2 91:9)) using from 0% to 5% EtOAc in *n*-hexane as the eluent. The spectroscopy data matched with previously reported in the literature.[14]

2-(Adamantan-1-yl)-4-methylquinoline (26):

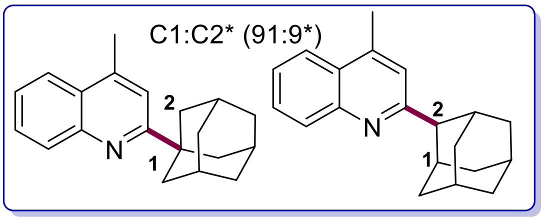

TLC: Rf = 0.74 (97:3 hexane/EtOAc, UV).

GC(C1) (Ti= 80 ºC): Rt 9.785 min.

MS(C1): *m/z* (%) 277 (M^+,100), 220 (39), 278 (30), 276 (23), 222 (18), 234 (18).

GC(C2) (Ti= 80 ºC): Rt 9.789 min.

MS(C2): *m/z* (%) 277 (M^+, 100), 157 (35), 276 (35), 234 (23), 278 (22), 262 (20).

^{1}H NMR (500 MHz, $CDCl_3$): δ 8.10 (d, *J* = 9.4 Hz, 1H), 7.94 (dd, *J* = 8.2, 1.4 Hz, 1H), 7.67 (ddd, *J* = 8.4, 6.8, 1.5 Hz, 1H), 7.49 (ddd, *J* = 8.2, 6.7, 1.3 Hz, 1H), 7.34 (s, 1H), 2.70 (s, 3H), 2.23 – 2.07 (m, 10H), 1.84 (t, *J* = 3.1 Hz, 6H).

^{13}C NMR (126 MHz, $CDCl_3$): δ 168.6, 164.1, 147.4, 143.5, 129.9, 128.7, 126.7, 126.5, 125.4, 123.4, 120.5, 118.5, 50.4, 41.8, 39.6, 39.2, 37.9, 36.9, 32.7, 30.9, 29.7, 28.9, 28.1, 27.9, 19.0, 18.9.

Alkylation of Phenanthridine with 2,3-Dimethylbutane

Following the GP (but adding 20 mol% of pyridine *N*-oxide at the beginning of the reaction and an extra 10 mol% after 24 h) with lepidine (43 µL, 0.30 mmol) and 2,3-dimethylbutane (193 µL, 1.5 mmol, 5 equiv) in 46 h. After purification by FC, the product was obtained as a colorless oil (37.4 mg, 0.16 mmol, 55%) using 0% to 2% EtOAc in *n*-hexane as the eluent. The spectroscopy data matched with previously reported in the literature.[22]

2-(2,3-Dimethylbutan-2-yl)-4-methylquinoline (27):

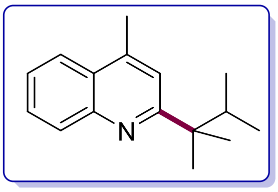

TLC: Rf = 0.81 (95:5 hexane/EtOAc, UV).

GC (Ti= 80 ºC): Rt 7.122 min.

MS: *m/z* (%) 227 (M^+,6), 185 (100), 184 (77), 212 (61), 157 (19), 170 (19).

^{1}H NMR (400 MHz, $CDCl_3$): δ 8.12 – 8.04 (m, 1H), 7.94 (dd, *J* = 8.3, 1.4 Hz, 1H), 7.66 (ddd, *J* = 8.4, 6.8, 1.5 Hz, 1H), 7.49 (ddd, *J* = 8.2, 6.8, 1.3 Hz, 1H), 7.31 (d, *J* = 1.2 Hz, 1H), 2.69 (d, *J* = 1.1 Hz, 3H), 2.35-2.25 (m, 1H), 1.38 (s, 6H), 0.82 (d, *J* = 6.8 Hz, 6H).

^{13}C NMR (101 MHz, $CDCl_3$): δ 168.8, 126.4, 125.3, 123.3, 119.6, 43.8, 37.1, 23.6, 19.0, 17.9.

Alkylation of Lepidine with 2-Methylbutane

Following the GP (but adding 20 mol% of pyridine *N*-oxide at the beginning of the reaction and an extra 10 mol% after 24 h) with lepidine (43 µL, 0.30 mmol) and isopentane (175 µL, 1.5 mmol, 5 equiv) in 46 h. After purification by FC, the product was obtained as a colorless oil (51.7 mg, 0.24 mmol, 81%) using 0% to 5% EtOAc in *n*-hexane as the eluent. The spectroscopy data matched with previously reported in the literature.[23]

4-Methyl-2-(*tert*-pentyl)quinoline (28):

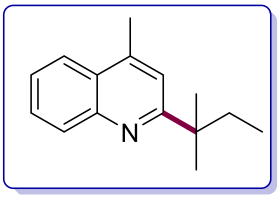

TLC: Rf = 0.72 (97:3 hexane/EtOAc, UV).

GC (Ti= 80 ºC): Rt 6.657 min.

MS: *m/z* (%) 213 (M^+,4), 185 (10), 198 (88), 184 (62), 170 (30), 157 (27), 186 (20).

^{1}H NMR (400 MHz, $CDCl_3$): δ 8.02 (s, 1H), 7.85 (ddd, *J* = 8.3, 1.5, 0.6 Hz, 1H), 7.57 (ddd, *J* = 8.4, 6.8, 1.4 Hz, 1H), 7.41 (ddd, *J* = 8.2, 6.9, 1.3 Hz, 1H), 7.22 (d, *J* = 1.2 Hz, 1H), 2.60 (d, *J* = 1.0 Hz, 3H), 1.77 (q, *J* = 7.5 Hz, 2H), 1.35 (s, 6H), 0.66 (t, *J* = 7.5 Hz, 3H).

^{13}C NMR (101 MHz, $CDCl_3$): δ 168.1, 147.3, 143.6, 129.9, 128.9, 126.6, 125.6, 123.5, 119.5, 41.3, 35.9, 27.5, 19.1, 9.4.

Alkylation of Lepidine with Pentane

Following the GP-C with lepidine (43 µL, 0.30 mmol) and pentane (800 µL, 77 mmol, 23 equiv) in 46 h. After purification by FC, the product was obtained as a colorless oil (56.2 mg, 0.26 mmol, 88% (C2:C3 = 69:31) using 0% to 5% EtOAc in *n*-hexane as the eluent. The spectroscopy data matched with previously reported in the literature.[13]

4-Methyl-2-(pentan-2-yl)quinoline and 4-methyl-2-(pentan-3-yl)quinoline (29):

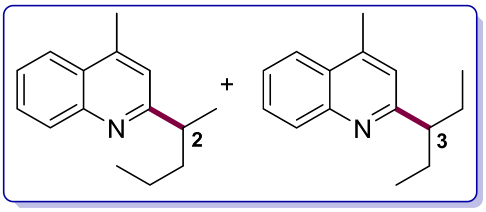

TLC: Rf = 0.53 (95:5 hexane/EtOAc, UV).

GC(C2) (Ti= 80 ºC): Rt 6.813 min.

MS(C2): *m/z* (%) 213 (M^+, 2), 171 (100), 170 (99), 184 (62), 172 (32), 198 (27), 115 (25).

GC(C3) (Ti= 80 ºC): Rt 6.694 min.

MS(C3): *m/z* (%) 213 (M^+, 3), 170 (100), 184 (75), 185 (75), 198 (29), 157 (25), 171 (23).

^{1}H NMR (400 MHz, $CDCl_3$) δ 8.15 – 8.07 (m, 3H), 7.95 (ddd, *J* = 8.5, 3.5, 1.4 Hz, 3H), 7.67 (ddt, *J* = 8.4, 6.9, 1.5 Hz, 3H), 7.50 (dddd, *J* = 8.2, 6.9, 2.4, 1.3 Hz, 3H), 7.14 (d, *J* = 1.2 Hz, 2H), 7.11 (d, *J* = 1.1 Hz, 1H), 3.15 – 3.04 (m, 2H), 2.78 (ddd, *J* = 8.2, 6.4, 1.7 Hz, 1H), 2.69 (t, *J* = 1.1 Hz, 10H), 1.83 – 1.78 (m, 4H), 1.65 (dd, *J* = 23.6, 1.4 Hz, 3H), 1.36 (d, *J* = 7.0 Hz, 10H), 1.26 (s, 4H), 0.90 (t, *J* = 7.3 Hz, 8H), 0.84 (t, *J* = 7.4 Hz, 6H).

^{13}C NMR (101 MHz, $CDCl_3$) δ 166.8, 165.6, 144.6, 144.4, 129.34, 129.30, 129.1, 129.0, 127.1, 127.0, 125.5, 123.6, 120.7, 120.2, 52.0, 42.5, 39.3, 28.2, 20.8, 20.7, 18.8, 14.2, 12.2.

Alkylation of Lepidine with Mesitylene

Following the GP (but adding 20 mol% of pyridine *N*-oxide at the beginning of the reaction and an extra 10 mol% after 24 h) with lepidine (43 µL, 0.30 mmol) and mesitylene (125 µL, 0.9 mmol, 3 equiv) in 46 h. After purification by FC, the product was obtained as a colorless oil (26 mg, 0.1 mmol, 34%) using 1% to 10% EtOAc in *n*-hexane as the eluent. The spectroscopy data matched with previously reported in the literature.[21]

2-(3,5-Dimethylbenzyl)-4-methylquinoline (30):

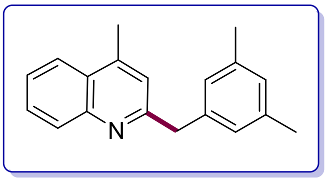

TLC: Rf = 0.3 (9:1 hexane/EtOAc, UV).

GC (Ti= 80 ºC):Rt: 9.0 min

MS: *m/z* (%) 260 (M^+, 100), 261 (74), 246 (69), 231 (31), 245 (25), 207 (24).

^{1}H NMR (400 MHz, $CDCl_3$): δ 8.22 (d, *J* = 8.3 Hz, 1H), 7.96 (d, *J* = 10.3 Hz, 1H), 7.78 – 7.69 (m, 1H), 7.59 – 7.51 (m, 1H), 7.10 (s, 1H), 6.95 (d, *J* = 0.8 Hz, 2H), 6.88 (d, *J* = 3.2 Hz, 1H), 4.28 (s, 2H), 2.64 (s, 3H), 2.28 (s, 6H).

^{13}C NMR (101 MHz, $CDCl_3$): δ 160.7,146.4, 145.9, 138.6, 138.2, 129.7, 128.7, 128.3, 127.1, 126.9, 126.1, 123.7, 122.2, 44.6, 21.2, 18.8.

Alkylation of Lepidine with *p*-cymene

Following the GP (but adding 20 mol% of pyridine *N*-oxide at the beginning of the reaction and an extra 10 mol% after 24 h) with lepidine (43 µL, 0.30 mmol) and *p*-cymene (234 µL, 1.5 mmol, 5 equiv) in 46 h. After purification by FC, the product was obtained as a yellow oil (25 mg, 0.10 mmol, 30 %) using 1% to 10% EtOAc in *n*-hexane as the eluent. The product was unreported in the literature.

2-(4-Isopropylbenzyl)-4-methylquinoline (31):

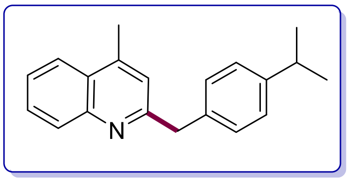

TLC: Rf = 0.40 (97:3 Hexane/EtOAc), UV).

GC (Ti= 80 ºC): 9.3 min

MS: *m/z* (%) 274 (M^+, 100), 275 (80), 260 (91), 218 (31), 231 (27), 261 (21).

HRMS (EI-TOF): *m/z* calcd for $C_{20}H_{21}N$ 275.1672, found 275.1672.

^{1}H NMR (400 MHz, $CDCl_3$): *δ* 8.14 (d, J = 8.4 Hz, 1H), 7.94 (ddd, *J* = 8.4, 1.5, 0.6 Hz, 1H), 7.70 (ddd, *J* = 8.4, 6.9, 1.4 Hz, 1H), 7.56 – 7.50 (m, 1H), 7.28 – 7.24 (m, 2H), 7.17 (d, *J* = 8.1 Hz, 2H), 7.10 (t, *J* = 1.0 Hz, 1H), 4.29 (s, 2H), 2.93 – 2.82 (m, 1H), 2.63 (d, *J* = 2.8, 1.0 Hz, 3H), 1.24 (d, *J* = 6.9 Hz, 6H).

^{13}C NMR (101 MHz, $CDCl_3$): *δ* 161.1 ,147.3, 147.1, 145.1, 136.5, 129.4, 129.3, 129.25, 129.20, 127.1, 127.0, 126.8, 125.9, 123.7, 122.3, 33.8, 24.1, 18.8.

Alkylation of Lepidine with Pentanenitrile

Following the GP (but adding 20 mol% of pyridine *N*-oxide at the beginning of the reaction and an extra 10 mol% after 24 h) with lepidine (43 µL, 0.30 mmol) and pentanitrile (311 µL, 3 mmol, 10 equiv) in 46 h. After purification by FC, the product was obtained as a colorless oil (22.9 mg, 0.10 mmol, 34%) using 0% to 5% EtOAc in *n*-hexane as the eluent. The product was unreported in the literature.

4-(4-Methylquinolin-2-yl)pentanenitrile (32):

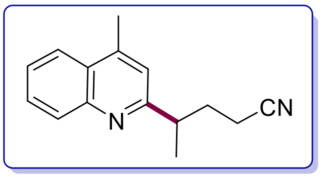

TLC: Rf = 0.18 (95:5 hexane/EtOAc, UV).

GC (Ti= 80 ºC): Rt 7.980 min.

MS: *m/z* (%) 224 (M^+,14), 171 (100), 184 (75), 170 (64), 115 (15), 172 (14).

HRMS (EI-TOF): *m/z* calcd for $C_{15}H_{16}N_2$ 224,1313, found 224.1307.

^{1}H NMR (400 MHz, $CDCl_3$) δ 8.22 (d, *J* = 8.3 Hz, 1H), 8.00 (dd, *J* = 8.4, 1.4 Hz, 1H), 7.75 (ddd, *J* = 8.3, 6.8, 1.4 Hz, 1H), 7.63 – 7.52 (m, 1H), 7.22 (s, 1H), 3.35 (s, 1H), 2.75 (s, 3H), 2.41-2.29 (m, 3H), 2.17-2.06 (m, 1H), 1.44 (d, *J* = 6.9 Hz, 3H).

^{13}C NMR (101 MHz, $CDCl_3$) δ 163.2, 152.6, 147.1, 130.2, 128.3, 127.2, 126.6, 123.8, 120.9, 119.7, 40.5, 31.7, 20.7, 19.1, 15.5.

Alkylation of Lepidine with isoamyl acetate

Following the GP (but adding 20 mol% of pyridine *N*-oxide at the beginning of the reaction and an extra 10 mol% after 24 h) with lepidine (43 µL, 0.30 mmol) and isoamyl acetate (390 µL, 3 mmol, 10 equiv) in 46 h. After purification by FC, the product was obtained as a colorless oil (26 mg, 0.1 mmol, 32%) using 1% to 10% EtOAc in *n*-hexane as the eluent. The product was unreported in the literature.

Synthesis of 3-methyl-3-(4-methylquinolin-2-yl)butyl acetate (33):

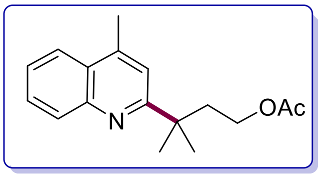

TLC: Rf = 0.54 (9:1 Hexane/EtOAc, UV)

GC (Ti= 80 ºC): The product is not seen in GC/MS.

HRMS (EI-TOF): *m/z* calcd for $C_{17}H_{21}NO_2$ 271.1572 found 271.1569.

^{1}H NMR (400 MHz, $CDCl_3$): δ 8.05 (d, *J* = 8.4 Hz, 1H), 7.94 (dd, *J* = 8.4, 1.4 Hz, 1H), 7.66 (ddd, *J* = 8.4, 6.8, 1.5 Hz, 1H), 7.50 (ddd, *J* = 8.2, 6.8, 1.3 Hz, 1H), 7.31 (d, *J* = 1.2 Hz, 1H), 4.04 (dd, *J* = 7.9, 7.0 Hz, 2H), 2.69 (d, *J* = 1.0 Hz, 3H), 2.22 (t, *J* = 7.4 Hz, 2H), 1.86 (s, 3H), 1.49 (s, 6H).

^{13}C NMR (101 MHz, $CDCl_3$): δ 171.0, 166.7, 147.2, 143.9, 129.9, 128.8, 126.5, 125.6, 123.3, 118.9, 62.2, 40.9, 39.8, 28.2, 20.9, 19.0.

Alkylation of Lepidine with *N*-methylacetamide

Following the GP (but adding 20 mol% of pyridine *N*-oxide at the beginning of the reaction and an extra 10 mol% after 24 h) with lepidine (43 µL, 0.30 mmol) and *N*-methylacetamide (228 µL, 3 mmol, 10 equiv) in 46

h. After purification by FC, the product was obtained as a green oil (27 mg, 0.13 mmol, 42%) using from 5% to 40% EtOAc:EtOH (2:1) in *n*-hexane as the eluent. The spectroscopy data matched with previously reported in the literature.[24]

Synthesis of *N*-((4-methylquinolin-2-yl)methyl)acetamide (34):

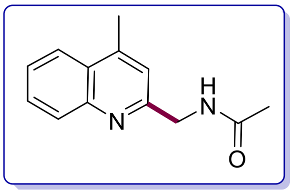

TLC: Rf = 0.33 (7:3 hexane/(2:1)EtOAc:EtOH, UV).

GC (Ti= 80 °C): Rt: 8.5 min

MS: *m/z* (%) 171 (M^+, 100), 214 (31).

^{1}H NMR (400 MHz, $CDCl_3$):δ 8.20 (d, *J* = 8.4 Hz, 1H), 8.01 (dd, *J* = 8.4, 1.4 Hz, 1H), 7.76 (ddd, *J* = 8.3, 6.8, 1.3 Hz, 1H), 7.61 (dd, *J* = 8.4, 6.9 Hz, 1H), 7.35 (s, 1H), 7.29 (s, 1H), 4.72 (s, 2H), 2.74 (d, *J* = 0.8 Hz, 3H), 2.12 (s, 3H).

^{13}C NMR (101 MHz, $CDCl_3$): δ 170.3, 155.7, 146.4, 146.0, 129.7, 128.7, 127.4, 126.4, 123.8, 120.8, 44.6, 23.2, 18.8.

Alkylation of Lepidine with pyrrolidone

Following the GP (but adding 20 mol% of pyridine *N*-oxide at the beginning of the reaction and an extra 10 mol% after 24 h) with lepidine (43 µL, 0.30 mmol) and pyrrolidone (228 µL, 3 mmol, 10 equiv) in 46 h. After purification by FC, the product was obtained as a green solid (25 mg, 0.11 mmol, 37%) using from 10% to 50% EtOAc:EtOH (2:1) in *n*-hexane as the eluent. The spectroscopy data matched with previously reported in the literature.[25]

Synthesis of 5-(4-methylquinolin-2-yl) pyrrolidin-2-one (35):

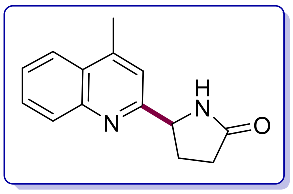

TLC: Rf = 0.25 (7:3 hexane/EtOAc:EtOH (2:1), UV).

GC (Ti= 80 °C): Rt: 9.5 min

MS: *m/z* (%) 143 (M^+, 100), 198 (88), 171 (41), 226 (39), 144 (32), 170 (24), 115 (22)

^{1}H NMR (400 MHz, $CDCl_3$): δ 8.07 (d, *J* = 8.4 Hz, 1H), 7.98 (dd, *J* = 8.4, 1.6 Hz, 1H), 7.71 (ddd, *J* = 8.4, 6.7, 1.6 Hz, 1H), 7.57 (ddd, *J* = 8.3, 5.1, 1.3 Hz, 1H), 7.27 (d, *J* = 8.7 Hz, 1H), 6.76 (s, 1H), 5.05 (t, *J* = 7.3 Hz, 1H), 2.72 (s, 1H), 2.71 (s, 3H), 2.50 (ddd, *J* = 9.5, 6.7, 4.6 Hz, 2H), 2.16 (dd, *J* = 6.6, 3.0 Hz, 1H).

^{13}C NMR (101 MHz, $CDCl_3$): δ 178.7, 178.0, 160.9, 146.7, 129.9, 129.1, 127.5, 126.6, 123.7, 118.1, 59.3, 30.1, 29.0, 19.0.

Alkylation of Lepidine with 1,4-Dioxane

Following the GP (but adding 20 mol% of pyridine *N*-oxide at the beginning of the reaction and an extra 10 mol% after 24 h) with lepidine (43 µL, 0.30 mmol) and dioxane (258 µL, 3 mmol, 10 equiv) in 46 h. After purification by FC, the product was obtained as a colorless oil (55.6 mg, 0.24 mmol, 81%) using from 10% to

20% EtOAc in *n*-hexane as the eluent. The spectroscopy data matched with previously reported in the literature.[13]

2-(1,4-dioxan-2-yl)-4-methylquinoline (36):

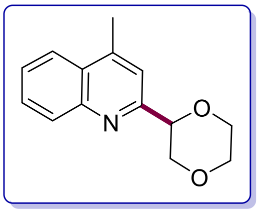

TLC: Rf = 0.18 (9:1 hexane/EtOAc, UV).

GC (Ti= 80 ºC): Rt 8.095 min.

MS: *m/z* (%) 229 (M^+, 15), 172 (42), 171 (52), 143 (36), 142 (14), 115 (20).

^{1}H NMR (300 MHz, $CDCl_3$) δ 8.08 (ddd, *J* = 8.5, 1.3, 0.6 Hz, 1H), 7.97 (ddd, *J* = 8.4, 1.5, 0.7 Hz, 1H), 7.69 (ddd, *J* = 8.4, 6.9, 1.5 Hz, 1H), 7.54 (ddd, *J* = 8.3, 6.8, 1.3 Hz, 1H), 7.46 (d, *J* = 1.1 Hz, 1H), 4.90 (dd, *J* = 10.1, 2.9 Hz, 1H), 4.23 (ddd, *J* = 11.6, 2.9, 0.7 Hz, 1H), 4.06 – 3.93 (m, 2H), 3.88 – 3.72 (m, 2H), 3.63 (dd, *J* = 11.6, 10.1 Hz, 1H), 2.71 (d, *J* = 0.9 Hz, 3H).

^{13}C NMR (101 MHz, $CDCl_3$) δ 157.8, 147.2, 145.5, 129.8, 129.5, 127.7, 126.4, 123.8, 119.2, 78.8, 71.2, 67.2, 66.5, 19.0.

Alkylation of Lepidine with Tetrahydrofuran

Following the GP (but adding 20 mol% of pyridine *N*-oxide at the beginning of the reaction and an extra 10 mol% after 24 h) with lepidine (43 µL, 0.30 mmol) and tetrahydrofuran (243 µL, 3 mmol, 10 equiv) in 48 h. After purification by FC, the product was obtained as a colorless oil (38.3 mg, 0.18 mmol, 60%) using a gradient from 20% to 30% EtOAc in *n*-hexane as the eluent. The spectroscopy data matched with previously reported in the literature.[13]

4-methyl-2-(tetrahydrofuran-2-yl)quinoline (37):

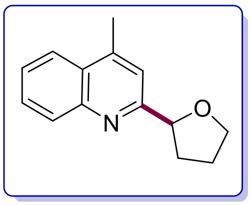

TLC: Rf = 0.36 (8:2 hexane/EtOAc, UV).

GC (Ti= 80 ºC): Rt 7.679 min.

MS: *m/z* (%) 213 (M^+,4), 184 (12), 171 (28), 170 (100), 157 (75), 143 (22), 115 (22).

^{1}H NMR (400 MHz, $CDCl_3$) δ 8.06 (dd, *J* = 8.5, 1.4 Hz, 1H), 7.97 (dd, *J* = 8.4, 1.5 Hz, 1H), 7.68 (ddd, *J* = 8.4, 6.8, 1.4 Hz, 1H), 7.52 (ddd, *J* = 8.2, 6.8, 1.3 Hz, 1H), 7.44 (d, *J* = 1.2 Hz, 1H), 5.14 (t, *J* = 6.9 Hz, 1H), 4.18 (dt, *J* = 8.0, 6.3 Hz, 1H), 4.04 (dt, *J* = 7.9, 6.5 Hz, 1H), 2.71 (d, *J* = 1.0 Hz, 3H), 2.56 – 2.45 (m, 1H), 2.10 – 1.99 (m, 3H).

^{13}C NMR (101 MHz, $CDCl_3$) δ 163.2, 147.3, 145.2, 129.6, 129.3, 127.6, 125.9, 123.8, 118.7, 82.2, 69.4, 33.5, 26.1, 19.1.

Alkylation of Lepidine with *tert*-butyl methyl ether

Synthesis of 2-(tert-butoxymethyl)-4-methylquinoline (38):

Following the GP (but adding 20 mol% of pyridine *N*-oxide at the beginning of the reaction and an extra 10 mol% after 24 h) with lepidine (43 µL, 0.30 mmol) and *tert*-butyl methyl ether (356 µL, 3 mmol, 10 equiv) in 46 h. After purification by FC, the product was obtained as a yellow oil (45 mg, 0.20 mmol, 67%) using from

5% to 10% EtOAc in *n*-hexane as the eluent. The spectroscopy data matched with previously reported in the literature.[12]

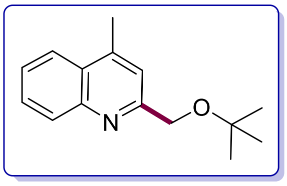

TLC: Rf = 0.28 (9:1 hexane/EtOAc, UV).

GC (Ti= 80 ºC): Rt: 7.2 min

MS: *m/z* (%) 172 (M^+, 91), 172 (66), 199 (65), 173 (59), 143 (33), 144 (29).

^{1}H NMR (400 MHz, $CDCl_3$) δ 8.02 (ddd, *J* = 8.5, 1.3, 0.6 Hz, 1H), 7.96 (ddd, *J* = 8.3, 1.5, 0.6 Hz, 1H), 7.67 (ddd, *J* = 8.4, 6.9, 1.4 Hz, 1H), 7.54 – 7.47 (m, 2H), 4.72 (s, 2H), 2.71 (d, *J* = 1.0 Hz, 3H), 1.34 (s, 9H).

^{13}C NMR (101 MHz, $CDCl_3$) δ 160.4, 147.2, 144.9, 129.4, 129.2 , 127.6, 125.8, 123.8, 120.1, 74.1, 66.1, 27.8, 19.9.

Alkylation of Lepidine with Methanol

Following the GP (methanol instead of HFIP) with lepidine (43 µL, 0.30 mmol) and methanol (121 µL, 3 mmol, 10 equiv) in 46 h. After purification by FC, the product was obtained as a colorless oil (34 mg, 0.2 mmol, 66%) using from 10% to 30% EtOAc in *n*-hexane as the eluent. The spectroscopy data matched with previously reported in the literature.[21]

4-Methylquinolin-2-yl)methanol (39):

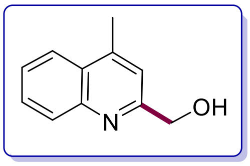

TLC: Rf = 0.2 (7:3 hexane/EtOAc, UV).

GC (Ti= 80 ºC): Rt: 6.8 min.

MS: *m/z* (%) 173 (M^+, 70), 144 (63), 143 (49), 115 (32), 207 (23).

^{1}H NMR (400 MHz, $CDCl_3$) δ 8.07 (dt, *J* = 8.4, 1.1 Hz, 1H), 7.97 (dd, *J* = 8.4, 1.4 Hz, 1H), 7.71 (ddd, *J* = 8.3, 6.9, 1.4 Hz, 1H), 7.55 (ddd, *J* = 8.3, 6.9, 1.3 Hz, 1H), 7.12 (d, *J* = 1.2 Hz, 1H), 4.87 (s, 2H), 2.69 (d, *J* = 1.0 Hz, 3H).

^{13}C NMR (101 MHz, $CDCl_3$) δ 158.8, 146.5, 145.3, 129.6, 129.1, 127.7, 126.2, 123.9, 119.1, 64.1, 18.9.

Alkylation of Lepidine with Methanol-d4

Following the GP with lepidine (43 µL, 0.30 mmol) and CD_3OD (136 µL, 3 mmol, 10 equiv) in 46 h. After purification by FC, the product was obtained as a colorless oil (32 mg, 0.18 mmol, 60%) using from 10% to 30% EtOAc in *n*-hexane as the eluent. The product was unreported in the literature.

4-Methylquinolin-2-yl)methan-d2-ol (40):

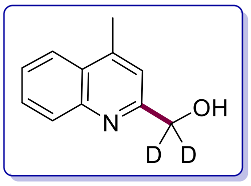

TLC: Rf = 0.16 (7:3 hexane/EtOAc, UV).

GC (Ti= 80 ºC): Rt: 6.3 min

MS: *m/z* (%) 271 (M^+, 100), 174 (86), 158,1 (69), 143 (40), 115 (29), 202 (29), 159 (26), 142 (26), 144 (21), 207 (21).

HRMS: (EI-TOF): *m/z* calcd for $C_{11}H_9D_2NO$ 175.0966, found 175.0964.

^{1}**H NMR (300 MHz, CDCl$_3$)** δ 8.06 (ddd, *J* = 8.4, 1.3, 0.6 Hz, 1H), 7.97 (ddd, *J* = 8.3, 1.5, 0.6 Hz, 1H), 7.70 (ddd, *J* = 8.4, 6.9, 1.5 Hz, 1H), 7.55 (ddd, *J* = 8.2, 6.9, 1.3 Hz, 1H), 7.12 (d, *J* = 1.0 Hz, 1H), 2.68 (d, *J* = 1.0 Hz, 3H).

^{13}**C NMR (101 MHz, CDCl$_3$)** δ 158.7, 146.6, 145.3, 129.6, 129.2, 127.7, 126.2, 123.9, 119.1, 18.9.

Alkylation of Lepidine with Ethanol

Following the GP (but adding 20 mol% of pyridine *N*-oxide at the beginning of the reaction and an extra 10 mol% after 24 h) with lepidine (43 µL, 0.30 mmol) and ethanol (175 µL, 3 mmol, 10 equiv) in 46 h. After purification by FC, the product was obtained as a colorless oil (20.2 mg, 0.11 mmol, 36%) using from 10% to 30% EtOAc in *n*-hexane as the eluent. The spectroscopy data matched with previously reported in the literature.[26]

1-(4-Methylquinolin-2-yl)ethan-1-ol (41):

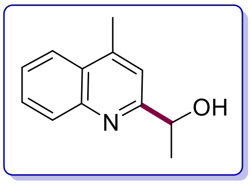

TLC: Rf = 0.31 (7:3 hexane/EtOAc, UV).

GC (Ti= 80 ºC): Rt 6.776 min.

MS: *m/z* (%) 187 (M^+,20), 186 (19), 170 (60), 144 (54), 143 (40), 142 (27), 115 (26).

^{1}**H NMR** (400 MHz, CDCl$_3$) *δ* 8.09 (d, *J* = 8.4 Hz, 1H), 7.98 (d, *J* = 9.4 Hz, 1H), 7.72 (ddd, *J* = 8.3, 6.9, 1.3 Hz, 1H), 7.56 (ddd, *J* = 8.2, 7.0, 1.1 Hz, 1H), 7.20 (s, 1H), 5.01 (q, *J* = 6.6 Hz, 1H), 2.72 (s, 3H), 1.57 (d, *J* = 6.6 Hz, 3H).

^{13}**C NMR** (101 MHz, CDCl$_3$) *δ* 162.5, 145.8, 145.7, 129.6, 129.0, 127.5, 126.3, 123.8, 118.6, 68.6, 24.1, 19.0.

Alkylation of Lepidine with Isoamyl Alcohol

Following the GP (but adding 20 mol% of pyridine *N*-oxide at the beginning of the reaction and an extra 10 mol% after 24 h) with lepidine (43 µL, 0.30 mmol) and isoamyl alcohol (326 µL, 3 mmol, 10 equiv) in 46 h. After purification by FC, the product was obtained as a colorless oil (37.8 mg, 0.16 mmol, 55%) using 0% to 5% EtOAc in *n*-hexane as the eluent. The product was unreported in the literature.

3-Methyl-2-(4-methylquinolin-2-yl)butan-1-ol and 3-Methyl-3-(4-methylquinolin-2-yl)butan-1-ol (42):

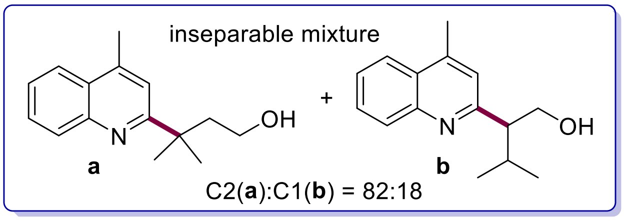

TLC: Rf = 0.18 (9:1 hexane/EtOAc, UV).

GC (Ti= 80 ºC): Rt 7.930 min.

HRMS (EI-TOF): *m/z* calcd for $C_{15}H_{19}NO$ 229,1467, found 229.1465.

3-Methyl-2-(4-methylquinolin-2-yl)butan-1-ol (42a):

^{1}**H NMR** (400 MHz, CDCl$_3$) *δ* 8.02 (dd, *J* = 8.3, 1.0 Hz, 1H), 7.94 (dd, *J* = 8.3, 1.3 Hz, 1H), 7.66 (ddd, *J* = 8.3, 6.9, 1.3 Hz, 1H), 7.51 (ddd, *J* = 8.3, 6.9, 1.0 Hz, 1H), 7.36 (d, *J* = 1.1 Hz, 1H), 3.80 (t, *J* = 5.7 Hz, 2H), 2.70 (d, *J* = 0.9 Hz, 3H), 2.13 (t, *J* = 5.7 Hz, 2H), 1.46 (s, 6H).

^{13}**C NMR** (101 MHz, CDCl$_3$) *δ* 167.9, 145.9, 145.3, 129.6, 128.8, 126.7, 126.2, 123.6, 119.8, 59.3, 43.9, 42.0, 29.6, 19.2.

3-Methyl-3-(4-methylquinolin-2-yl)butan-1-ol (42b): **^{1}H NMR** (400 MHz, $CDCl_3$) δ 8.07 (d, J = 8.3 Hz, 1H), 7.97 (dd, J = 8.3, 1.6 Hz, 1H), 7.71 – 7.68 (m, 1H), 7.56 – 7.53 (m, 1H), 7.13 (d, J = 1.0 Hz, 1H), 4.11 (dd, J = 11.0, 5.2 Hz, 1H), 4.01 (dd, J = 11.0, 2.7 Hz, 1H), 2.68 – 2.61 (m, 1H), 2.70 (d, J = 0.9 Hz, 3H), 2.36 (dp, J = 8.9, 6.7 Hz, 1H), 1.11 (d, J = 6.7 Hz, 2H), 0.82 (d, J = 6.7 Hz, 2H).

^{13}C NMR (101 MHz, $CDCl_3$) δ 164.8, 146.4, 145.3, 129.7, 129.0, 127.0, 126.1, 123.8, 123.1, 63.3, 54.8, 29.8, 21.7, 20.8, 19.0.

Alkylation of Lepidine with 3-methylbutanal

Following the GP (but adding 20 mol% of pyridine *N*-oxide at the beginning of the reaction and an extra 10 mol% after 24 h) with lepidine (43 µL, 0.30 mmol) and 3-methylbutanal (322 µL, 3 mmol, 10 equiv) in 46 h. After purification by FC, the product was obtained as a colorless oil (25 mg, 0.11 mmol, 37%) using from 1% to 20% EtOAc in *n*-hexane as the eluent. The spectroscopy data matched with previously reported in the literature.[26]

3-Methyl-1-(4-methylquinolin-2-yl(butan-1-ol) (43):

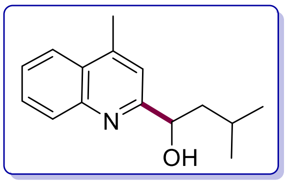

TLC: Rf = 0.37 (9:1 hexane/EtOAc, UV).

GC (Ti= 80 ºC): Rt: 7.7 min

MS: *m/z* (%) 173 (M^+, 100), 172 (61)

^{1}H NMR (400 MHz, $CDCl_3$): δ 8.10 (ddd, J = 8.4, 1.3, 0.6 Hz, 1H), 7.98 (ddd, J = 8.3, 1.4, 0.6 Hz, 1H), 7.71 (ddd, J = 8.4, 6.9, 1.4 Hz, 1H), 7.56 (ddd, J = 8.2, 6.9, 1.3 Hz, 1H), 7.18 (d, J = 1.0 Hz, 1H), 4.97 – 4.87 (m, 1H), 2.71 (d, J = 1.0 Hz, 3H), 2.12 – 1.94 (m, 1H), 1.68 – 1.56 (m, 2H), 1.07 (d, J = 6.6 Hz, 3H), 0.97 (d, J = 6.7 Hz, 3H).

^{13}C NMR (101 MHz, $CDCl_3$): δ 162.4, 145.8, 145.6, 129.6, 128.9, 127.5, 126.2, 123.7, 119.0, 70.9, 47.8, 24.9, 23.7, 21.8, 18.9.

Alkylation of Lepidine with lilial

Following the GP (but adding 20 mol% of pyridine *N*-oxide at the beginning of the reaction and an extra 10 mol% after 24 h) with lepidine (43 µL, 0.30 mmol) and buthylphenyl methylpropional (lilial) (290 µL, 1.5 mmol, 5 equiv) in 46 h. After purification by FC, the product was obtained as a colorless oil (60 mg, 0.20 mmol, 63 %) using 1% to 5% EtOAc in *n*-hexane as the eluent. The spectroscopy data matched with previously reported in the literature.[21]

Synthesis of 2-(1-(4-(tert-butyl)phenyl)propan-2-yl)-4-methylquinoline (44):

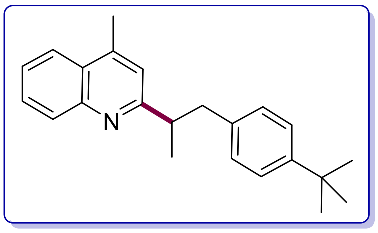

TLC: Rf = 0.60 (97:3 Hexane/EtOAc), UV).

GC (Ti= 80 ºC): 9.7 min

MS: *m/z* (%) 302 (M^+, 100), 317 (51), 303 (45).

^{1}H NMR (400 MHz, $CDCl_3$): δ 8.14 (d, J = 8.4 Hz, 1H), 7.96 (d, J = 10.5 Hz, 1H), 7.70 (t, J = 8.4 Hz, 1H), 7.57 – 7.45 (m, 1H), 7.27 (d, J = 6.3 Hz,

2H), 7.14 (d, J = 8.4 Hz, 2H), 3.40 (h, J = 6.8 Hz, 1H), 3.22 (dd, J = 13.5, 6.1 Hz, 1H), 2.88 (dd, J = 13.6, 8.8 Hz, 1H), 2.68 (s, 3H), 1.36 (d, J = 6.9 Hz, 3H), 1.30 (s, 9H).

^{13}C NMR (101 MHz, $CDCl_3$): δ 165.9, 148.6, 137.4, 129.3, 129.1, 128.9, 127.0, 125.6, 125.0, 123.6, 120.8, 44.2, 42.3, 34.3, 31.4, 19.9, 18.8.

Alkylation of Lepidine with pivalaldehyde

Following the GP (but adding 20 mol% of pyridine *N*-oxide at the beginning of the reaction and an extra 10 mol% after 24 h) with lepidine (43 µL, 0.30 mmol) and pivaldehyde (335 µL, 3 mmol, 10 equiv) in 46 h. After purification by FC, the product was obtained as a yellow oil (40 mg, 0.20 mmol, 68%) using 1% to 5% EtOAc in *n*-hexane as the eluent. The spectroscopy data matched with previously reported in the literature.[21]

Synthesis of 2-(tert-butyl)-4-methylquinoline (45):

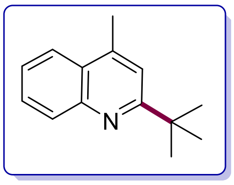

TLC: Rf = 0.6 (9:1 hexane/EtOAc, UV).

GC (Ti= 80 ºC): Rt: 6.1 min

MS: *m/z* (%) 184 (M^+, 100), 199 (49) 157 (30) 143 (28) 185 (25) 198 (24), 157 (24) 198 (22).

^{1}H NMR (400 MHz, $CDCl_3$): δ 8.12 (s, 1H), 7.97 – 7.91 (m, 1H), 7.67 (ddd, J = 8.4, 6.8, 1.5 Hz, 1H), 7.53 – 7.47 (m, 1H), 7.37 (s, 1H), 2.70 (d, J = 1.0 Hz, 3H), 1.49 (s, 9H).

^{13}C NMR (101 MHz, $CDCl_3$): δ 168.8, 147.0, 143.9, 129.7, 128.9, 126.5, 125.5, 123.4, 118.9, 37.9, 30.1, 19.0.

Alkylation of (*S*)-Nicotine with Cyclohexane

Following the GP with (*S*)-nicotine (44 µL, 0.30 mmol) and cyclohexane (162 µL, 3.0 mmol, 5 equiv) in 46 h, but using 3 equivalents of TFA (60 µL, 0.90 mmol, 3 equiv). After purification by FC, the product was obtained as a yellow oil (45 mg, 0.18 mmol, 61%) using a mixture of 40% *n*-hexane, 20% EtOAc and 10% MeOH as the eluent. The spectroscopy data matched with previously reported in the literature.[18]

(*S*)-2-Cyclohexyl-5-(1-methylpyrrolidin-2-yl)pyridine (46):

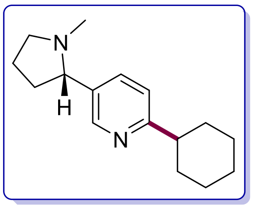

TLC: Rf = 0.33 (4:2:1 hexane/EtOAc/MeOH, UV).

GC (Ti= 80 ºC): Rt 7.939 min.

MS: *m/z* (%) 244 (M^+, 85), 189 (100), 243 (75), 176 (57), 215 (46), 133 (18).

$[\alpha]_D^{25}$ – 68.6 (*c* 1.0, $CHCl_3$)

^{1}H NMR (400 MHz, $CDCl_3$): δ 8.43 – 8.36 (m, 1H), 7.62 (dd, J = 8.1, 2.3 Hz, 1H), 7.13 – 7.07 (m, 1H), 3.28 – 3.18 (m, 1H), 3.10 – 2.99 (m, 1H), 2.67 (t, J = 11.7 Hz, 1H), 2.34 – 2.24 (m, 1H), 2.16 (d, J = 6.6 Hz, 4H), 1.96 – 1.90 (m, 2H), 1.86 – 1.74 (m, 5H), 1.55 – 1.42 (m, 3H), 1.40 – 1.29 (m, 3H).

^{13}C NMR (101 MHz, $CDCl_3$): δ 165.5, 148.7, 135.4, 135.3, 120.9, 68.7, 56.9, 46.2, 40.3, 34.9, 32.9, 26.6, 26.1, 22.5.

Alkylation of Cinchonine with Cyclohexane

Following the GP with cinchonine (88 mg, 0.30 mmol) and cyclohexane (0.7 mL, 6.9 mmol, 23 equiv) in 46 h, but using 3 equivalents of TFA (60 µL, 0.90 mmol, 3 equiv). After purification by FC, the product was obtained as a white solid (60 mg, 0.16 mmol, 53%) using a mixture of 70% *n*-hexane and 30% EtOAc:EtOH:NH_3 (75:25:2 v/v) as the eluent. The spectroscopy data matched with previously reported in the literature.[27]

(S)-(2-Cyclohexylquinolin-4-yl)((2R,4S,5R)-5-vinylquinuclidin-2-yl)methanol (47)

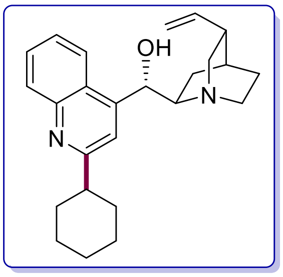

TLC: Rf = 0.3 (7:3 Hexane:(75:25:2, EtOAc/EtOH/NH_3), UV).

$[\alpha]_D^{25}$ + 100.8 (*c* 1.0, MeOH)

^{1}H NMR (300 MHz, $CDCl_3$): δ 8.04 (dt, *J* = 8.6, 1.9 Hz, 1H), 7.92 – 7.82 (m, 1H), 7.67 – 7.53 (m, 2H), 7.35 (ddd, *J* = 8.4, 6.8, 1.4 Hz, 1H), 6.00 (ddd, *J* = 16.4, 11.0, 7.7 Hz, 1H), 5.78 (d, *J* = 3.6 Hz, 1H), 5.12 – 4.92 (m, 2H), 3.37 (dd, *J* = 13.6, 7.9 Hz, 1H), 3.11 – 3.00 (m, 1H), 2.91 (ddt, *J* = 15.3, 11.9, 5.4 Hz, 3H), 2.84 – 2.74 (m, 1H), 2.23 (q, *J* = 8.5 Hz, 1H), 1.97 (d, *J* = 12.0 Hz, 2H), 1.87 (d, *J* = 12.0 Hz, 2H), 1.77 (d, *J* = 12.5 Hz, 2H), 1.63 (d, *J* = 12.6 Hz, 2H), 1.42 (dt, *J* = 25.1, 11.7 Hz, 5H), 1.12 (ddd, *J* = 13.8, 9.3, 4.7 Hz, 1H), 0.87 (t, *J* = 6.8 Hz, 1H).

^{13}C NMR (126 MHz, $CDCl_3$): δ 166.4, 148.3, 147.7, 139.6, 129.7, 128.7, 125.7, 124.1, 122.3, 116.7, 115.2, 70.6, 60.0, 49.9, 49.3, 47.7, 39.5, 32.8, 32.7, 28.2, 26.5, 26.0, 25.6, 19.9, 11.7.

Alkylation of Quinine with Cyclohexane

Following the GP with quinine (97 mg, 0.30 mmol) and cyclohexane (0.7 mL, 6.9 mmol, 23 equiv) in 46 h, but using 3 equivalents of TFA (60 µL, 0.90 mmol, 3 equiv). After purification by FC, the product was obtained as a yellow solid (55 mg, 0.13 mmol, 45%) using a mixture of 70% *n*-hexane and 30% EtOAc:EtOH:NH_3 (75:25:2 v/v) as the eluent. The spectroscopy data matched with previously reported in the literature.[28]

(R)-(2-cyclohexyl-6-methoxyquinolin-4-yl)((1S,2S,4S,5R)-5-vinylquinuclidin-2-yl)methanol (48)

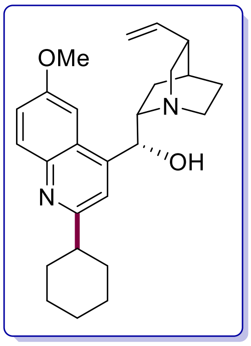

TLC: Rf = 0.53 (7:3 Hexane:(75:25:2, EtOAc/EtOH/NH_3), UV).

$[\alpha]_D^{25}$ – 47.2 (*c* 1.0, $CHCl_3$)

^{1}H NMR (400 MHz, $CDCl_3$) δ 7.93 (d, *J* = 9.2 Hz, 1H), 7.48 (s, 1H), 7.29 – 7.24 (m, 1H), 7.18 (d, *J* = 2.8 Hz, 1H), 5.74 – 5.64 (m, 1H), 5.63 (t, *J* = 3.1 Hz, 1H), 4.99 – 4.86 (m, 2H), 3.86 (s, 3H), 3.67 – 3.51 (m, 1H), 3.14 – 3.00 (m, 2H), 2.76 (s, 1H), 2.70 – 2.59 (m, 2H), 2.28 (s, 1H), 1.98 – 1.71 (m, 9H), 1.54 – 1.45 (m, 3H), 1.40 – 1.25 (m, 4H).

^{13}C NMR (101 MHz, $CDCl_3$) δ 164.0, 157.4, 147.7, 143.8, 141.6, 131.1, 125.1, 121.2, 116.8, 114.7, 101.3, 71.7, 59.8, 57.0, 55.8, 47.4, 43.5, 39.9, 32.9, 28.0, 27.4, 26.6, 26.1, 20.8.

Alkylation of Lepidine with *O*-Methylmenthol

Following the GP (but adding 20 mol% of pyridine *N*-oxide at the beginning of the reaction and an extra 10 mol% after 24 h) with lepidine (43 µL, 0.30 mmol) and *O*-methylmenthol (153 mg, 0.9 mmol, 3 equiv) in 46 h. After purification by FC, the product was obtained as a colorless oil (58.2 mg, 0.18 mmol, 62%) using 0% to 5% EtOAc in *n*-hexane as the eluent. The spectroscopy data matched with previously reported in the literature.[27]

2-(((2-Isopropyl-5-methylcyclohexyl)oxy)methyl)-4-methylquinoline (49):

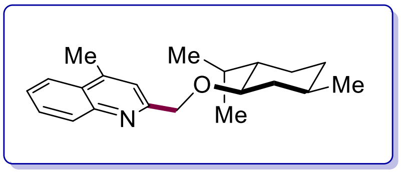

TLC: Rf = 0.42 (95:5 hexane/EtOAc, UV).

GC (Ti= 80 ºC): Rt 9.505 min.

MS: *m/z* (%) 311 (M^+,2), 157 (100), 172 (40), 156 (20), 173 (14), 158 (14).

^{1}H NMR (400 MHz, CDCl$_3$) δ 8.06 (dt, *J* = 8.5, 1.0 Hz, 1H), 7.97 (dd, *J* = 8.3, 1.4 Hz, 1H), 7.68 (ddd, *J* = 8.4, 6.9, 1.4 Hz, 1H), 7.53 (ddd, *J* = 8.2, 6.9, 1.3 Hz, 1H), 7.49 (d, *J* = 1.1 Hz, 1H), 4.91 (d, *J* = 13.2 Hz, 1H), 4.70 (d, *J* = 13.2 Hz, 1H), 3.27 (td, *J* = 10.6, 4.2 Hz, 1H), 2.72 (d, *J* = 1.0 Hz, 3H), 2.37 (pd, *J* = 7.0, 2.7 Hz, 1H), 2.25 (dtd, *J* = 12.1, 3.7, 1.8 Hz, 1H), 1.65 (dddd, *J* = 10.9, 9.0, 5.9, 2.9 Hz, 2H), 1.37 (tddd, *J* = 12.1, 6.3, 5.0, 3.3 Hz, 2H), 1.05 – 0.95 (m, 2H), 0.93 (dd, *J* = 6.8, 1.6 Hz, 6H), 0.90 – 0.84 (m, 1H), 0.74 (d, *J* = 7.0 Hz, 3H).

^{13}C NMR (101 MHz, CDCl$_3$) δ 159.4, 146.8, 144.7, 129.1, 129.0, 127.3, 125.7, 123.5, 120.2, 79.3, 71.6, 48.1, 40.1, 34.3, 31.3, 25.4, 23.0, 22.1, 20.9, 18.6, 15.9.

Alkylation of Lepidine with (-)-ambroxide

Following the GPB with lepidine (43 µL, 0.30 mmol) and ambroxide (700 mg, 3 mmol, 10 equiv) in 46 h. After purification by FC, the product was obtained as a colorless oil (80 mg, 0.21 mmol, 70 %) using from 5% to 20% EtOAc in *n*-hexane as the eluent. The product was unreported in the literature.

Synthesis of (2R,8aS)-2,5,5,8a-tetramethyl-1-(2-(4-methylquinolin-2-yl)ethyl)decahydronaphthalen-2-ol (50)

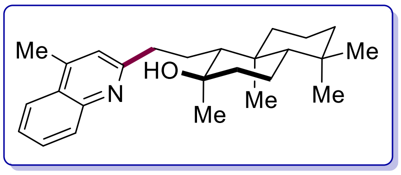

TLC: Rf = 0.24 (8:2 Hexane/EtOAc), UV).

HRMS (EI-TOF): *m/z* calcd for $C_{26}H_{37}NO$ 379.2875, found 379.2864.

$[\alpha]_D^{25}$ + 1.0 (*c* 1.0, MeOH)

^{1}H NMR (400 MHz, CDCl$_3$): *δ* 8.11 (dd, *J* = 8.5, 1.3 Hz, 1H), 7.93 (dd, *J* = 8.3, 1.4 Hz, 1H), 7.66 (s, 1H), 7.50 (s, 1H), 7.09 (d, *J* = 1.1 Hz, 1H), 3.16 (s, 2H), 2.66 (d, *J* = 0.9 Hz, 3H), 2.29 (d, *J* = 6.5 Hz, 1H), 1.94 (d, J = 12.5 Hz, 1H), 1.79 – 1.68 (m, 2H), 1.61 (d, *J* = 13.7 Hz, 2H), 1.45 (d, *J* = 2.0 Hz, 3H), 1.34 (d, *J* = 13.7 Hz, 1H), 1.29 – 1.24 (m, 1H), 1.23 (d, *J* = 0.9 Hz, 3H), 1.13 – 1.03 (m, 1H), 0.97 (t, *J* = 12.7 Hz, 1H), 0.91 (s, 1H), 0.87 (s, 3H), 0.82 (s, 3H), 0.79 (s, 3H).

^{13}C NMR (101 MHz, CDCl$_3$): *δ* 161.7, 146.5, 145.0, 129.5, 128.6, 126.8, 125.9, 123.6, 123.0, 73.4, 59.8, 56.0, 44.6, 42.0, 40.6, 39.6, 38.7, 33.5, 33.3, 24.5, 21.9, 21.6, 20.7, 18.8, 18.6, 15.5.

Unsuccessful substrates:

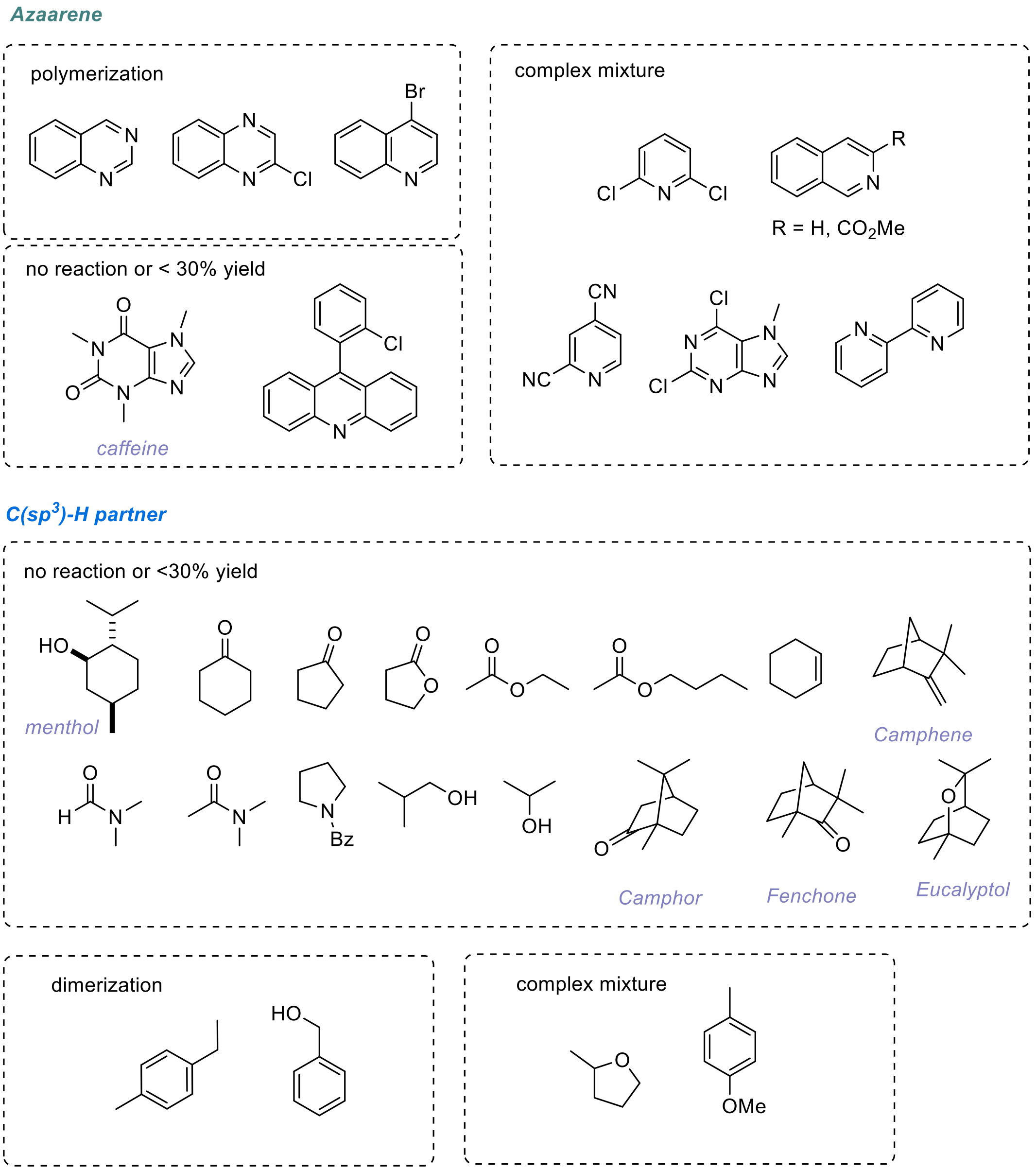

Figure S19: unsuccessful starting materials in the azaarene and the C-H partners

NMR spectra of synthesized compounds

^{1}H NMR (300 MHz, $CDCl_3$) **A1**

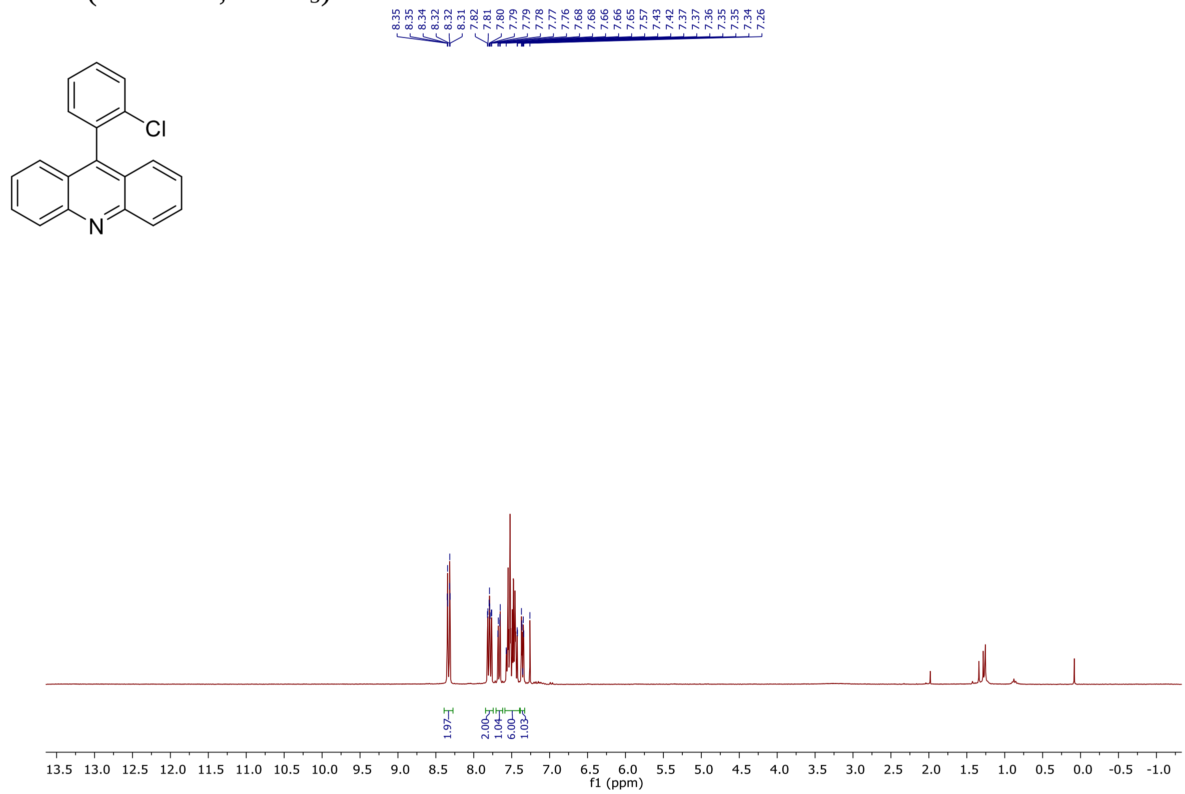

^{1}H NMR (300 MHz, $CDCl_3$) **A2**

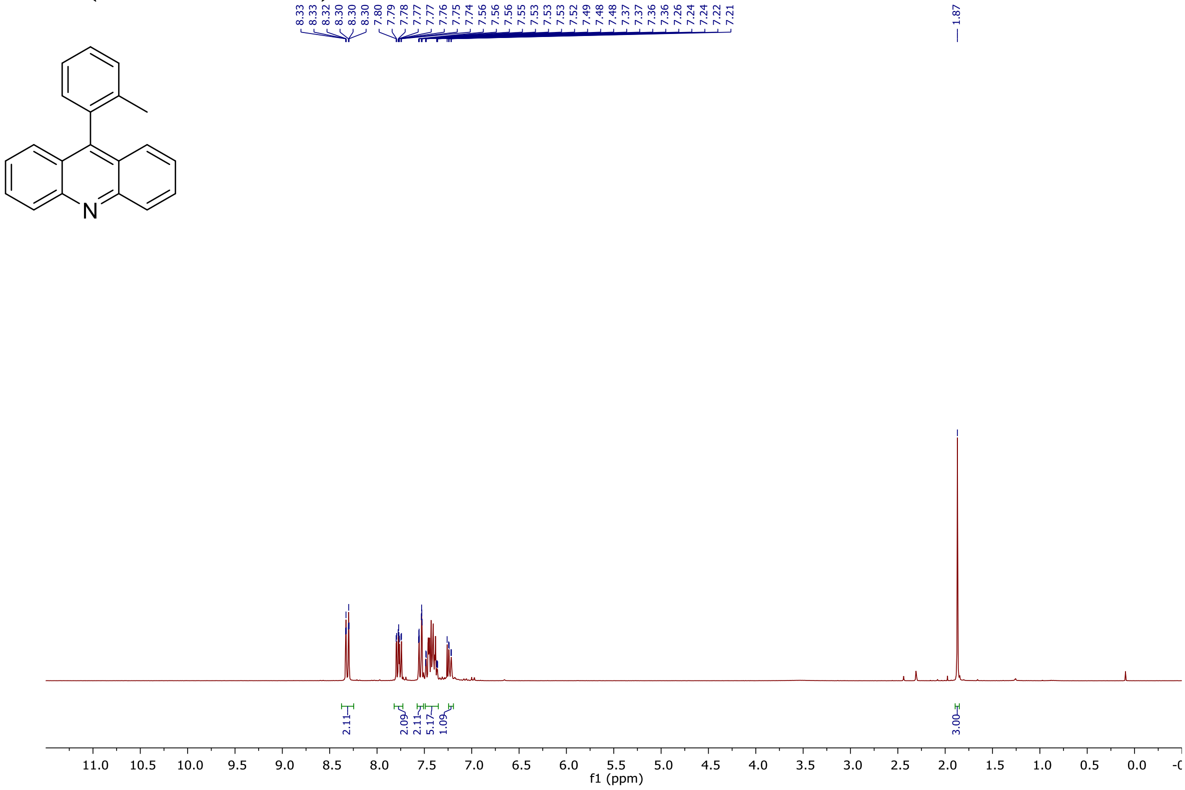

^{1}H NMR (300 MHz, $CDCl_3$) **A3**

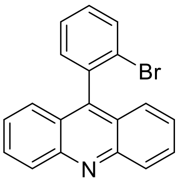

A3

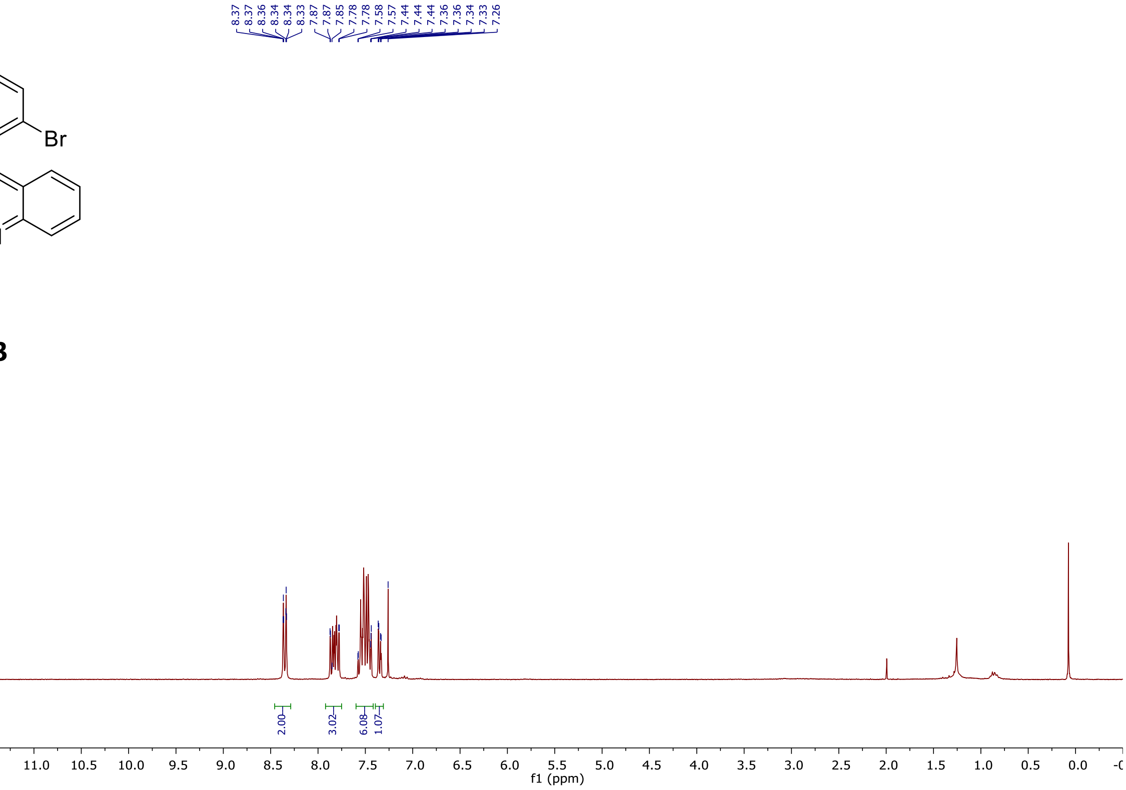

^{1}H NMR (300 MHz, $CDCl_3$) **A4**

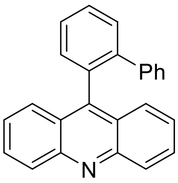

A4

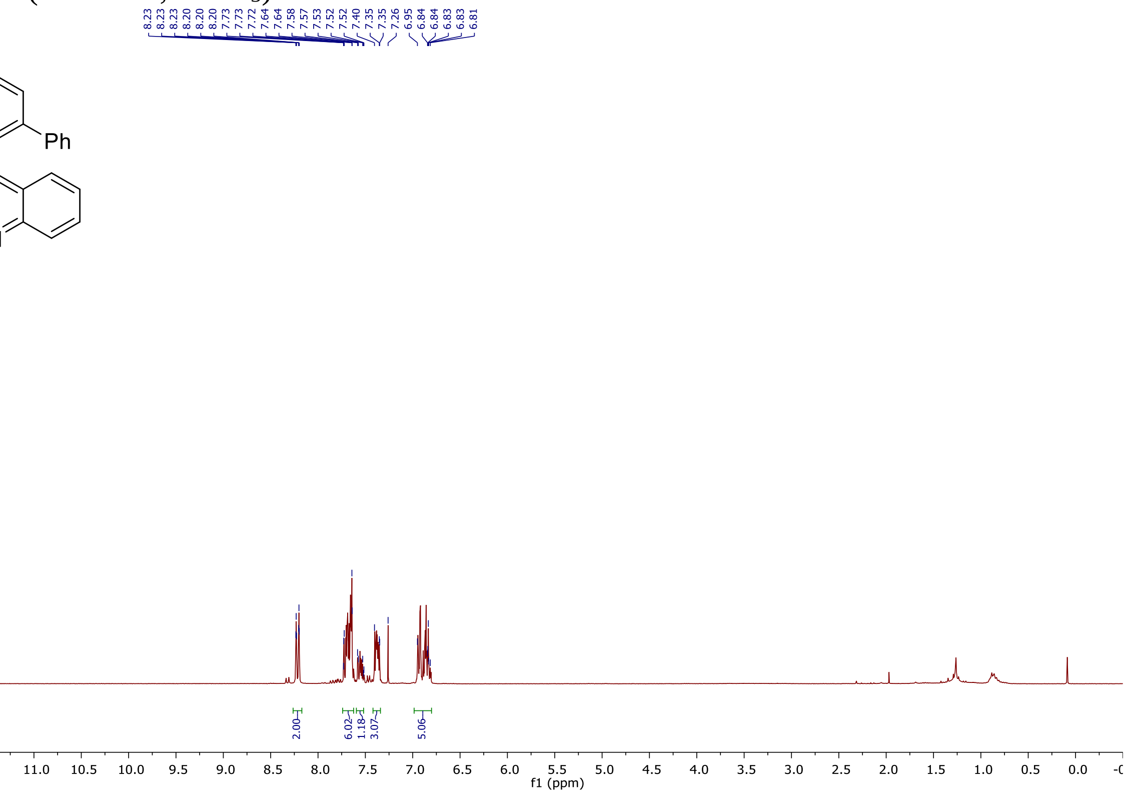

^{1}H NMR (300 MHz, $CDCl_3$) **A5**

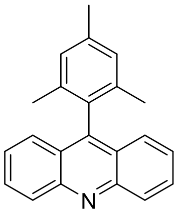

A5

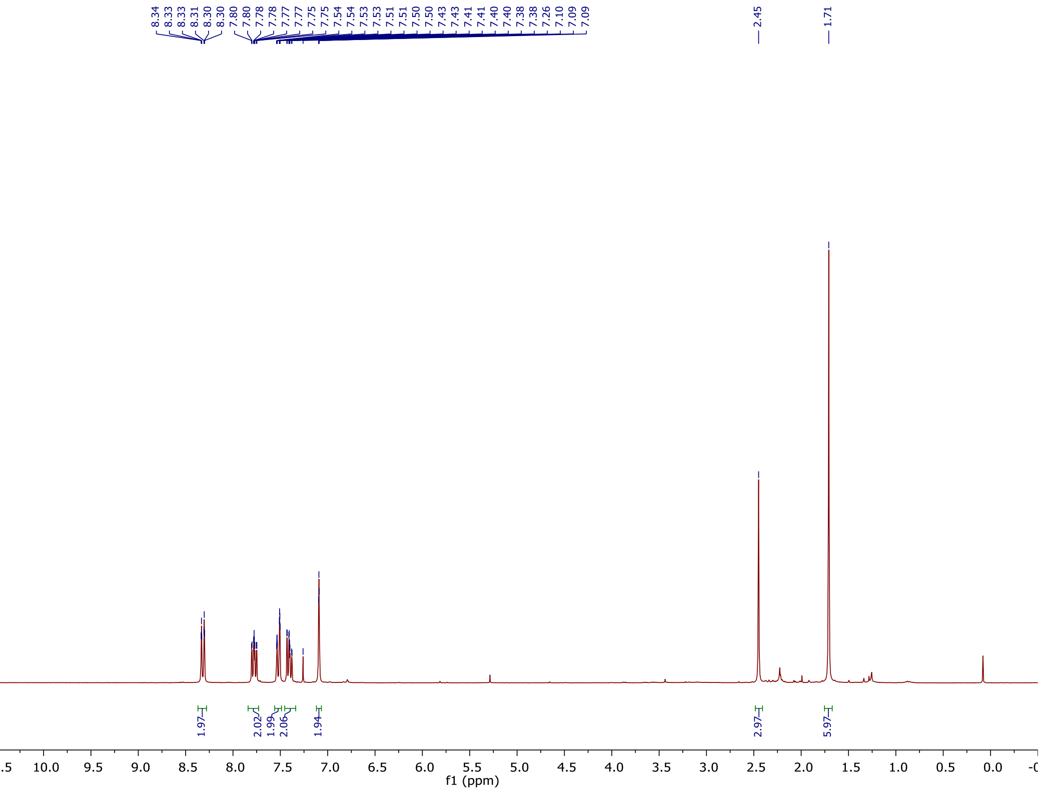

^{1}H NMR (400 MHz, $CDCl_3$) **1**

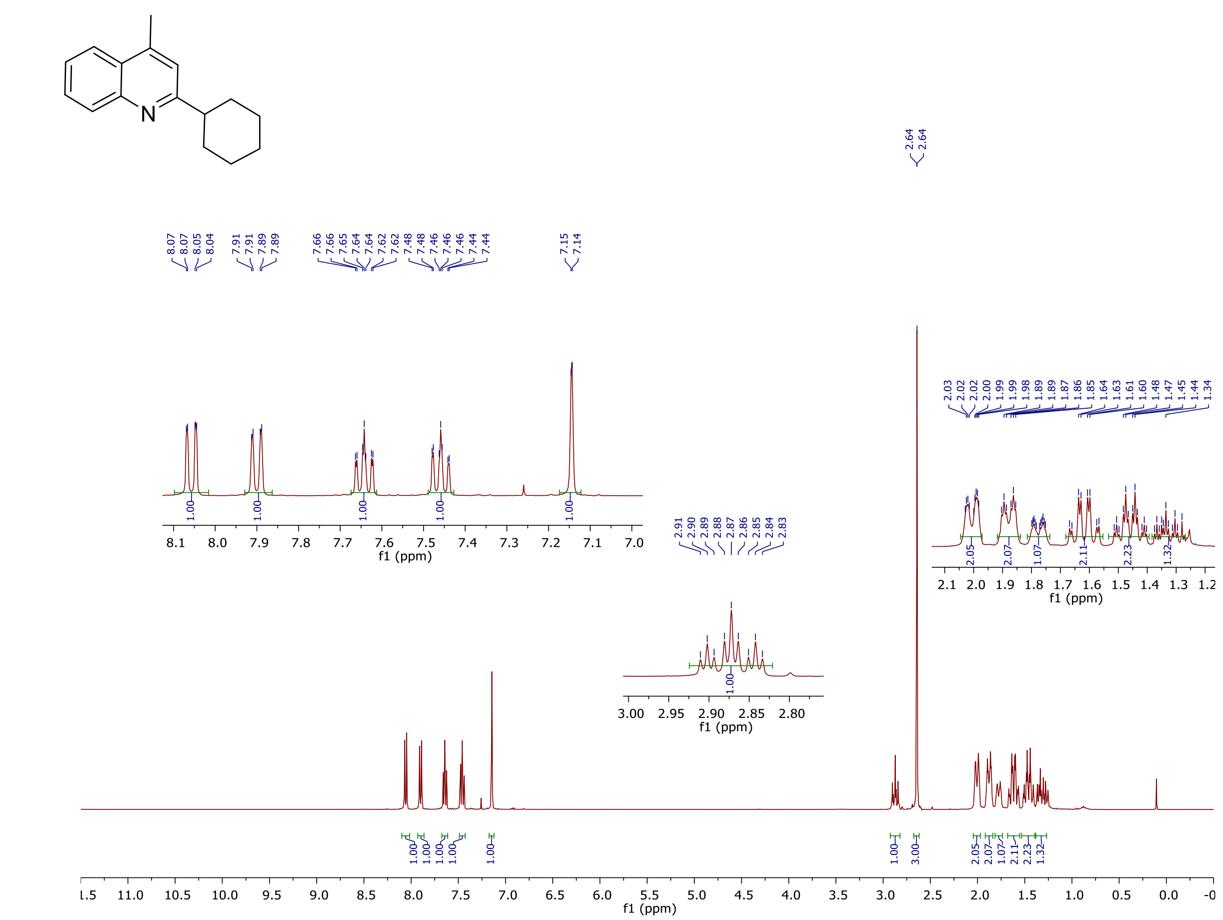

^{13}C NMR (101 MHz, $CDCl_3$) **1**

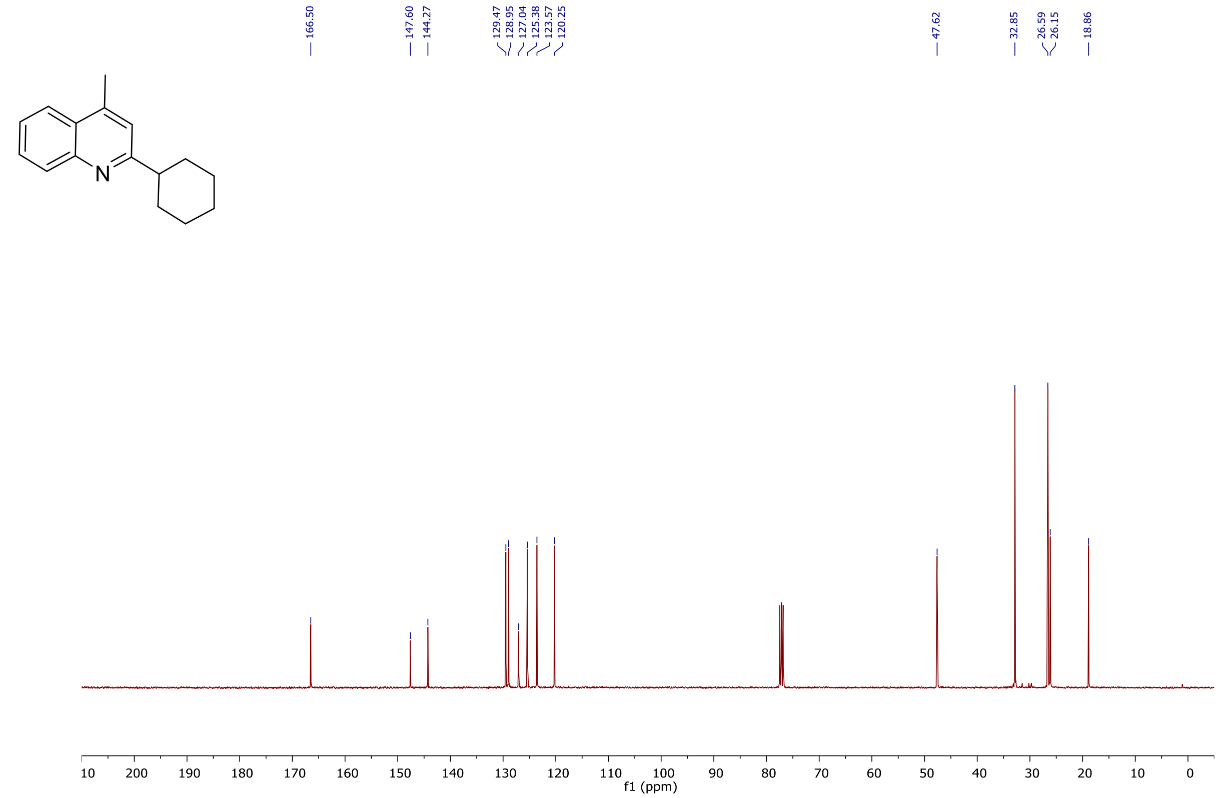

^{1}H NMR (300 MHz, $CDCl_3$) **2**

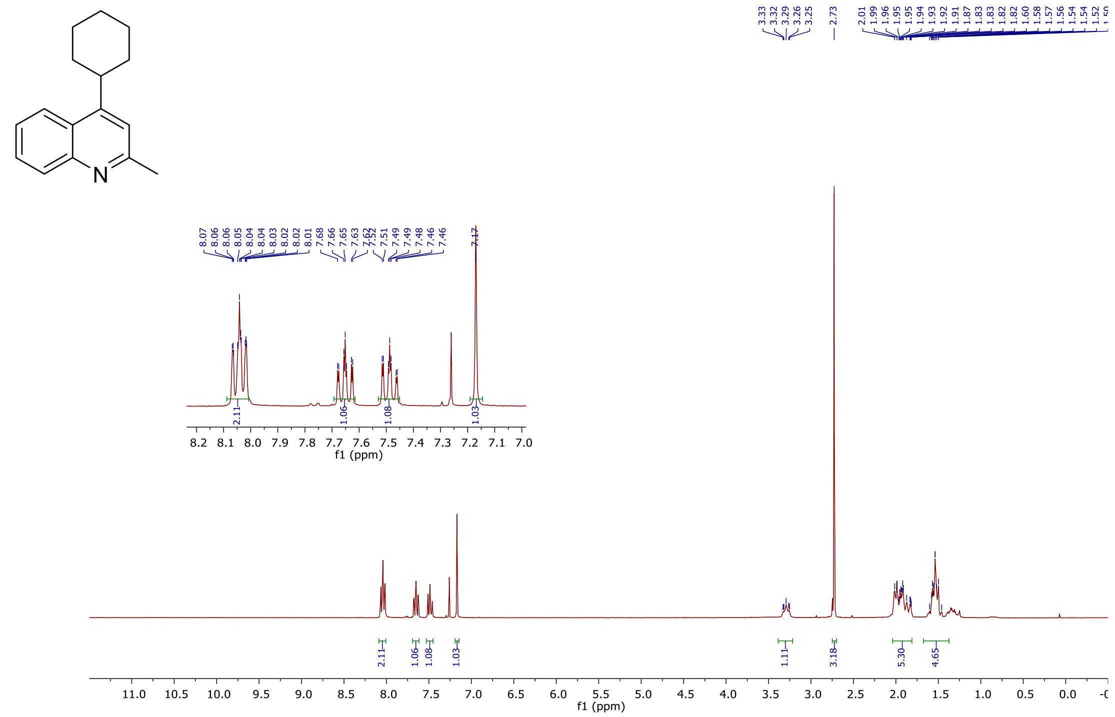

^{13}C NMR (101 MHz, $CDCl_3$) **2**

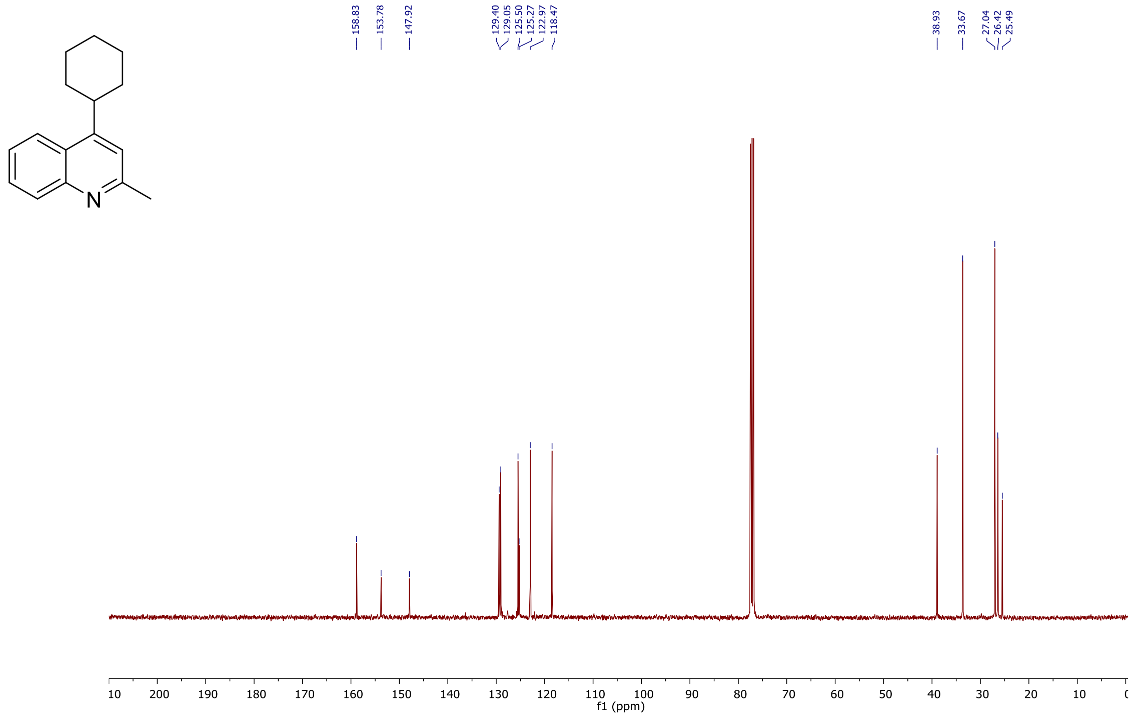

^{1}H NMR (400 MHz, $CDCl_3$) **3**

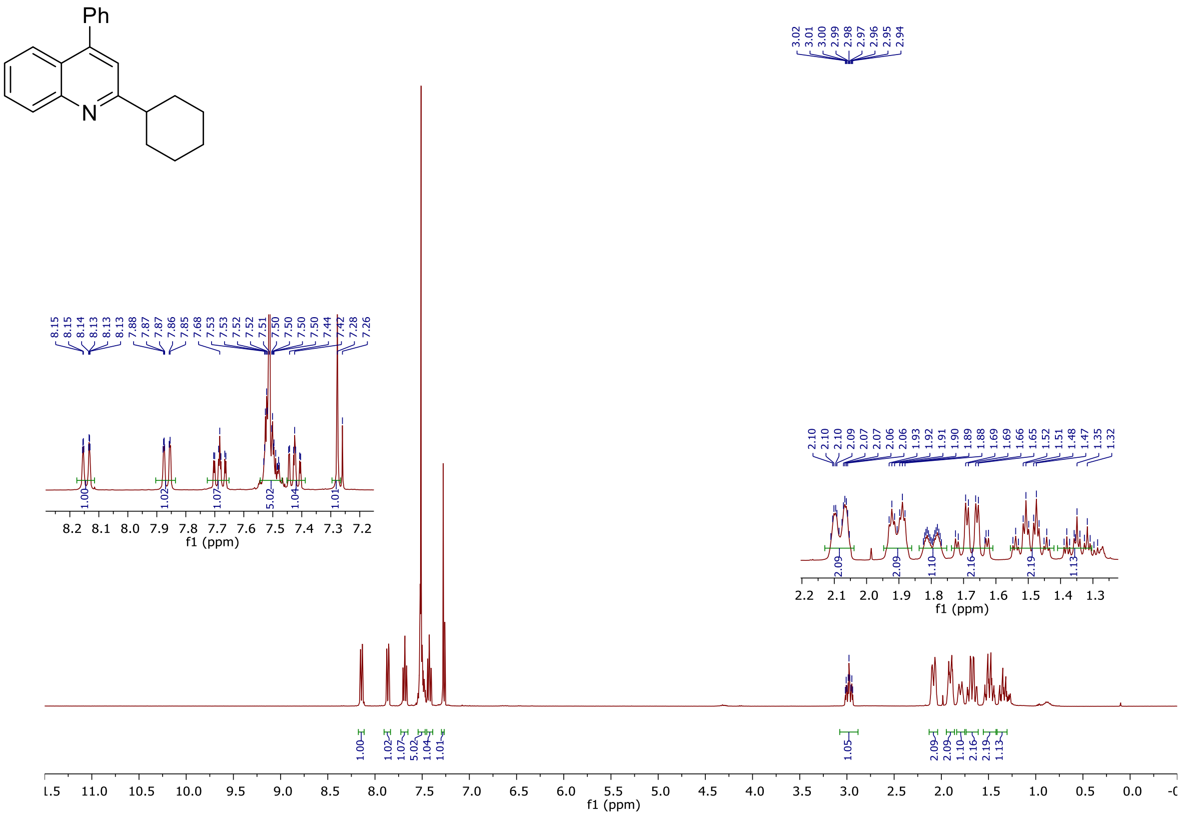

^{13}C NMR (101 MHz, $CDCl_3$) **3**

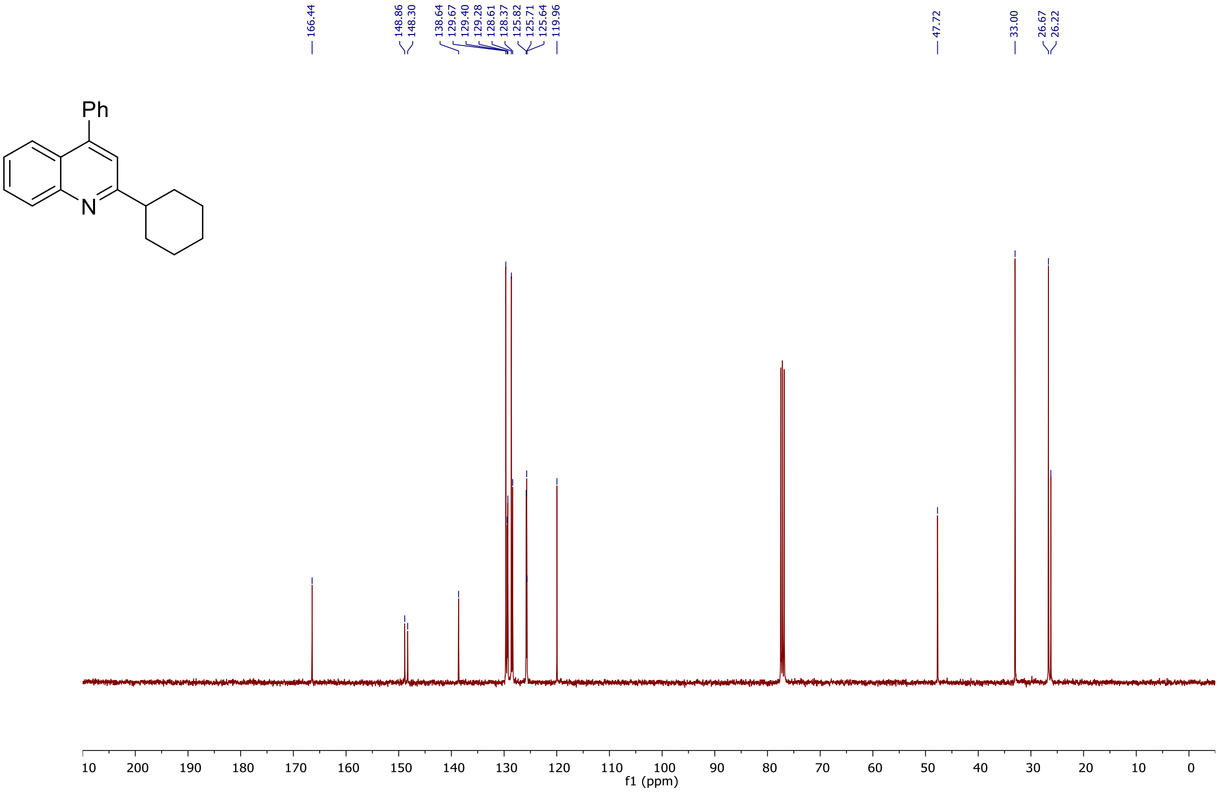

^{1}H NMR (400 MHz, $CDCl_3$) **4**

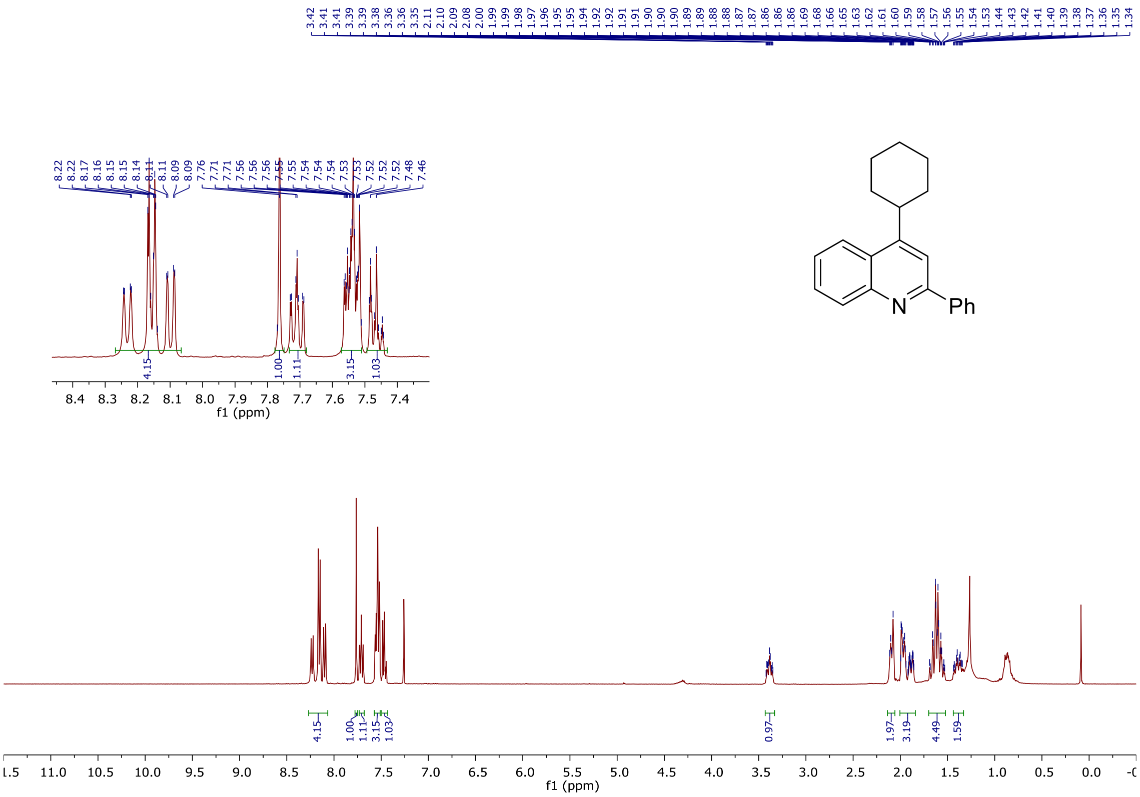

^{13}C NMR (101 MHz, $CDCl_3$) **4**

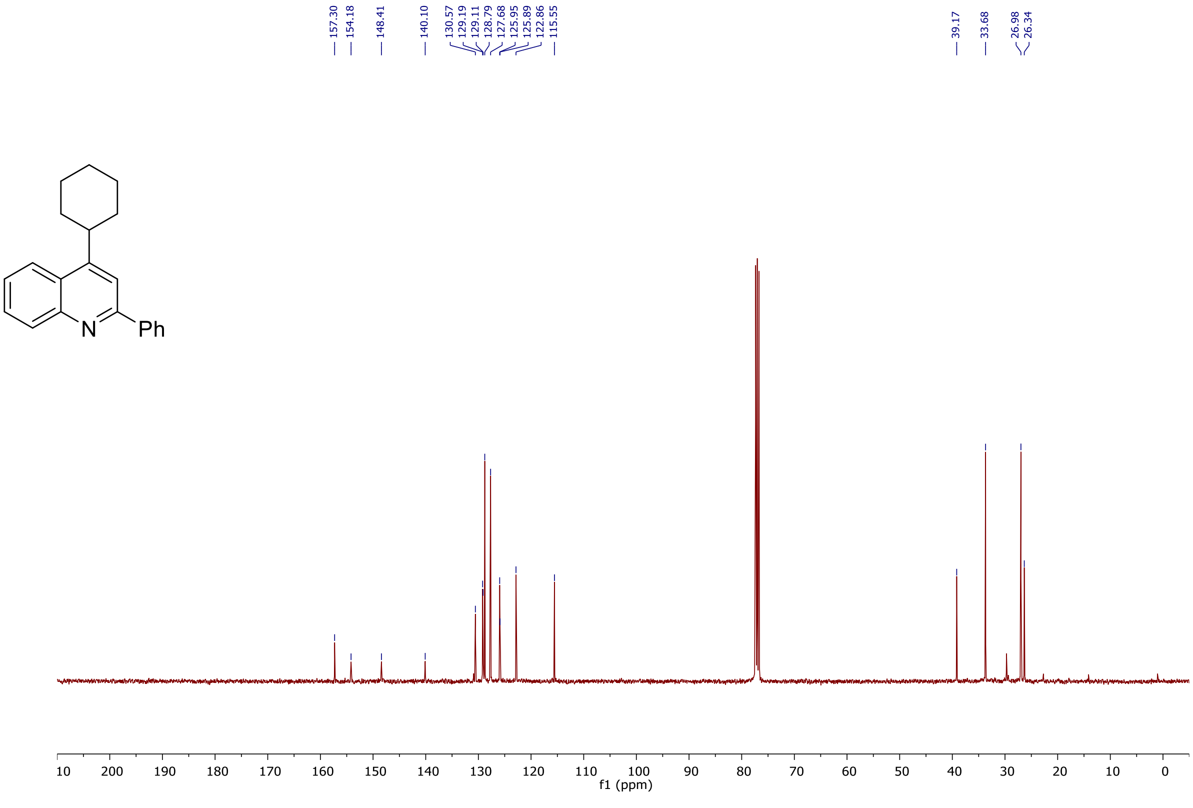

^{1}H NMR (400 MHz, $CDCl_3$) **5**

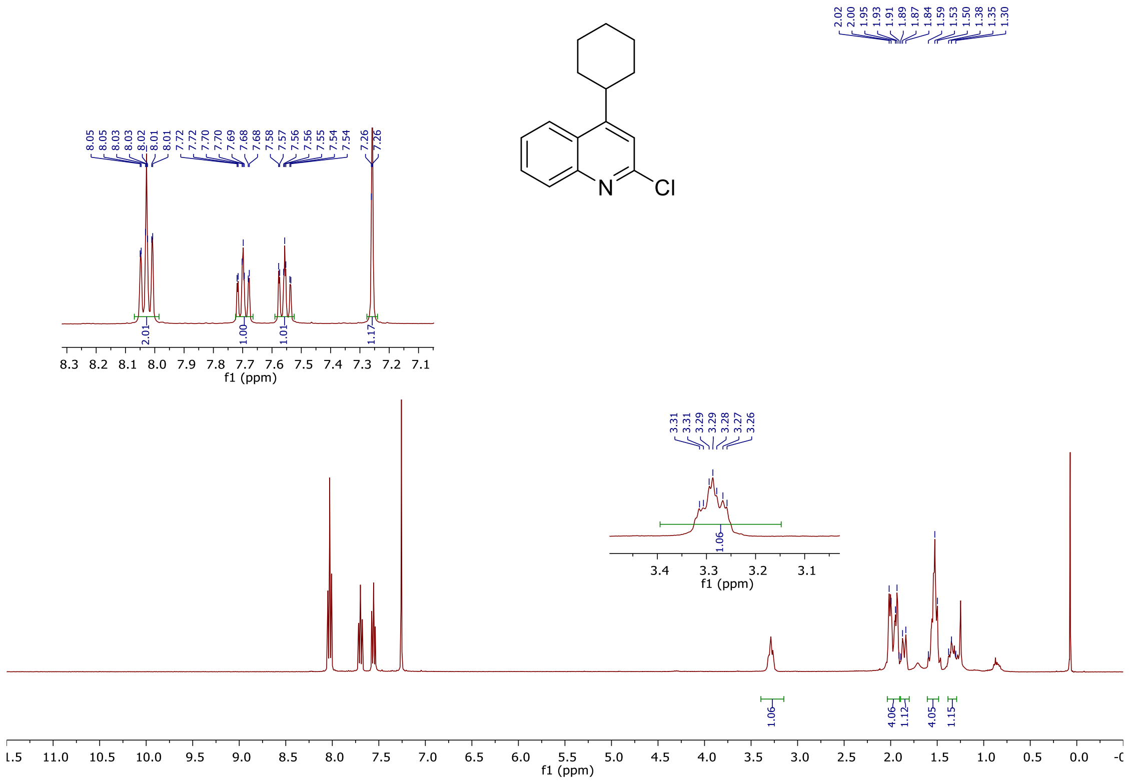

^{13}C NMR (101 MHz, $CDCl_3$) **5**

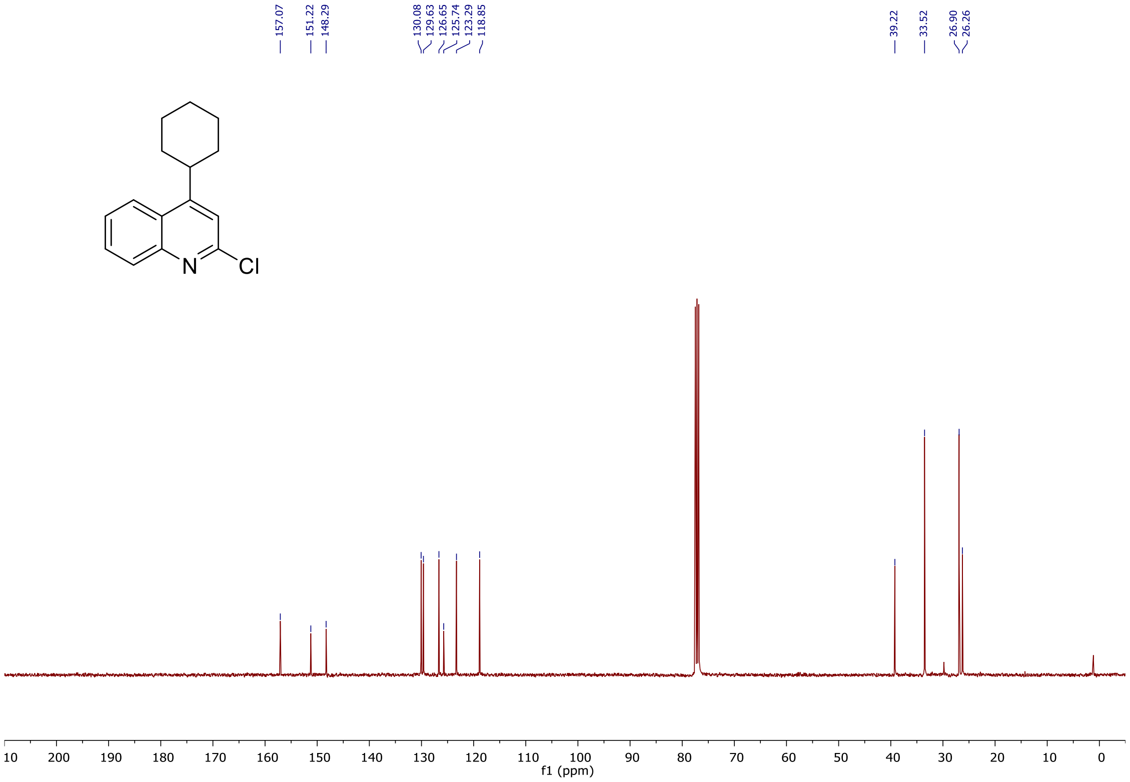

^{1}H NMR (300 MHz, $CDCl_3$) **6**

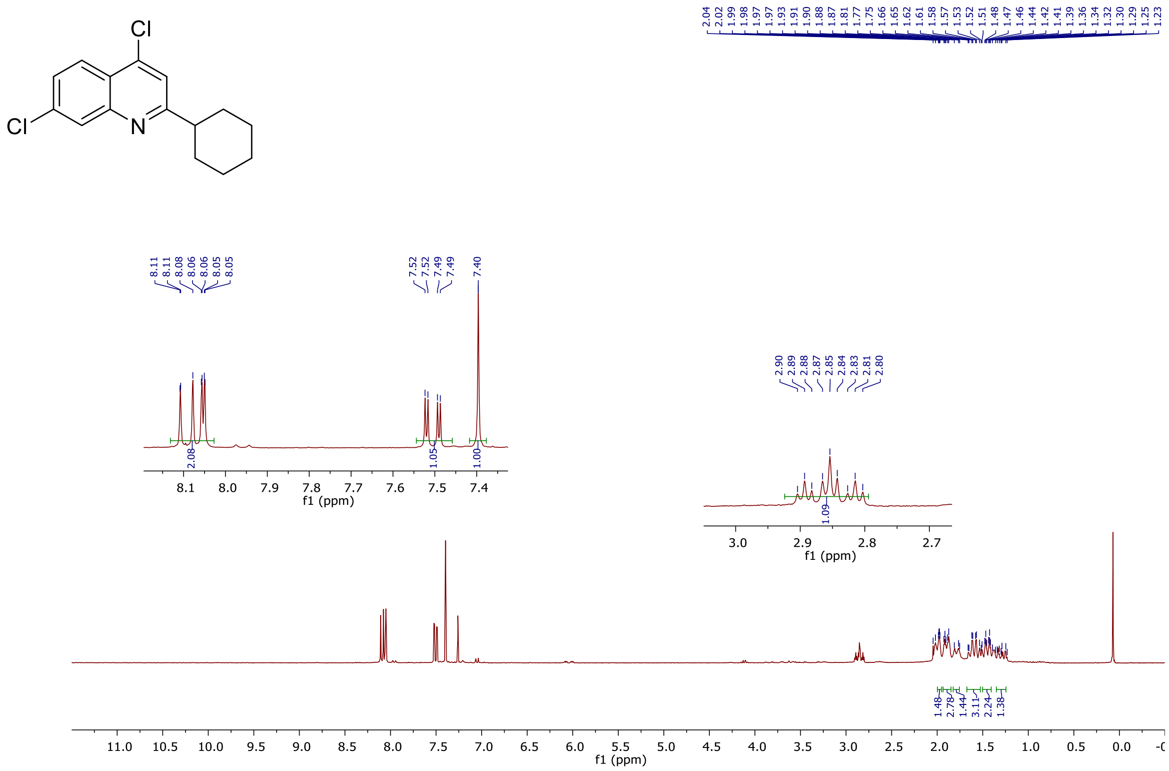

^{13}C NMR (101 MHz, $CDCl_3$) **6**

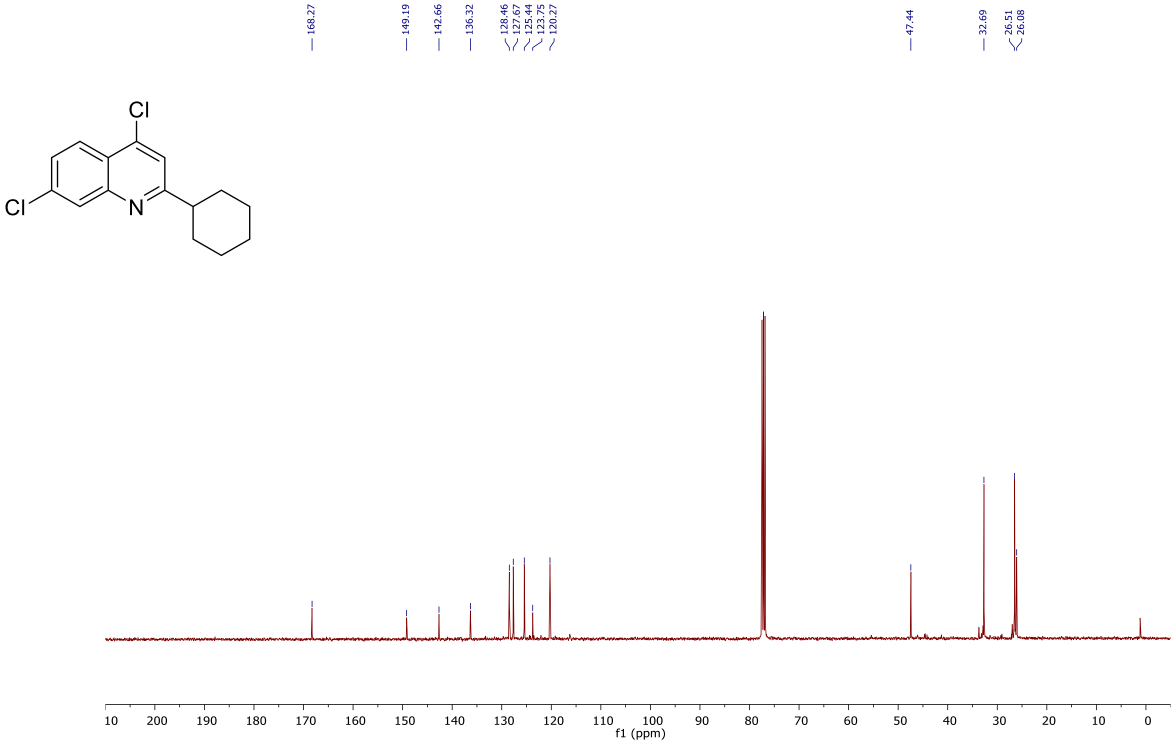

^{1}H NMR (400 MHz, $CDCl_3$) **7**

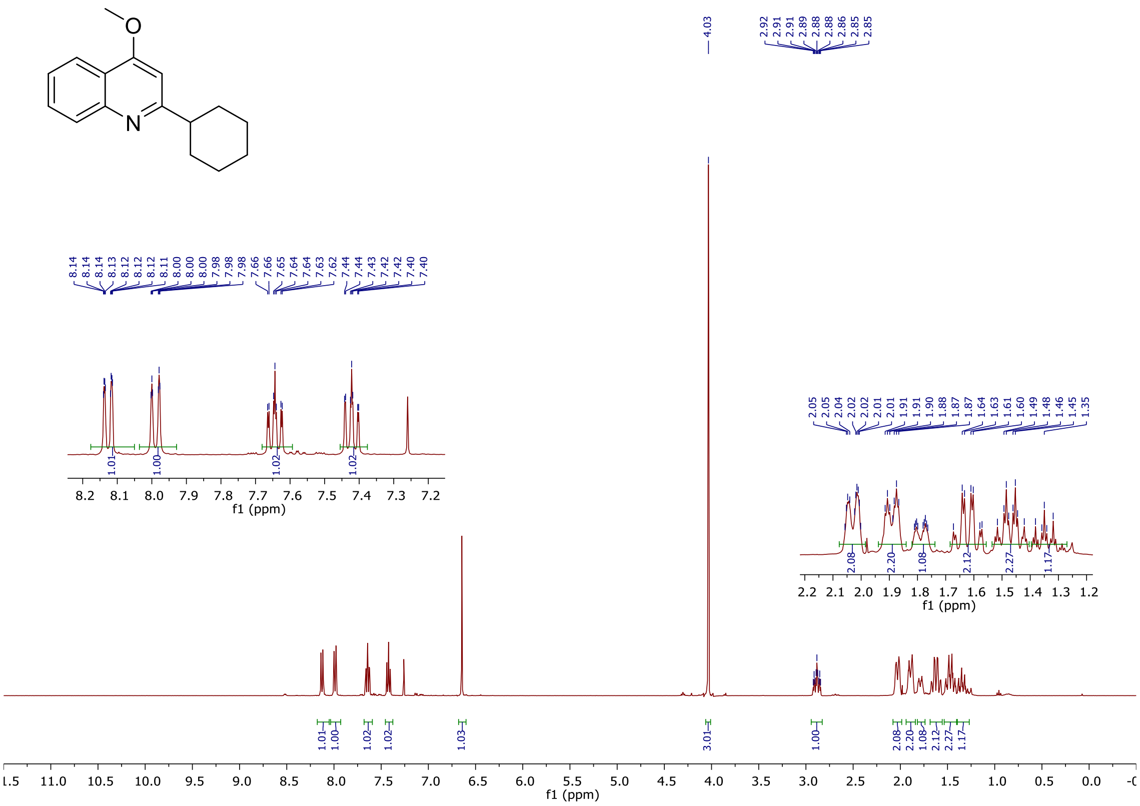

^{13}C NMR (101 MHz, $CDCl_3$) **7**

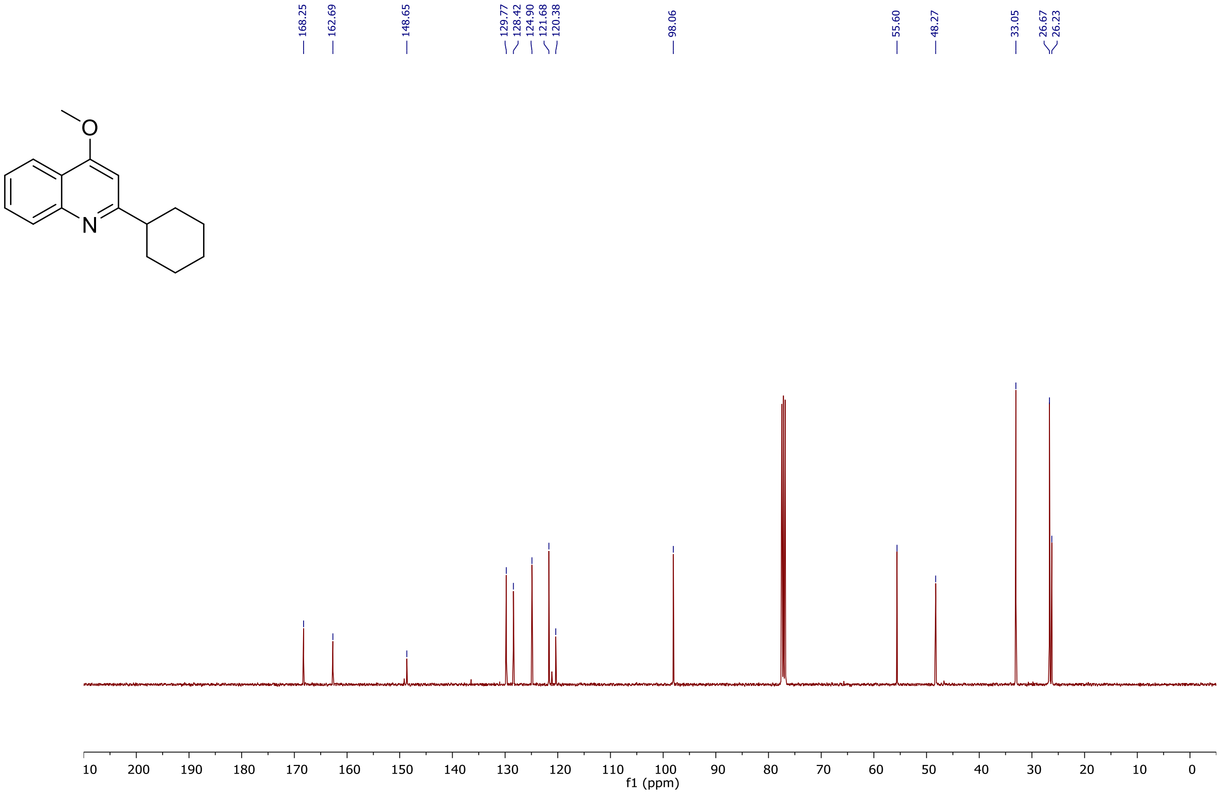

^{1}H NMR (400 MHz, $CDCl_3$) **8**

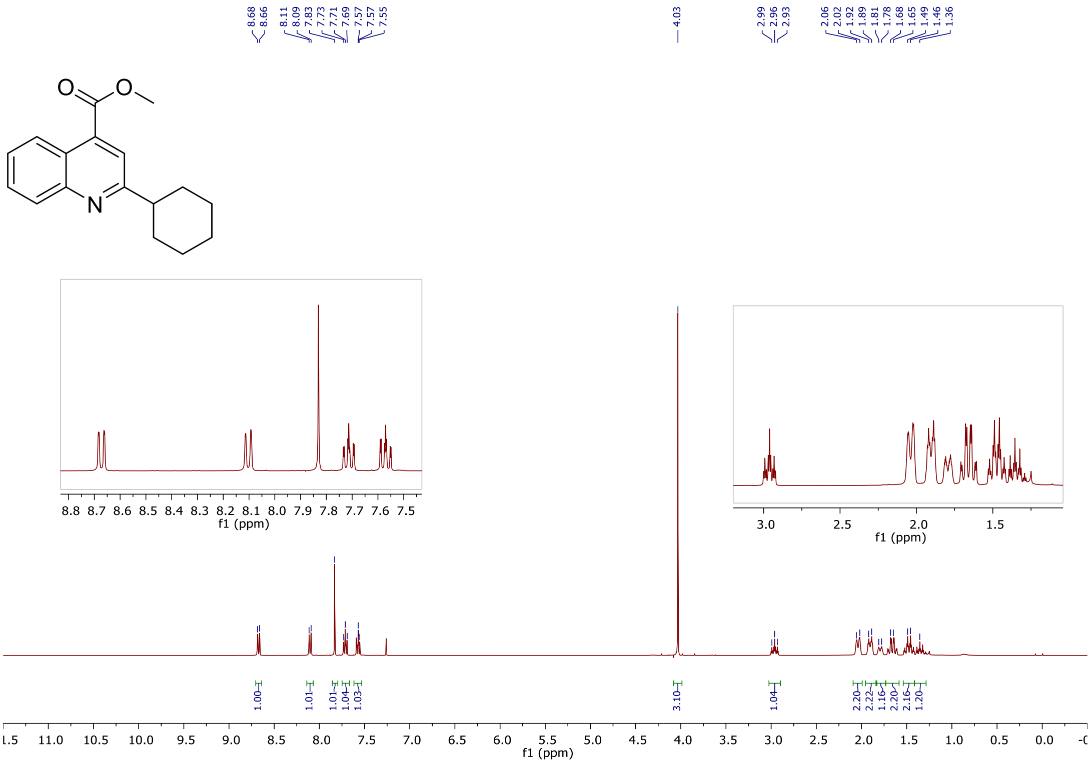

^{13}C NMR (101 MHz, $CDCl_3$) **8**

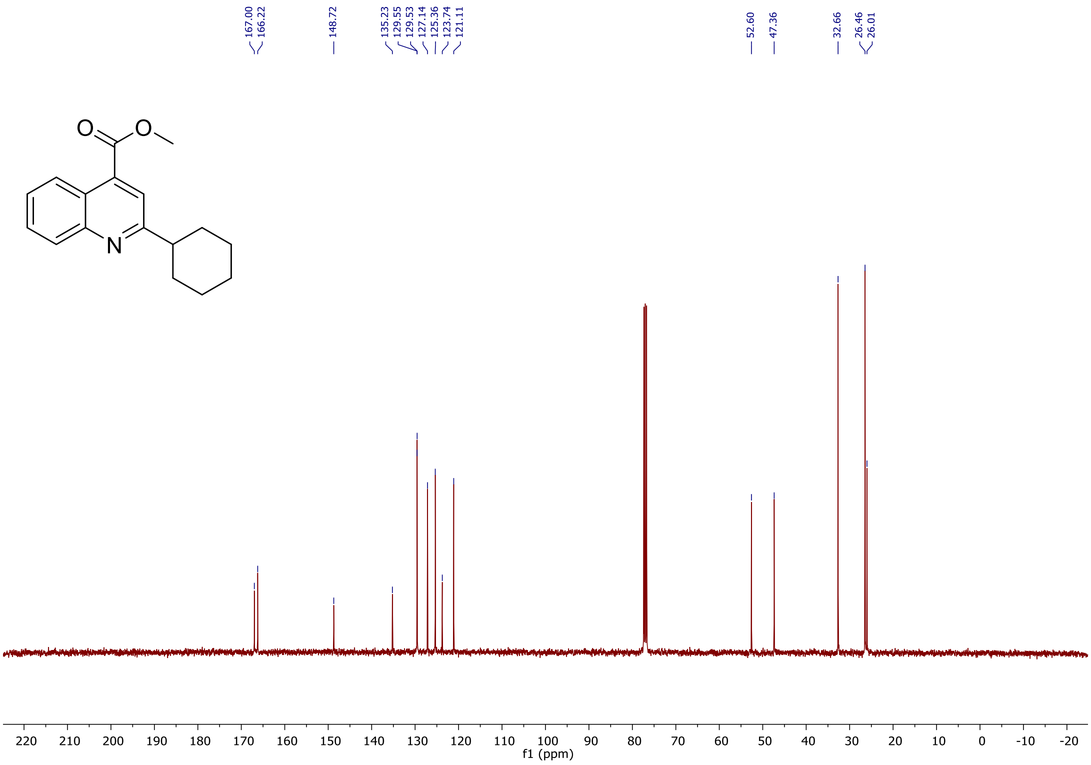

^{1}H NMR (400 MHz, $CDCl_3$) **9a+9b**

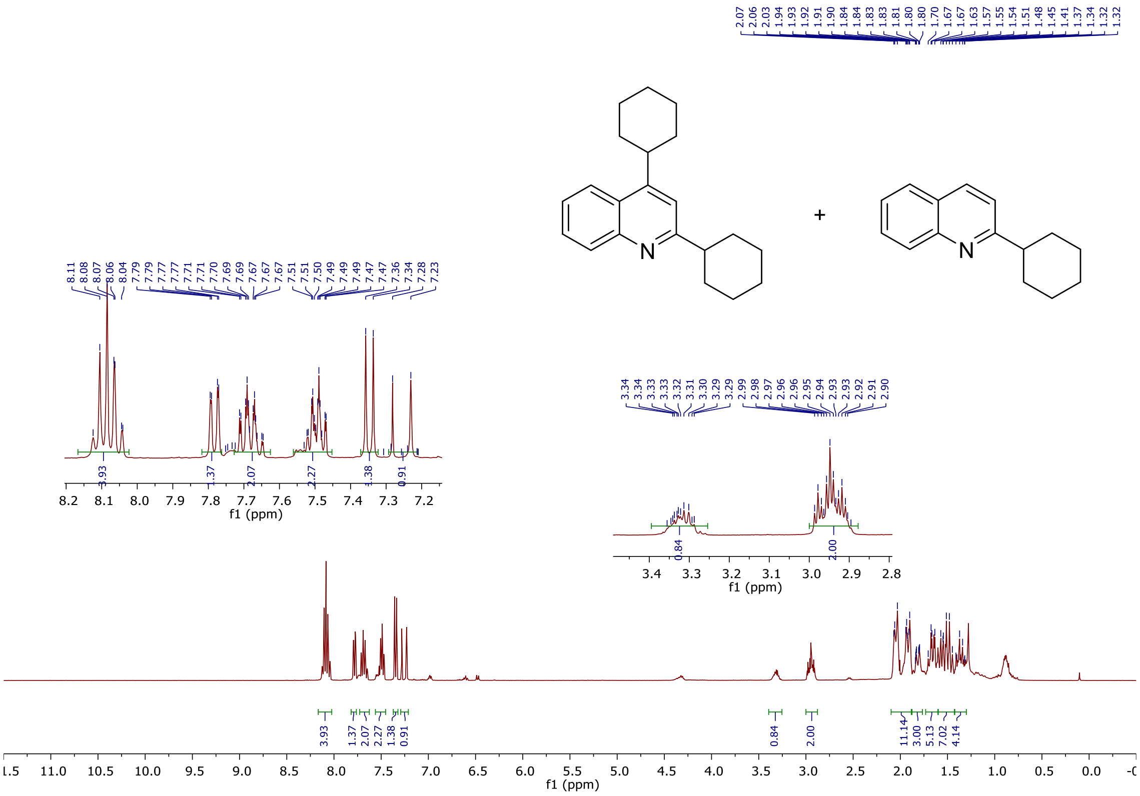

^{13}C NMR (101 MHz, $CDCl_3$) **9a+9b**

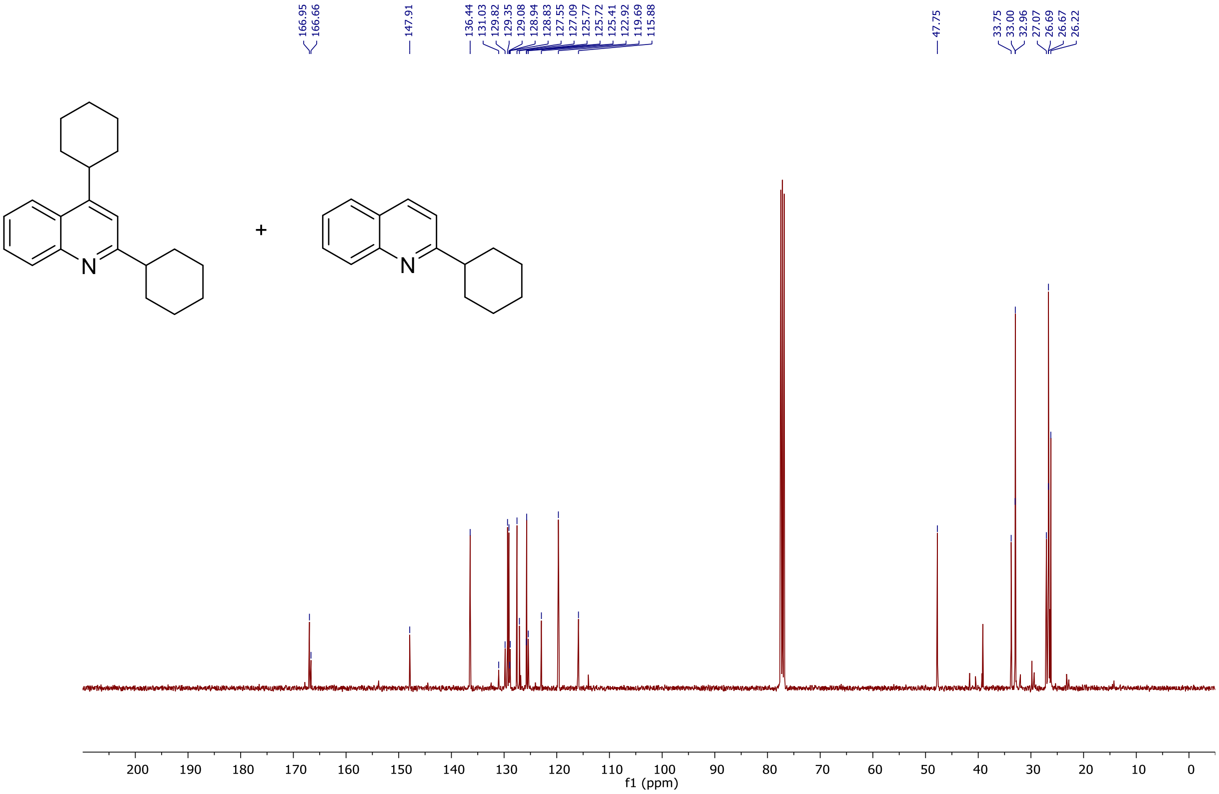

^{1}H NMR (400 MHz, $CDCl_3$) **9c**

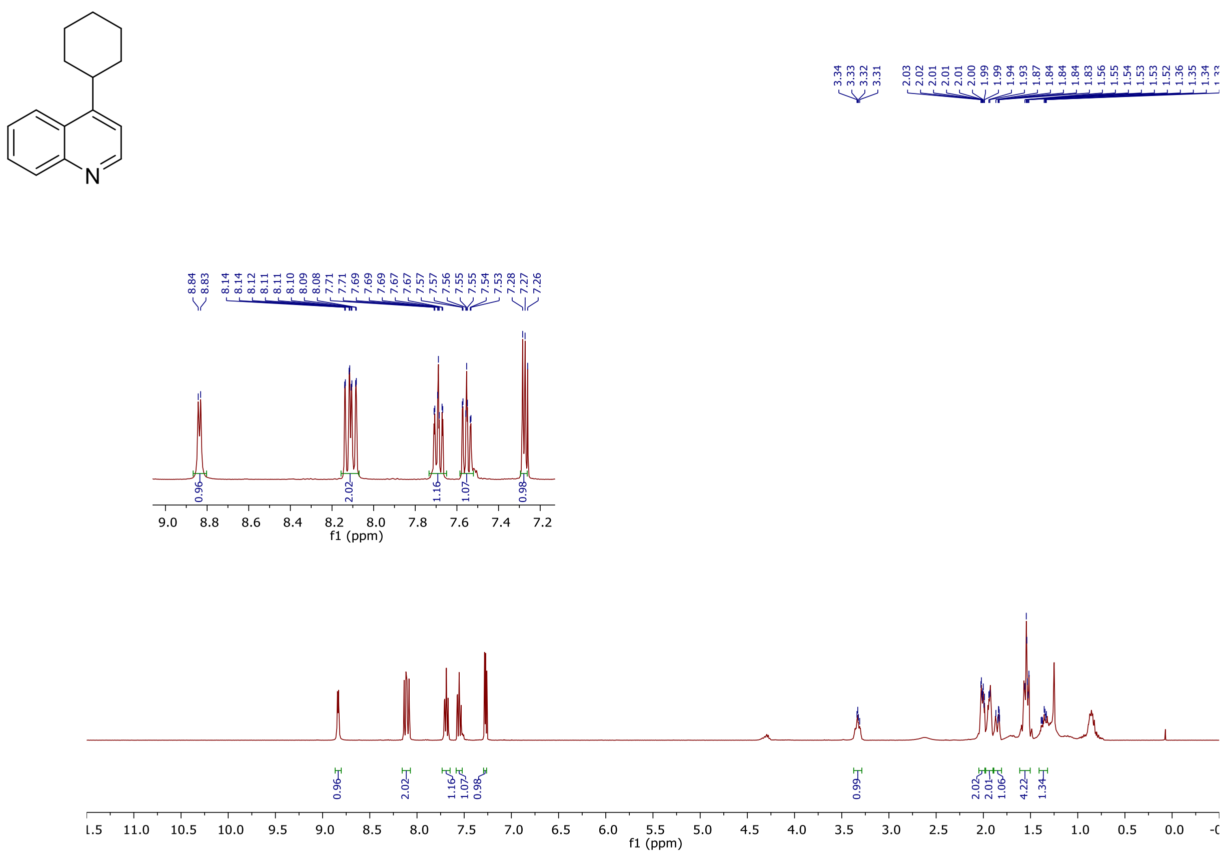

^{13}C NMR (101 MHz, $CDCl_3$) **9c**

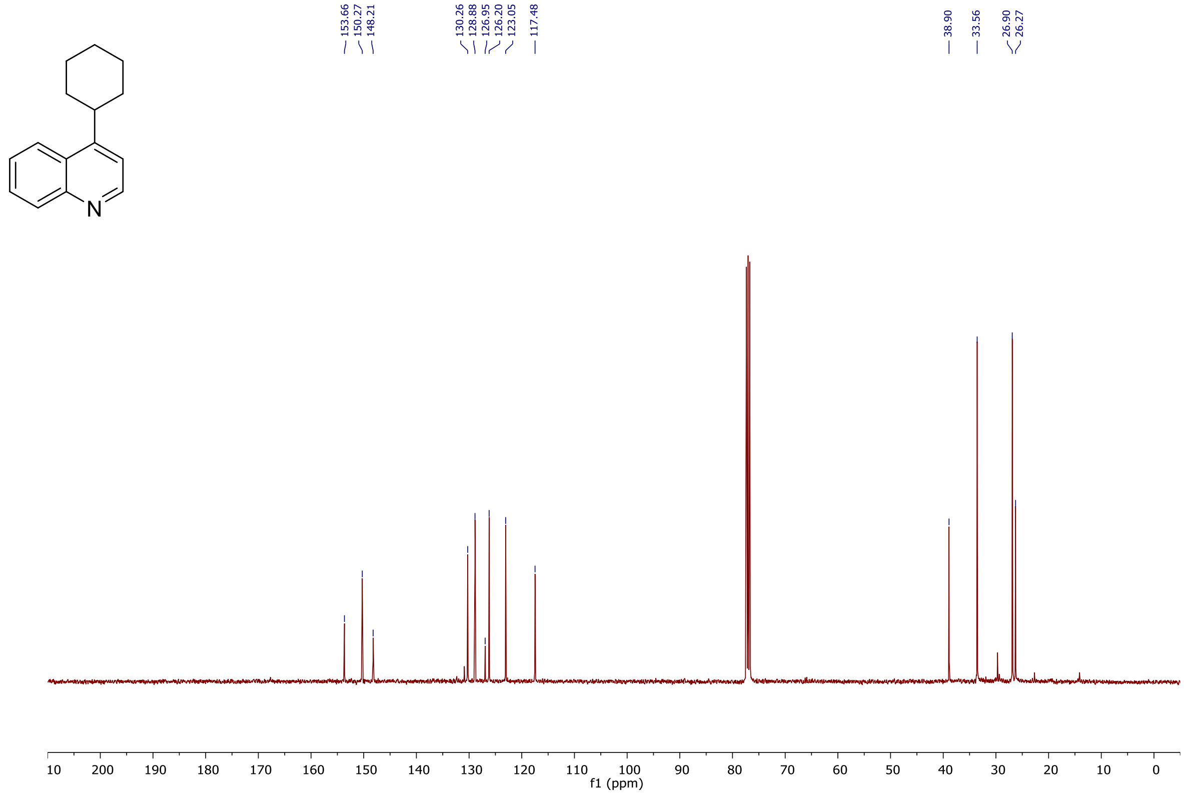

^{1}H NMR (400 MHz, $CDCl_3$) **10a + 10b**

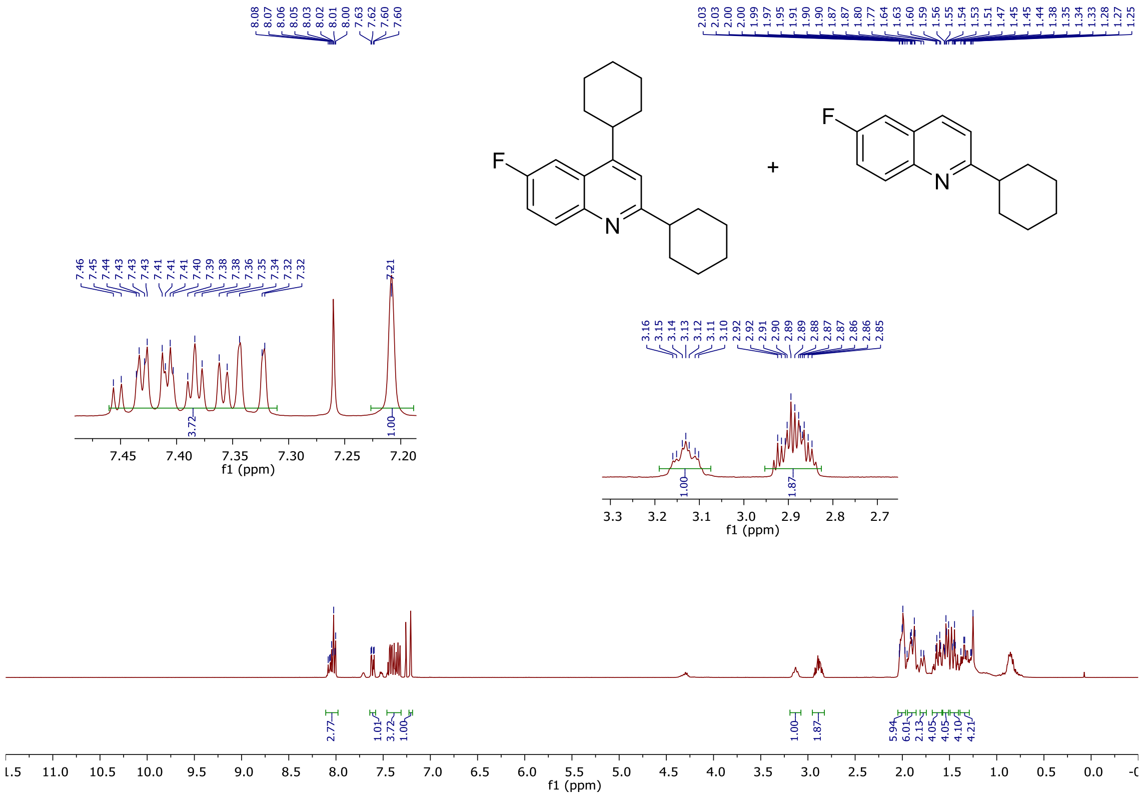

^{13}C NMR (101 MHz, $CDCl_3$) **10a + 10b**

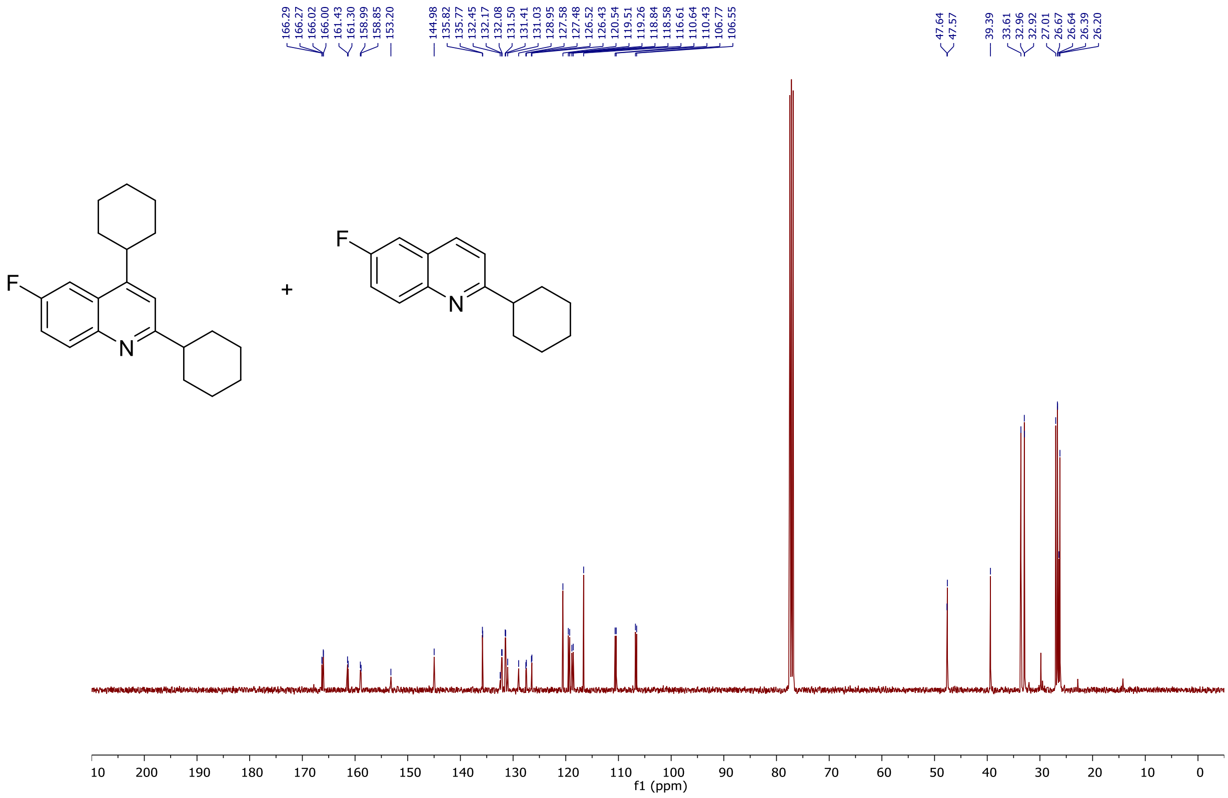

^{19}F NMR (400 MHz, $CDCl_3$) **10a + 10b**

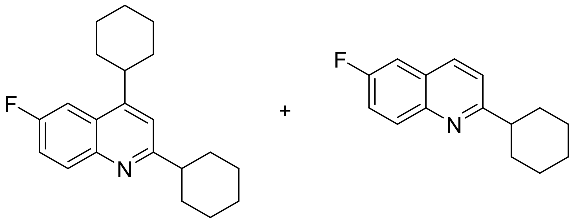

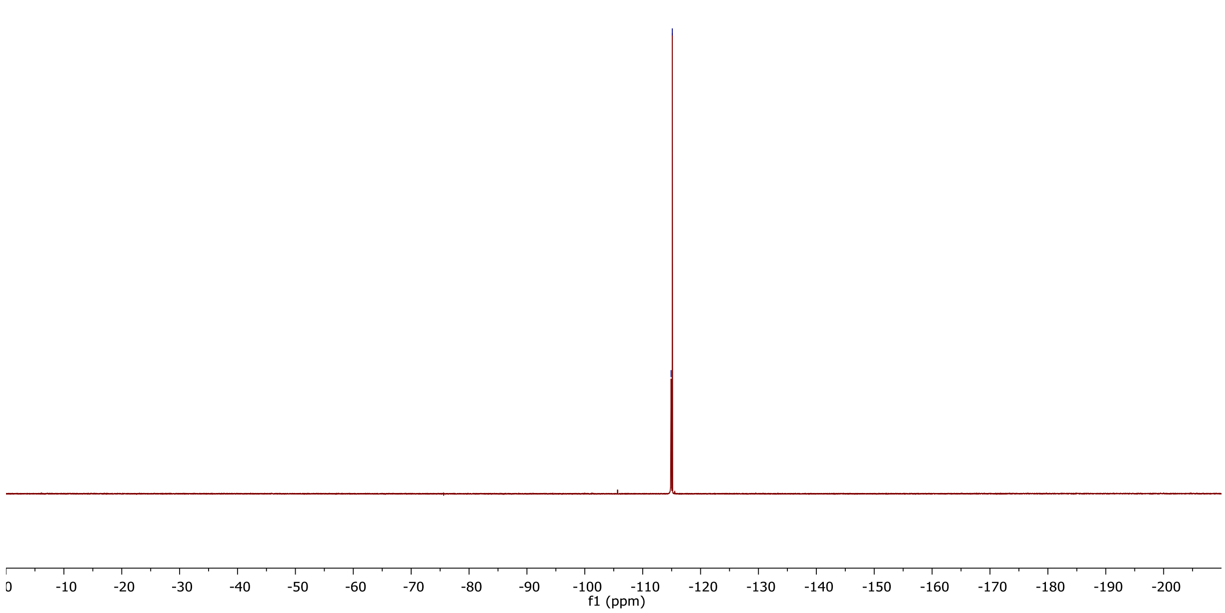

^{1}H NMR (400 MHz, $CDCl_3$) **10c**

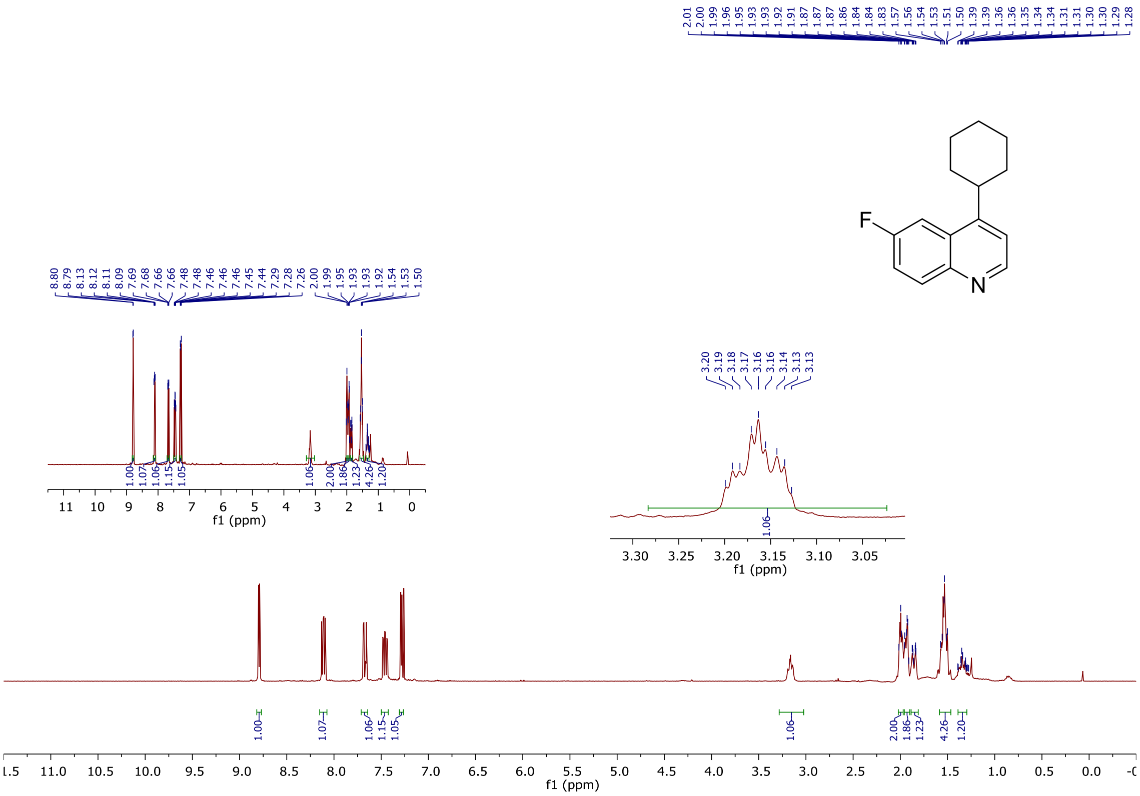

^{13}C NMR (101 MHz, $CDCl_3$) **10c**

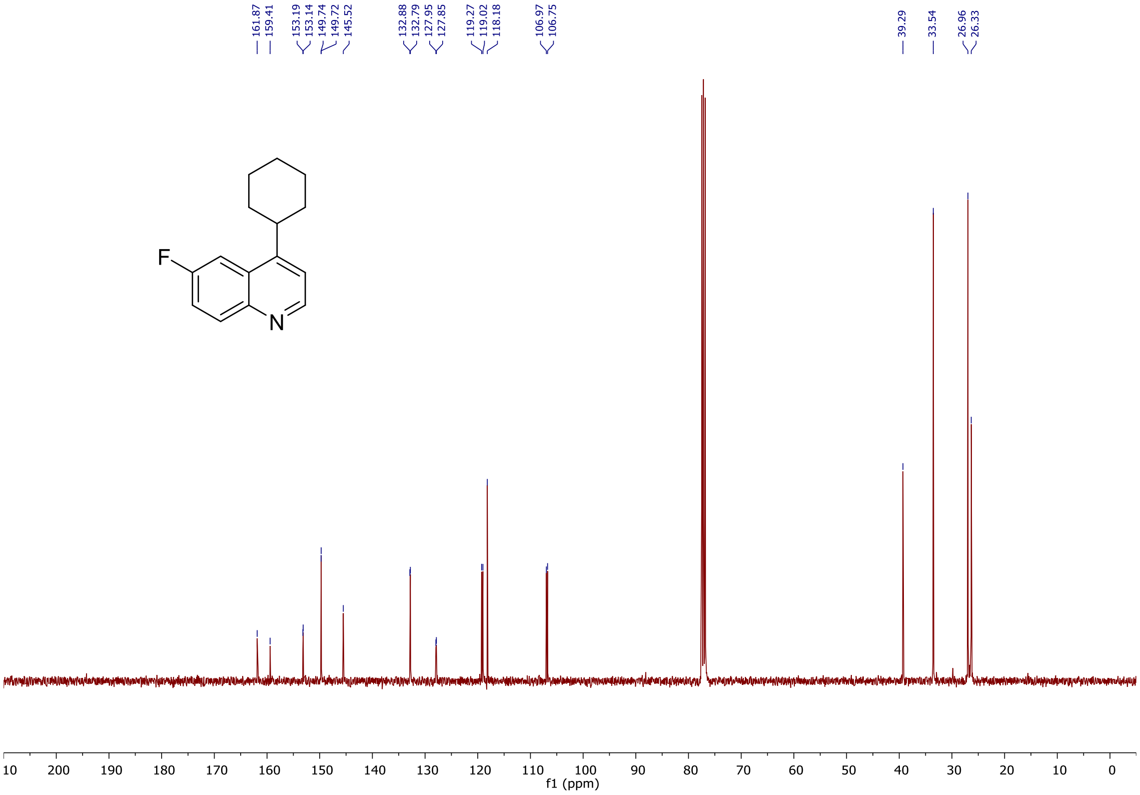

^{19}F NMR (400 MHz, $CDCl_3$) **10c**

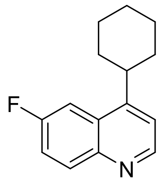

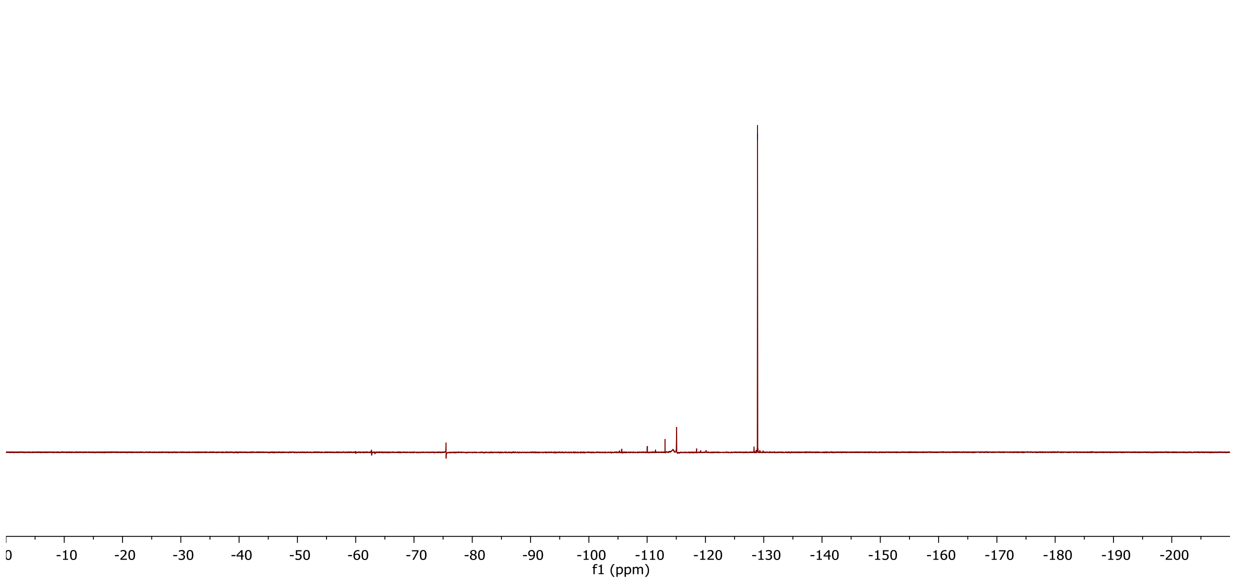

^{1}H NMR (400 MHz, $CDCl_3$) **11**

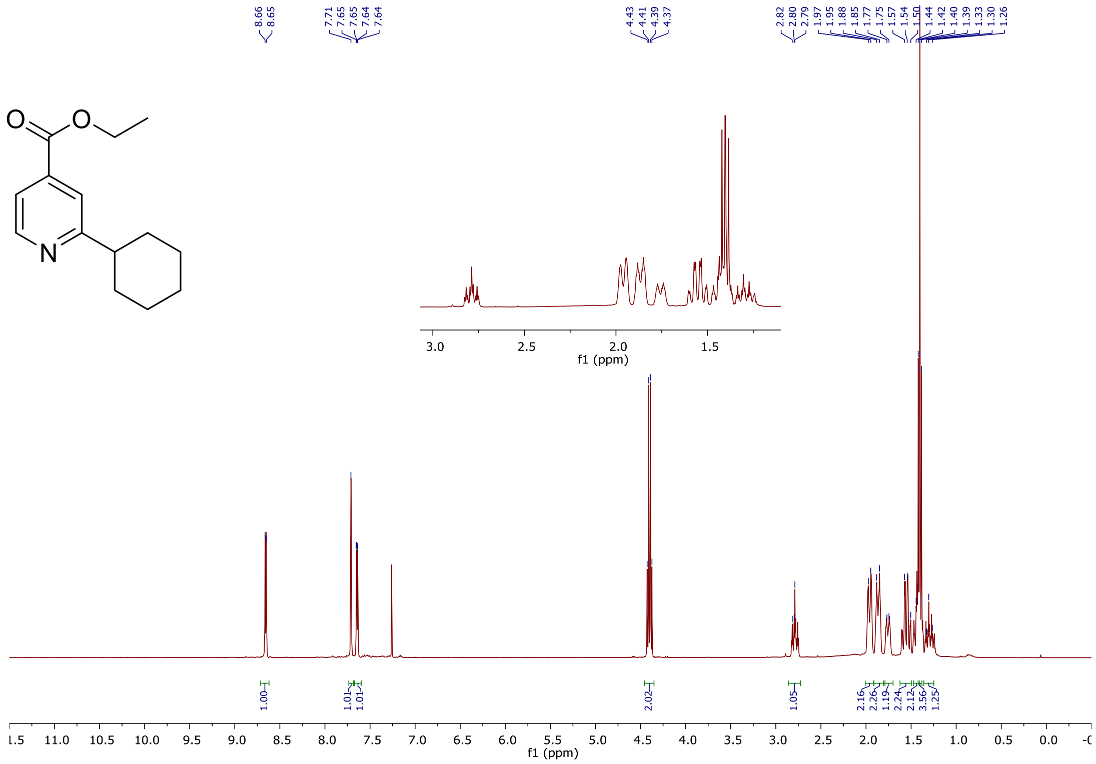

^{13}C NMR (101 MHz, $CDCl_3$) **11**

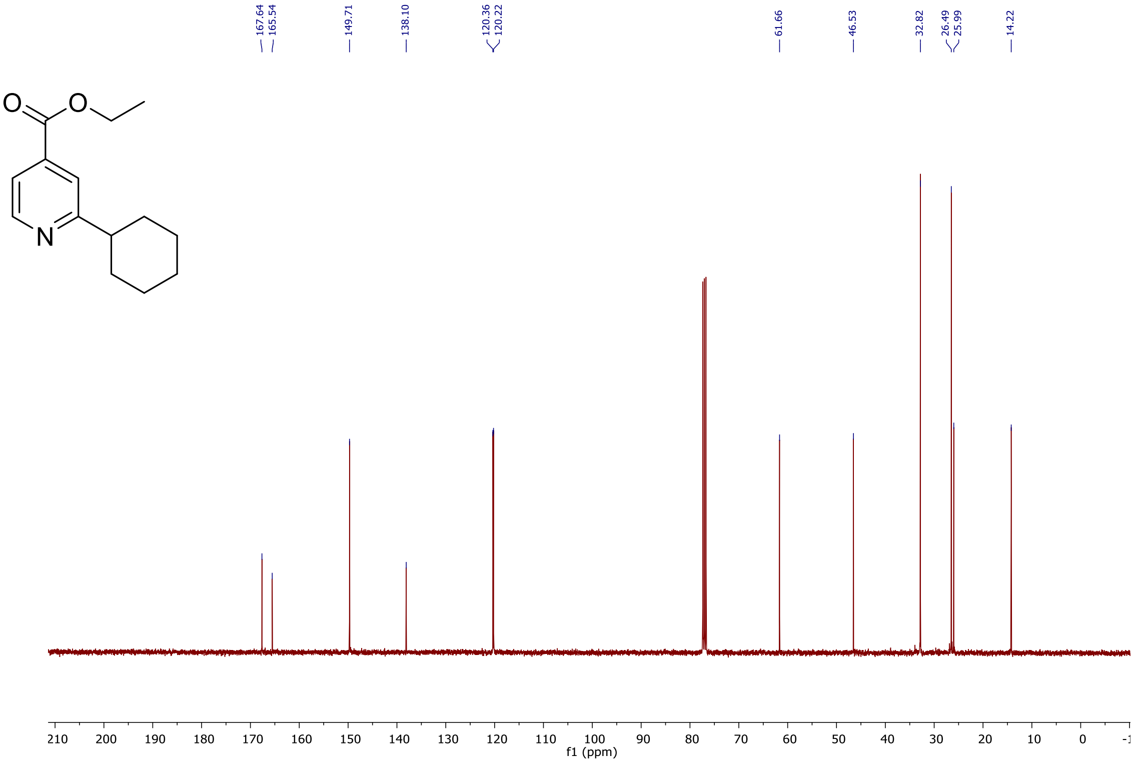

^{1}H NMR (400 MHz, $CDCl_3$) **12a**

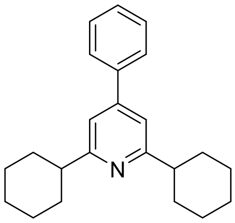

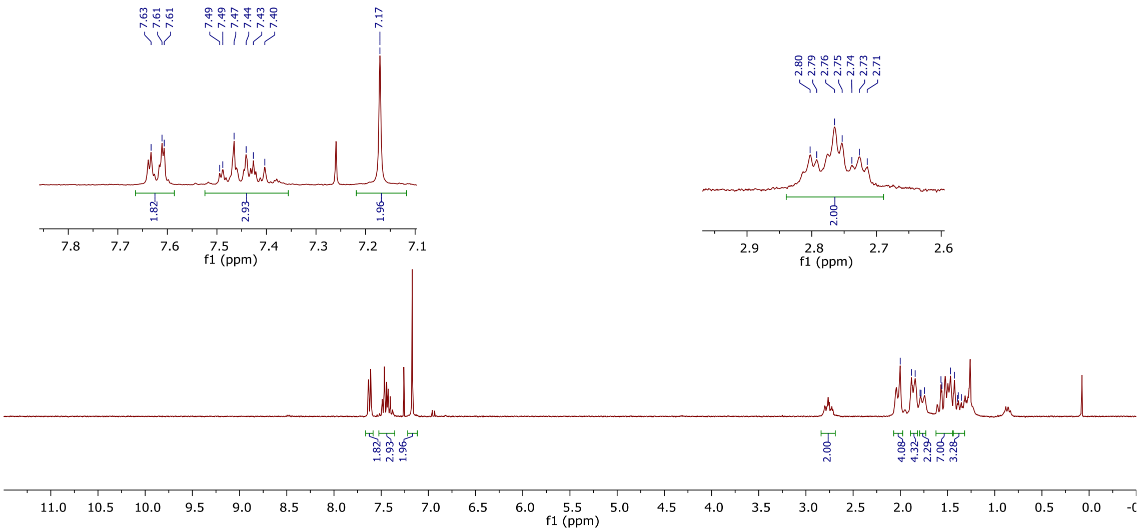

^{13}C NMR (101 MHz, $CDCl_3$) **12a**

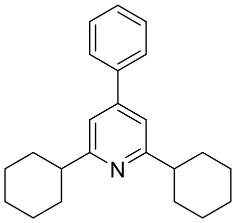

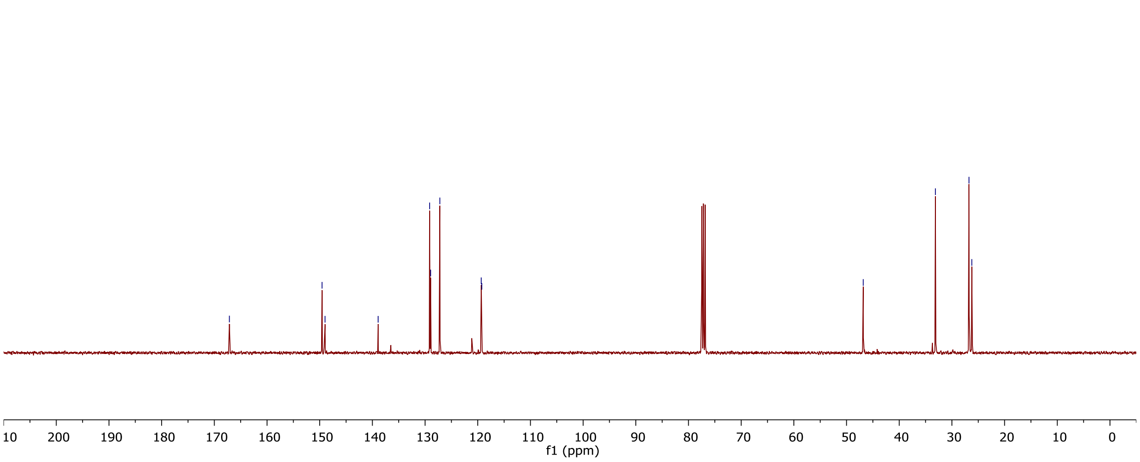

^{1}H NMR (400 MHz, $CDCl_3$) **12b**

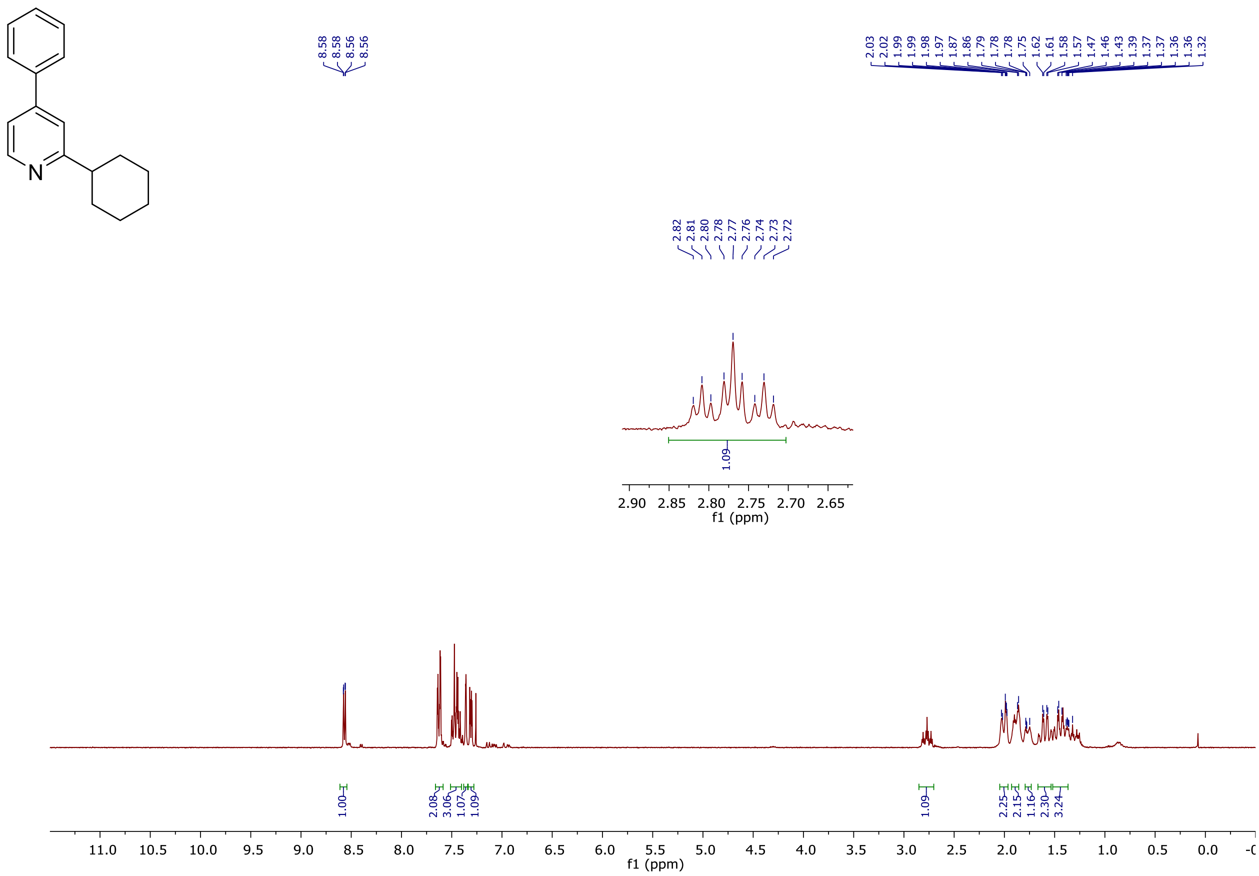

^{13}C NMR (101 MHz, $CDCl_3$) **12b**

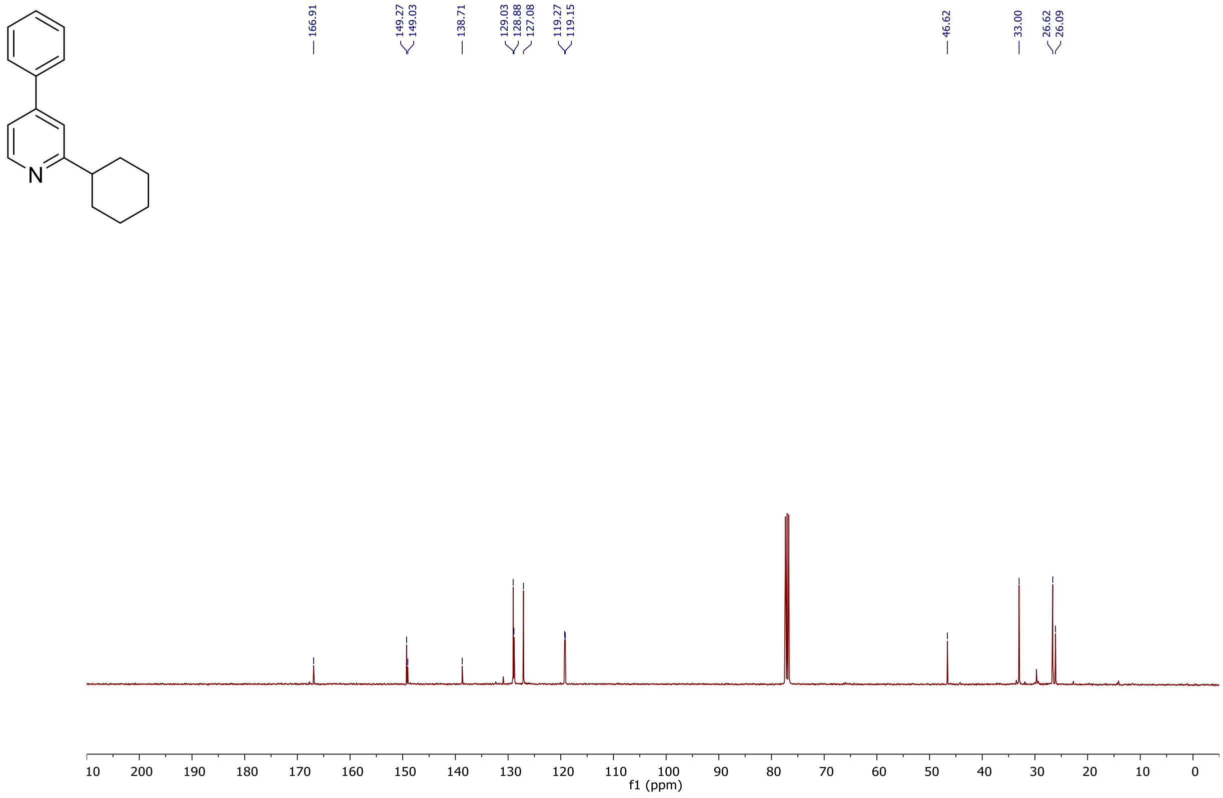

^{1}H NMR (300 MHz, $CDCl_3$) **13**

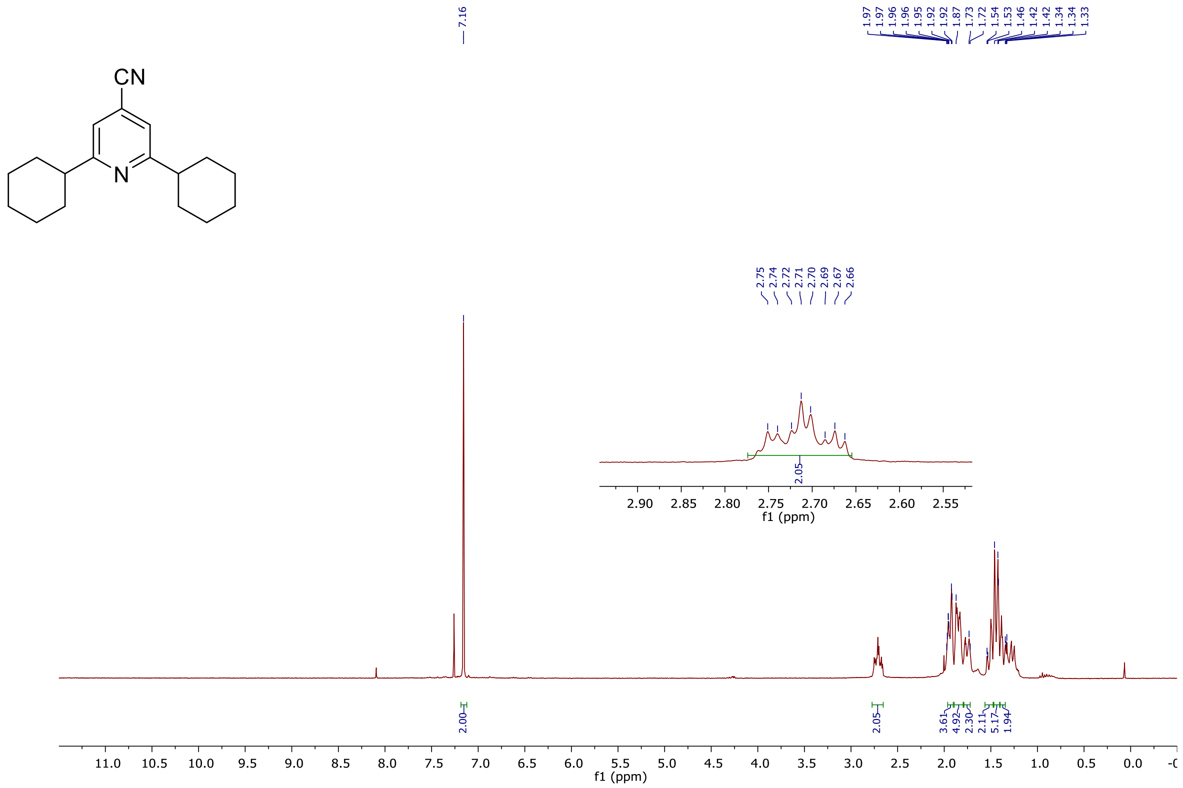

^{13}C NMR (101 MHz, $CDCl_3$) **13**

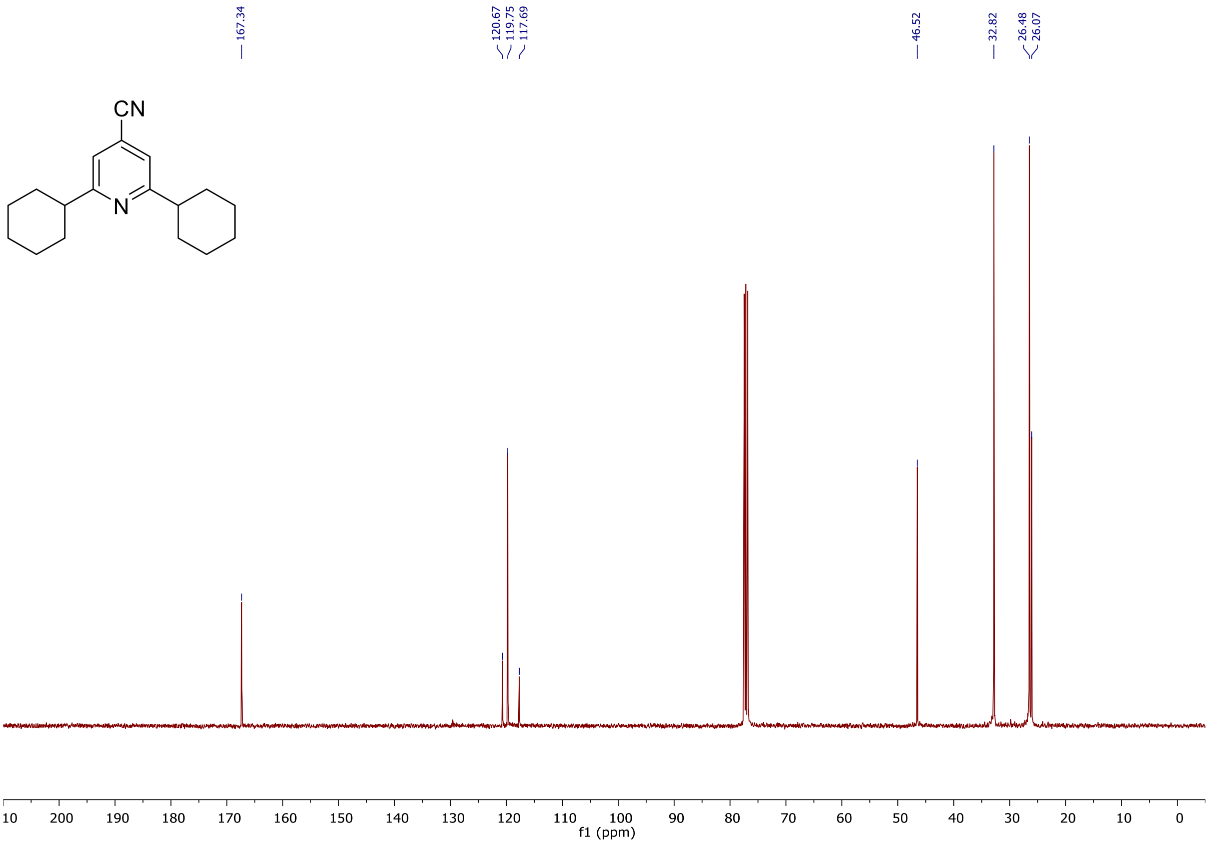

^{1}H NMR (400 MHz, $CDCl_3$) **14**

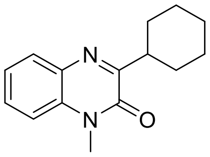

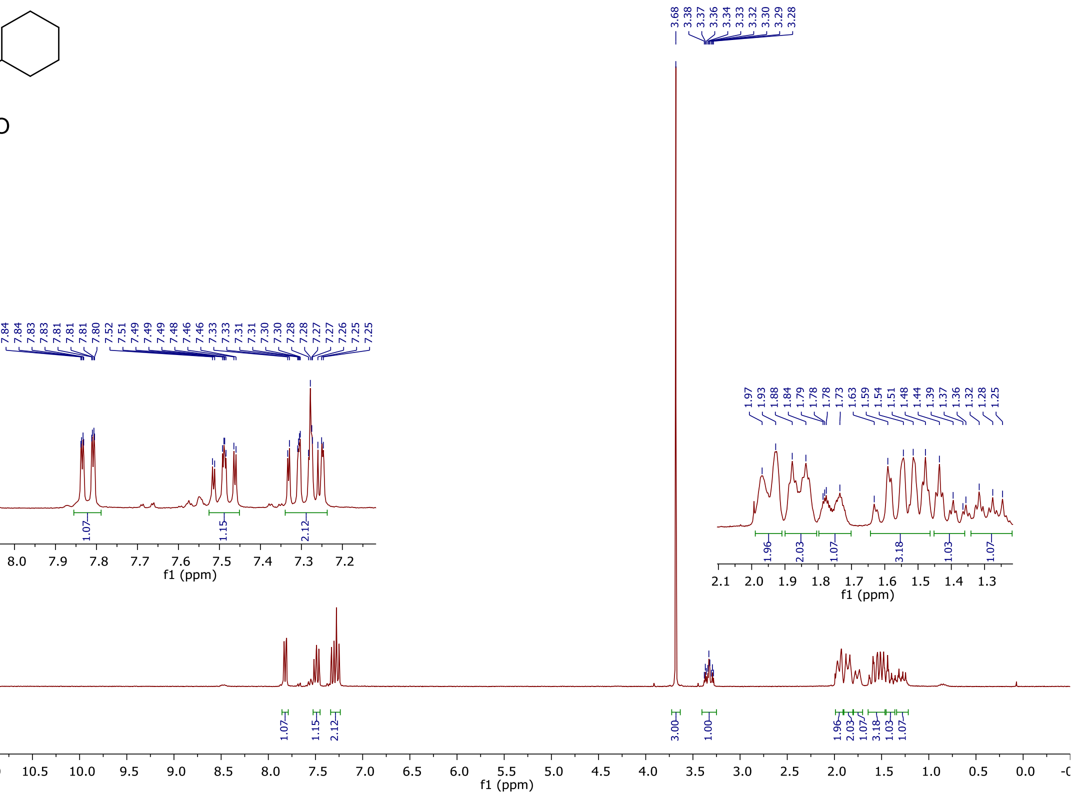

^{13}C NMR (101 MHz, $CDCl_3$) **14**

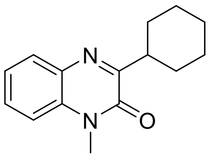

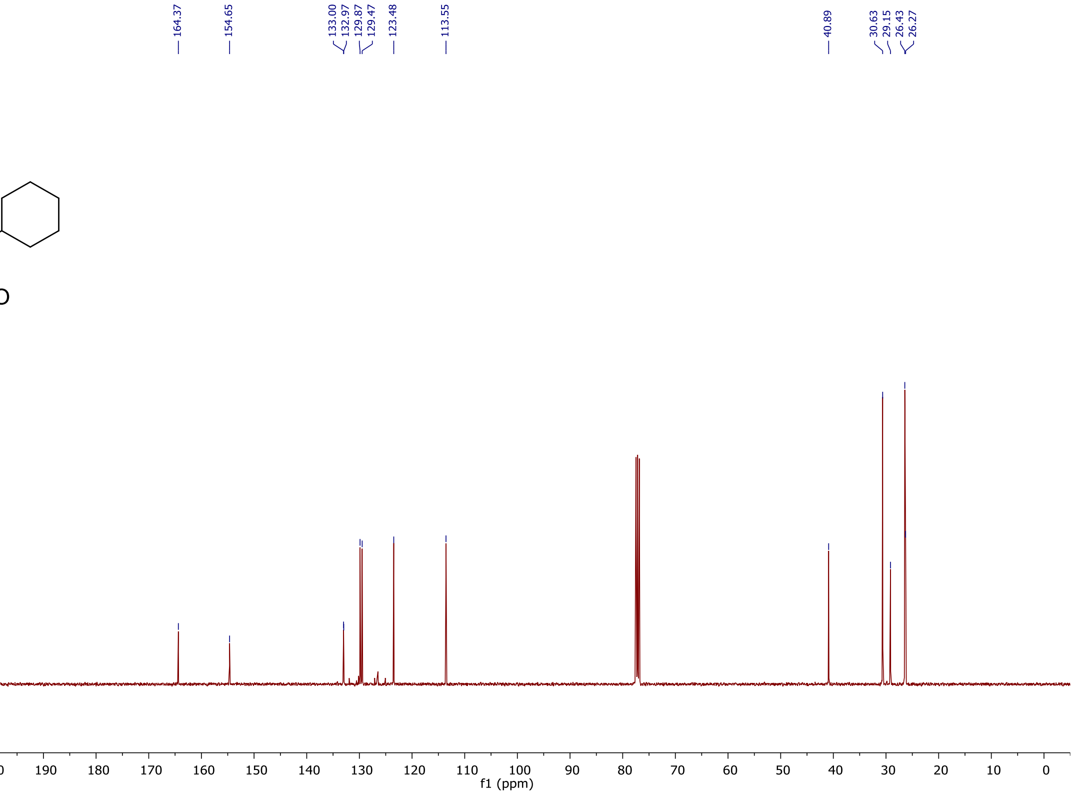

^{1}H NMR (500 MHz, DMSO-d_6) **15**

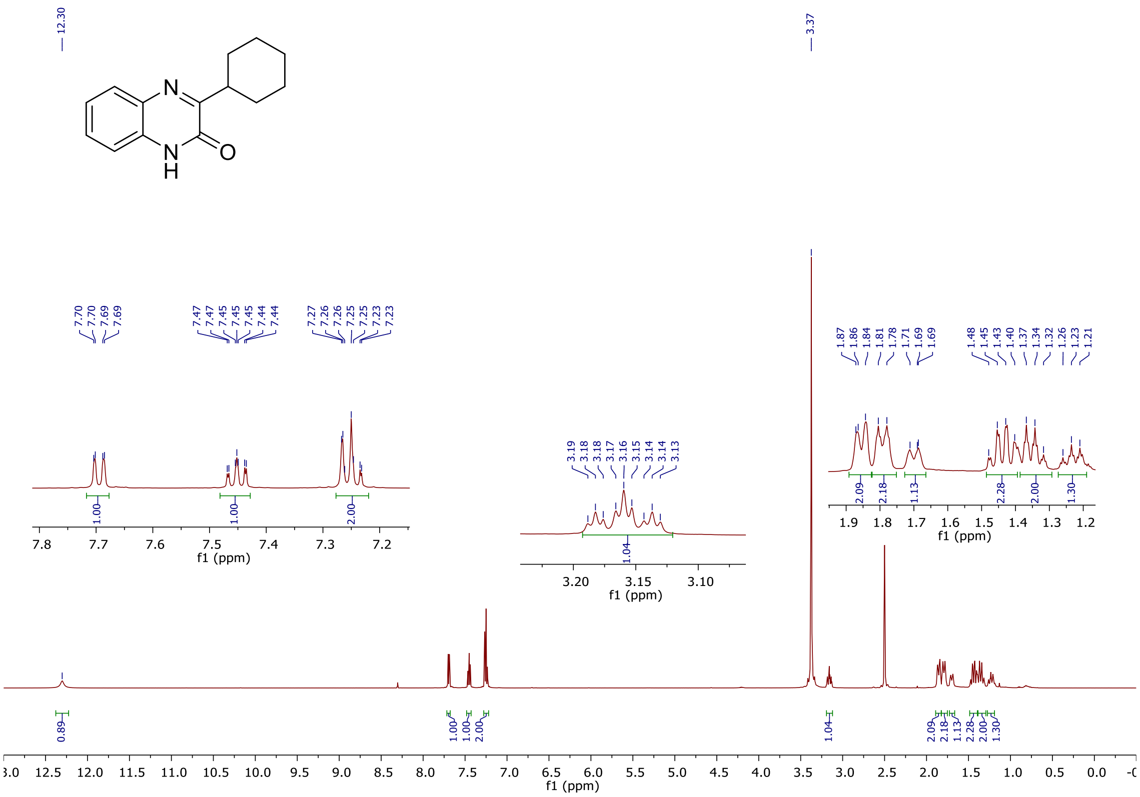

^{13}C NMR (126 MHz, DMSO-d_6) **15**

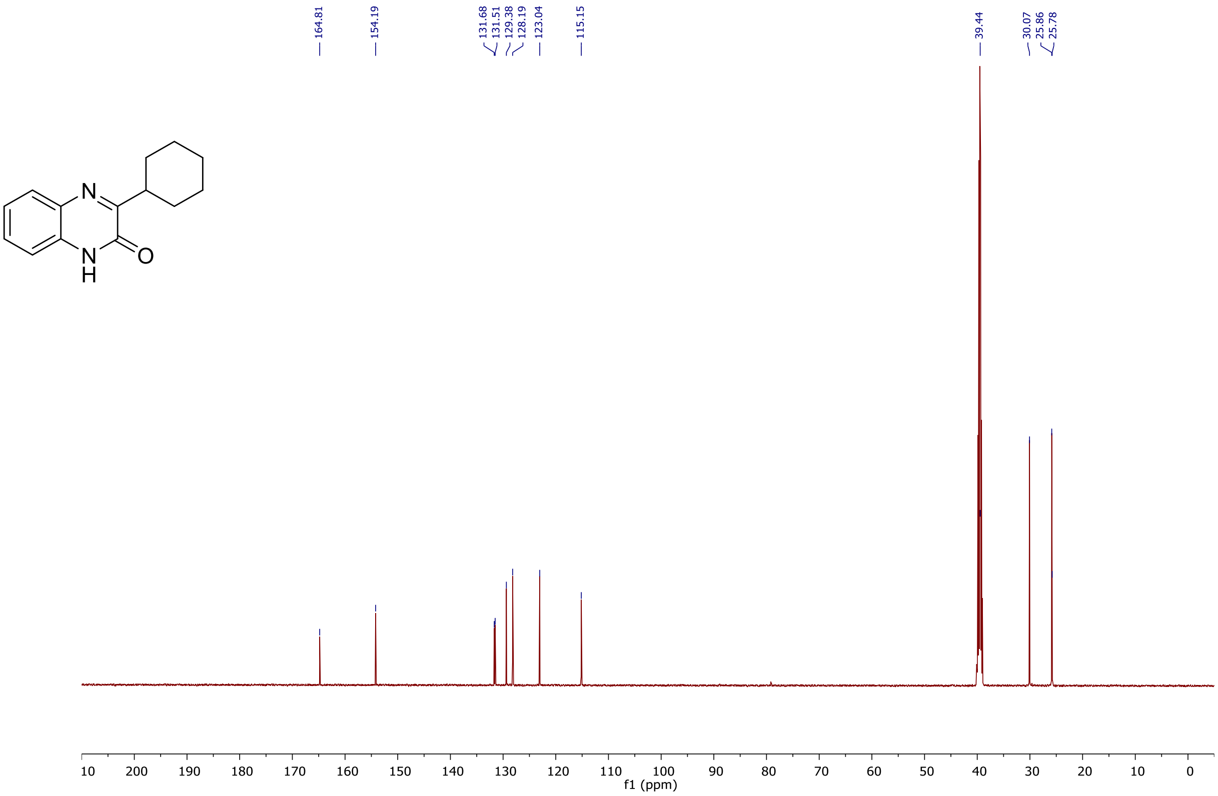

^{1}H NMR (400 MHz, $CDCl_3$) **16**

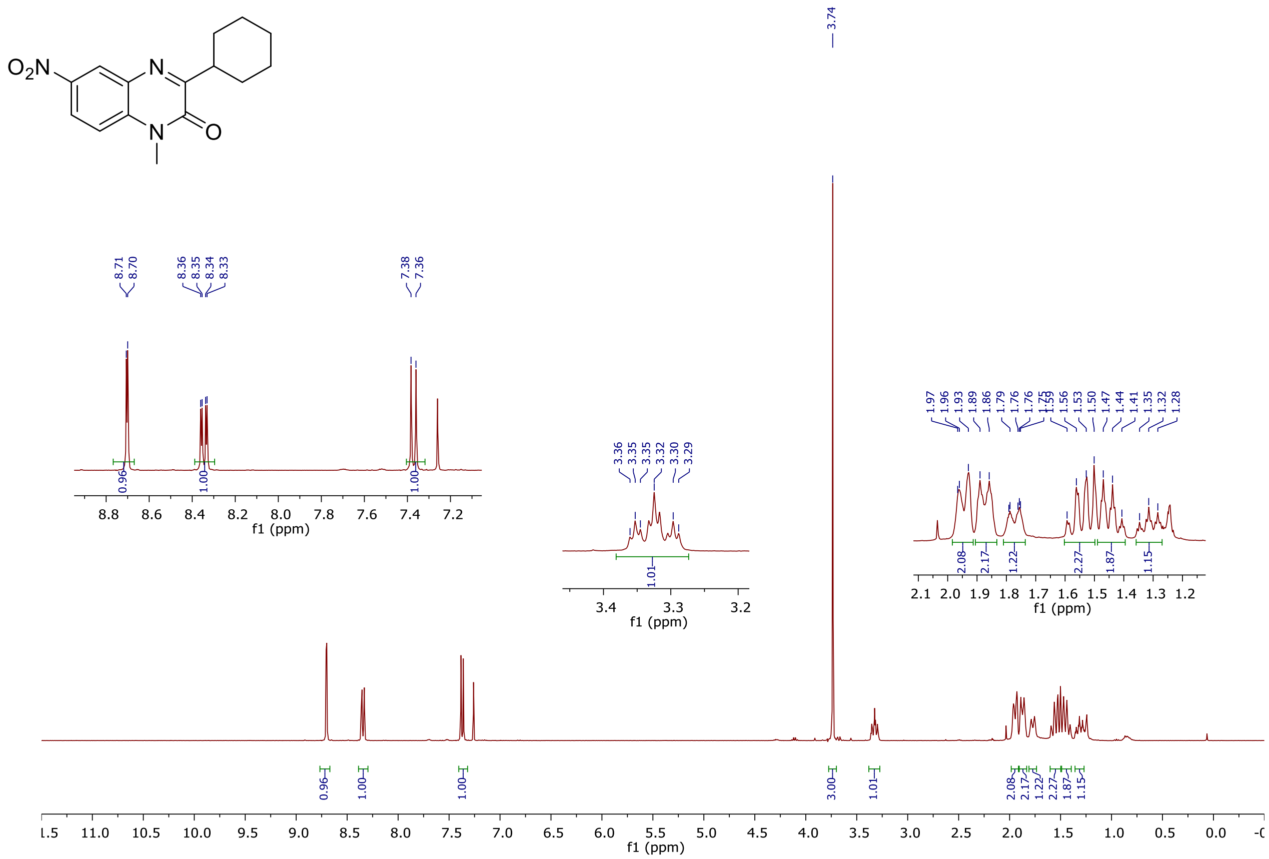

^{13}C NMR (101 MHz, $CDCl_3$) **16**

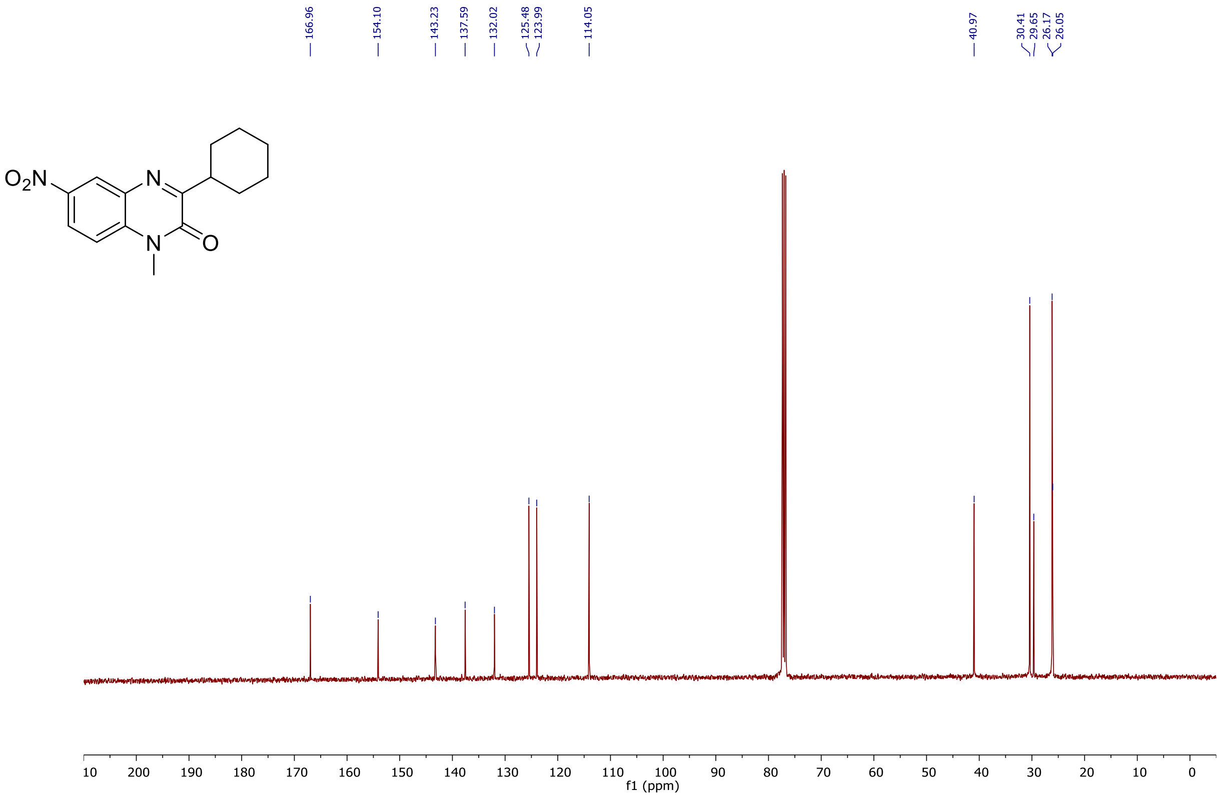

^{1}H NMR (300 MHz, $CDCl_3$) **17**

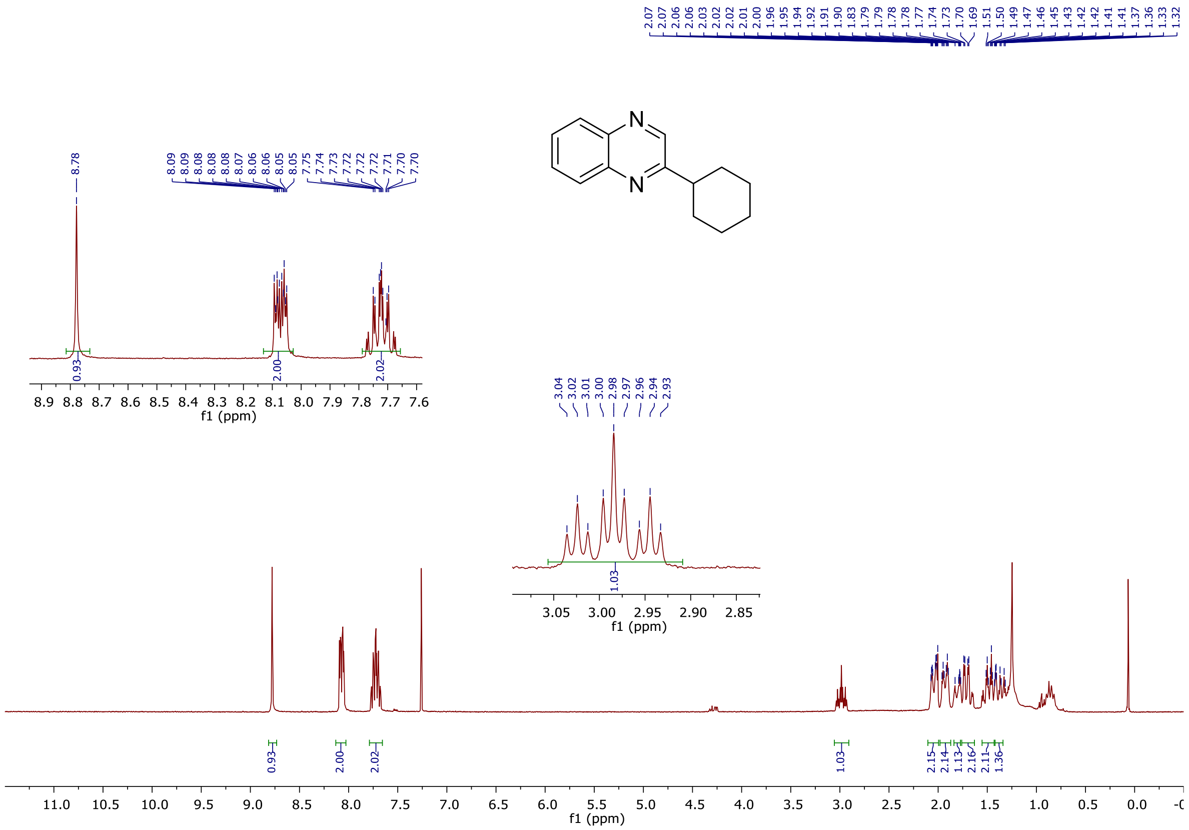

^{13}C NMR (101 MHz, $CDCl_3$) **17**

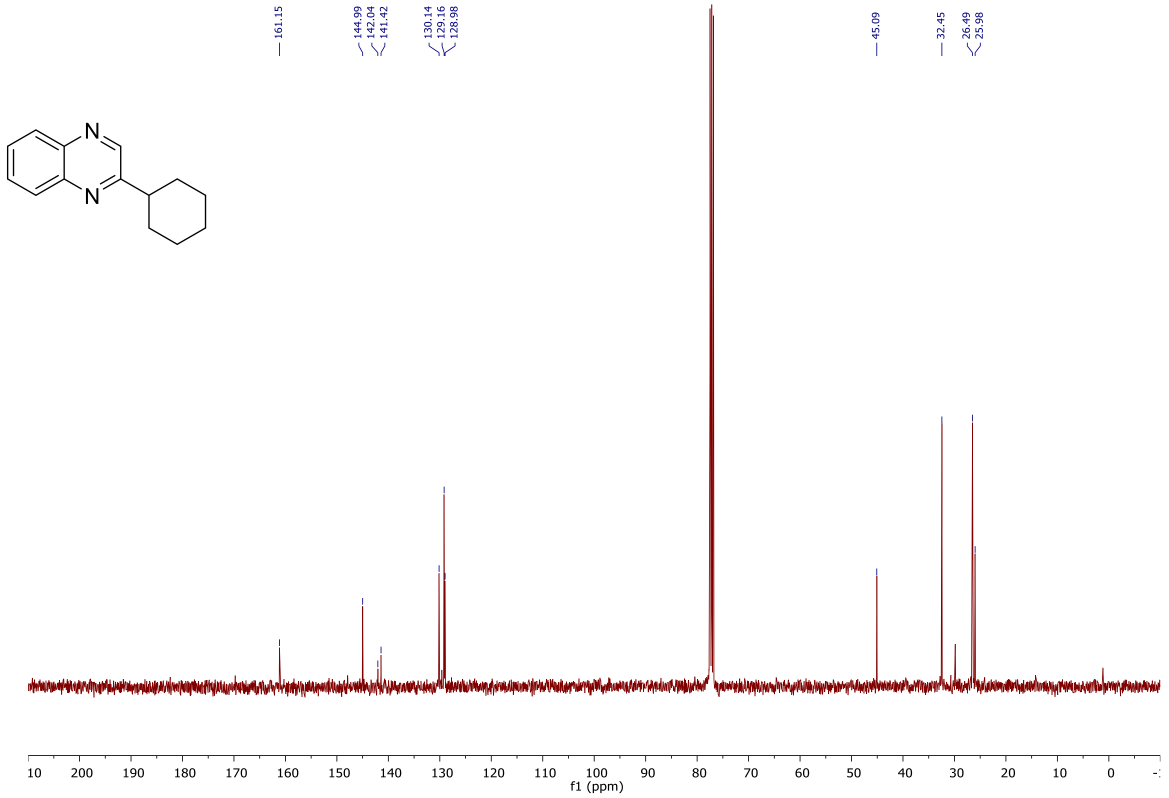

^{1}H NMR (300 MHz, $CDCl_3$) **18**

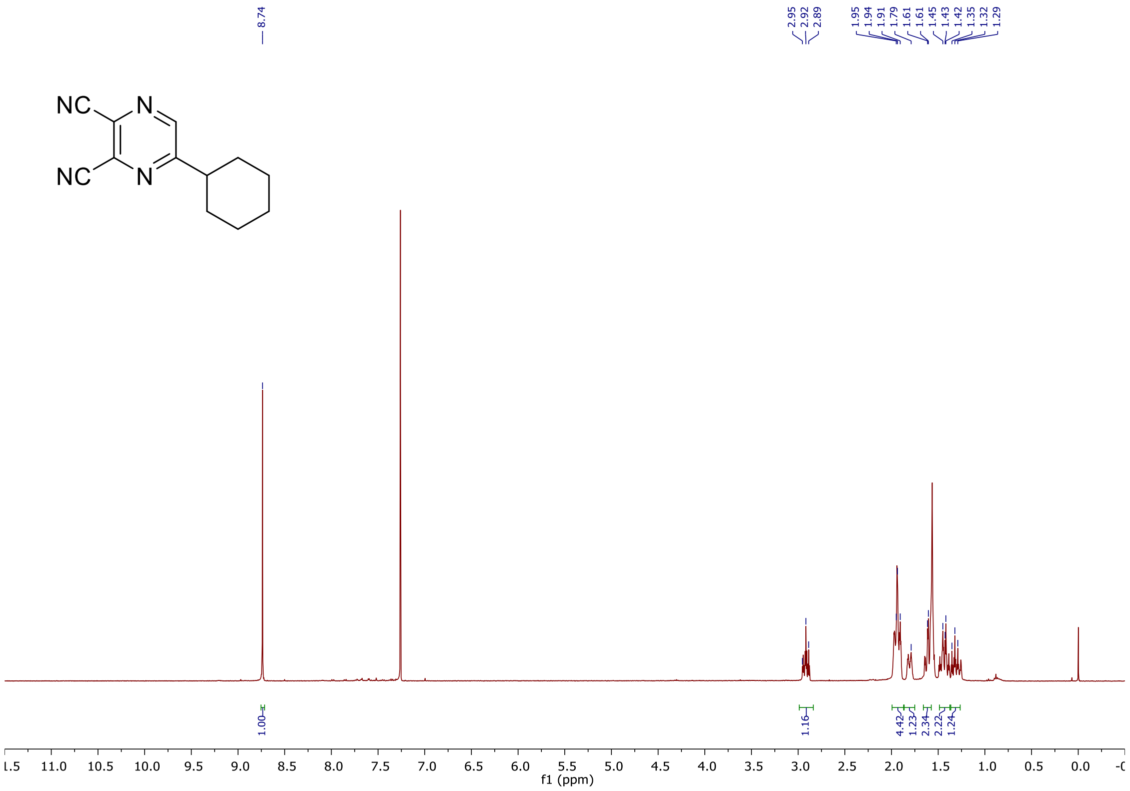

^{13}C NMR (101 MHz, $CDCl_3$) **18**

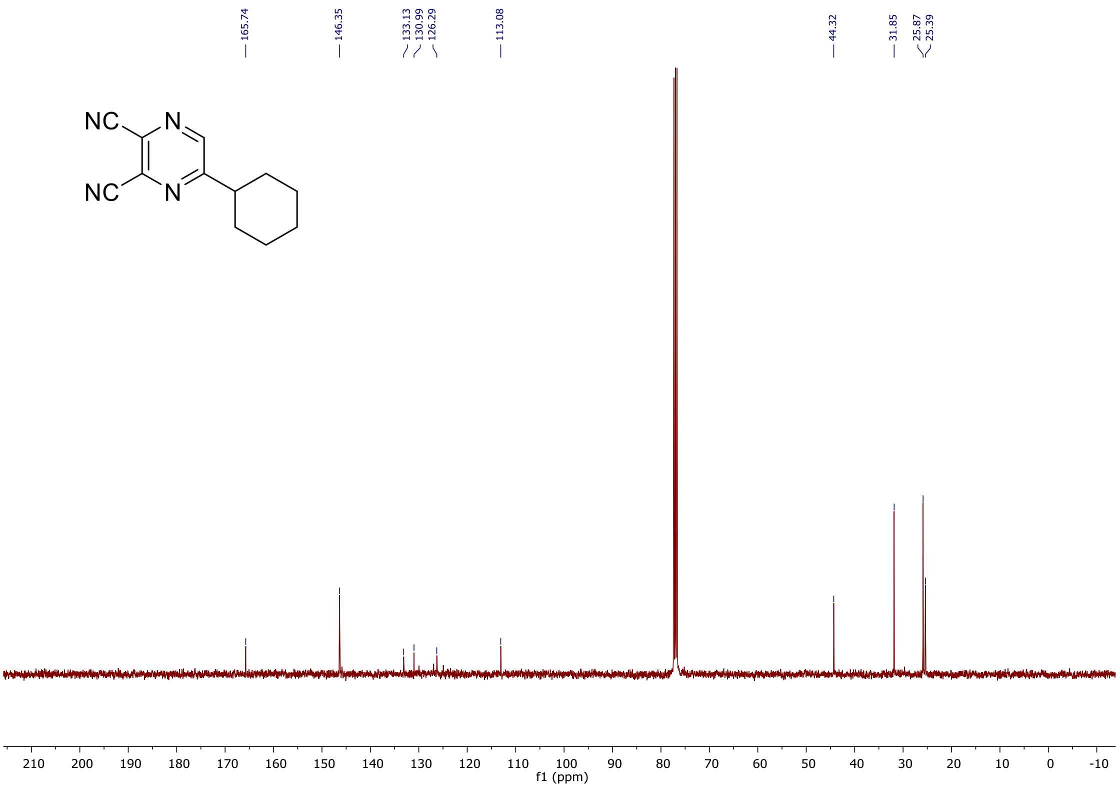

^{1}H NMR (400 MHz, $CDCl_3$) **19**

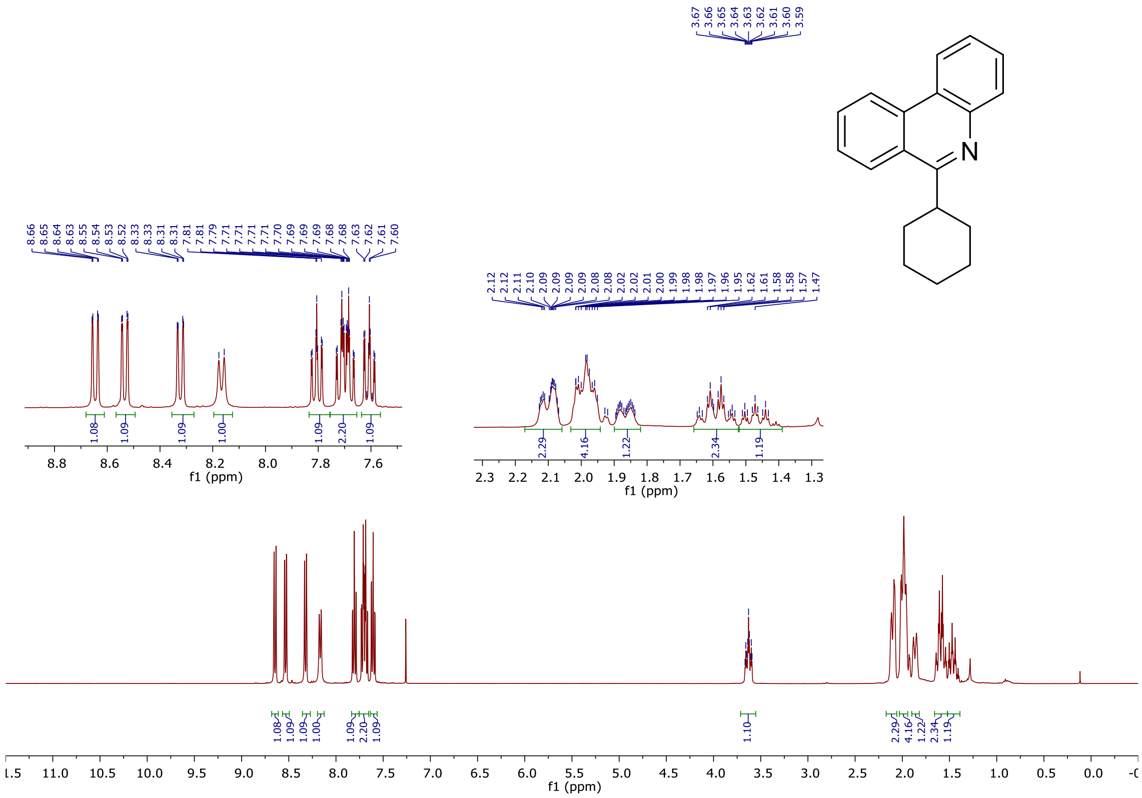

^{13}C NMR (101 MHz, $CDCl_3$) **19**

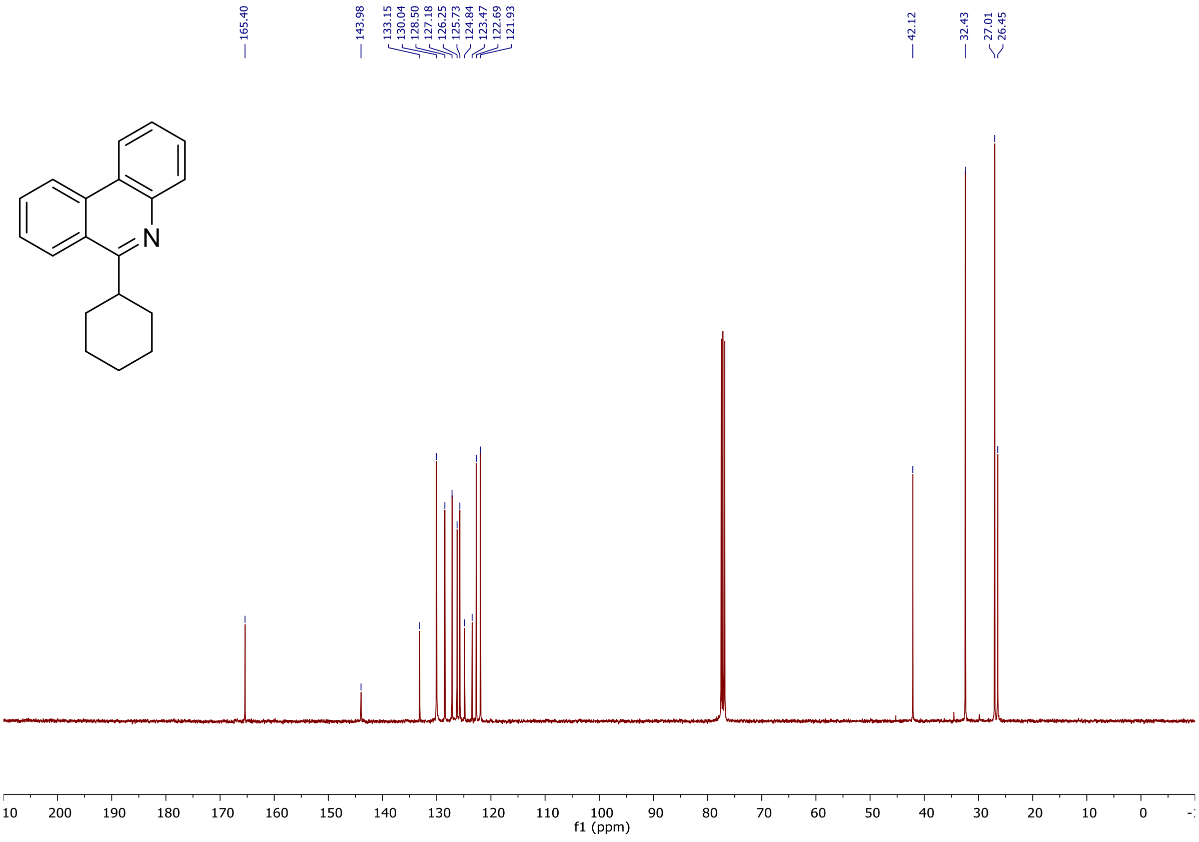

^{1}H NMR (300 MHz, $CDCl_3$) **20**

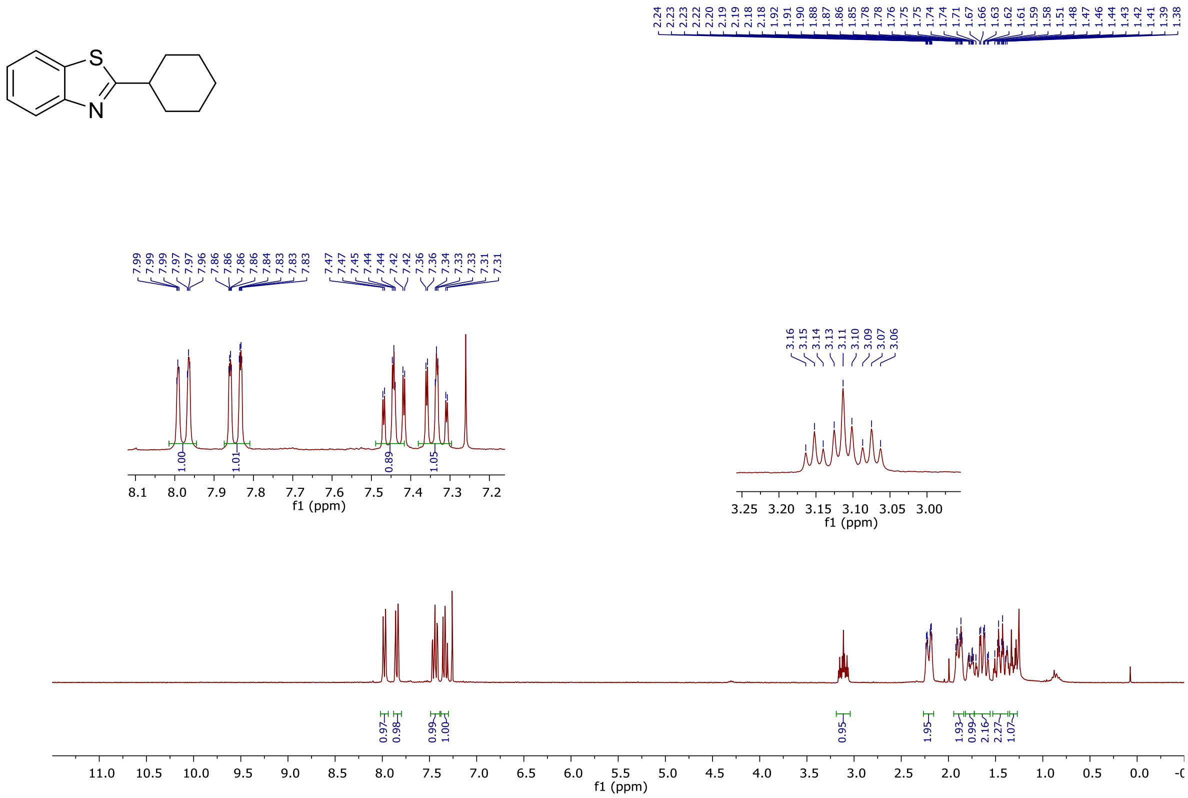

^{13}C NMR (101 MHz, $CDCl_3$) **20**

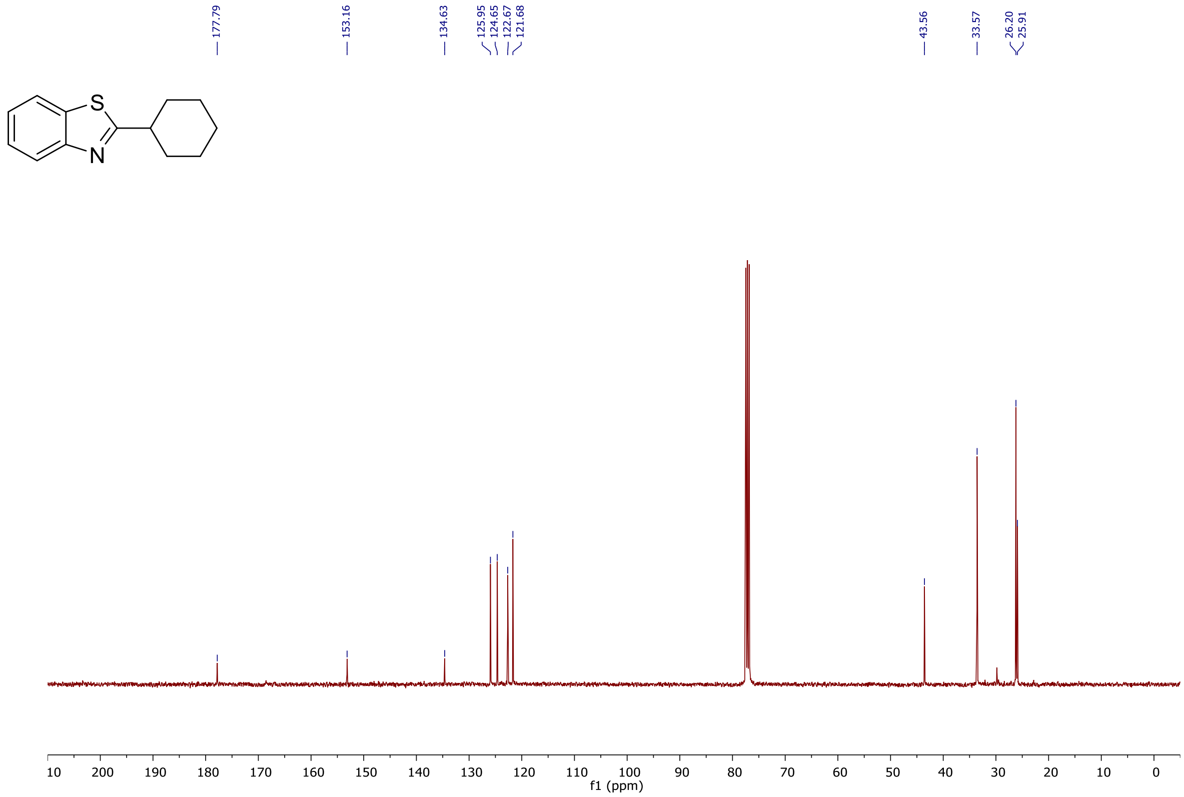

^{1}H NMR (400 MHz, $CDCl_3$) **21**

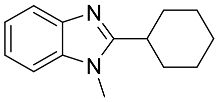

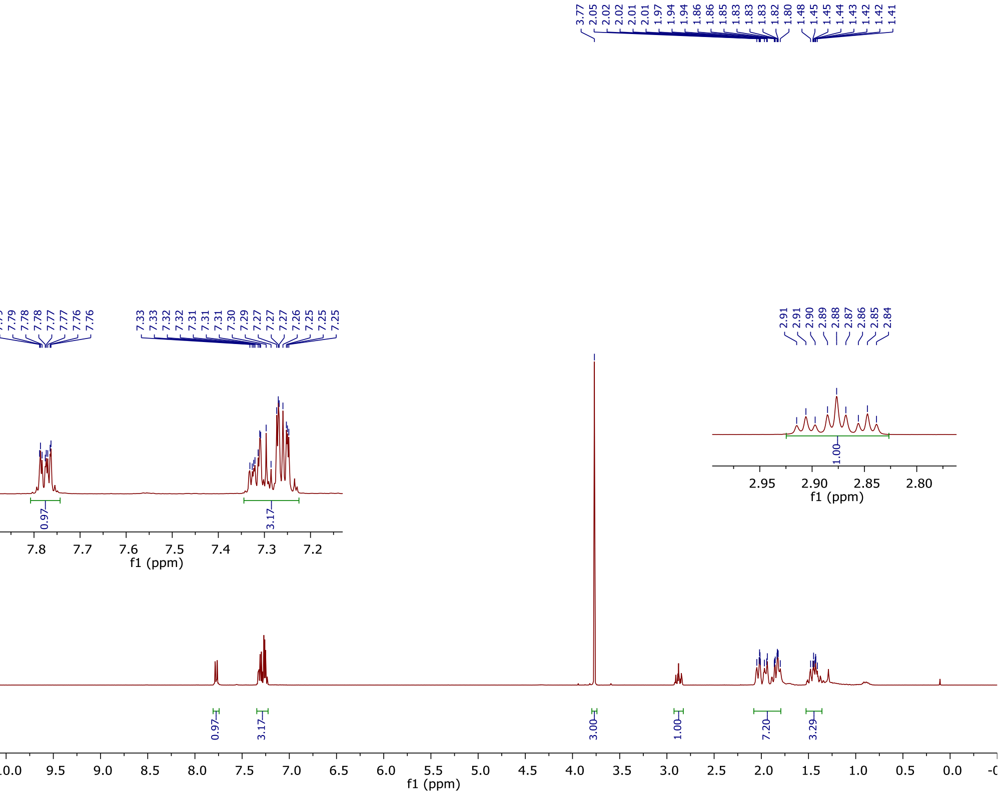

^{13}C NMR (101 MHz, $CDCl_3$) **21**

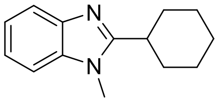

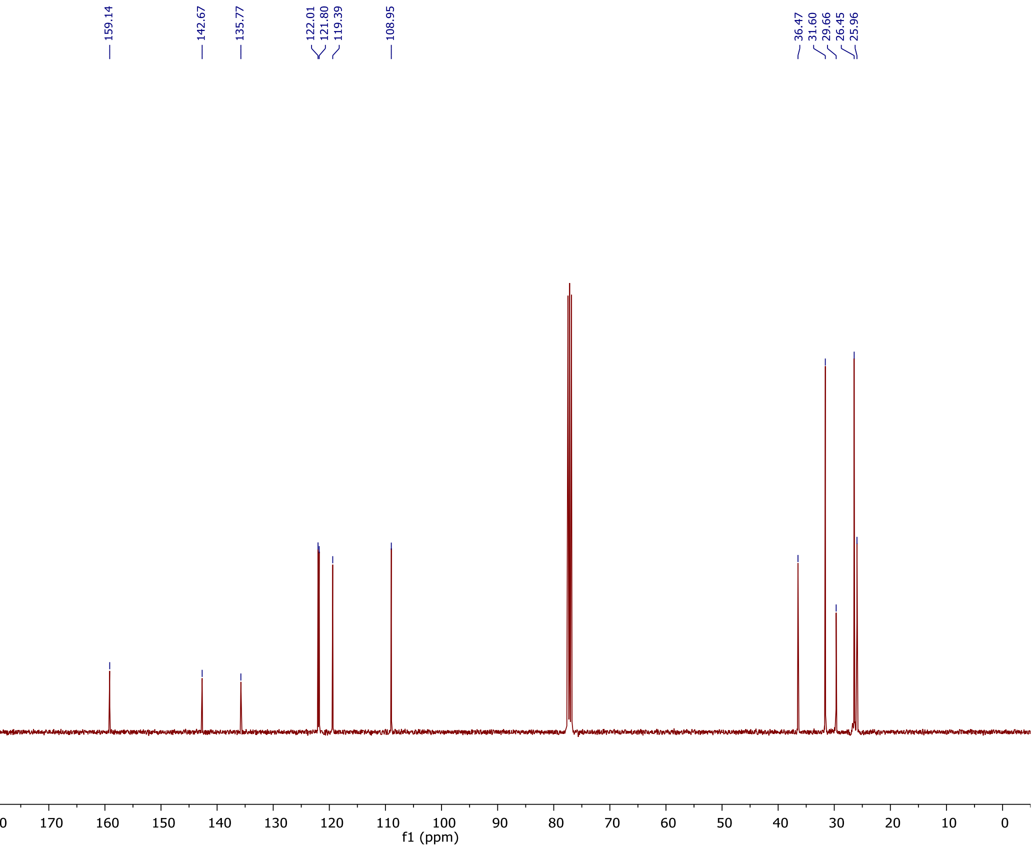

^{1}H NMR (400 MHz, $CDCl_3$) **22**

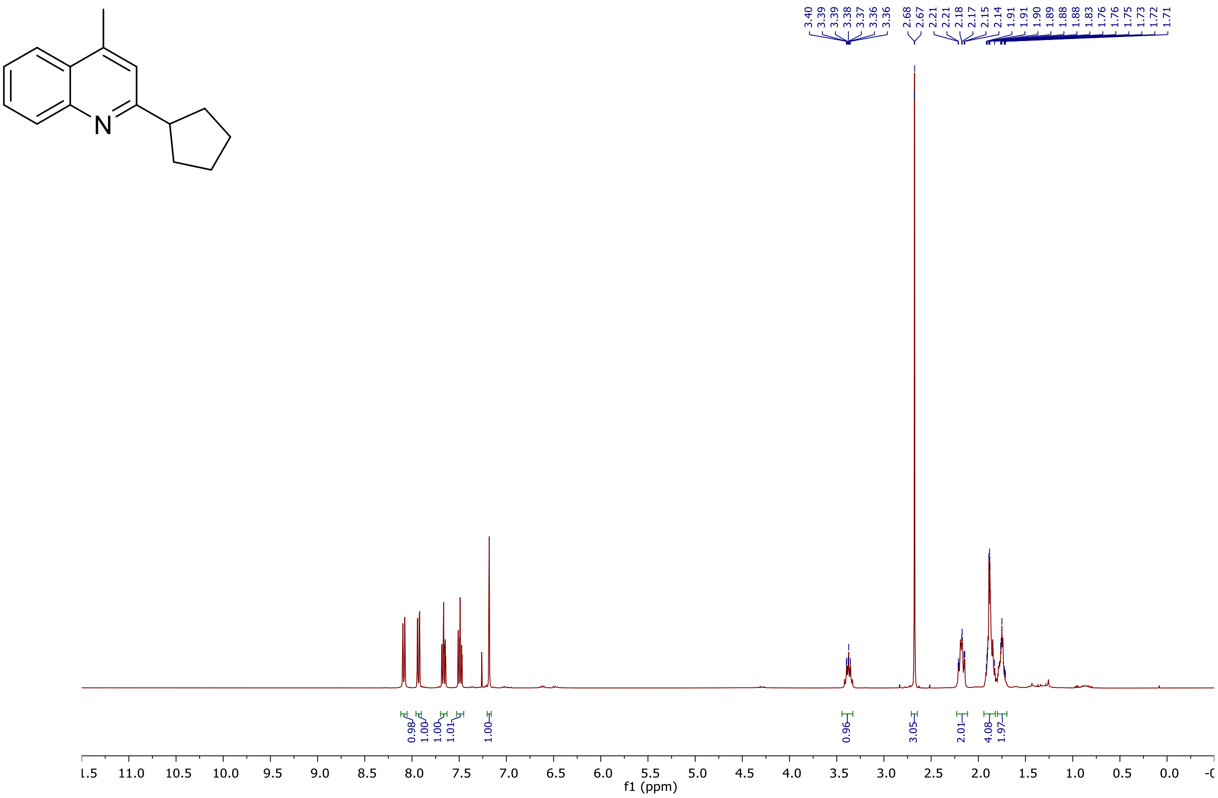

^{13}C NMR (101 MHz, $CDCl_3$) **22**

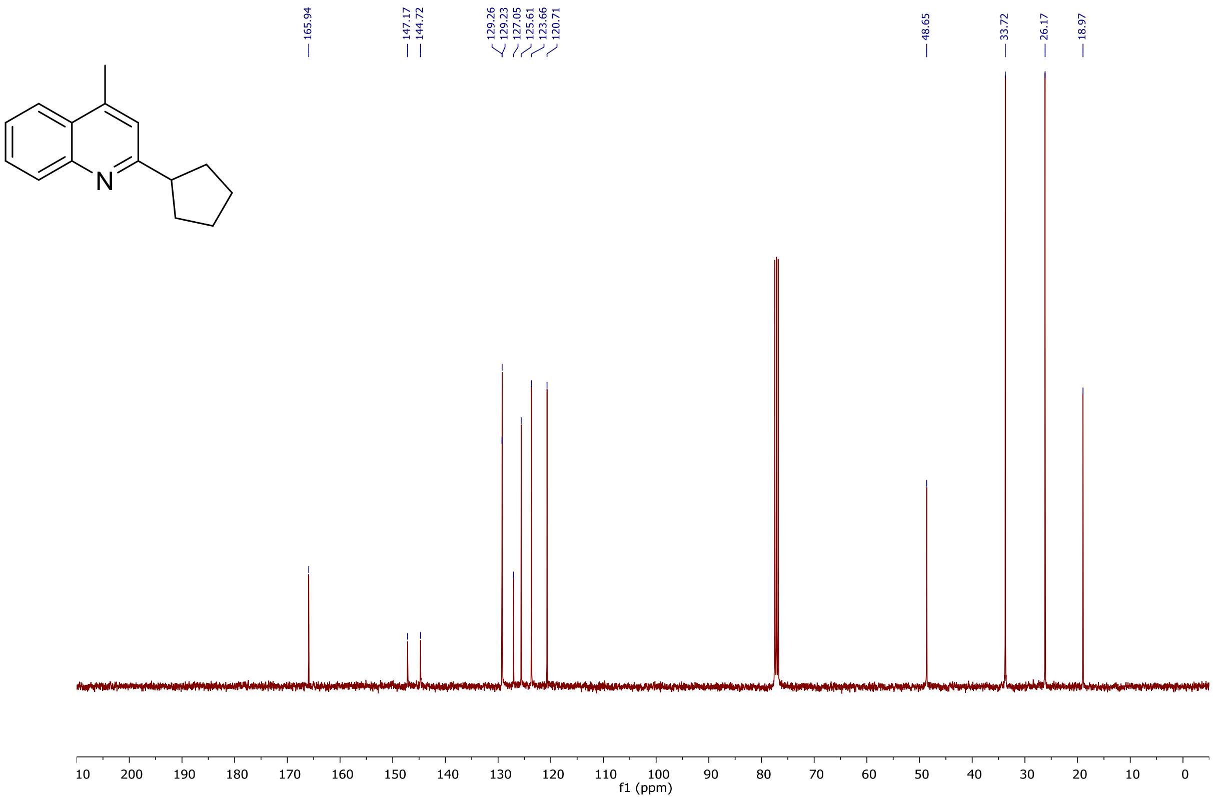

^{1}H NMR (400 MHz, $CDCl_3$) **23**

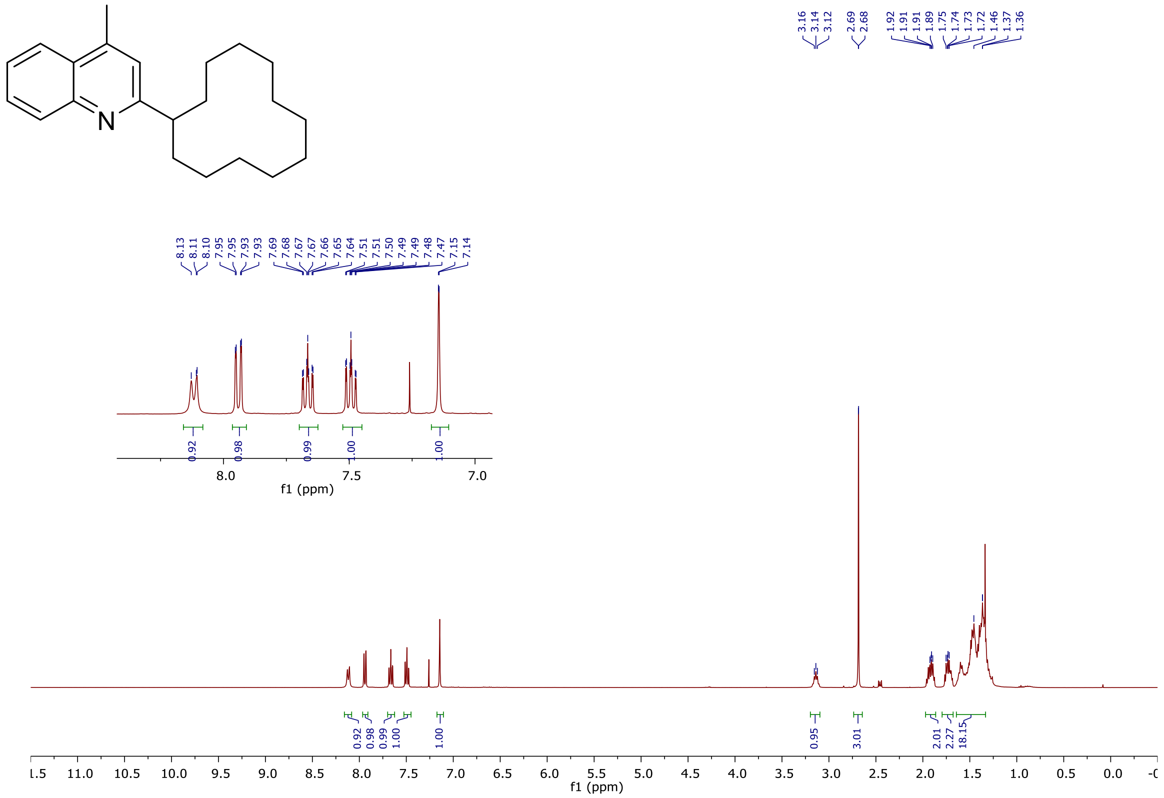

^{13}C NMR (101 MHz, $CDCl_3$) **23**

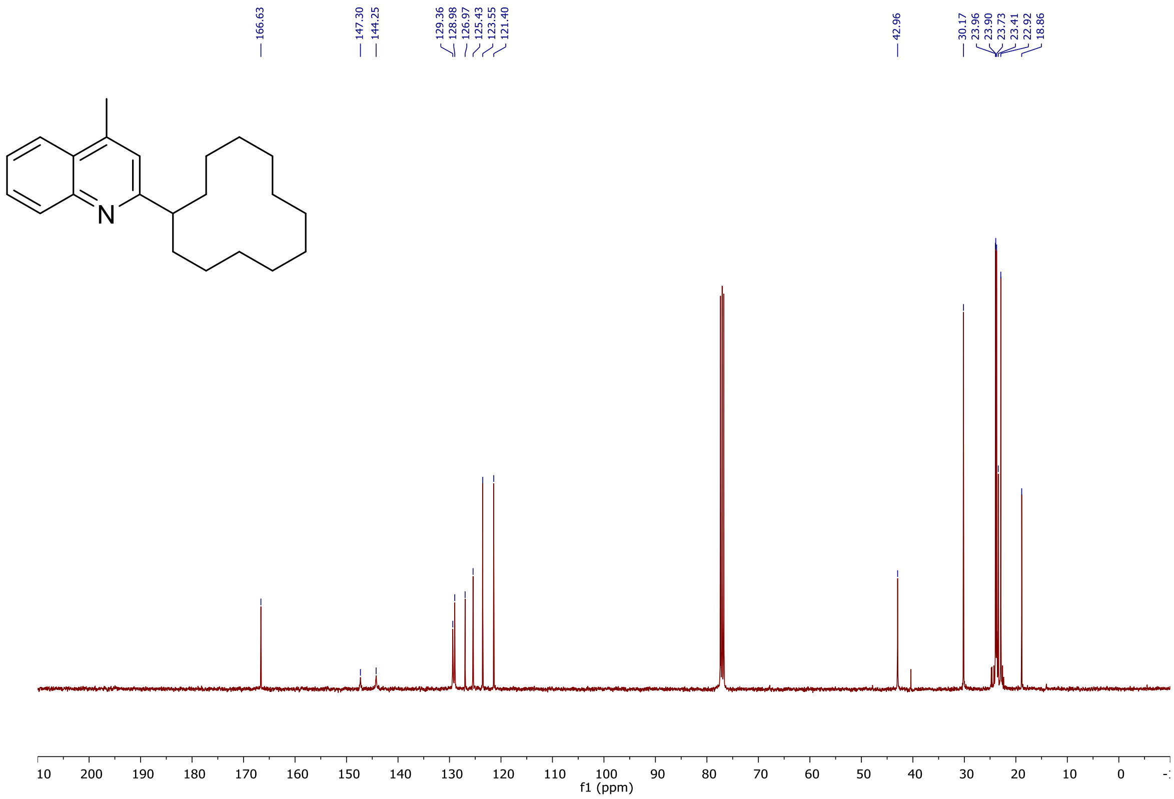

^{1}H NMR (400 MHz, $CDCl_3$) **LO24a**

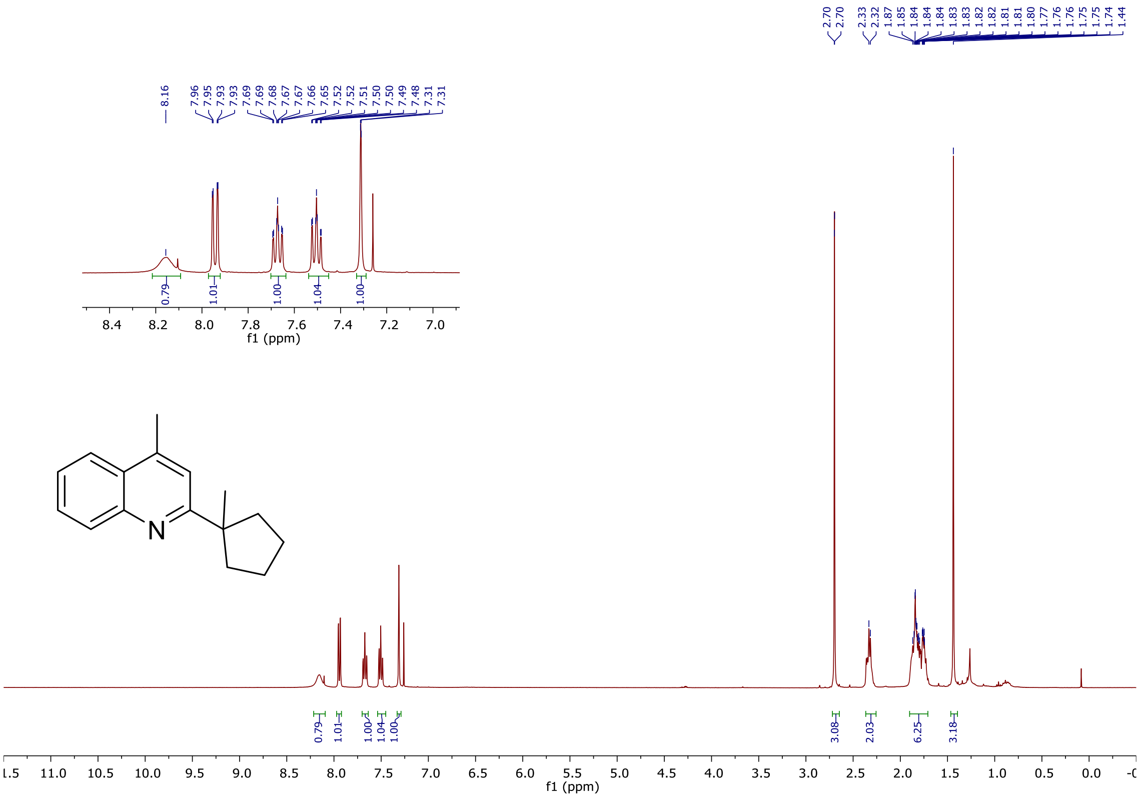

^{13}C NMR (101 MHz, $CDCl_3$) **LO24a**

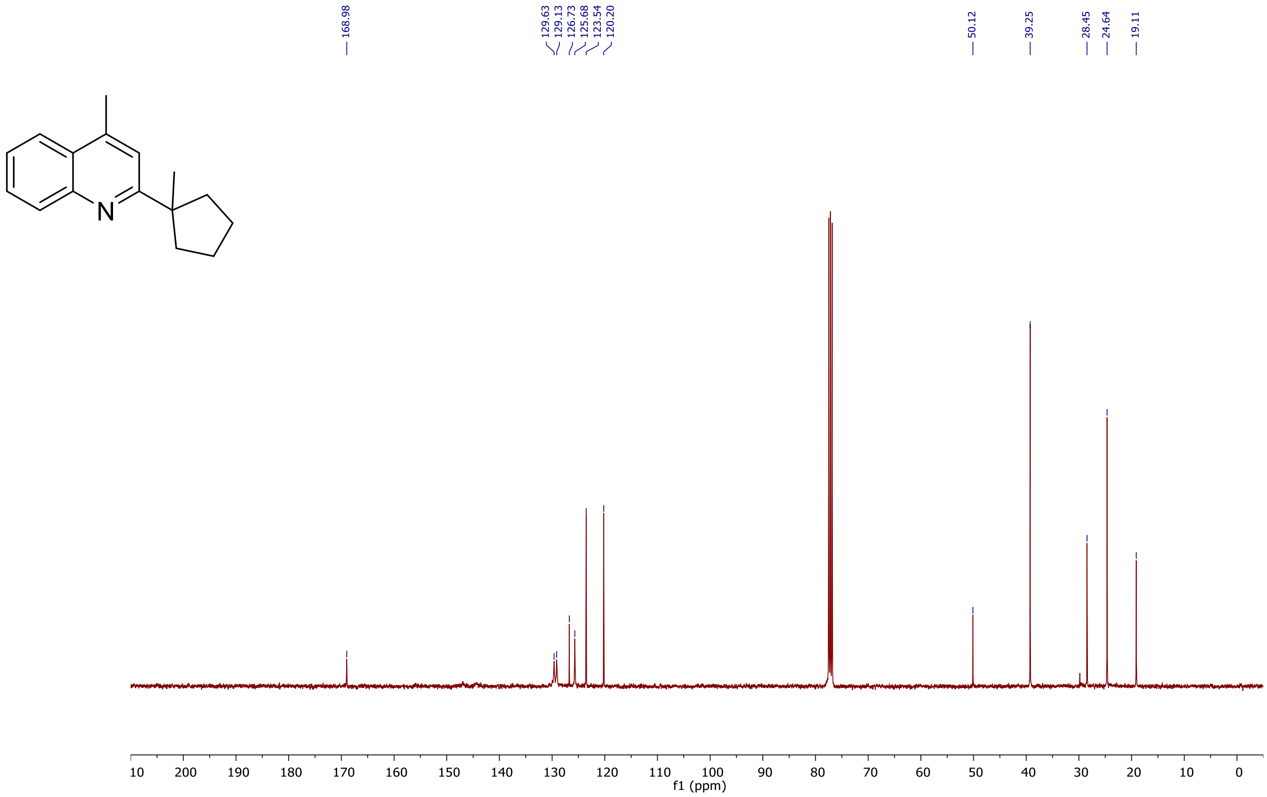

^{1}H NMR (400 MHz, $CDCl_3$) **24b**+**24c**

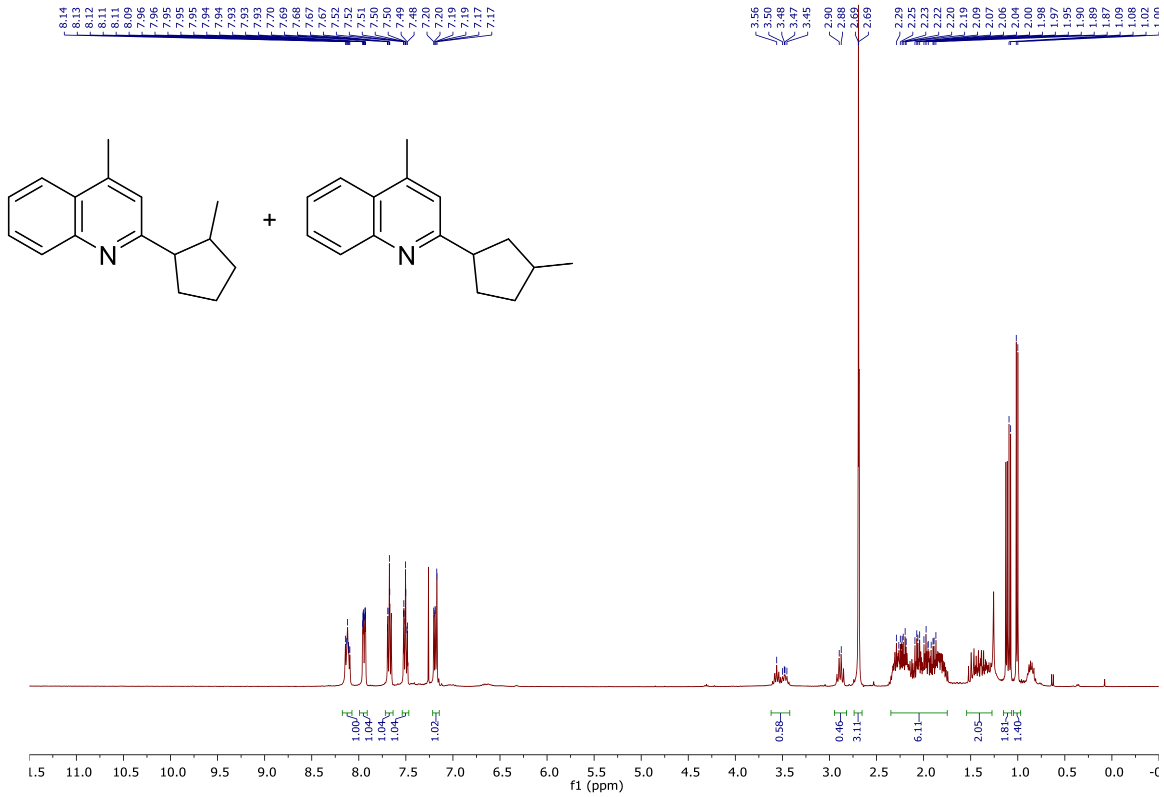

^{13}C NMR (101 MHz, $CDCl_3$) **24b**+**24c**

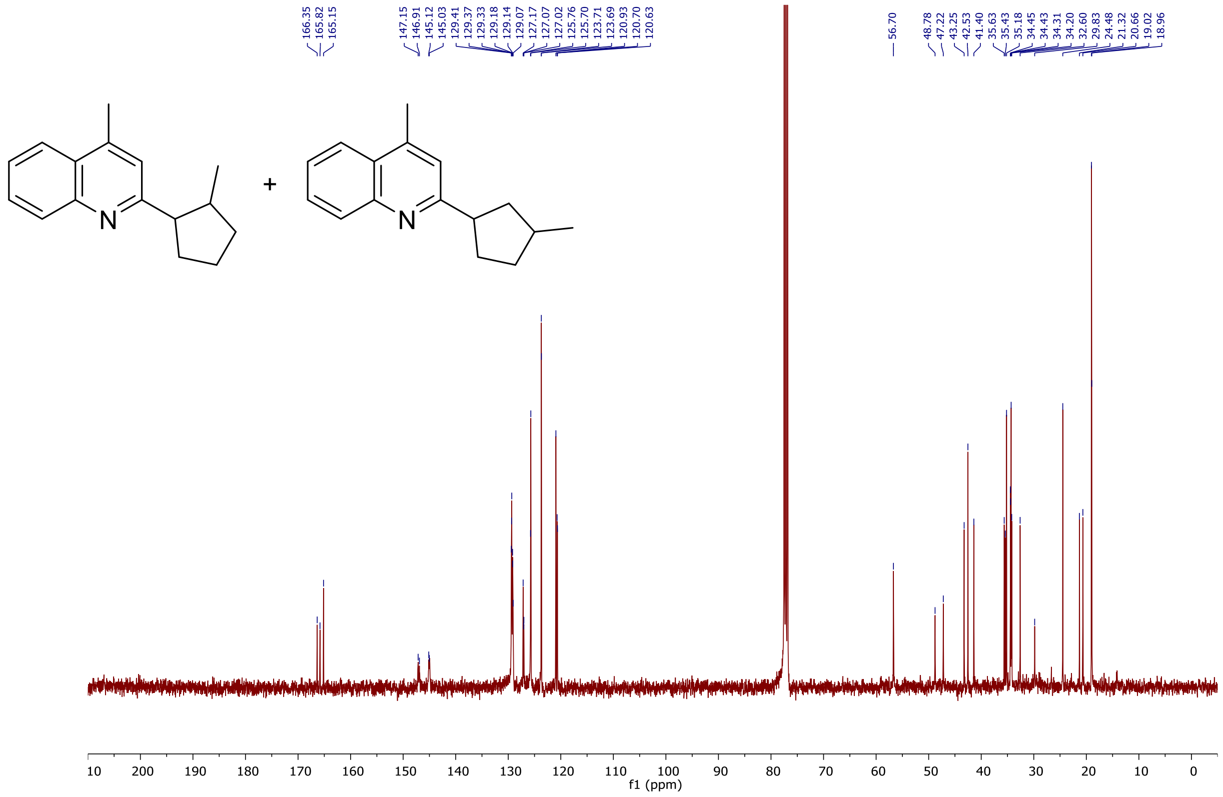

^{1}H NMR (400 MHz, $CDCl_3$) **25**

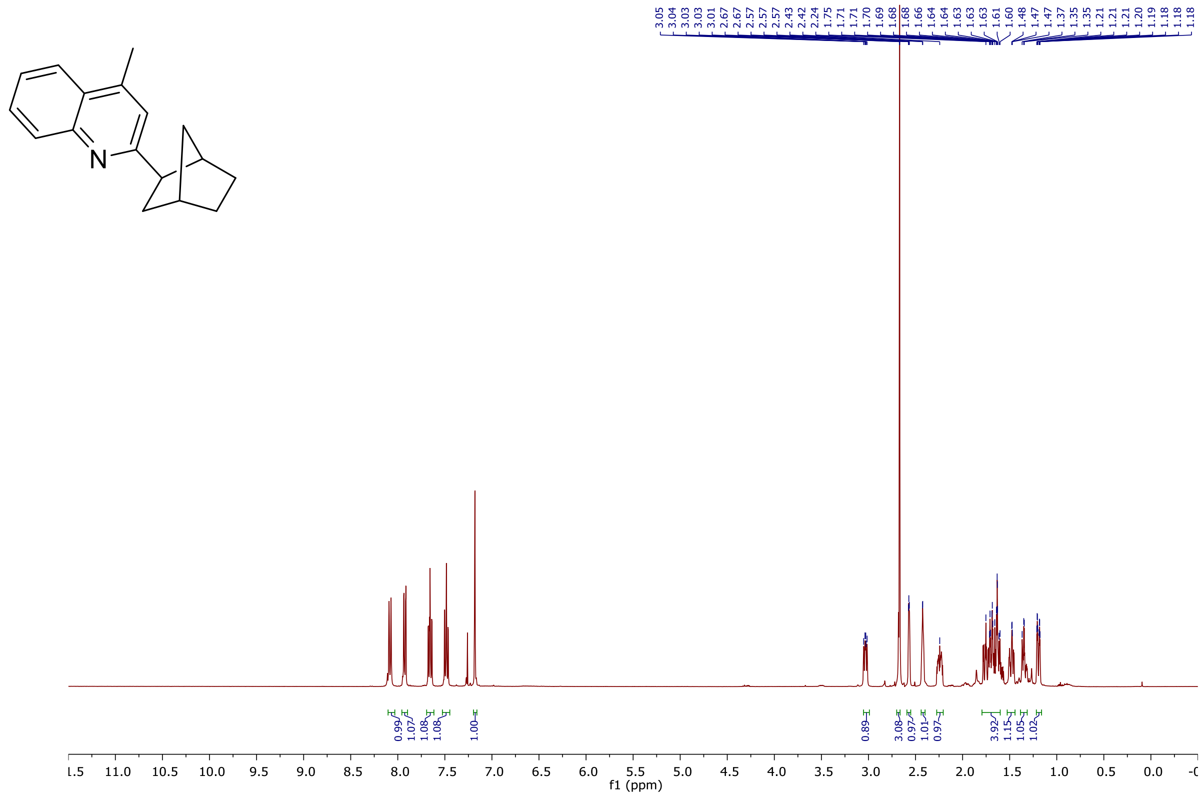

^{13}C NMR (101 MHz, $CDCl_3$) **25**

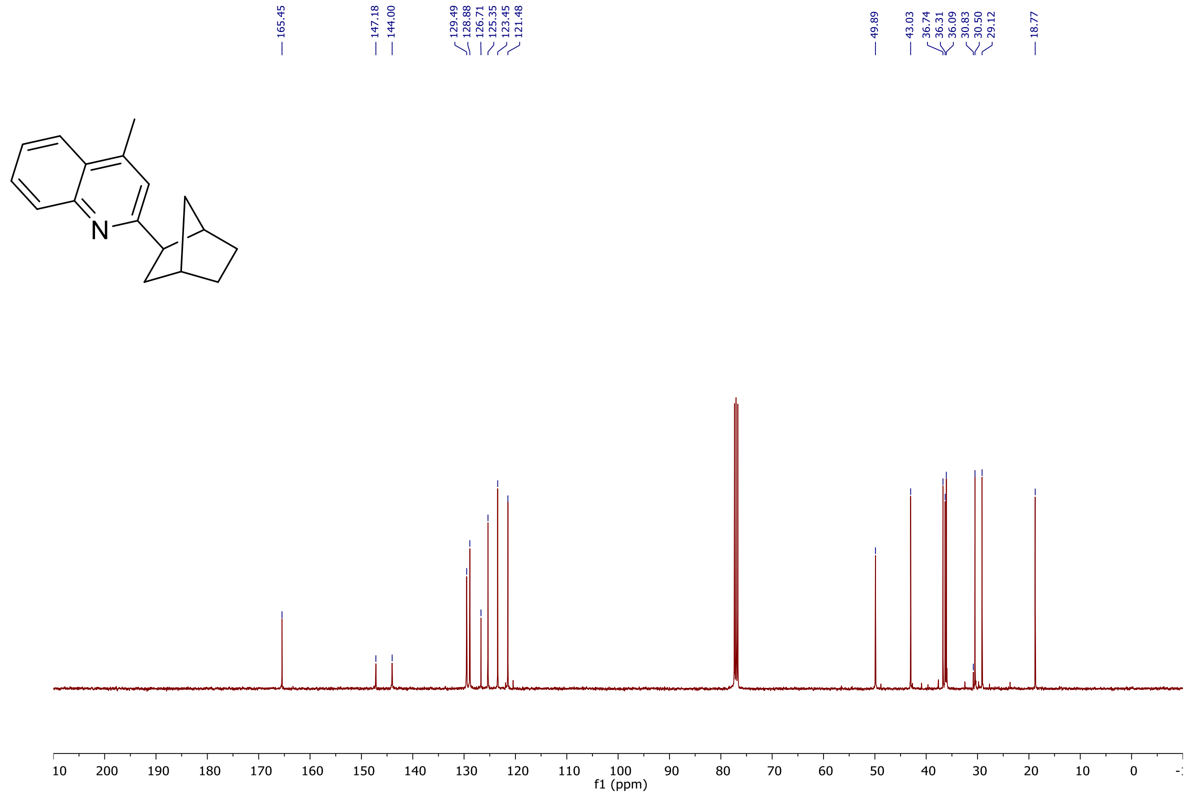

^{1}H NMR (500 MHz, $CDCl_3$) **26**

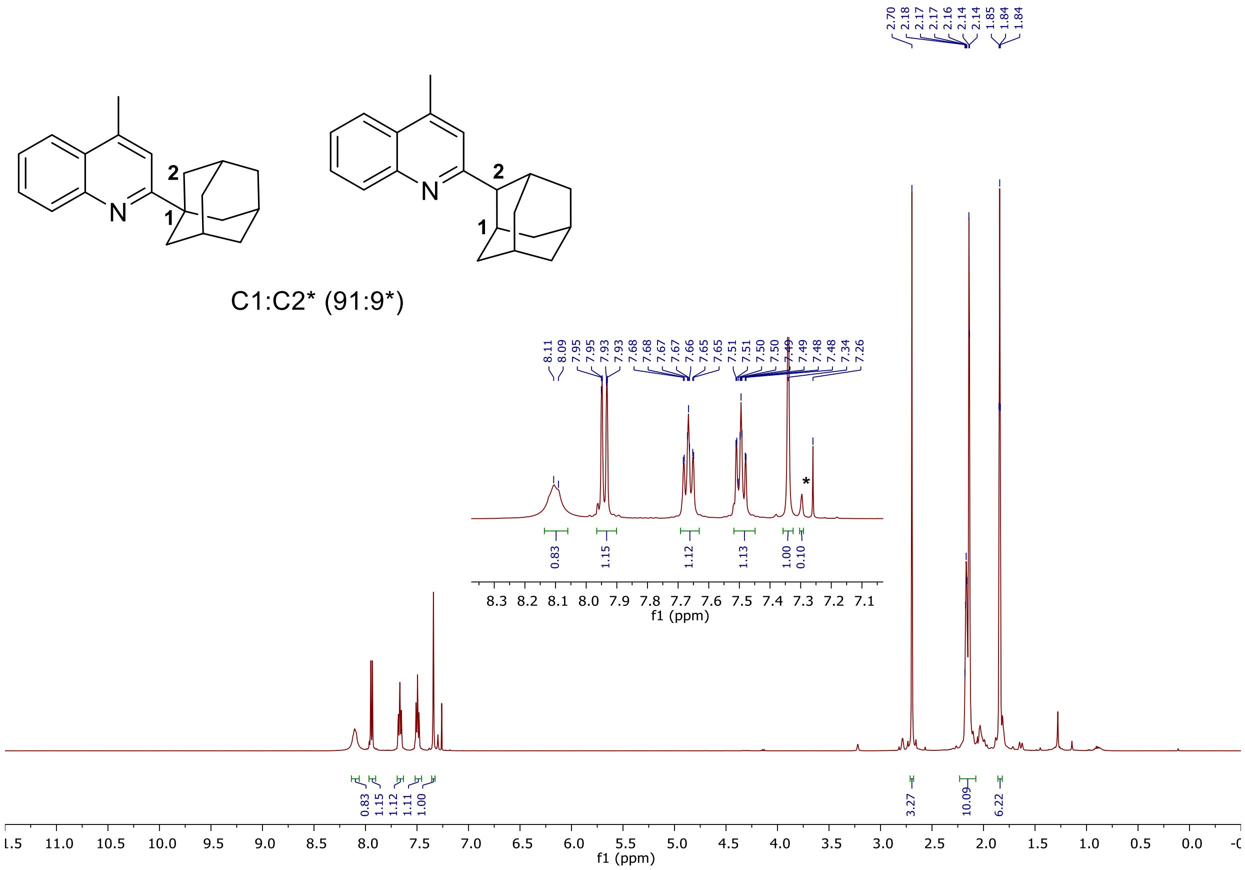

^{13}C NMR (126 MHz, $CDCl_3$) **26**

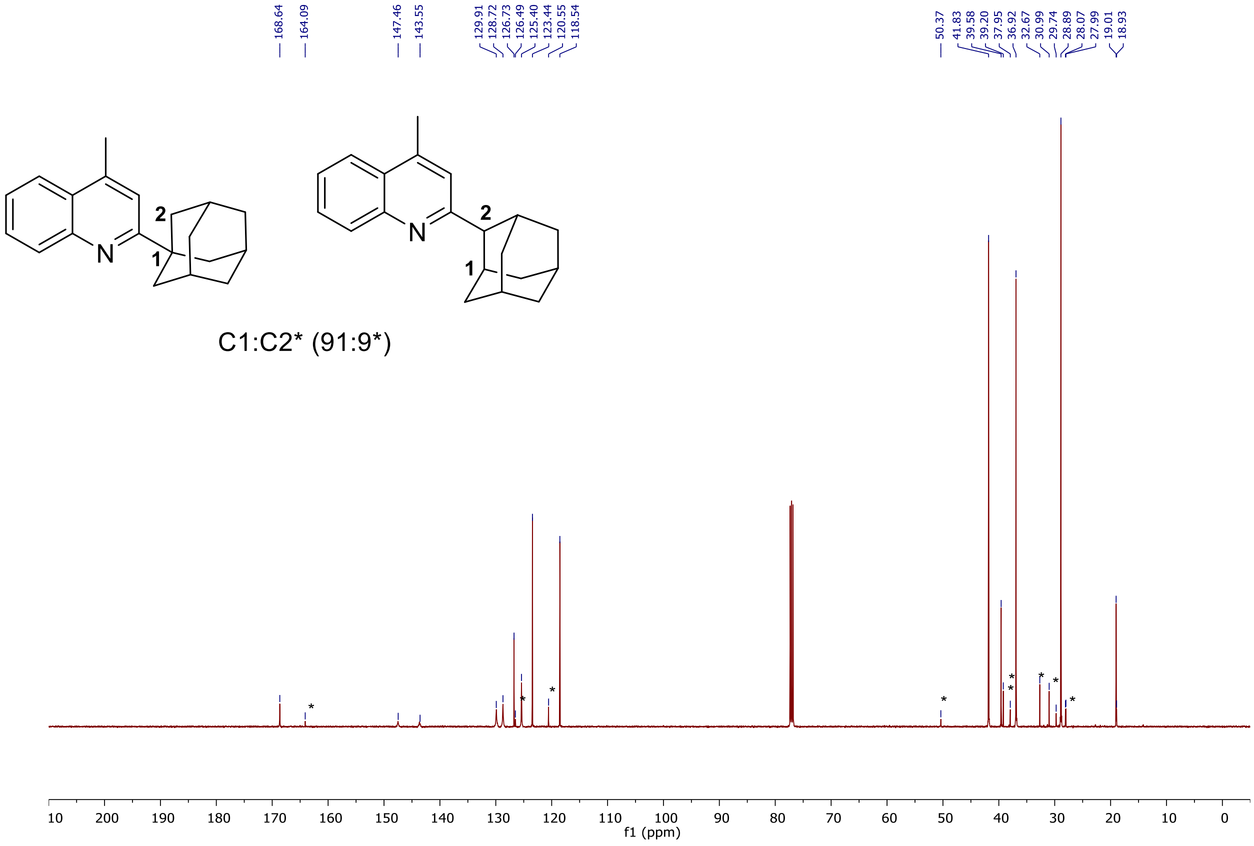

^{1}H NMR (400 MHz, $CDCl_3$) **27**

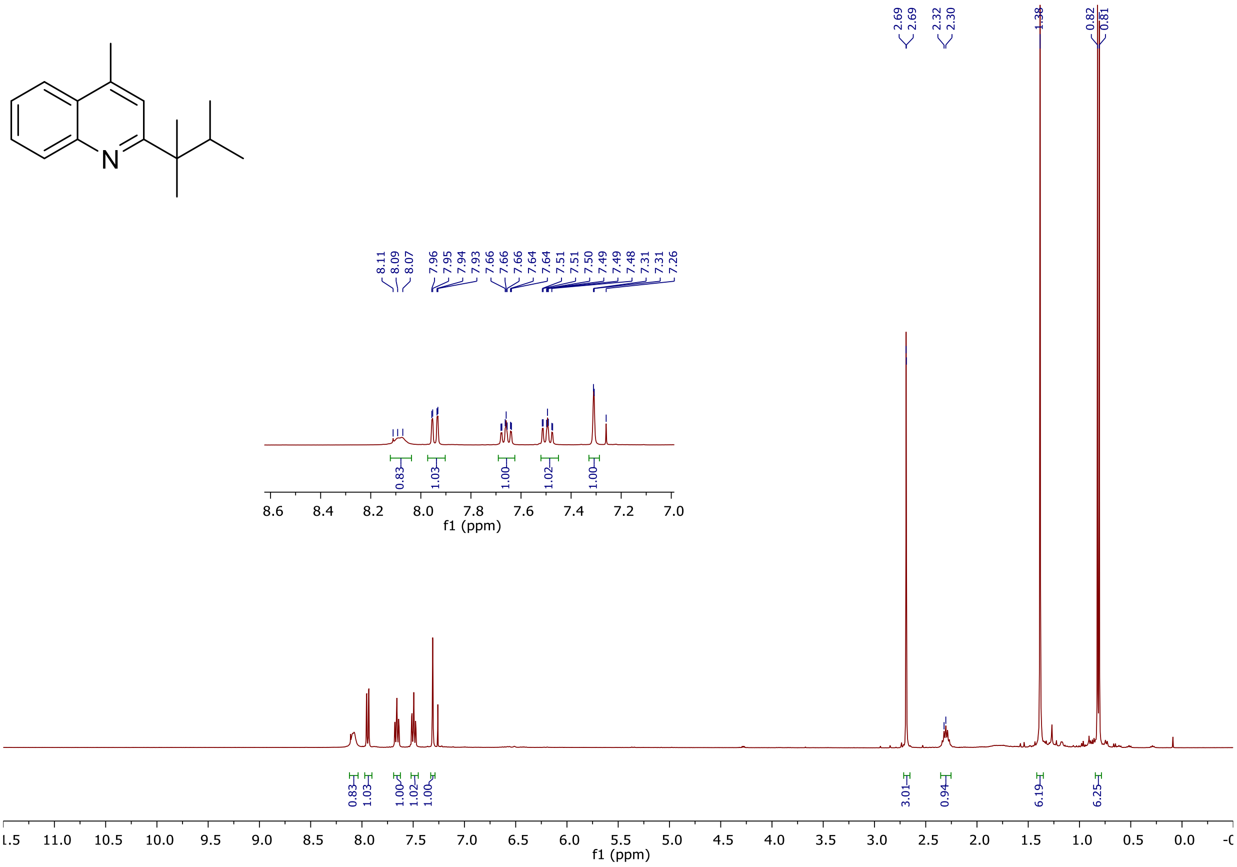

^{13}C NMR (101 MHz, $CDCl_3$) **27**

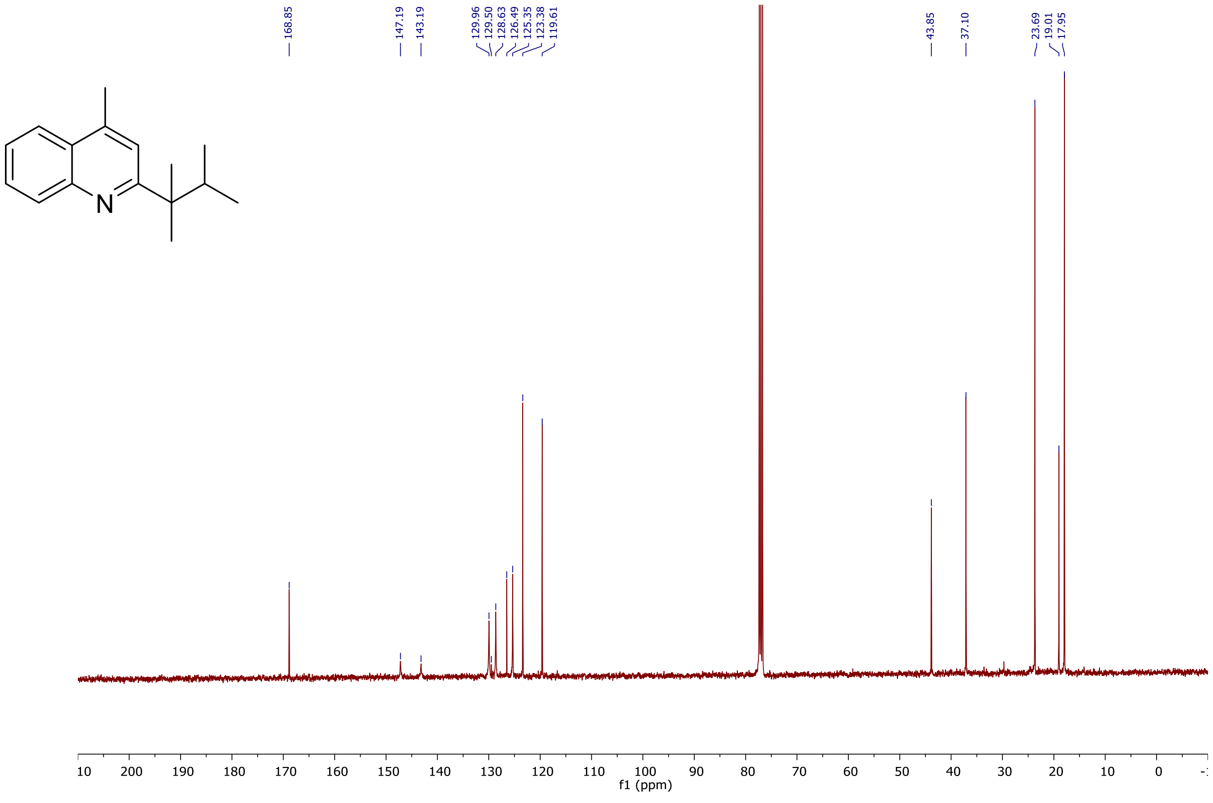

^{1}H NMR (400 MHz, $CDCl_3$) **28**

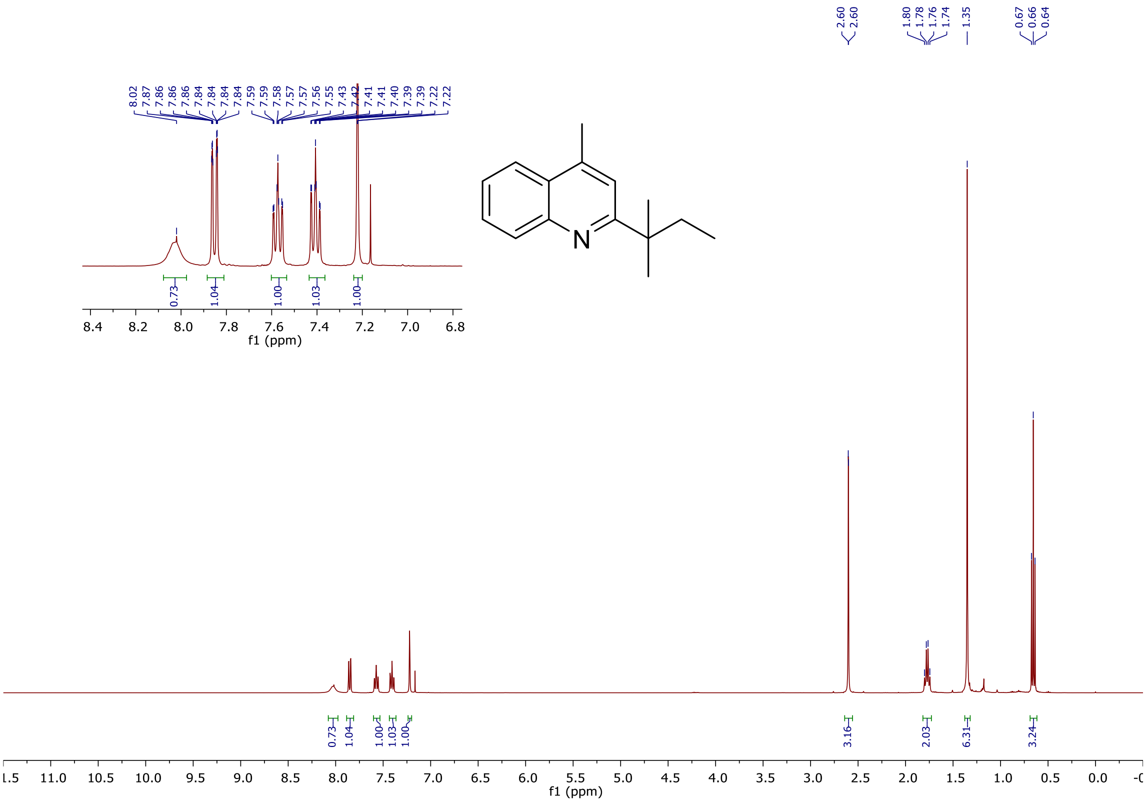

^{13}C NMR (101 MHz, $CDCl_3$) **28**

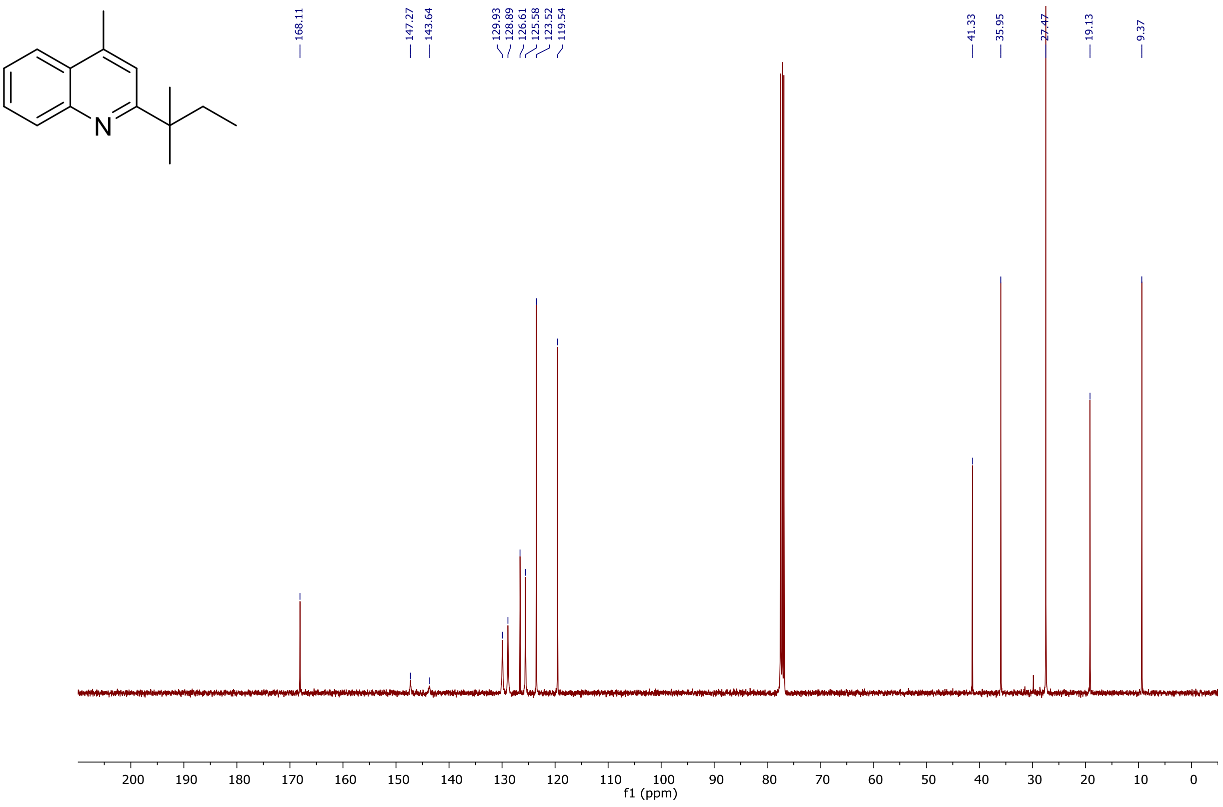

^{1}H NMR (400 MHz, $CDCl_3$) **29**

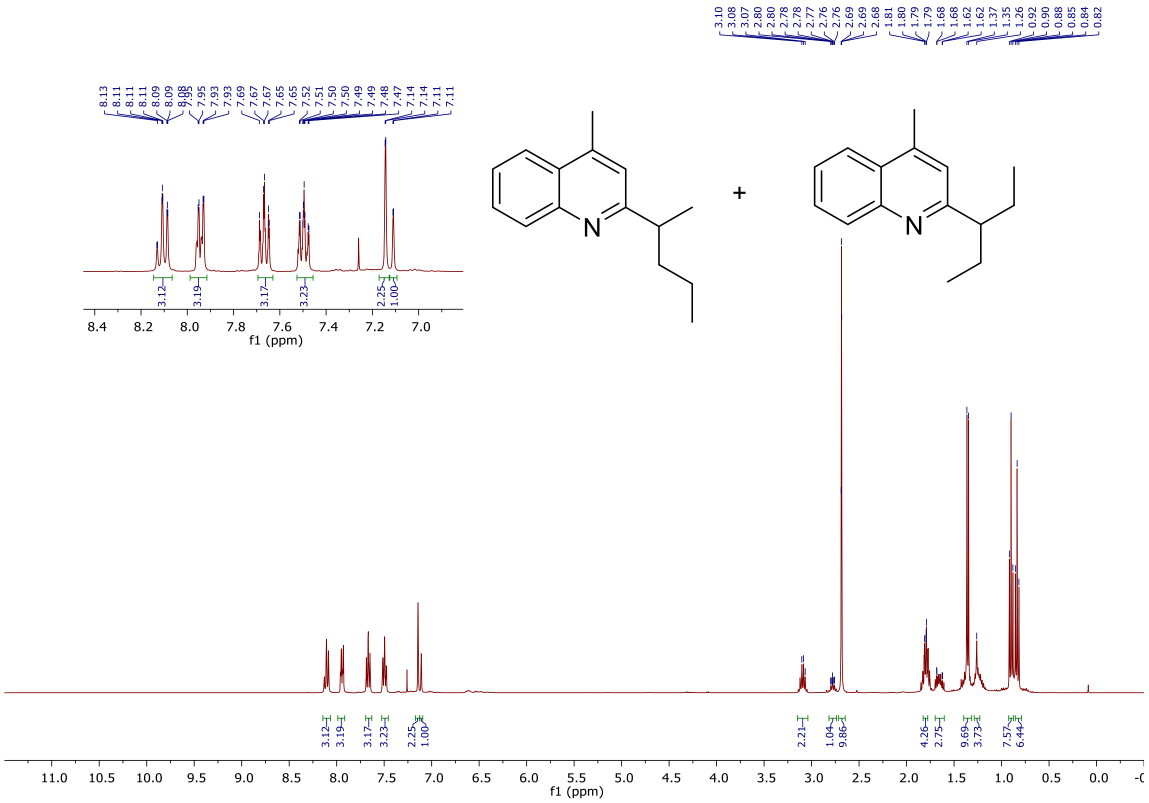

^{13}C NMR (101 MHz, $CDCl_3$) **29**

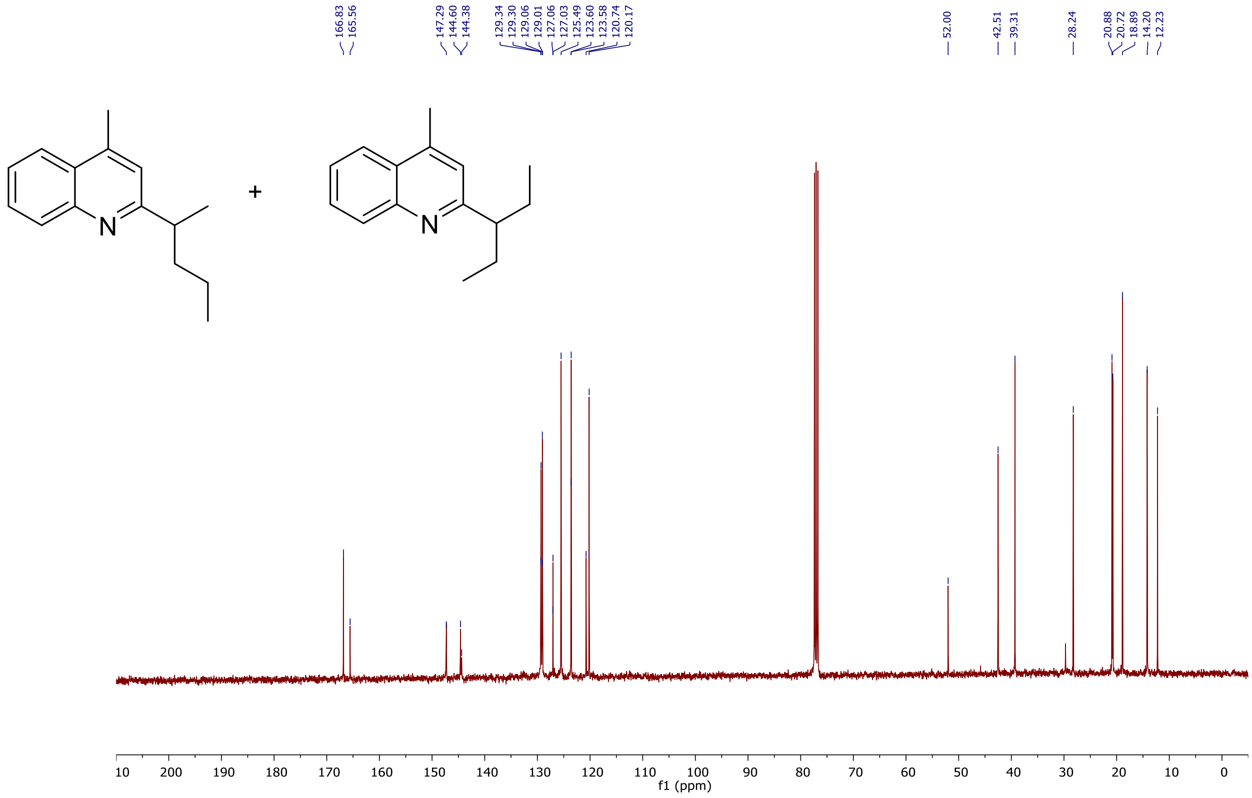

^{1}H NMR (400 MHz, $CDCl_3$) **30**

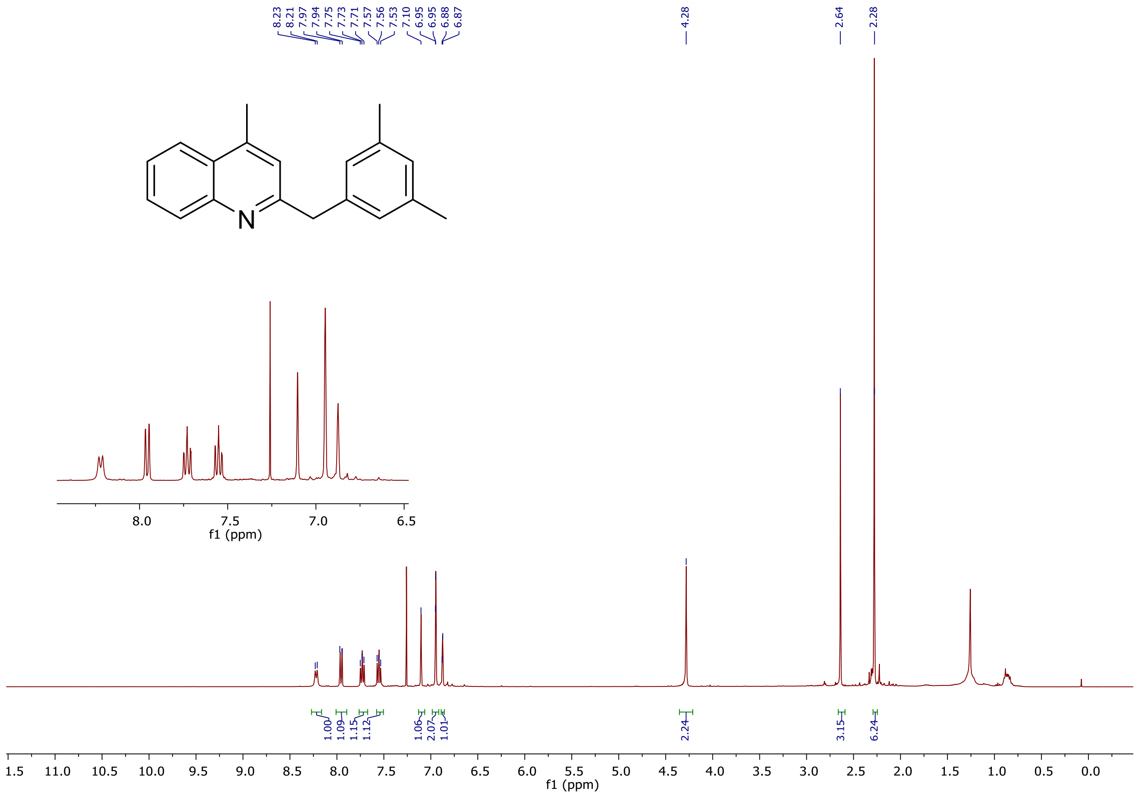

^{13}C NMR (101 MHz, $CDCl_3$) **30**

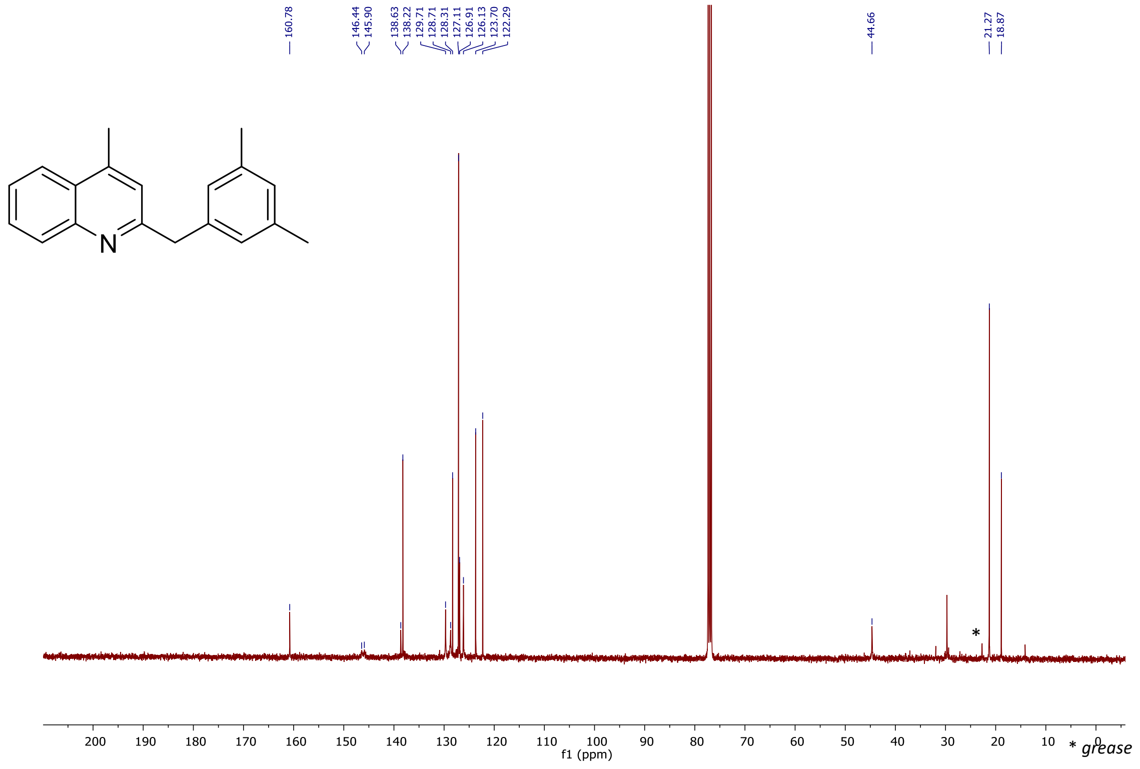

^{1}H NMR (400 MHz, $CDCl_3$) **31**

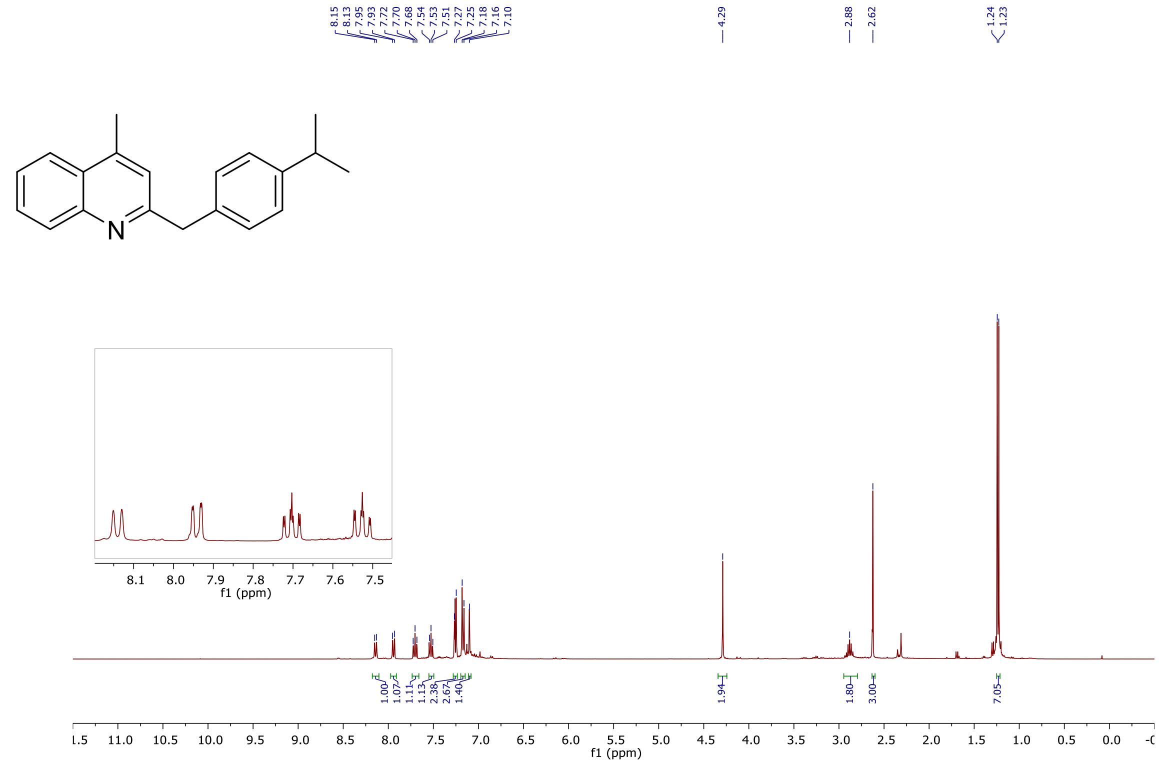

^{13}C NMR (101 MHz, $CDCl_3$) **31**

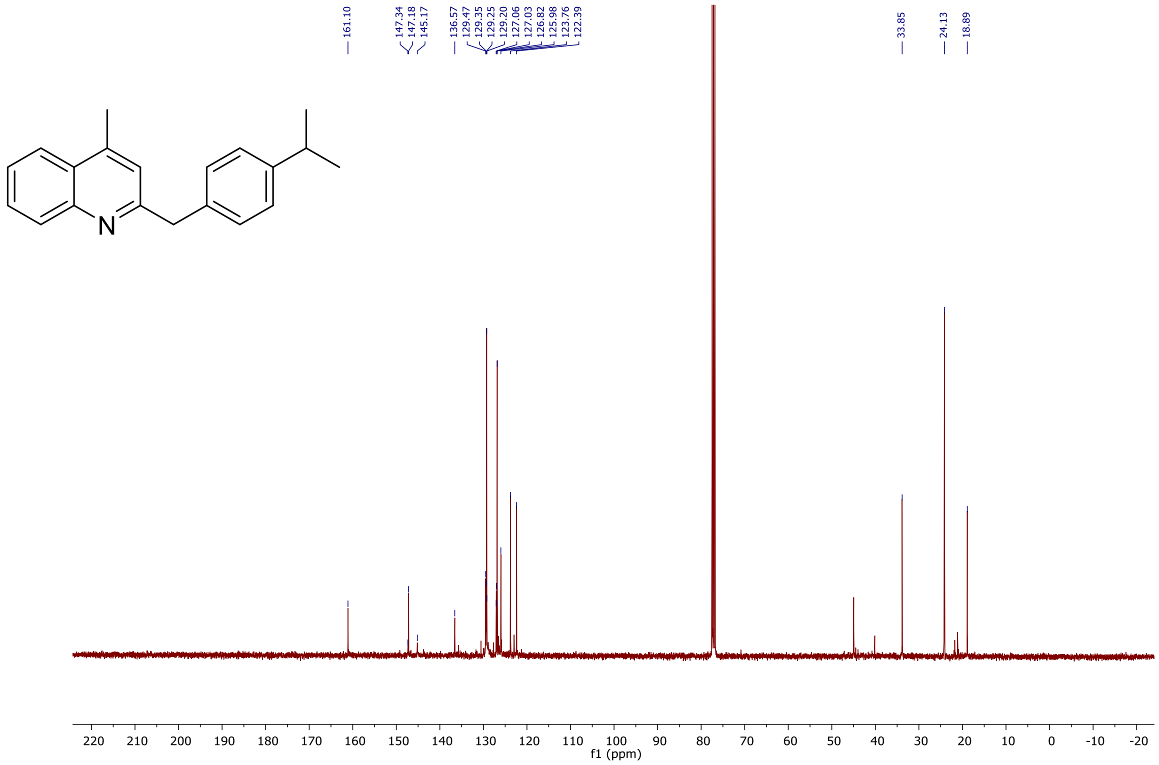

^{1}H NMR (400 MHz, $CDCl_3$) **32**

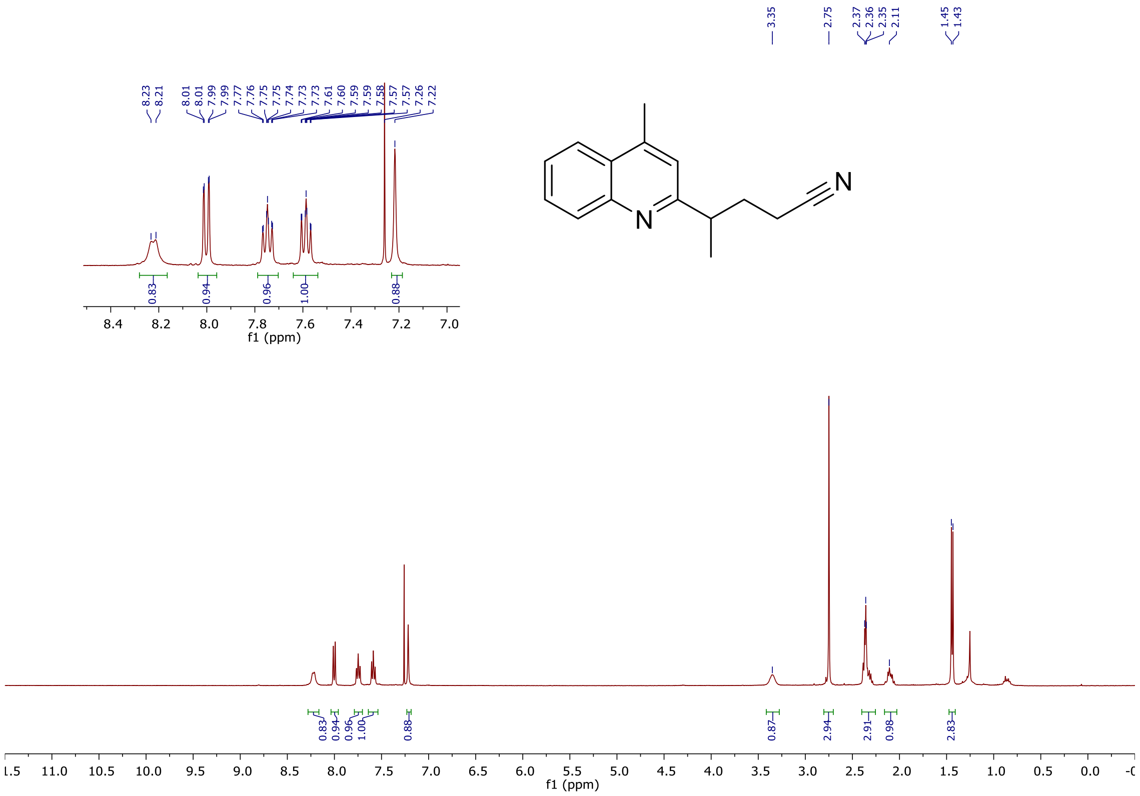

^{13}C NMR (101 MHz, $CDCl_3$) **32**

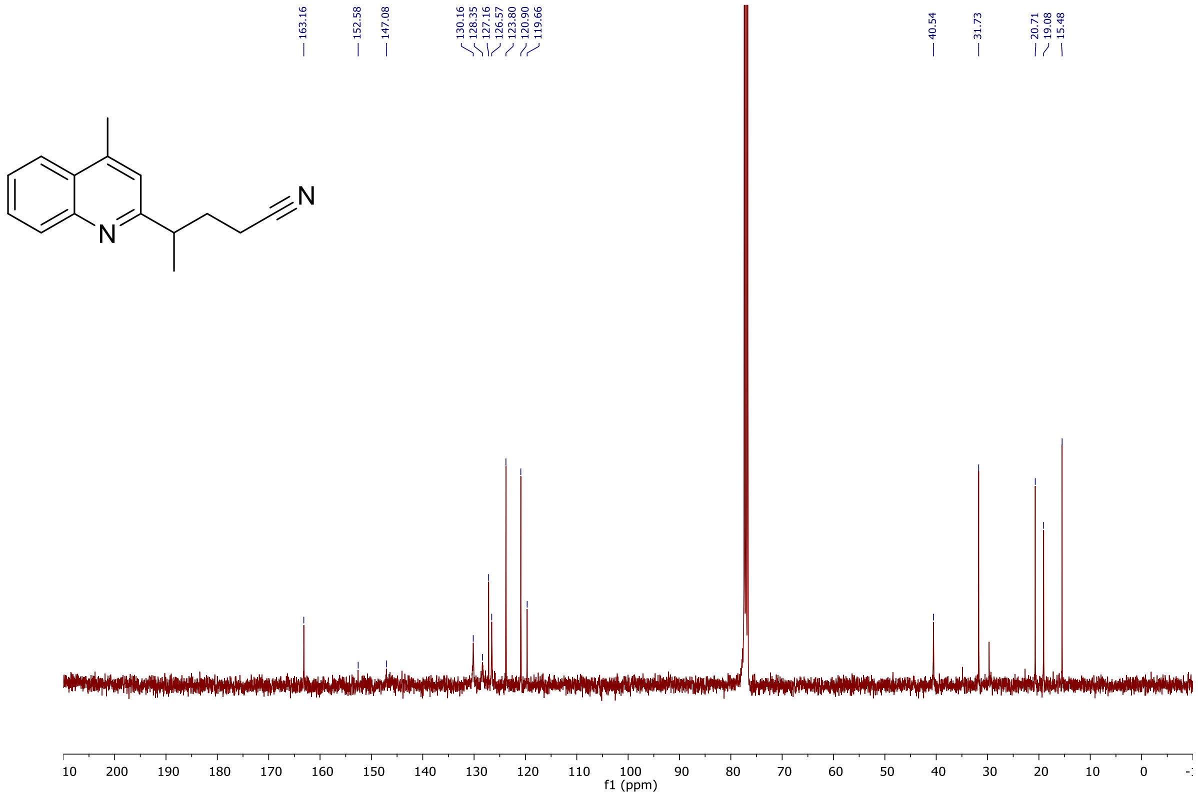

^{1}H NMR (400 MHz, $CDCl_3$) **33**

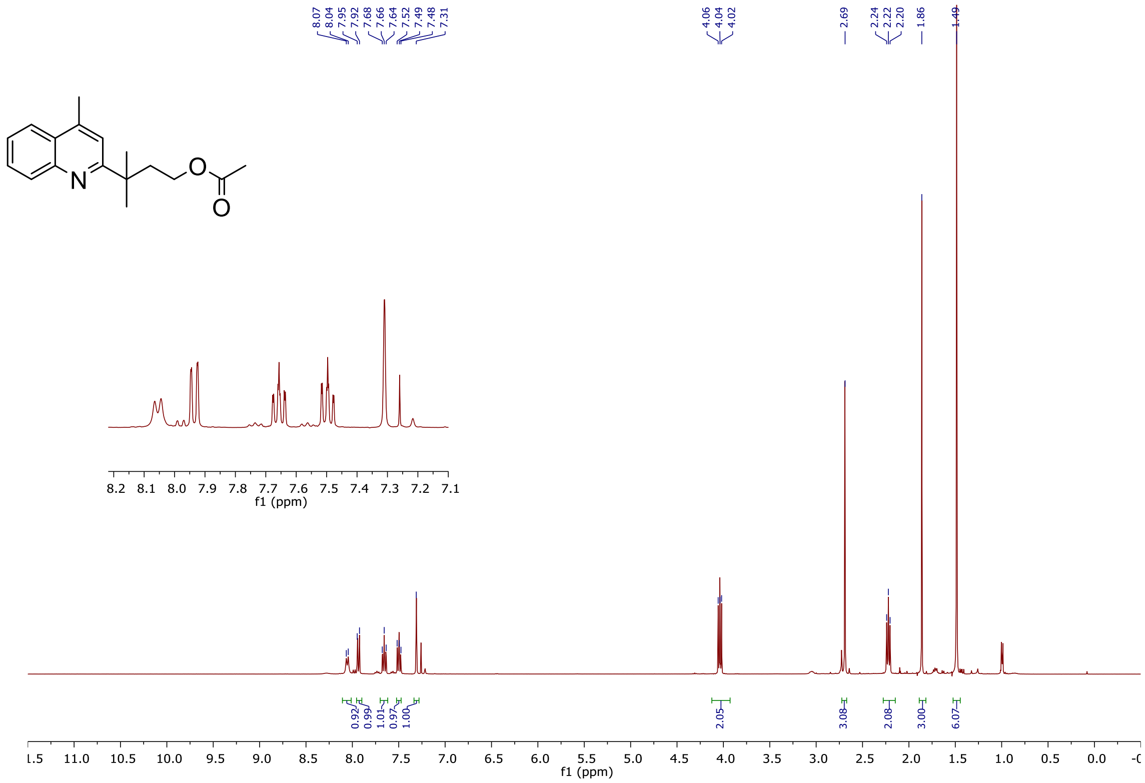

^{13}C NMR (101 MHz, $CDCl_3$) **33**

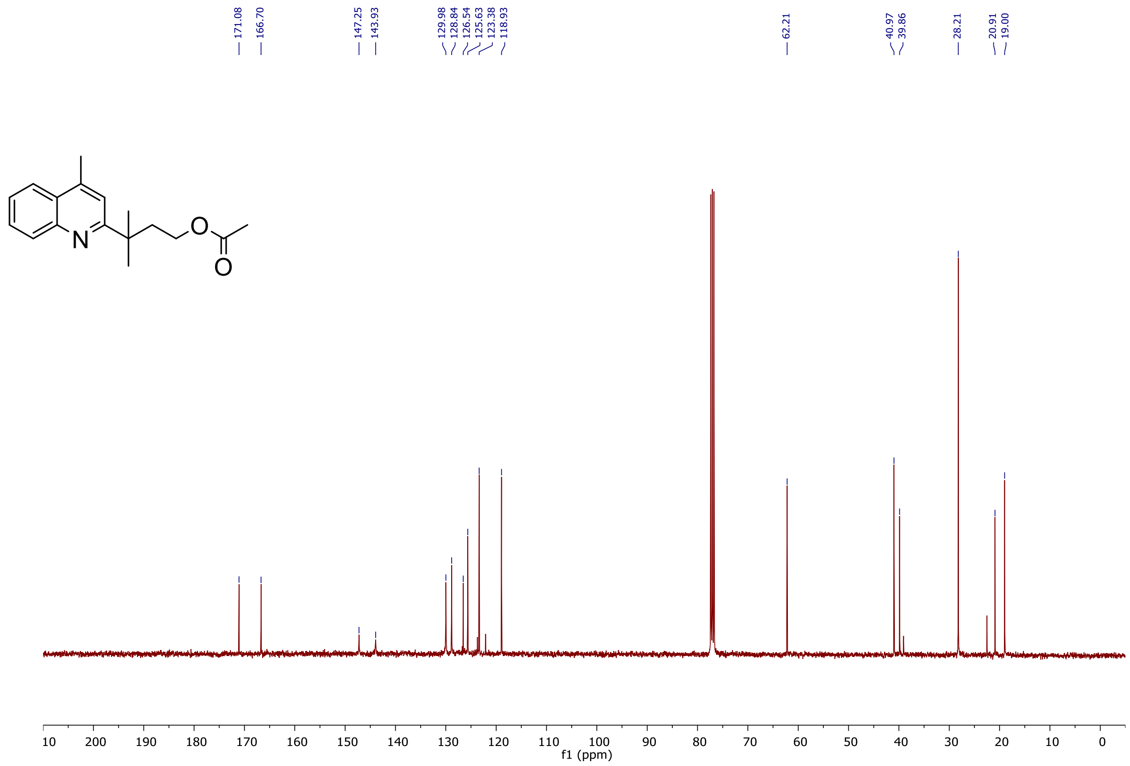

^{1}H NMR (400 MHz, $CDCl_3$) **34**

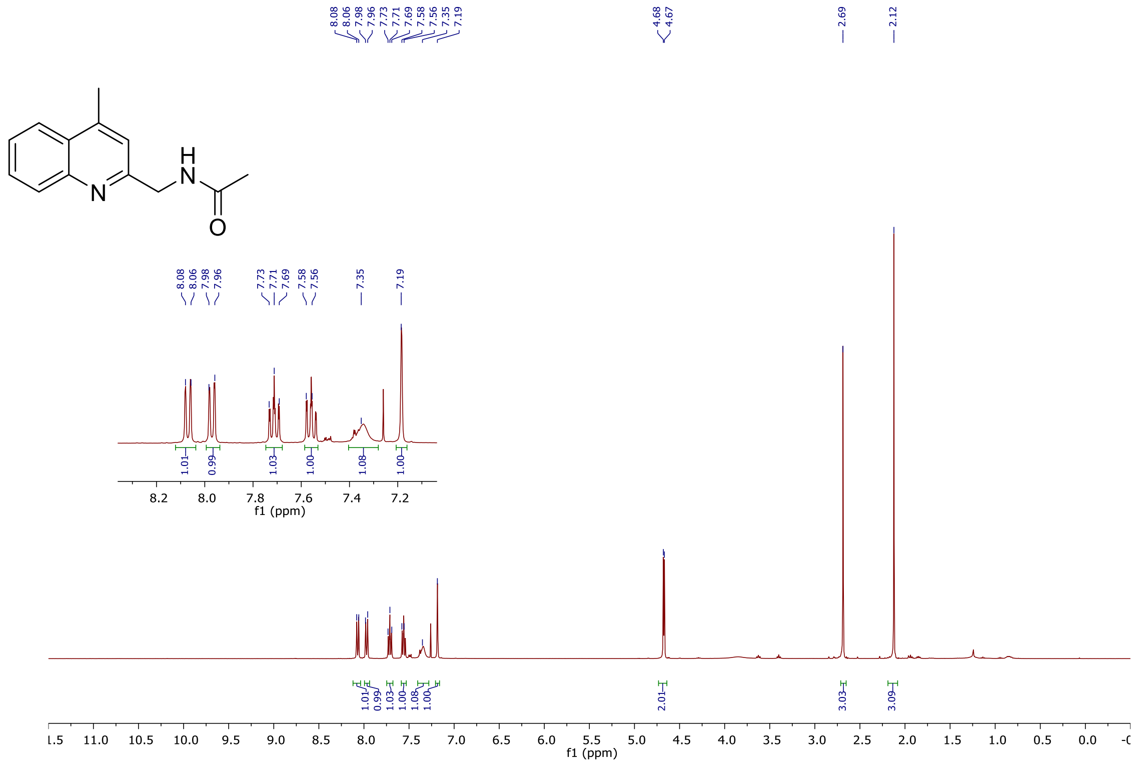

^{13}C NMR (101 MHz, $CDCl_3$) **34**

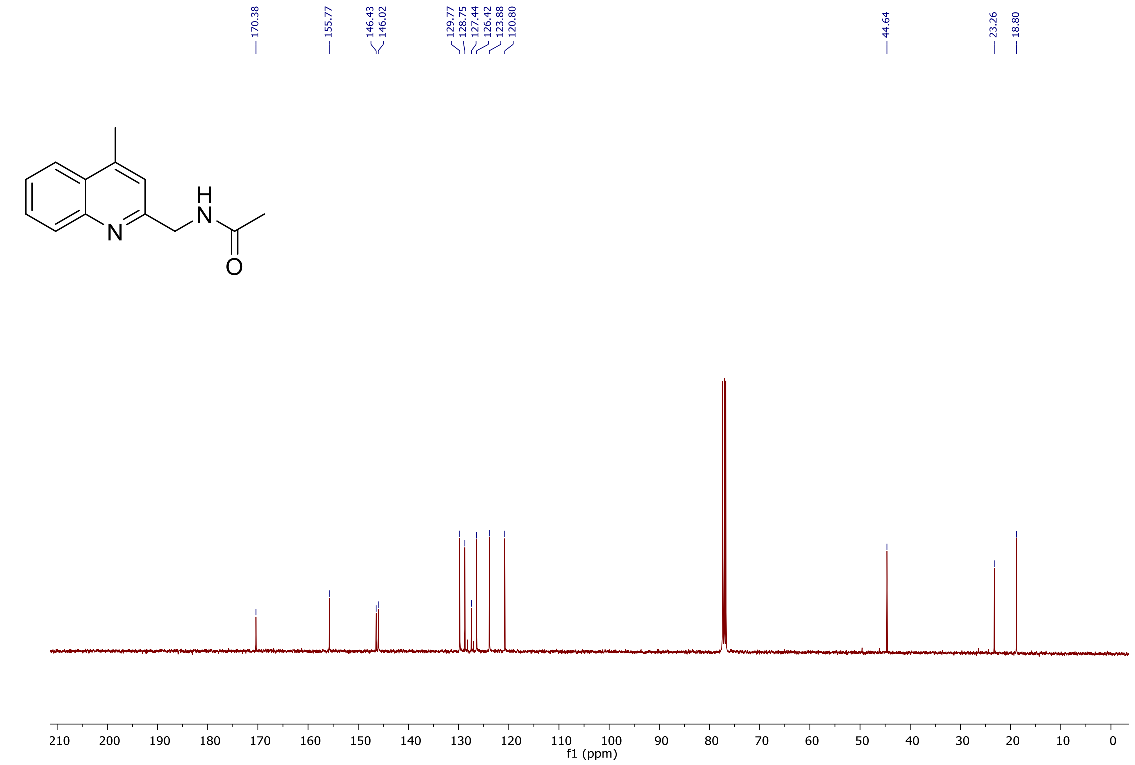

^{1}H NMR (400 MHz, $CDCl_3$) **35**

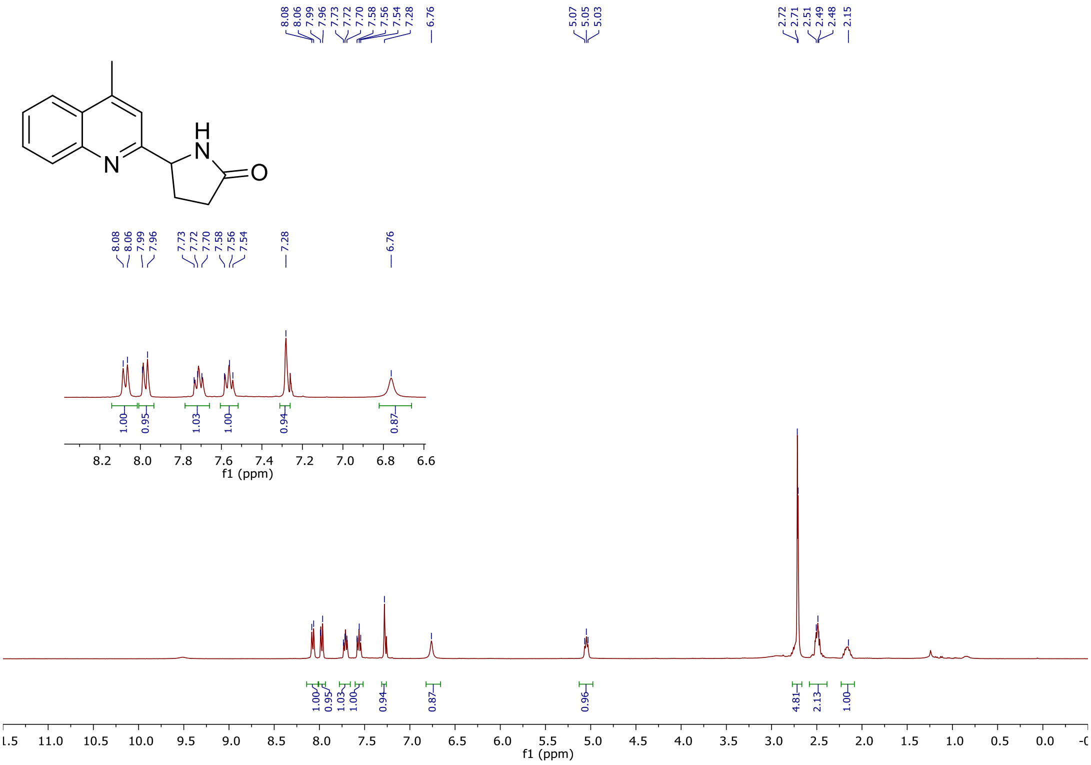

^{13}C NMR (101 MHz, $CDCl_3$) **35**

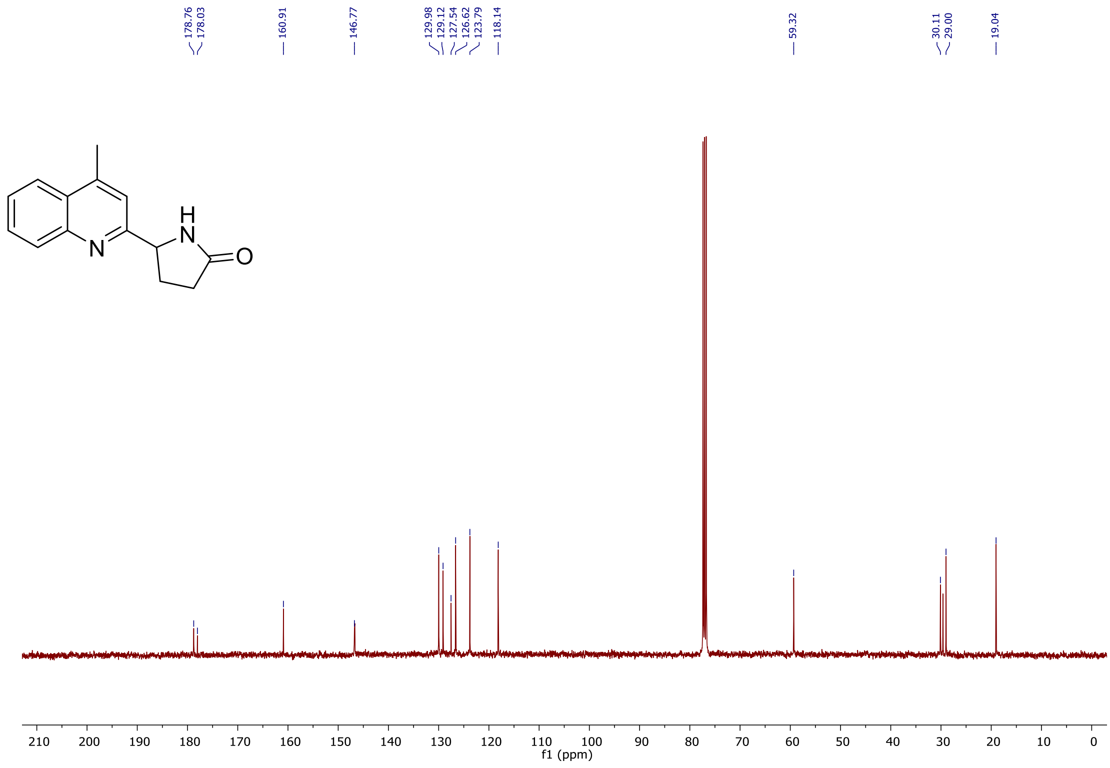

^{1}H NMR (300 MHz, $CDCl_3$) **36**

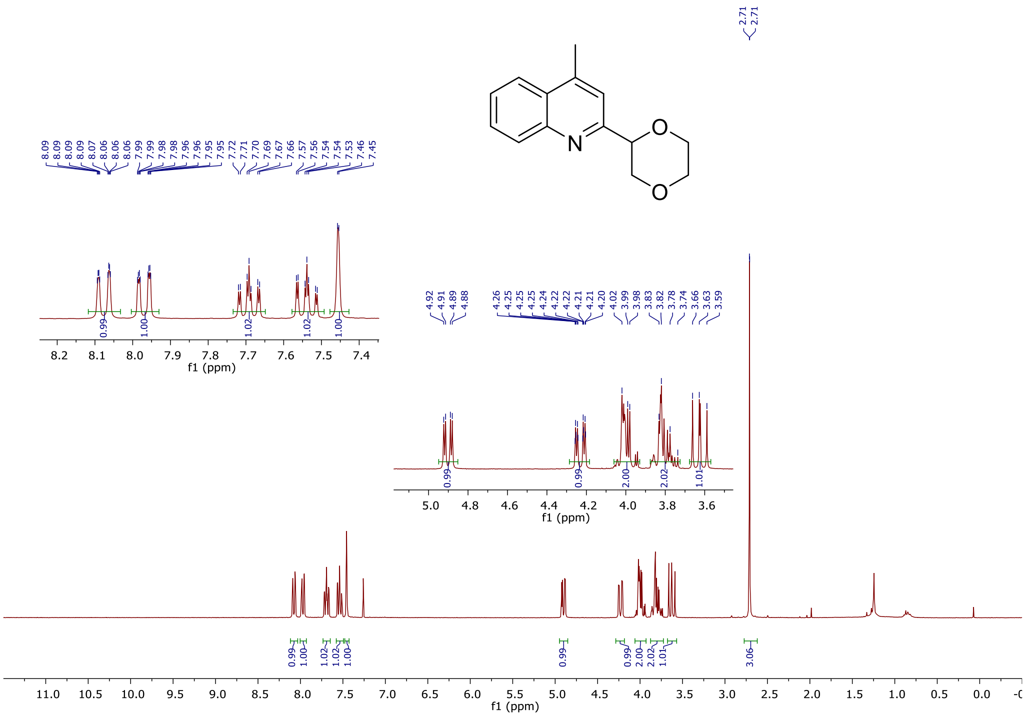

^{13}C NMR (101 MHz, $CDCl_3$) **36**

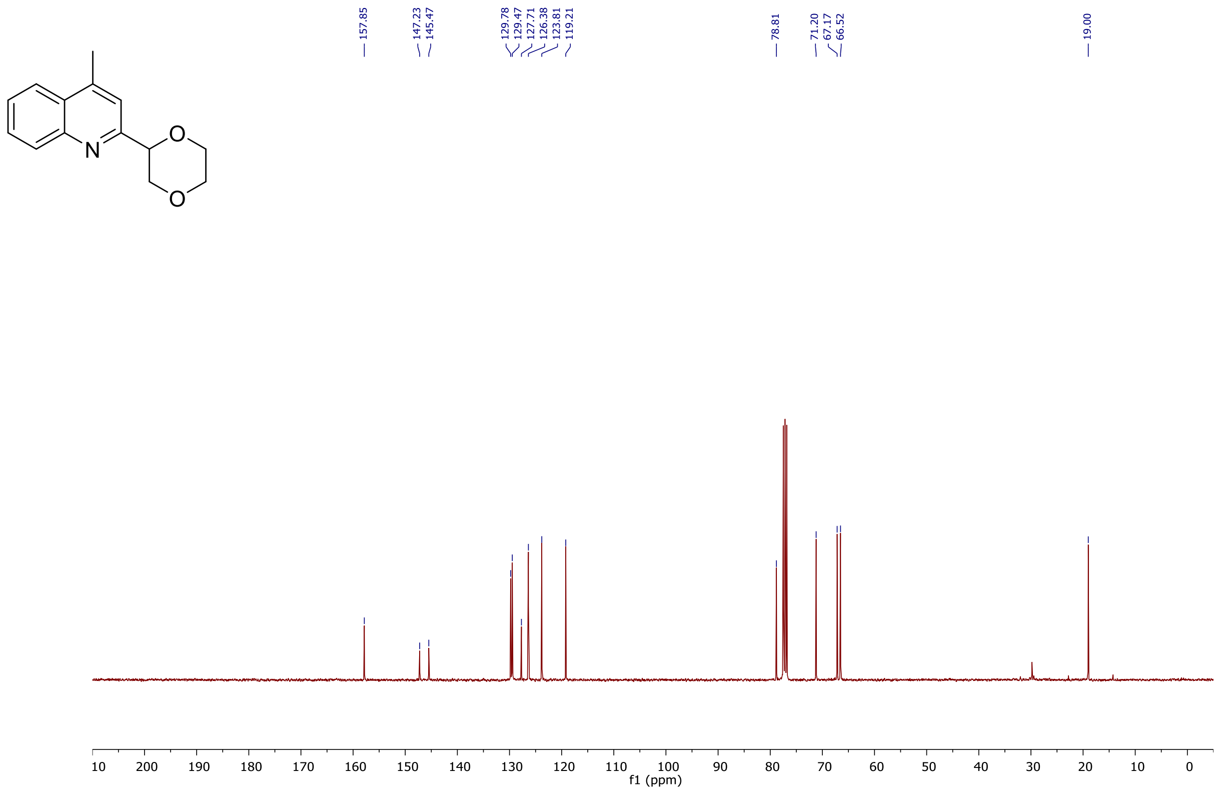

^{1}H NMR (400 MHz, $CDCl_3$) **37**

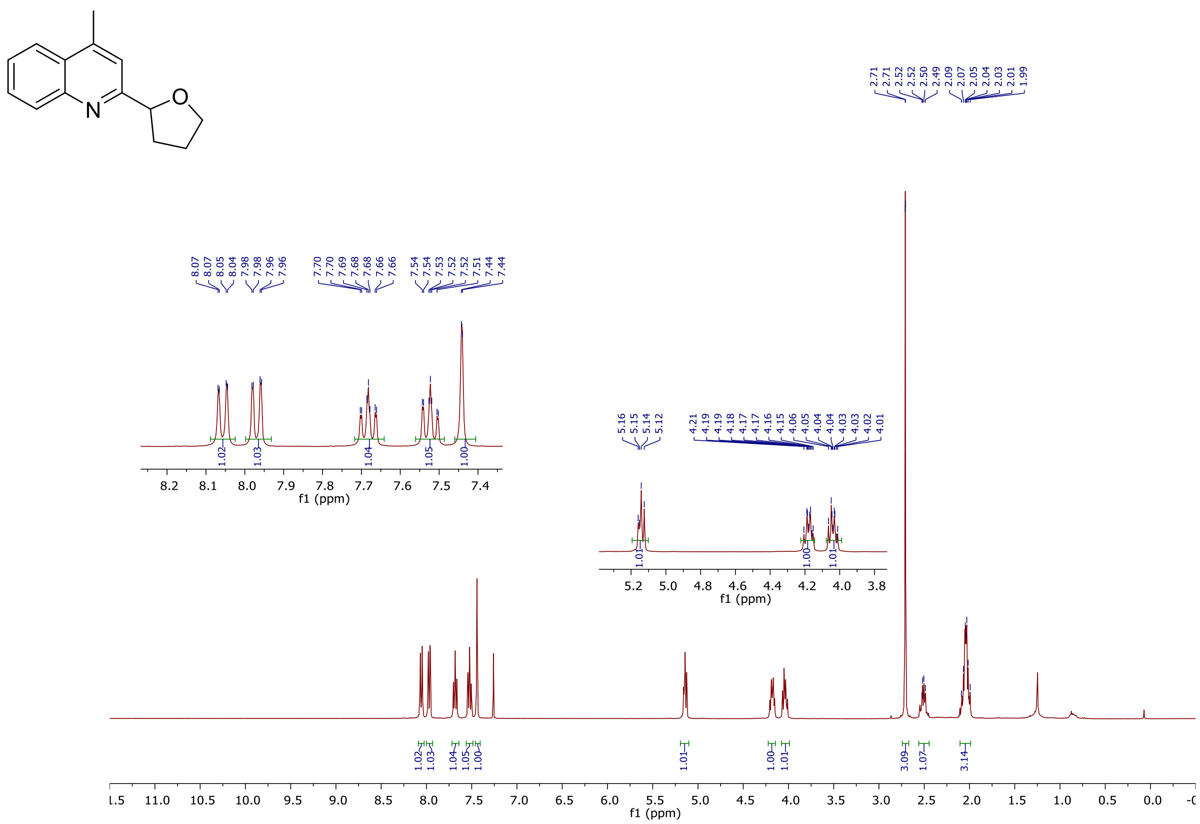

^{13}C NMR (101 MHz, $CDCl_3$) **37**

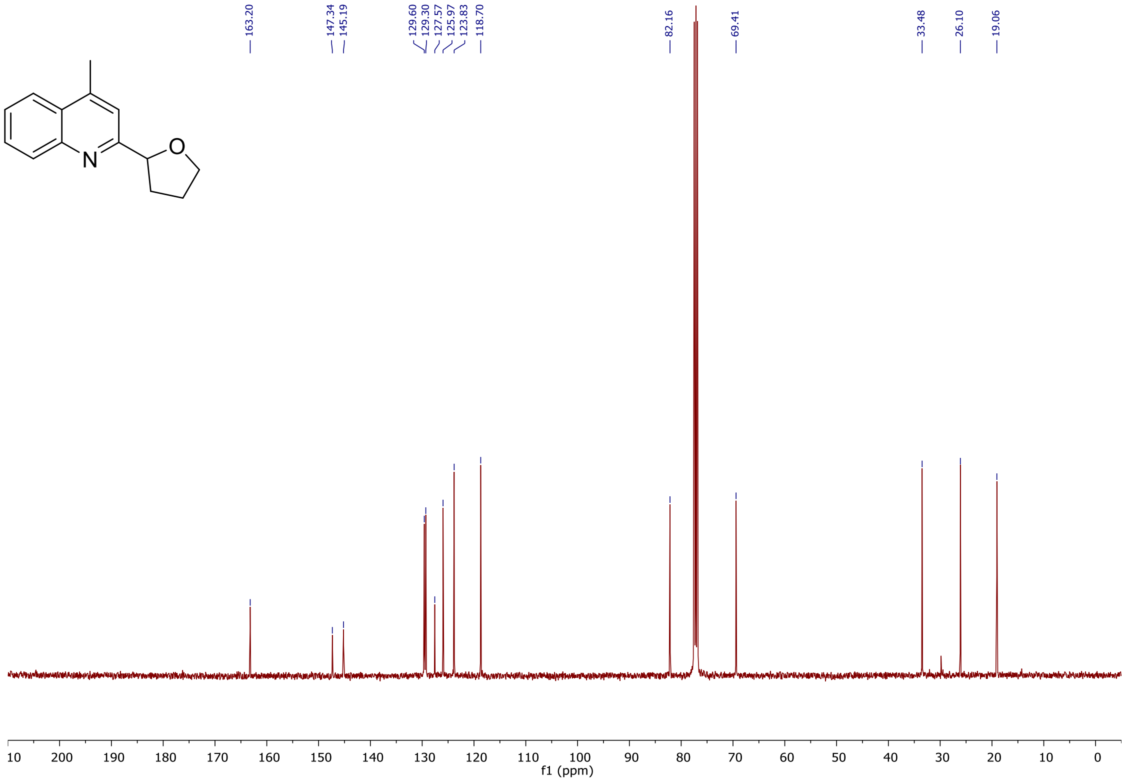

^{1}H NMR (400 MHz, $CDCl_3$) **38**

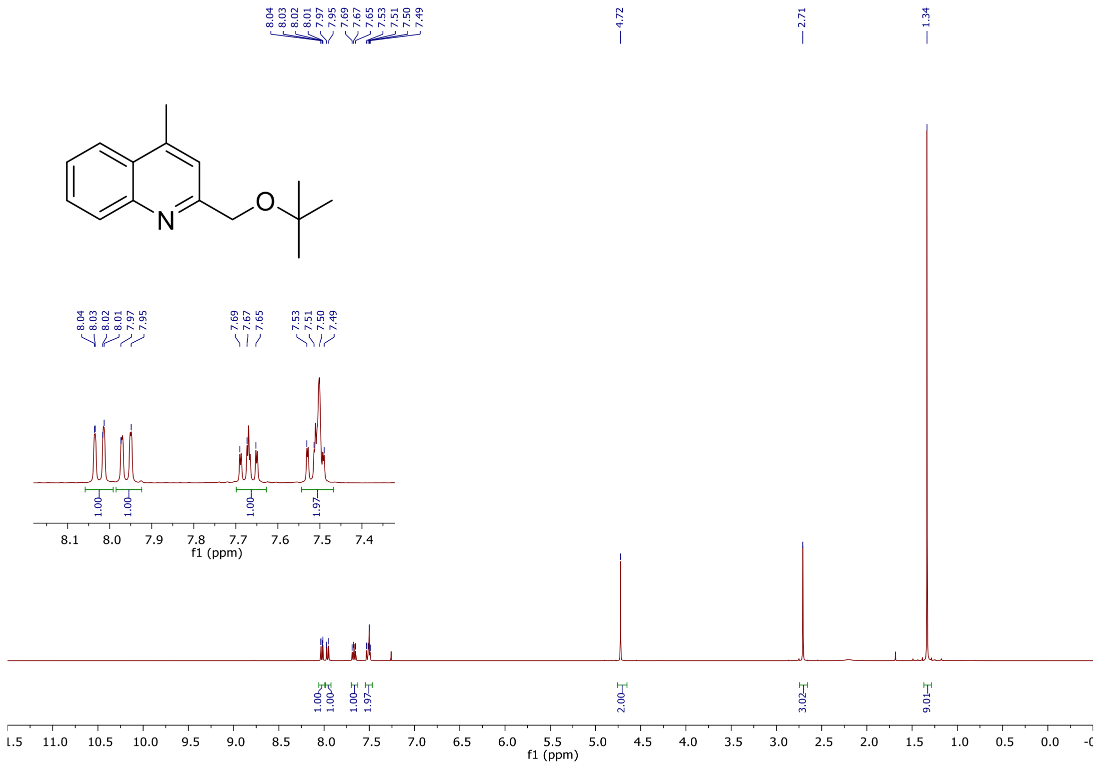

^{13}C NMR (101 MHz, $CDCl_3$) **38**

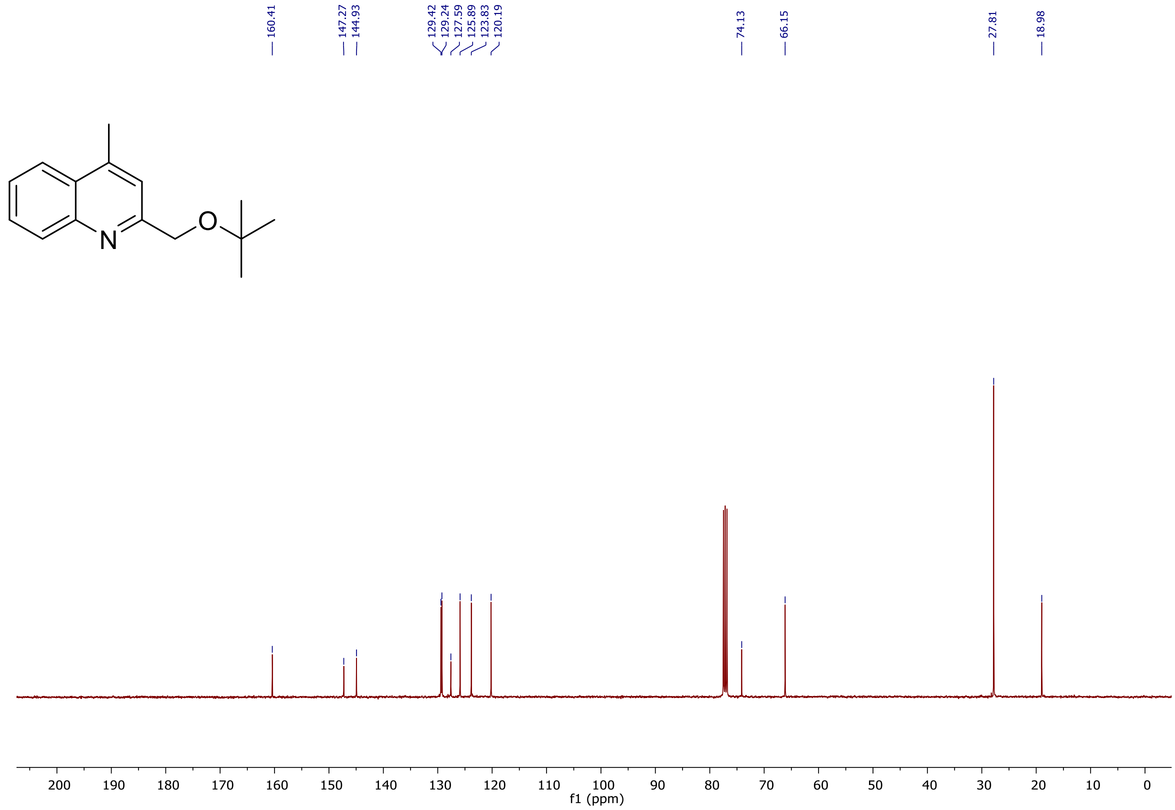

^{1}H NMR (400 MHz, $CDCl_3$) **39**

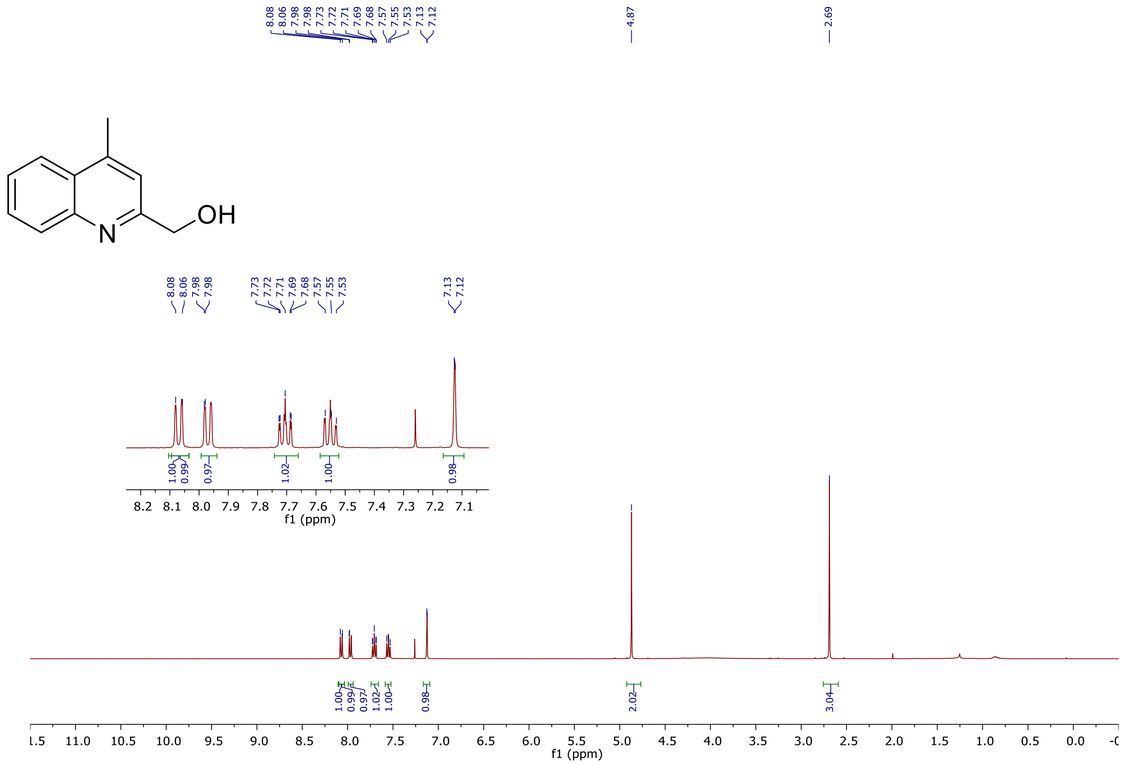

^{13}C NMR (101 MHz, $CDCl_3$) **39**

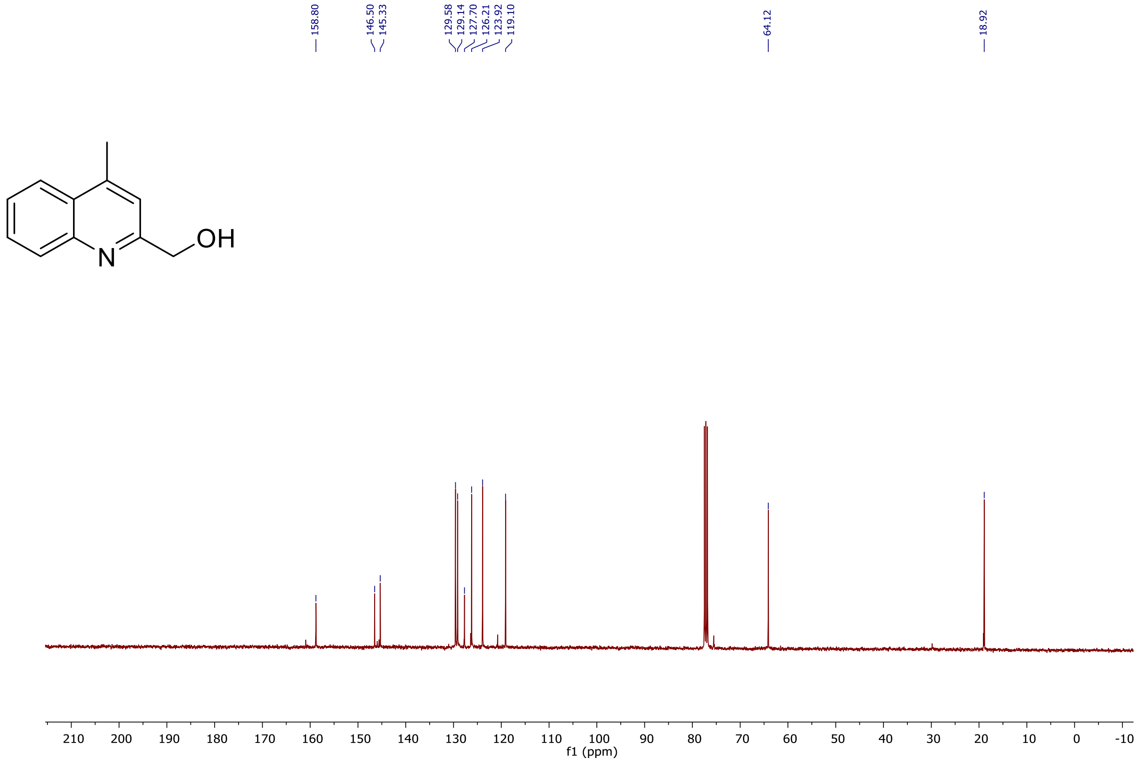

^{1}H NMR (400 MHz, $CDCl_3$) **40**

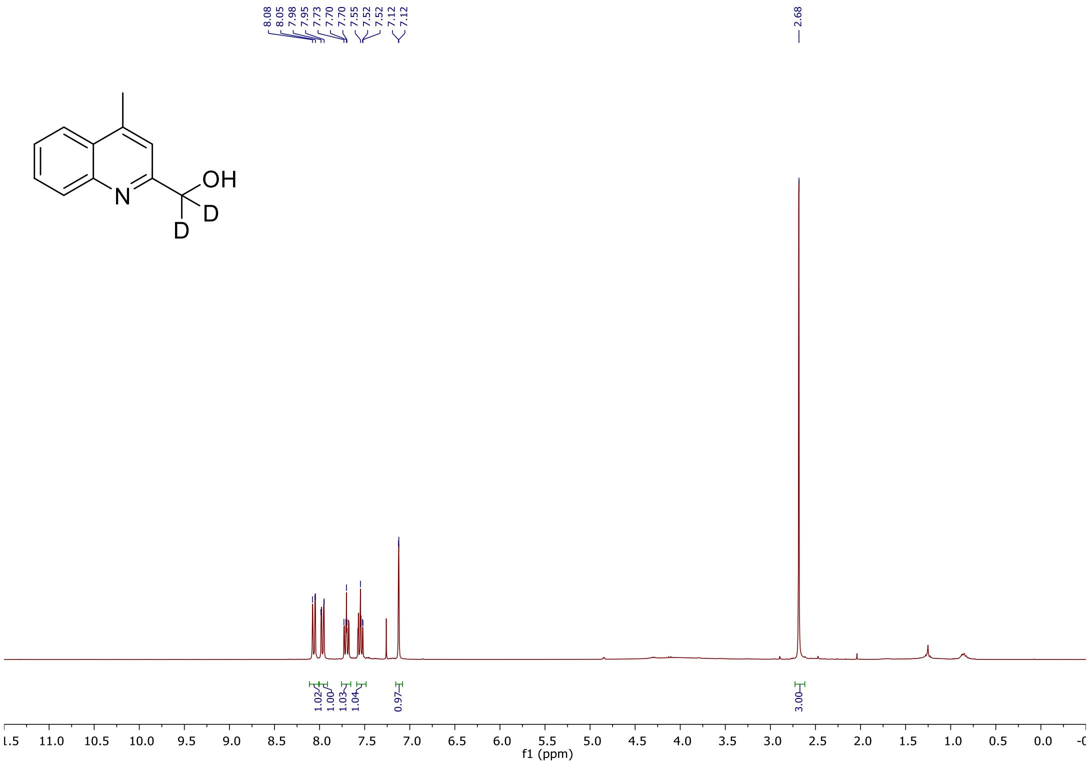

^{13}C NMR (101 MHz, $CDCl_3$) **40**

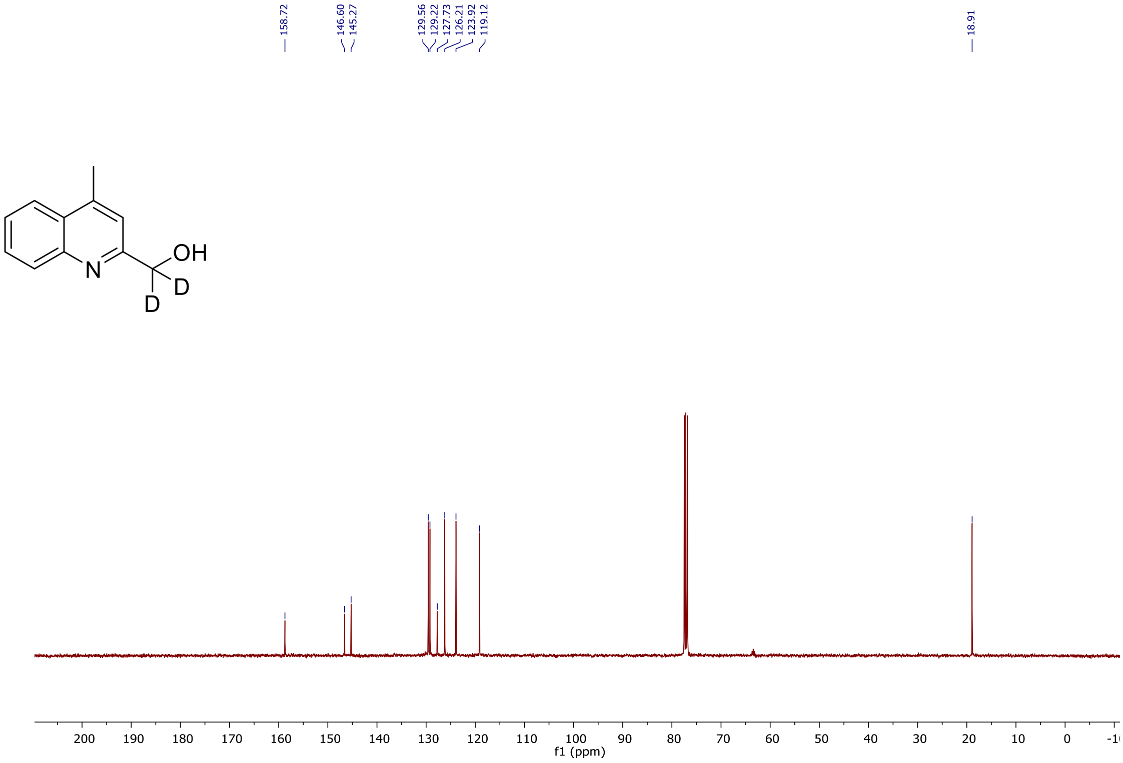

^{1}H NMR (400 MHz, $CDCl_3$) **41**

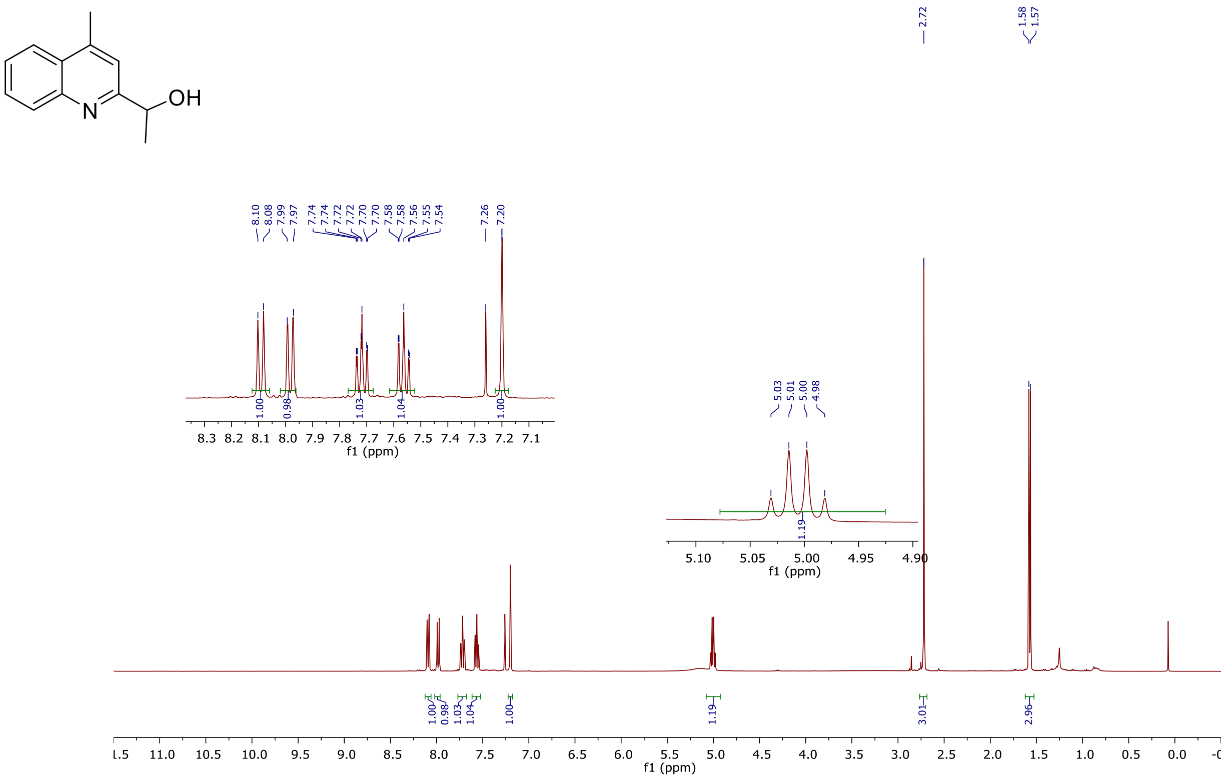

^{13}C NMR (101 MHz, $CDCl_3$) **41**

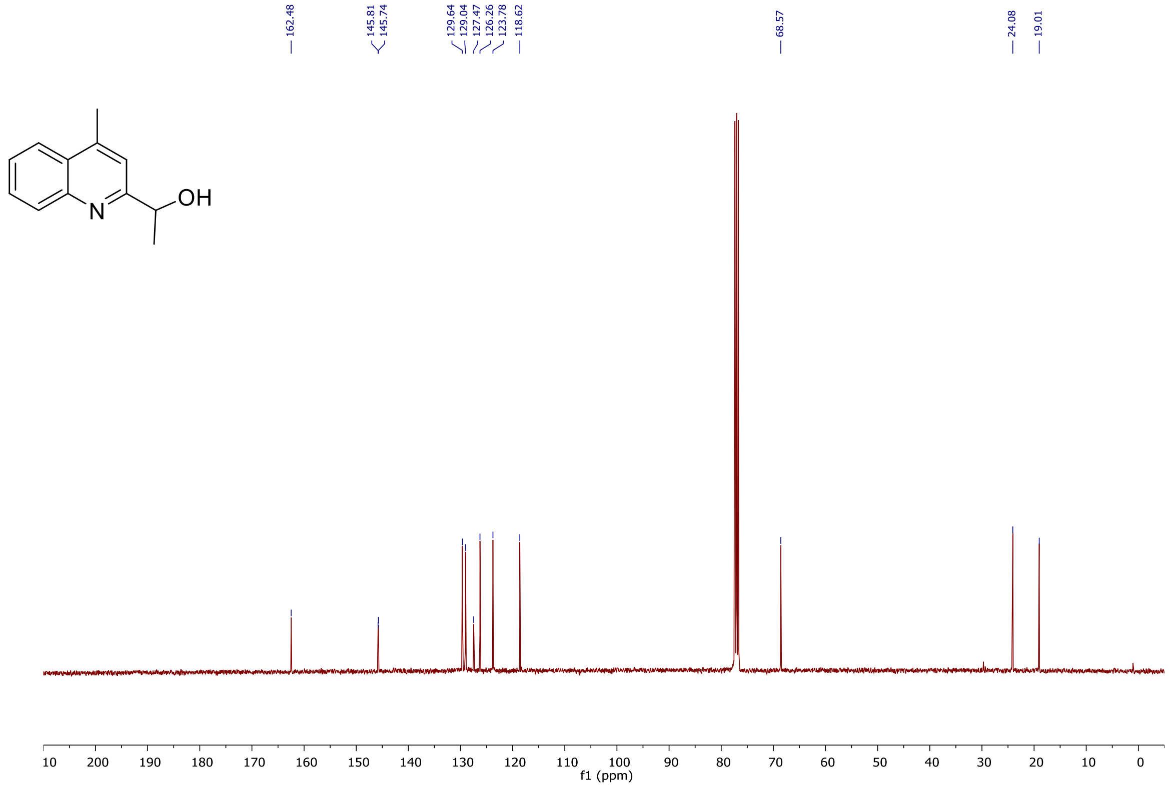

^{1}H NMR (400 MHz, $CDCl_3$) **42a+42b**

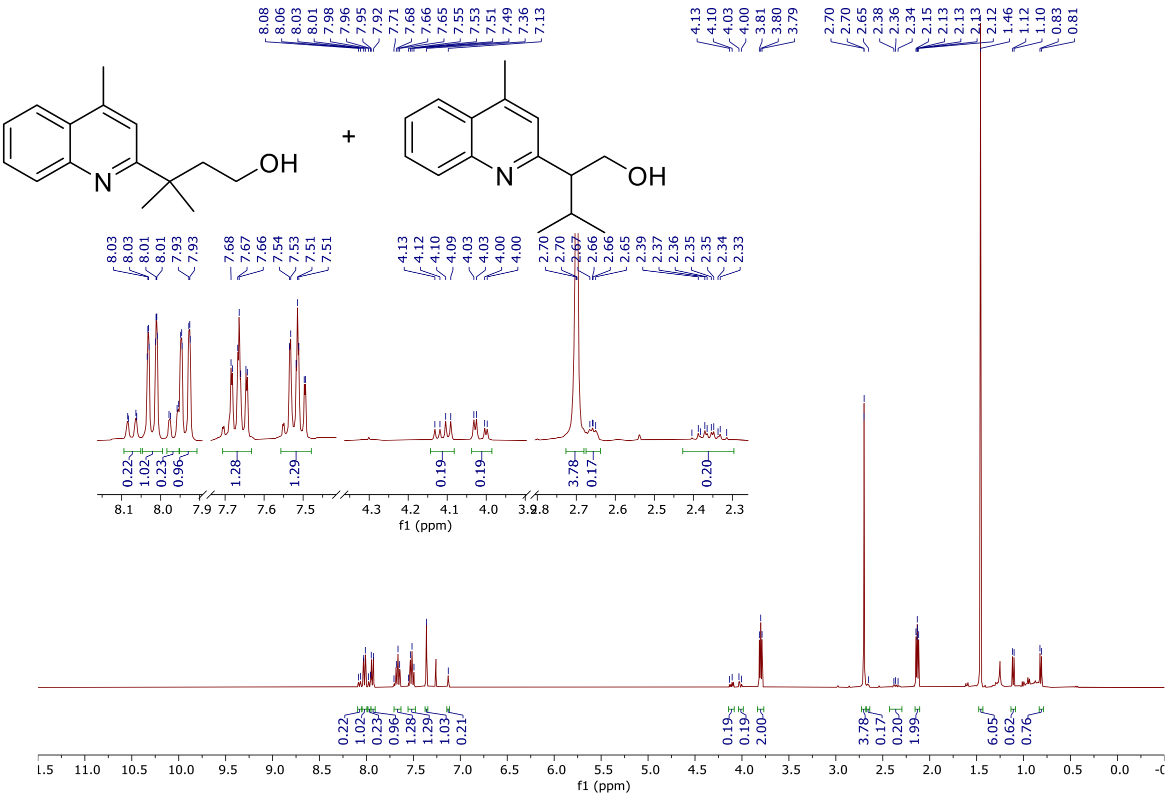

^{13}C NMR (101 MHz, $CDCl_3$) **42a+42b**

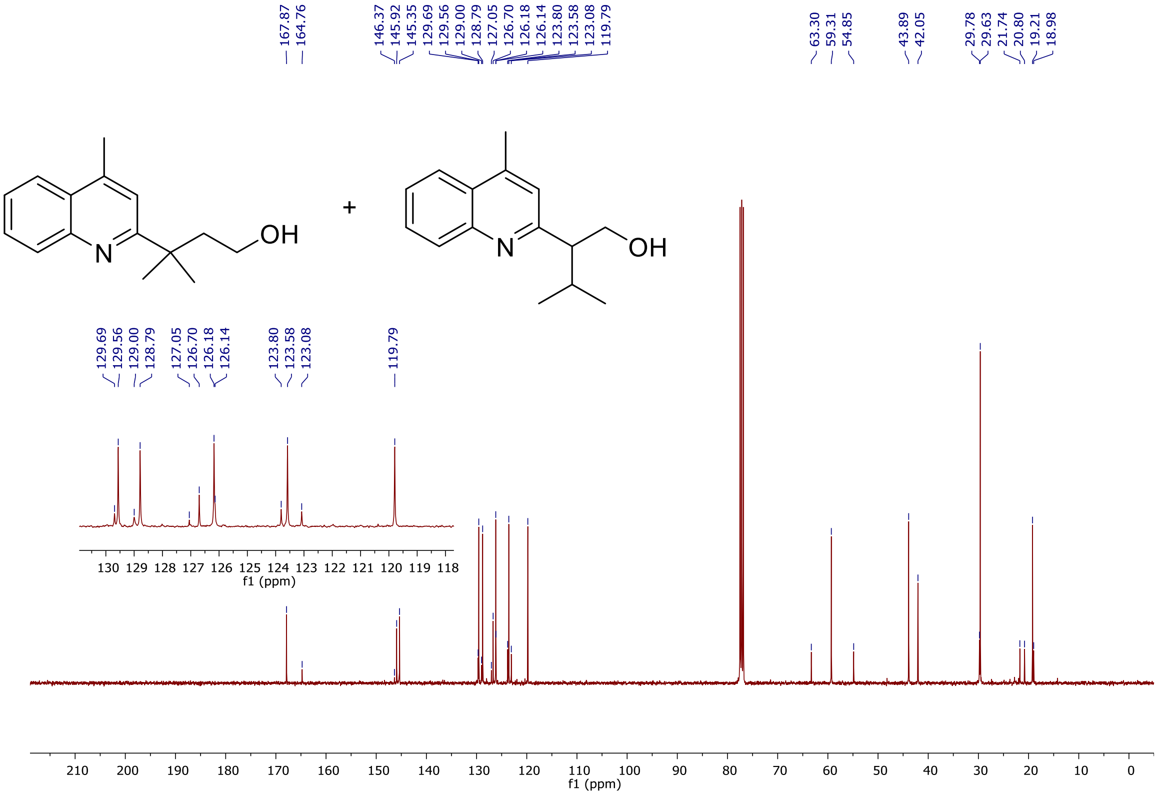

^{1}H NMR (400 MHz, $CDCl_3$) **43**

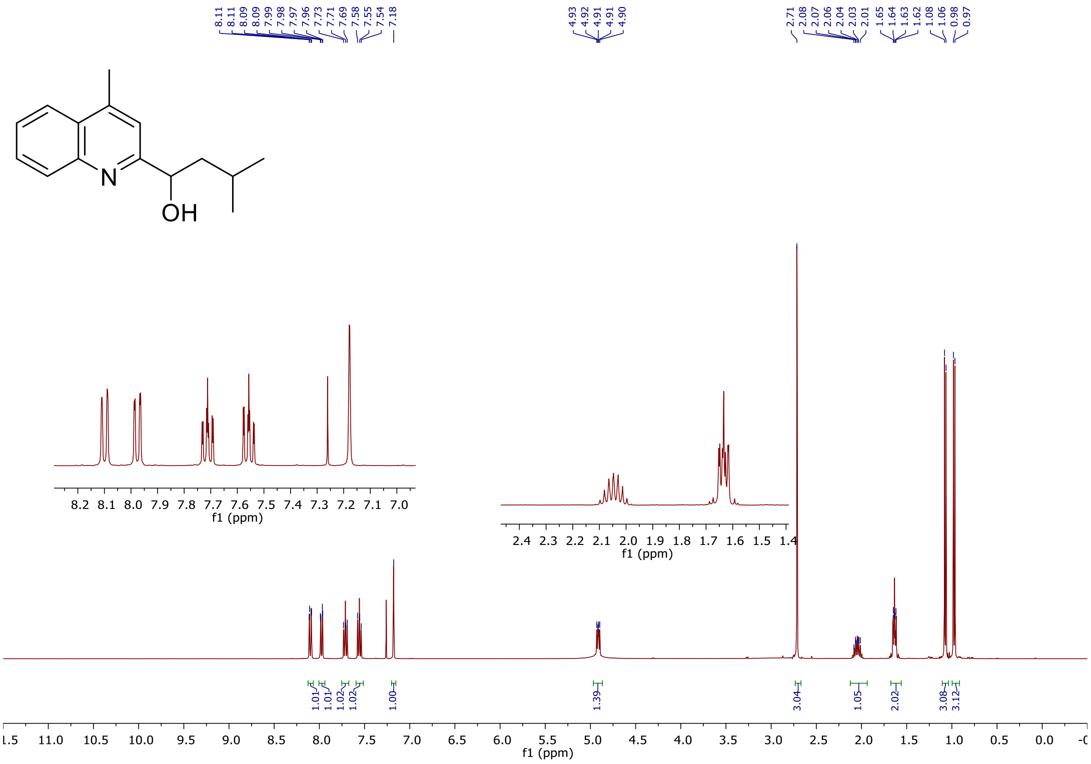

^{13}C NMR (101 MHz, $CDCl_3$) **43**

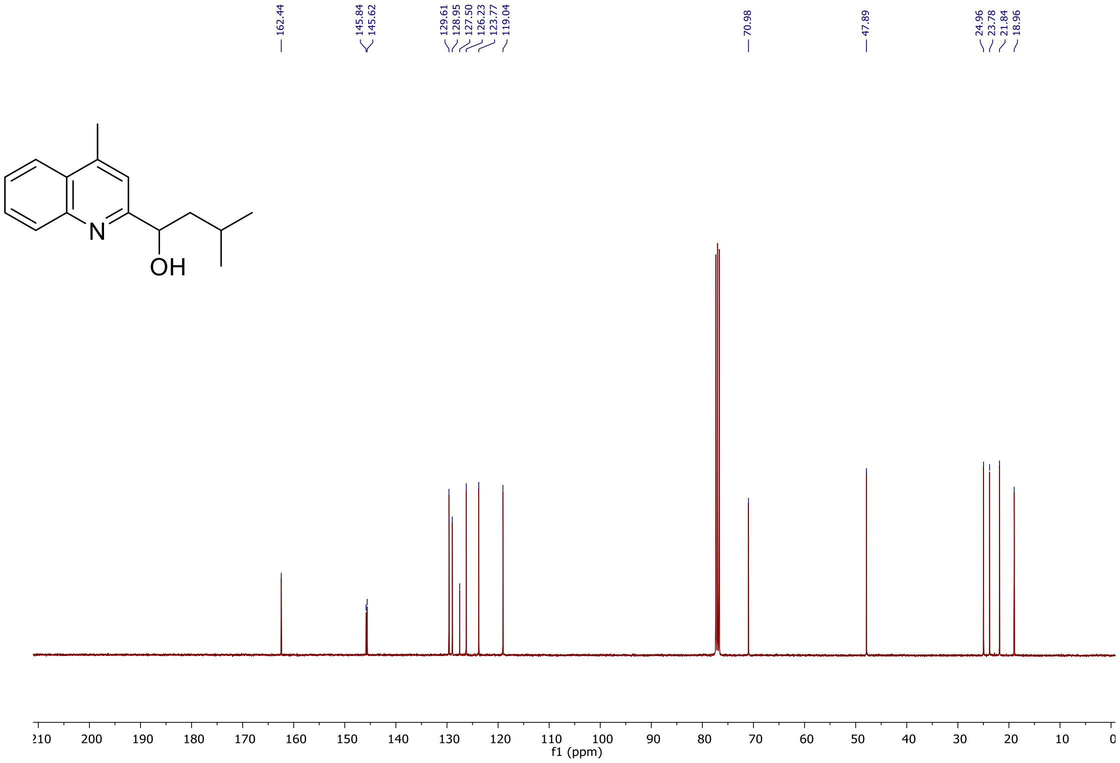

^{1}H NMR (400 MHz, $CDCl_3$) **44**

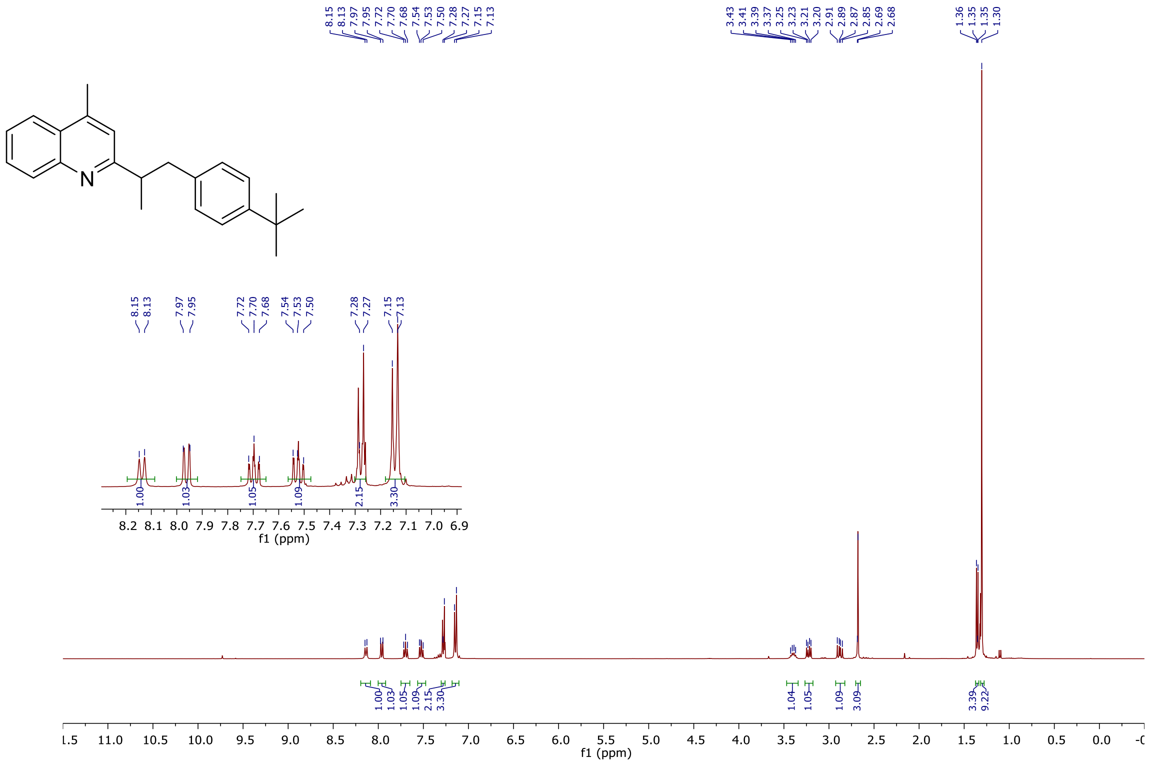

^{13}C NMR (101 MHz, $CDCl_3$) **44**

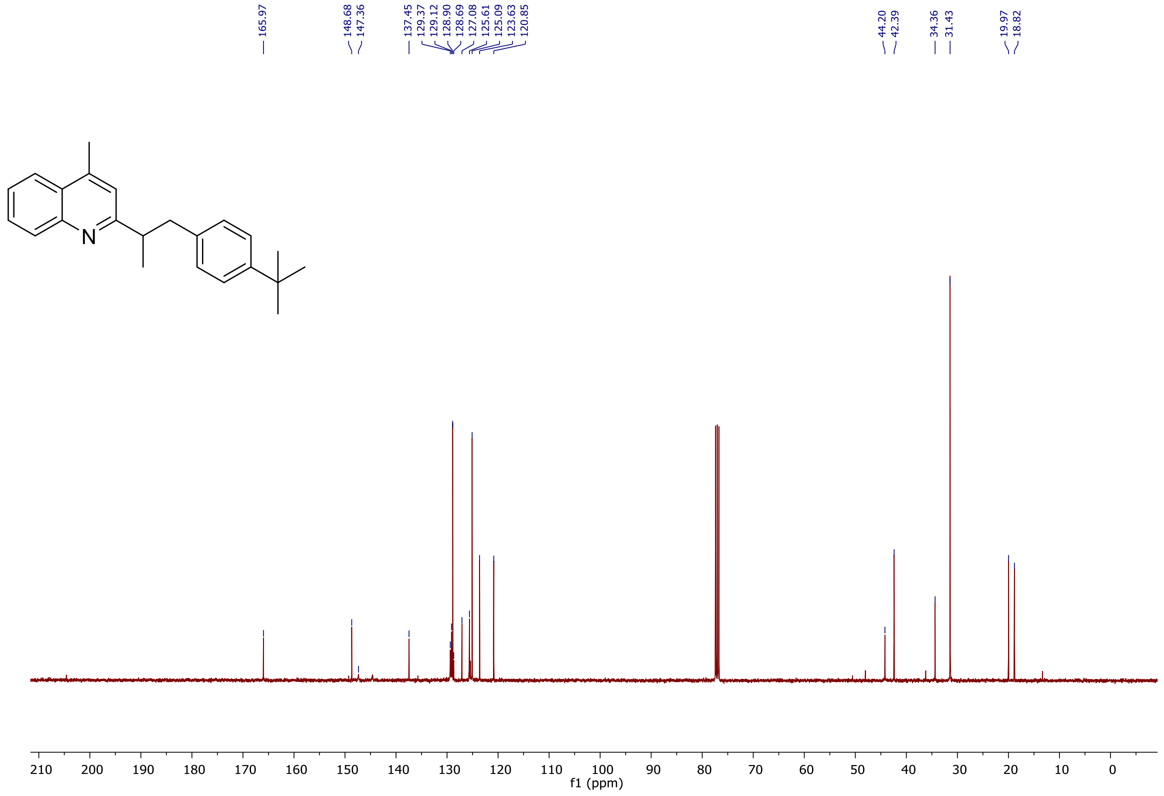

^{1}H NMR (400 MHz, $CDCl_3$) **45**

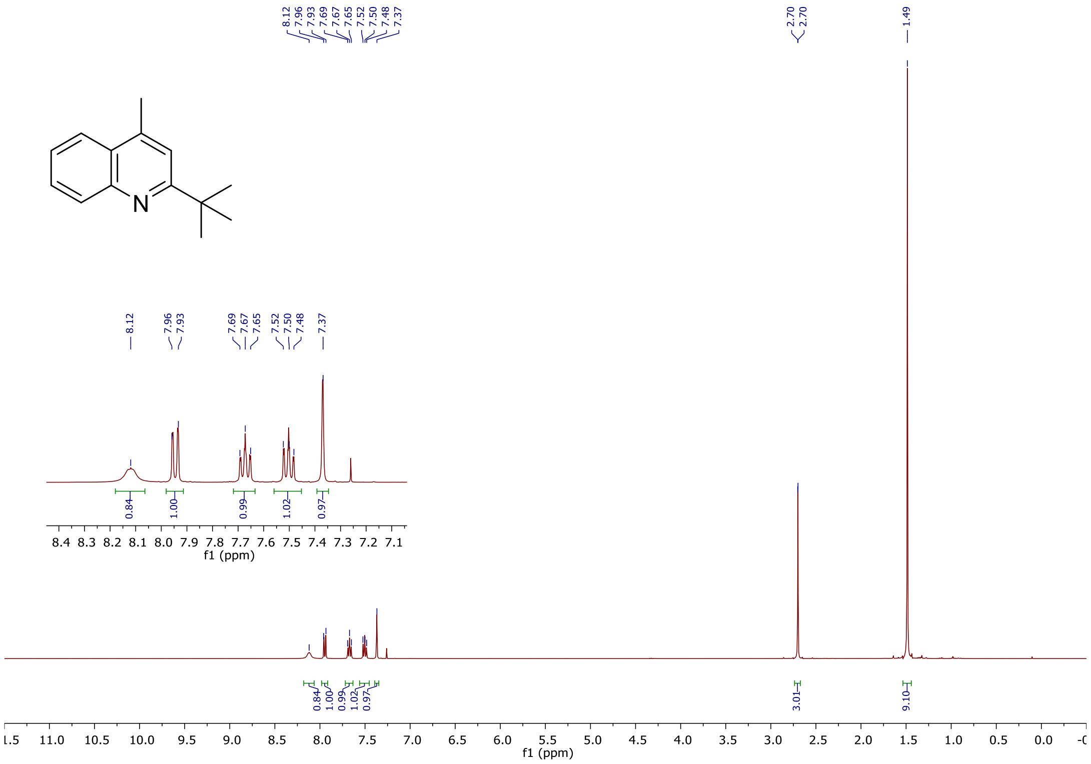

^{13}C NMR (101 MHz, $CDCl_3$) **45**

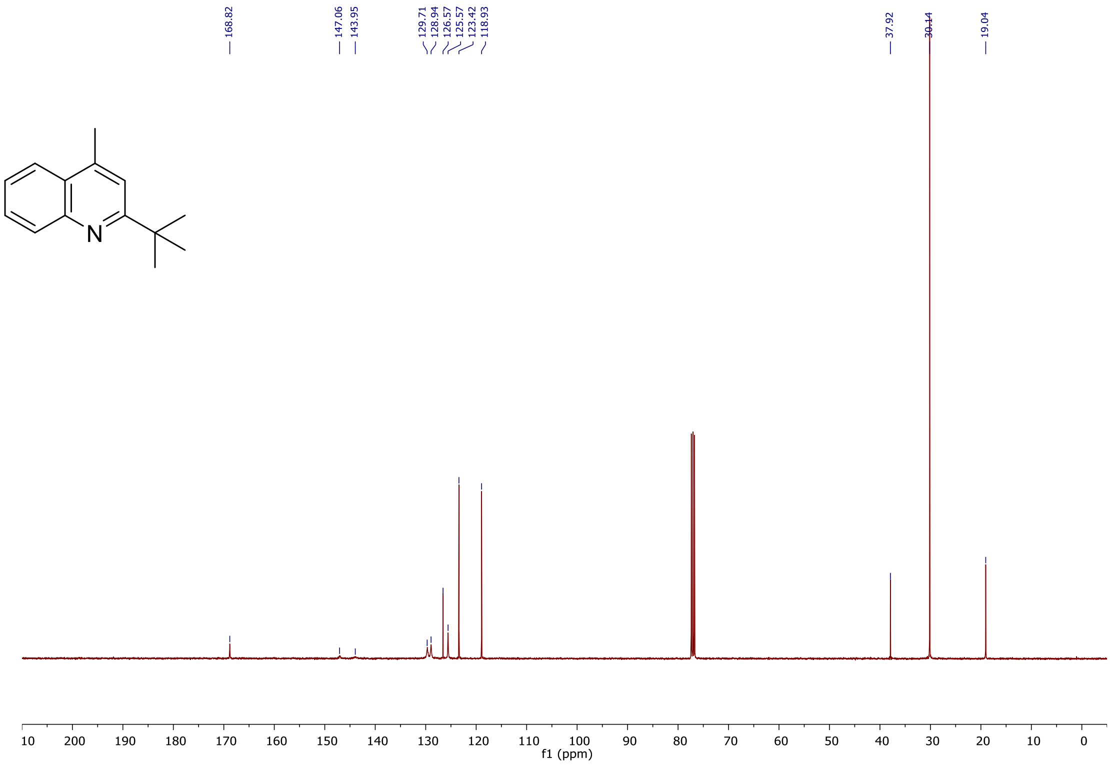

^{1}H NMR (400 MHz, $CDCl_3$) **46**

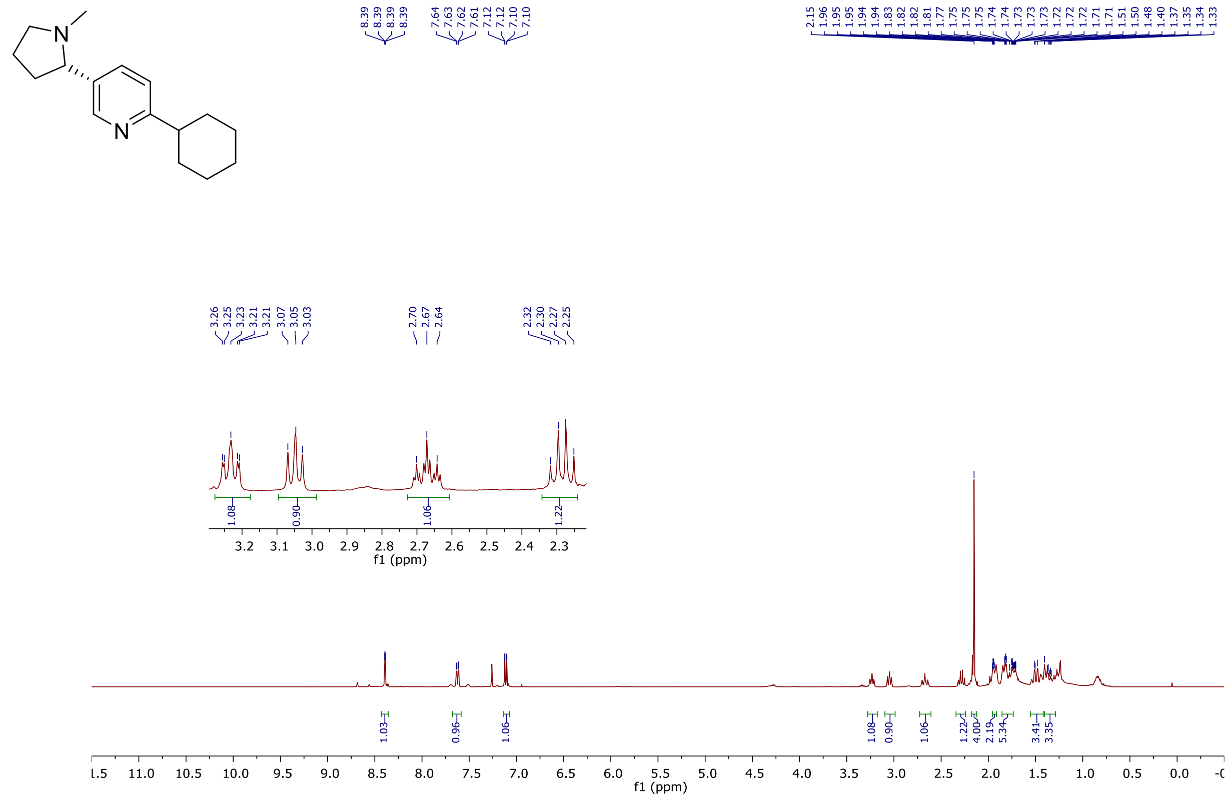

^{13}C NMR (101 MHz, $CDCl_3$) **46**

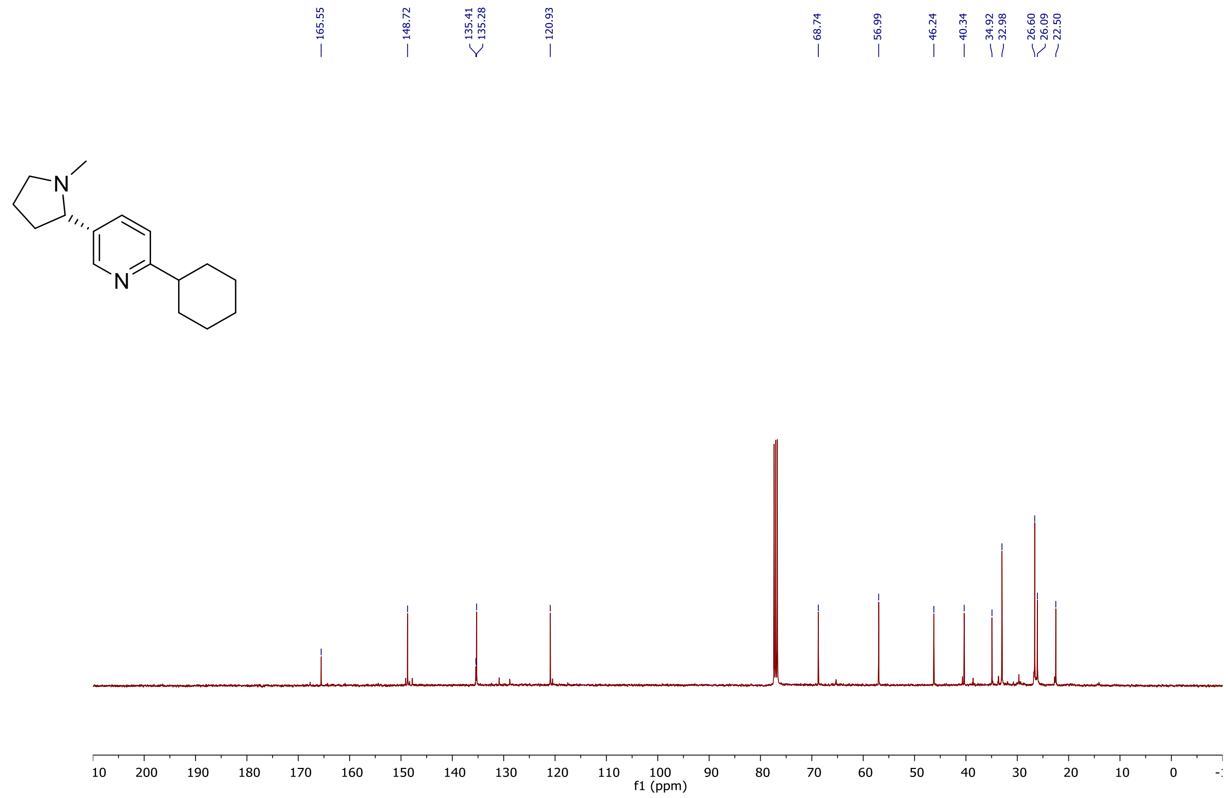

^{1}H NMR (300 MHz, $CDCl_3$) **47**

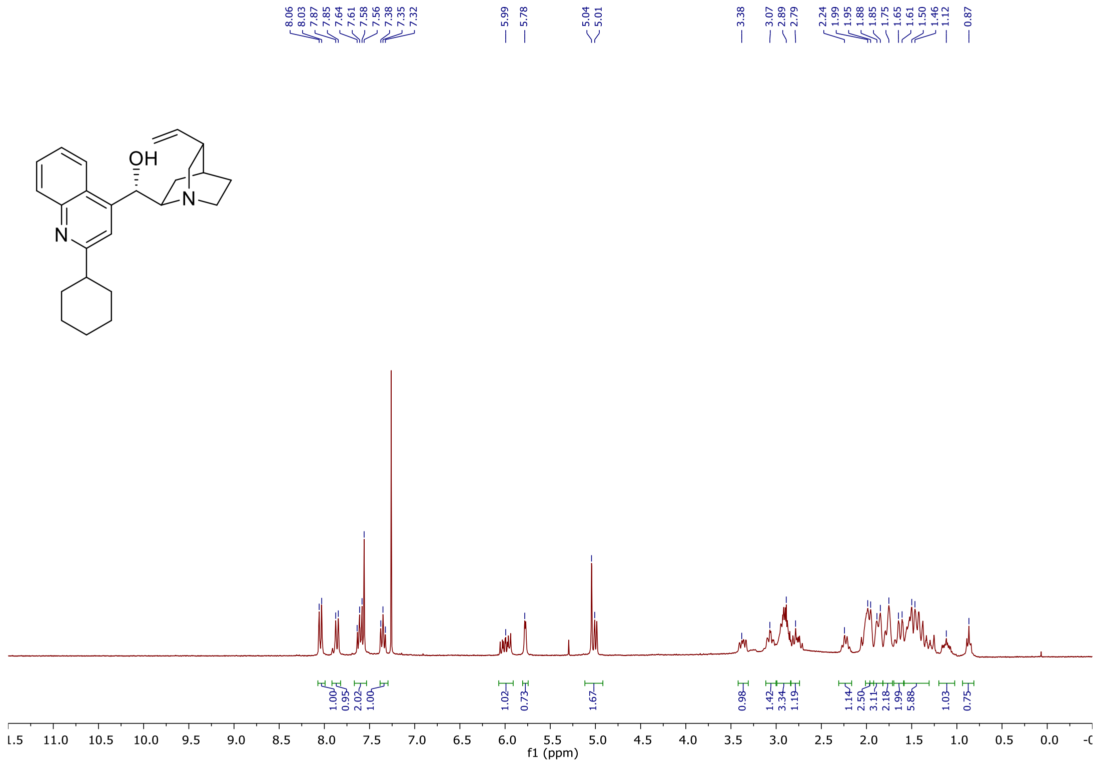

^{13}C NMR (126 MHz, $CDCl_3$) **47**

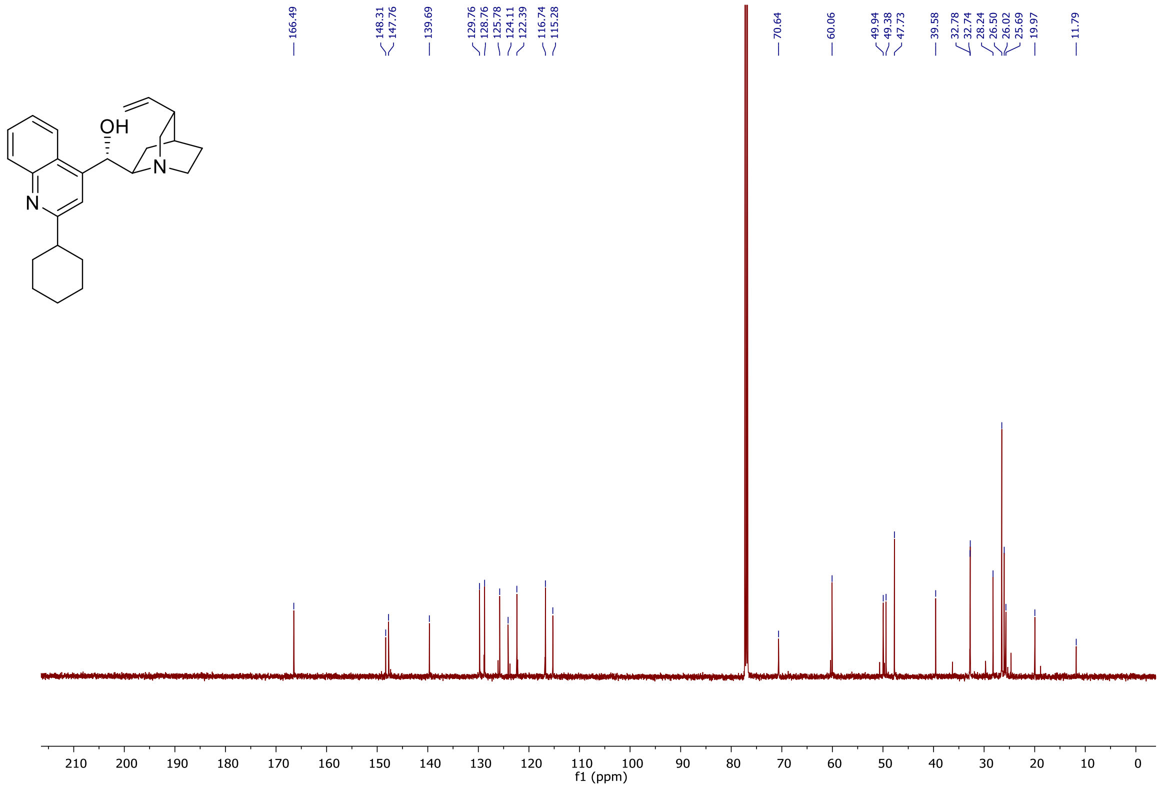

^{1}H NMR (400 MHz, CDCl$_3$) **48**

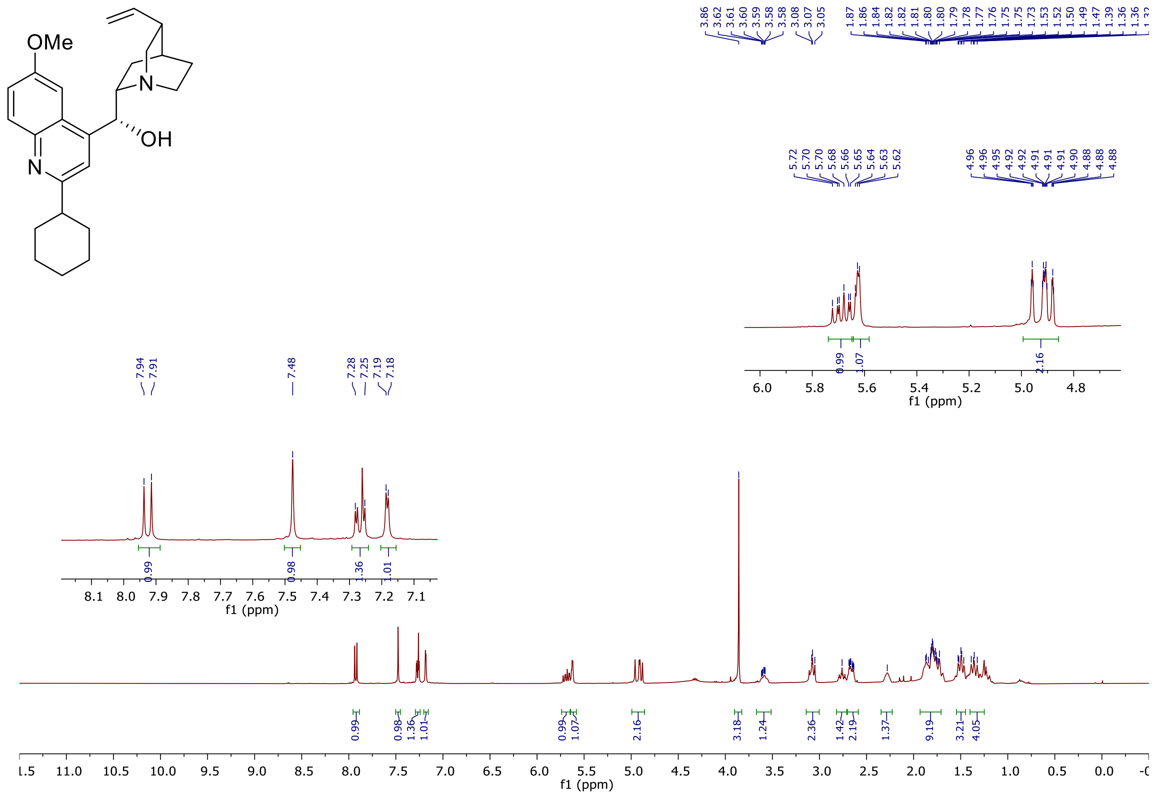

^{13}C NMR (101 MHz, CDCl$_3$) **48**

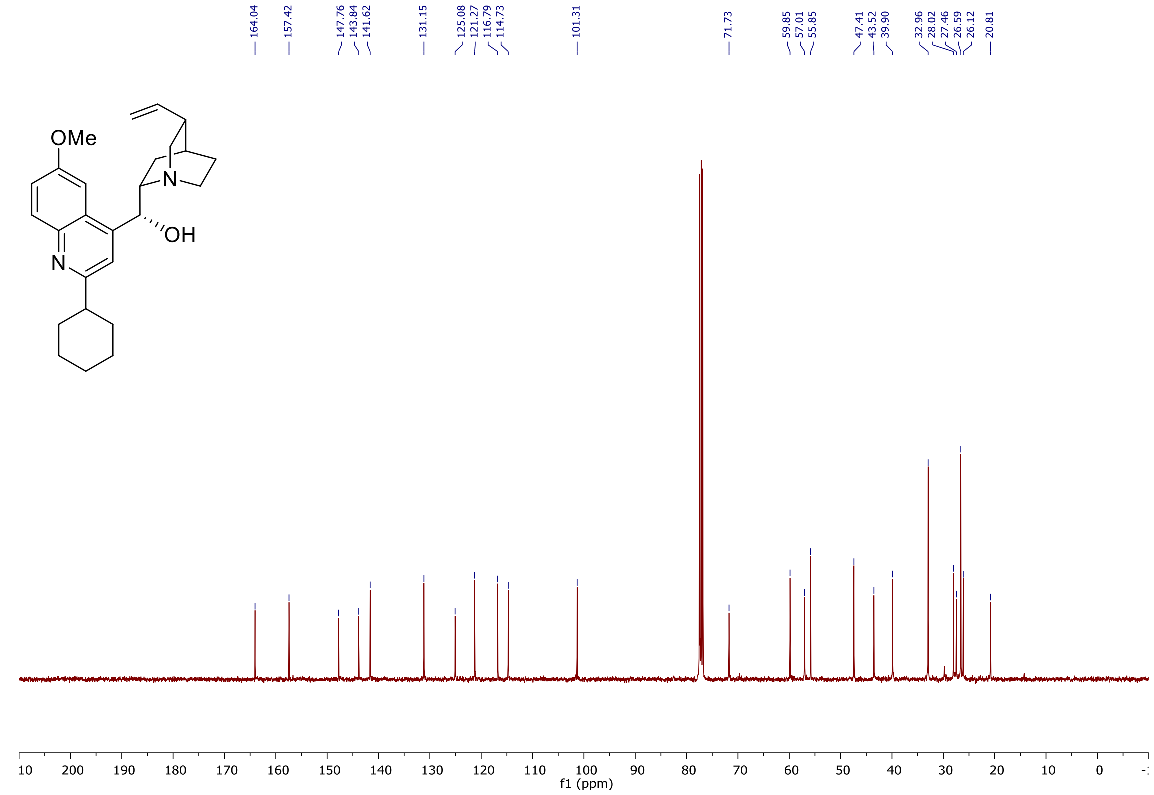

^{1}H NMR (400 MHz, $CDCl_3$) **49**

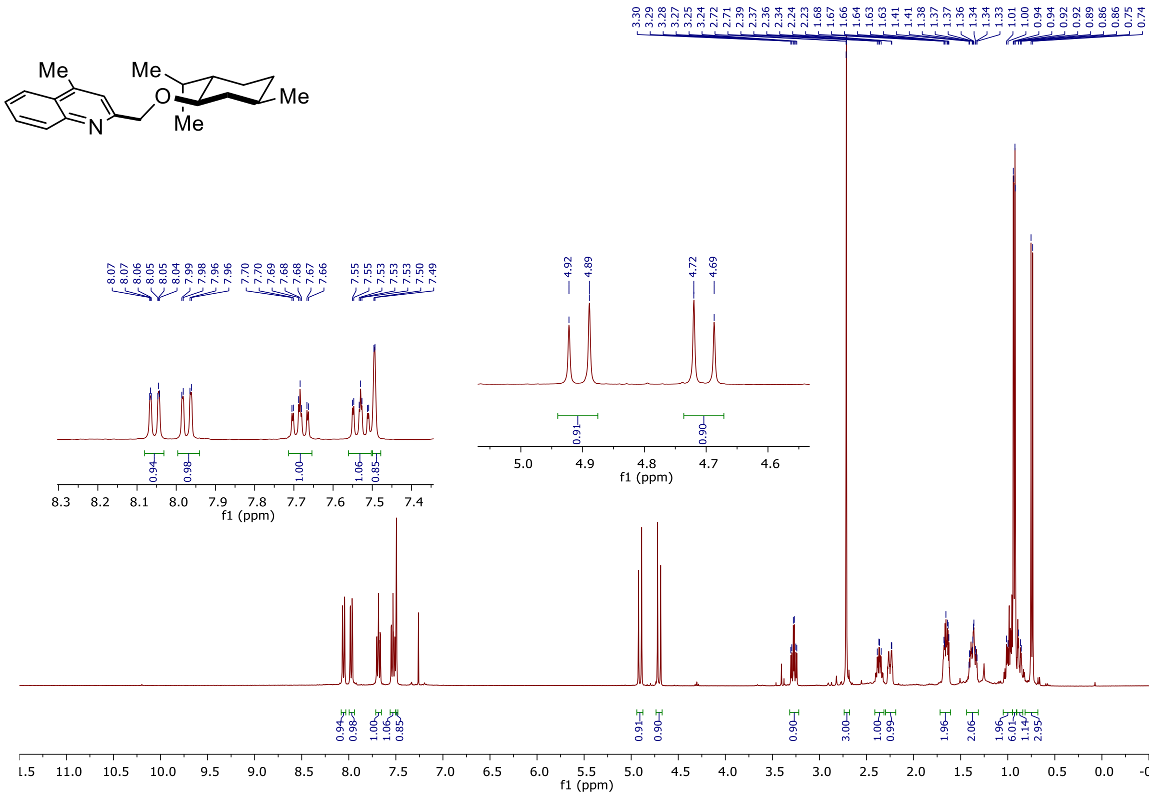

^{13}C NMR(101MHz,$CDCl_3$) **49**

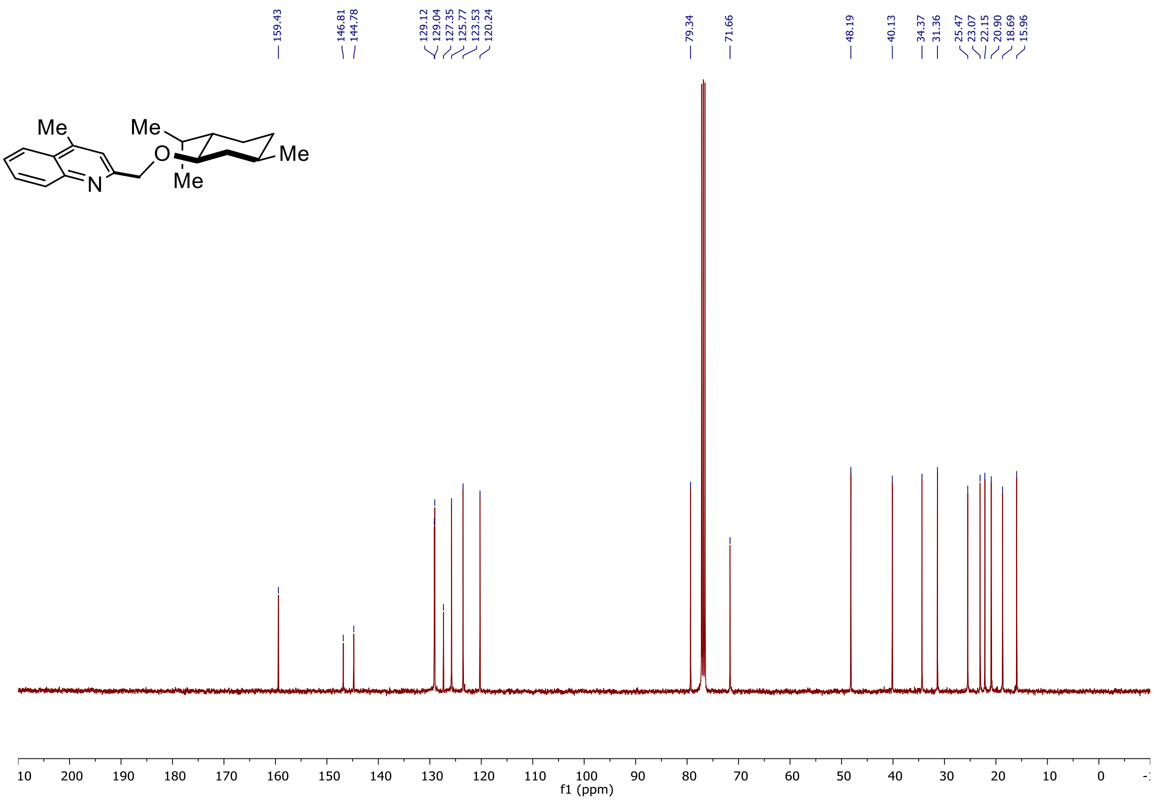

^{1}H NMR (400 MHz, $CDCl_3$) **50**

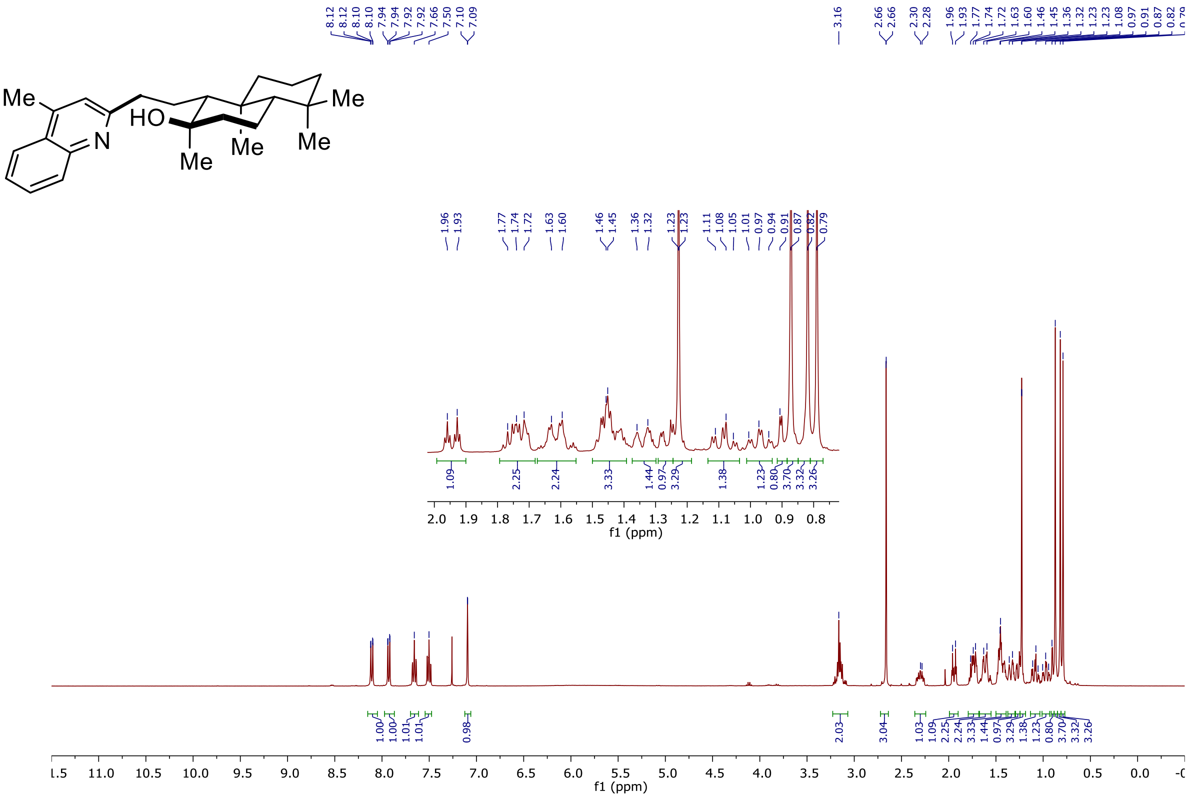

^{13}C NMR + DEPT-135 (101 MHz, $CDCl_3$) **50**

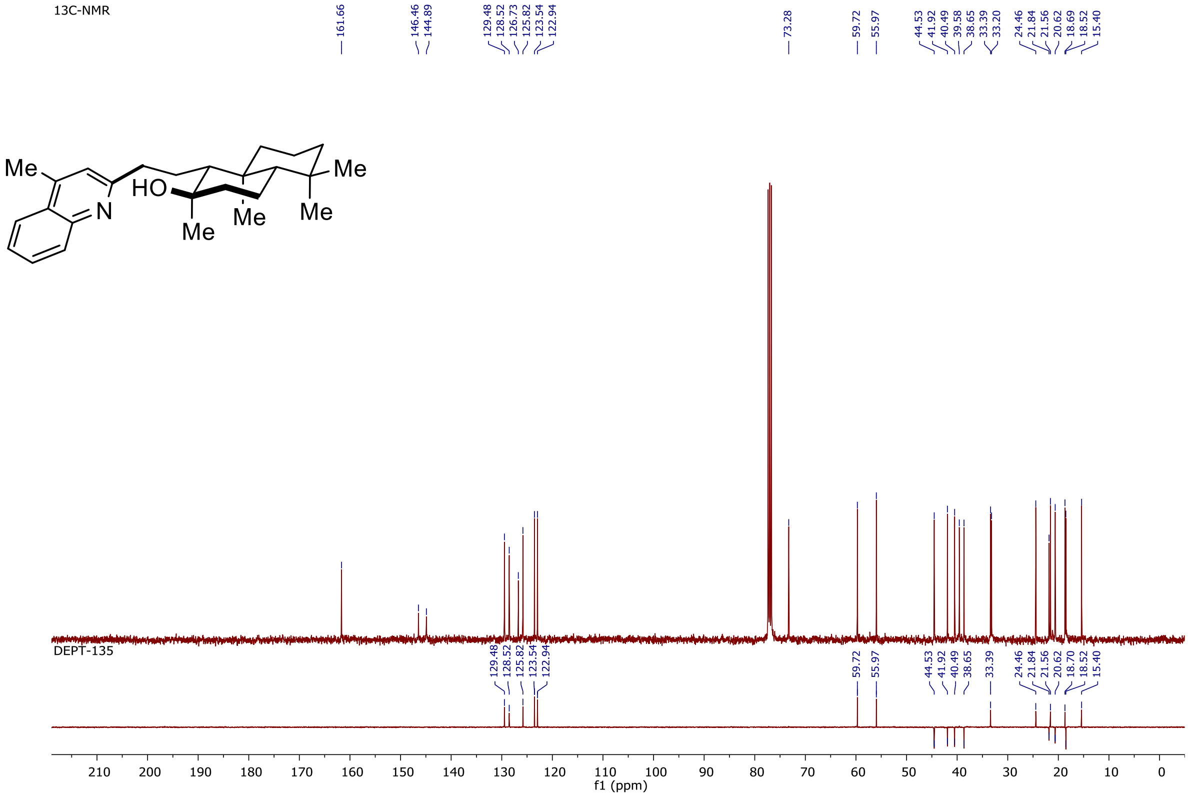

References

[1] Schlengel, M.; Qian, S.; Nicewicz, D. A. *ACS Catal.* **2022**, *12*, 10499.
[2] Wang, B.; Pettenuzzo, C. A.; Singh, J.; Mccabe, G. E.; Clark, L.; Young, R.; Pu, J.; Deng, Y. *ACS Catal.* **2022**, *12*, 10441.
[3] Nguyen, V. D.; Trevino, R.; Greco, S. G.; Arman, H. D.; Larionov, O. V. *ACS Catal*. **2022**, *12*, 8729.
[4] Zubkov, M. O.; Kosobokov, M. D.; Levin, V. V.; Dilman, A. D. *Org. Lett.* **2022**, *24*, 2354.
[5] Ramirez, N. P.; Bosque, I.; Gonzalez-Gomez, J.-C. *Org. Lett.* **2015**, *17*, 4550.
[6] Benniston, A. C.; Harriman, A.; Li, P.; Rostron, J. P.; van Ramesdonk, H. J.; Groeneveld, M. M.; Zhang, H.; Verhoeven, J. W. *J. Am. Chem. Soc.* **2005**, *127*, 16054.
[7] Nguyen, V. T.; Nguyen, V. D.; Haug, G. C.; Dang, H. T.; Jin, S.; Li, Z.; Flores-Hansen, C.; Benavides, B. S.; Arman, H. D.; Larionov, O. V. *ACS Catal.* **2019**, *9*, 9485.
[8] Chatterjee, A., König, B. *Angew. Chem. Int. Ed*. **2019**, *58*, 14289.
[9] Li, R.-J.; Xu, J.-H.; Yin, Y.-C.; Wirth, N.; Ren, J.-M.; Zeng, B.-B.; Yu, H.-L. *New J. Chem.*, **2016**, *40*, 8928.
[10] Ikarashi, G., Morofuji, T., Kano, N. *Chem. Commun.*, **2020**, *56*, 10006.
[11] Rostoll-Berenguer, J.; Martín-López, M.; Blay, G.; Pedro, J. R.; Vila, C. *J. Org. Chem*. **2022**, *87*, 9343.
[12] Ciszewski, Ł. W.; Gryko, D. *Chem. Commun.* **2022**, *58*, 10576.
[13] Zhao, H.; Jin, J. *Org. Lett.* **2019** *21*, 6179.
[14] Huang, C.-Y.; Li, J.; Li, C.-J.; *Nat. Commun.* **2021**, *12*, 4010.
[15] Ghosh, P.; Kwon, N. Y.; Byun, Y.; Mishra, N. K.; Park, J. S.; Kim, I. S. *ACS Catal*. **2022**, *12*, 15707.
[16] Wang, J.; Li, G.-X.; He, G.; Chen, G. *Asian J. Org. Chem*. **2018**, *7*, 1307.
[17] Bhakat, M.; Khatua, B.; Guin, J. *Org. Lett.* **2022,** *24*, 5276.
[18] Sherwood, T. C.; Li, N.; Yazdani A. N.; Dhar, T. G. M. *J. Org. Chem.* **2018**, *83*, 3000.
[19] Wang, M.; Zhang, Y.; Yang, X.; Sun, P. *Org. Biomol. Chem*., **2022**, *20*, 2467.
[20] Yao, L.; Zhu, D.; Wang, L.; Liu, J.; Zhang, Y.; Li, P. *Chinese Chem. Lett.* **2021**, *32*, 4033.
[21] Li, D.-S.; Liu, T.; Hong, Y.; Cao, C.-L.; Wu, J.; Deng, H.-P. *ACS Catalysis* **2022,** *12*, 4473.

[22] Rieder, S.; Meléndez, C.; Dénès, F.; Jangra, H.; Mulliri, K.; Zipse, H.; Renaud, P. *Chem. Sci.***, 2021**, 12, 15362.
[23] Zhang, J.; Kohlbouni, S. T.; Borhan, B. *Org. Lett.* **2019**, 21, 14, 5728.
[24] Okugawa, N., Moriyama, K., Togo, H., *J. Org. Chem.* **2017**, *82*, 170.
[25] Bhakat, M., Biswas, P., Dey, J., Guin, J., *Org. Lett.* **2021**, *23*, 6886.
[26] Yin, C., Hu, P., *Eur. J. Org. Chem*. **2023**, *26*, e202300015.

[27] Li, J.; Huang, C.-Y.; Han, J.-T.; Li, C.-J.; *ACS Catalysis* **2021** *11*, 14148.
[28] del Río-Rodríguez, R.; Fragoso-Jarillo, L.; Garrido-Castro, A. F.; Maestro, M. C.; Fernández-Salas, J.; Alemán, J. *Chem. Sci.*, **2022**, *13*, 6512.
